# Rethinking Borole Cycloaddition Reactivity

**DOI:** 10.1002/chem.202101290

**Published:** 2021-06-21

**Authors:** Felix Lindl, Xueying Guo, Ivo Krummenacher, Florian Rauch, Anna Rempel, Valerie Paprocki, Theresa Dellermann, Tom E. Stennett, Anna Lamprecht, Tobias Brückner, Krzysztof Radacki, Guillaume Bélanger‐Chabot, Todd B. Marder, Zhenyang Lin, Holger Braunschweig

**Affiliations:** ^1^ Institute for Inorganic Chemistry and Institute for Sustainable Chemistry & Catalysis with Boron (ICB) Julius-Maximilians-Universität Würzburg Am Hubland 97074 Würzburg Germany; ^2^ Department of Chemistry The Hong Kong University of Science and Technology Clear Water Bay, Kowloon Hong Kong P. R. China

**Keywords:** Boron, computational chemistry, isomer, isomerization, pericyclic reaction

## Abstract

Boroles are attracting broad interest for their myriad and diverse applications, including in synthesis, small molecule activation and functional materials. Their properties and reactivity are closely linked to the cyclic conjugated diene system, which has been shown to participate in cycloaddition reactions, such as the Diels‐Alder reaction with alkynes. The reaction steps leading to boranorbornadienes, borepins and tricyclic boracyclohexenes from the thermal reaction of boroles with alkynes are seemingly well understood as judged from the literature. Herein, we question the long‐established mechanistic picture of pericyclic rearrangements by demonstrating that seven‐membered borepins (i. e., heptaphenylborepin and two derivatives substituted with a thienyl and chloride substituent on boron) exist in a dynamic equilibrium with the corresponding bicyclic boranorbornadienes, the direct Diels‐Alder products, but are not isolable products from the reactions. Heating gradually converts the isomeric mixtures into fluorescent tricyclic boracyclohexenes, the most stable isomers in the series. Results from mechanistic DFT calculations reveal that the tricyclic compounds derive from the boranorbornadienes and not the borepins, which were previously believed to be intermediates in purely pericyclic processes.

## Graphical abstract

**New insights** into the reactivity of perarylated boroles toward alkynes have been derived from both experimental and computational studies. It is shown that borepins (the formal alkyne insertion products) can readily isomerize to the thermodynamically more favorable boranorbornadienes (the formal Diels‐Alder products), thus obscuring the mechanism of product formation (see Figure). In addition, the fluorescent nature of the tricyclic boracyclohexenes, the most stable isomers of the addition reactions, has been investigated by absorption and emission spectroscopy.

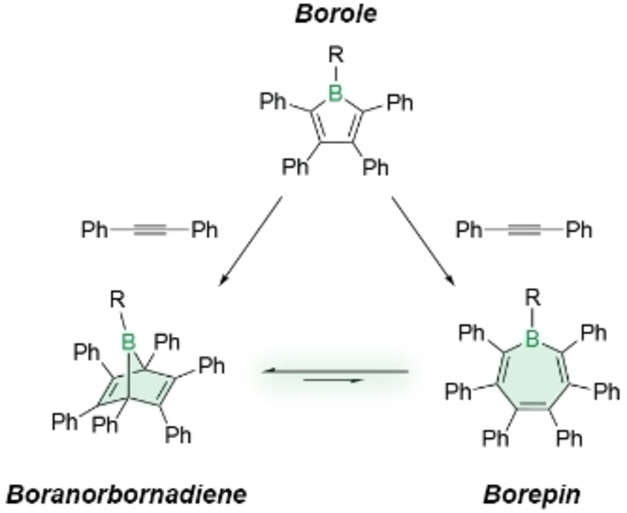

## Introduction

Over the last decade, our understanding of the properties and reactivity of boracyclopentadienes (or simply boroles) has improved substantially and they are now widely recognized as potent electron acceptors and platforms for the synthesis of a diverse range of boron heterocycles.^[1]^

**Scheme 1:**
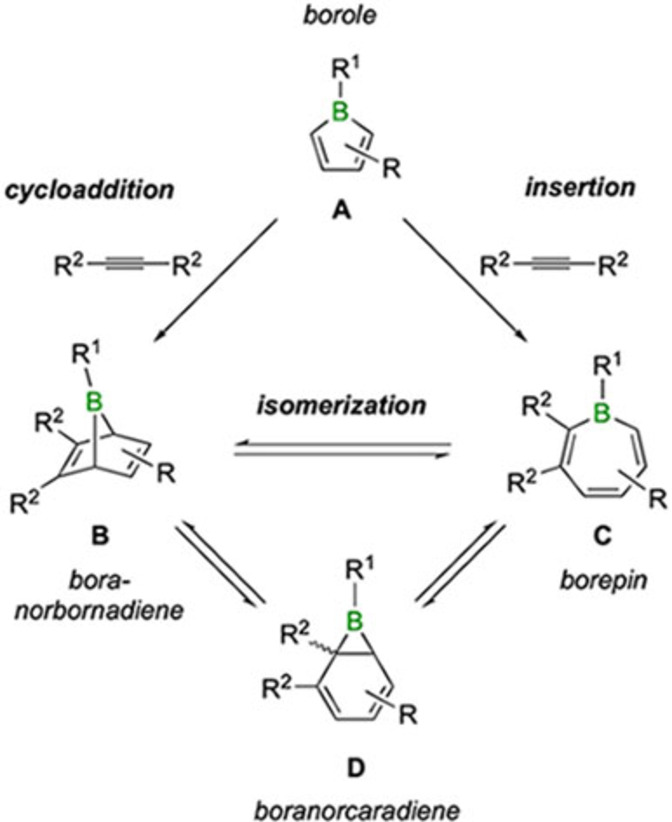
Distinct mechanisms of boranorbornadiene (**B**) and borepin (**C**) formation from boroles (**A**) and alkynes and their mutual isomerization via a boranorcaradiene intermediate (**D**). Regioisomers due to different substituents are not shown.

Among the defining characteristics of boroles is their marked propensity to undergo addition reactions. Being isoelectronic to the quintessential antiaromatic cyclopentadienyl cation ([C_5_H_5_]^+^), boroles are inherently unstable and reactive. For example, if their five‐membered ring is insufficiently sterically hindered, such as in 1‐phenyl‐2,3,4,5‐tetramethylborole, boroles react with themselves to form bicyclic 7‐boranorbornenes in a Diels‐Alder reaction.[^2^, ^3^, ^4^] Their proclivity to participate in cycloaddition reactions was first demonstrated by Eisch and co‐workers in seminal studies where they showed that pentaphenylborole (structural motif **A** in Scheme 1) undergoes a [4+2] cycloaddition with diphenylacetylene to the corresponding 7‐boranorbornadiene (structural motif **B**).^[5]^ A borepin species with a seven‐membered ring (structural motif **C**) was also reported to be a product of this transformation, and was proposed to be formed from the initial Diels‐Alder adduct via a sequence of pericyclic reactions that proceed through a boranorcaradiene intermediate (structural motif **D**).^[5]^ While direct evidence is missing for formation of a borepin in this reaction – after initial claims to the contrary – the studies of Piers and co‐workers on a highly fluorinated borole derivative lent some support to the proposed isomerization reaction (Scheme 1).^[6]^ In the reaction of perfluoropentaphenylborole with alkynes of different electronic and steric properties, borepins were clearly identified as products and, in one case, conversion of an unsymmetrically substituted boranorbornadiene into a borepin was observed by NMR spectroscopy.

Besides cycloaddition,[^7^, ^8^] boroles also undergo insertions with alkynes, a mode of reactivity that was first revealed by Piers and co‐workers with the isolation of a six‐membered boracyclohexadiene.^[6]^ This alternative reaction pathway can be viewed as a carboboration reaction in which the endocyclic B−C bond of the borole is added to the carbon‐carbon triple bond of the alkyne.^[9]^ Depending on the mode of addition, either one or two carbon atoms are added to the borole ring, resulting in six‐ and seven‐membered boracycles, respectively. Thus, the insertion pathway provides an alternative mechanism for the formation of seven‐membered borepins from the reaction of boroles with alkynes (Scheme 1). It also becomes evident that a distinction between the two mechanisms ‐ cycloaddition or insertion pathways ‐ cannot be readily made, if the borepin and boranorbornadiene isomers are readily interconverted. According to DFT calculations from one of our groups,^[10]^ this is exactly the case for the reaction of pentaphenylborole with diphenylacetylene: the perphenylated boranorbornadiene and borepin isomers are of similar energy and connected by a low barrier for isomerization. Moreover, both the cycloaddition and insertion pathways involve very similar activation barriers that are energetically accessible under the reaction conditions. Despite these insights, there still remains uncertainty about the products formed in the reaction and about possible equilibria between the isomeric products. A clearer picture exists about the nature of the most stable isomer in the reaction of pentaphenylborole with diphenylacetylene, which was identified to be a tricyclic compound consisting of a central boracyclohexene ring.^[5e]^ The latter is believed to be formed from the borepin isomer via an intramolecular ene reaction.

In this contribution we rationalize the formation of the diverse isomers accessible by the thermal reaction of boroles with alkynes by providing new insight into the mechanisms governing their formation. We demonstrate that borepins are indeed observable in equilibrium with the Diels‐Alder adducts but are not isolable species from the reactions. We further show that addition of the Lewis base *I*Me (*I*Me=1,3‐dimethylimidazol‐2‐ylidene) shifts the equilibrium to the borepin by forming the corresponding Lewis acid‐base adducts and that heating above 100 °C slowly and cleanly converts the isomeric mixtures of the base‐free boranorbornadienes and borepins into their highly fluorescent tricyclic isomers. Studies of their formation by DFT calculations suggest a mechanistic pathway that originates from the boranorbornadiene but does not involve a borepin intermediate as previously suggested.

## Results and Discussion

Our repeated failure to produce the seven‐membered heptaphenylborepin (**3a**) from the reaction of pentaphenylborole (**1a**) with diphenylacetylene[^5b^, ^5e^] prompted us to re‐examine this reaction and collect additional data for two sterically and electronically different boroles, namely 1‐thienyl‐ (**1b**)^[11]^ and 1‐chloro‐2,3,4,5‐tetraphenylborole (**1c**).^[12]^

### Synthesis of the boranorbornadienes

**Scheme 2:**
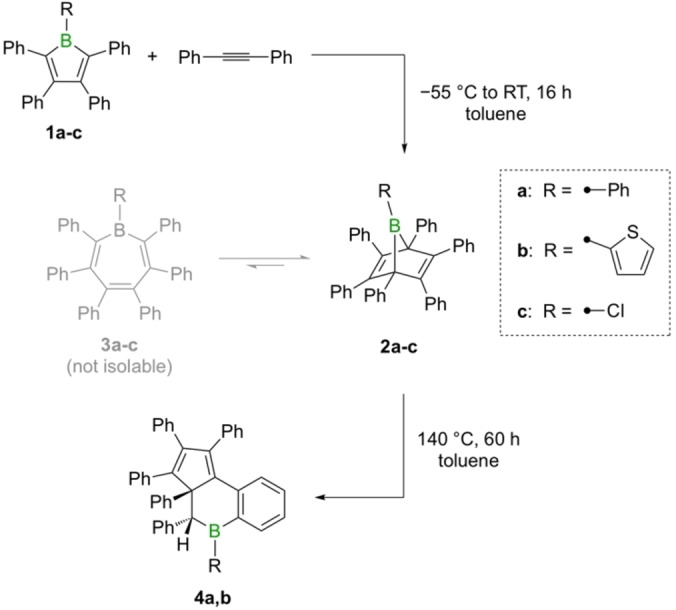
Isolable products from the thermal reaction of boroles **1a**‐**c** with diphenylacetylene.

**Figure 1:**
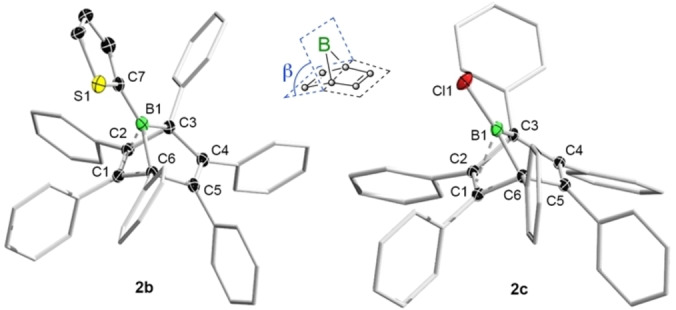
Molecular structures of compounds **2b** (left) and **2c** (right) showing 50 % probability ellipsoids. Hydrogen atoms and ellipsoids of the phenyl substituents have been omitted for clarity. The inset defines the tilt angle *β*.

As previously described by Eisch and one of our groups,[^5^, ^8^] pentaphenylborole (**1a**) reacts readily with diphenylacetylene at room temperature to give the perarylated boranorbornadiene **2a**, whose structure is confirmed by X‐ray crystallography and NMR spectroscopy (see Scheme 2). Similarly, the reactions of boroles **1b** and **1c** with diphenylacetylene lead to the respective boranorbornadienes **2b** and **2c**, indicated by the rapid fading of the initially purple solutions to a light yellow. The bicyclic products were obtained in over 80 % yield as colorless solids and characterized in both the solid and solution state. The most remarkable feature of the molecular structures of **2b** and **2c** is the pronounced tilt of the bridging boron atom towards one of the double bonds (see Figure 1). Consideration of the tilt angles in **2b** (*β*=84.9°) and **2c** (*β*=86.7°) suggests that the magnitude of the interaction is somewhat smaller than those of the phenyl derivative **2a** (*β*=83.1°)^[8]^ and its perfluorinated counterpart (*β*=83.0°).^[6]^ This is paralleled by the elongation of the respective C=C double bonds, which is less pronounced for **2b** and **2c**. The trigonal planar geometry around the boron atom is retained, with sums of bond angles of 359.9(1)° (**2b**) and 359.8(1)° (**2c**), respectively, despite the intramolecular complexation of the boron atom. The characteristic low‐frequency ^11^B NMR spectroscopic chemical shifts in benzene are consistent with the persistence of the intramolecular alkene‐borane complexation in solution. For **2b** and **2c**, the chemical shifts are −5.5 and −2.8 ppm, respectively, which compare well with those observed for other derivatives (e. g., *δ*=−4.2 ppm for **2a**).^[8]^ In addition, the bicyclic structures are readily recognized by characteristic low‐frequency ^13^C NMR signals at around *δ*=73 ppm for the bridgehead carbon atoms.^[8]^

### Equilibria between boranorbornadienes and borepins

**Figure 2:**
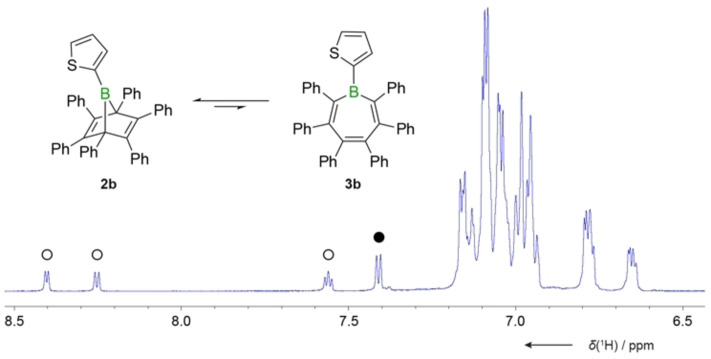
Excerpt of the ^1^H NMR spectrum of **2b** in CD_2_Cl_2_, showing the thiophene proton resonances. The open circles indicate the resonances of the borepin isomer **3b**, whereas the filled circle indicates a resonance of the bicyclic isomer **2b**.

Whereas the NMR spectra of **2a** are relatively clean, the presence of additional signals was noted for the chloro (**2c**) and the thienyl derivatives (**2b**). The latter showed an additional, broad ^11^B NMR resonance at 59.8 ppm in dichloromethane solution with about half the intensity of the major signal. By lowering the temperature to −40 °C, its corresponding ^1^H NMR intensity decreased to about a third, suggesting that the two species are in equilibrium with one another. This is supported by the dissolution of crystalline material of **2b** and the observation of two distinct sets of signals in the ^1^H NMR spectrum in the same 2 : 1 ratio (see Figure 2).

**Scheme 3:**
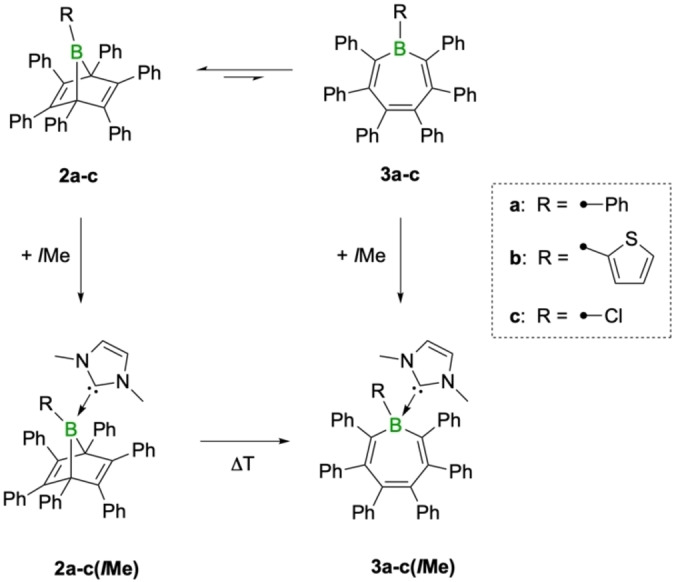
Observed equilibria between boranorbornadienes (**2a**‐**c**) and borepins (**3a**‐**c**) and conversion to their corresponding *I*Me adducts.

While crystallization results in only one isomer, namely the boranorbornadiene **2b**, analysis by heteronuclear NMR spectroscopy in solution revealed the identity of the minor isomer to be the corresponding borepin derivative, **3b** (Scheme 3). The presence of a borepin isomer (**3c**) could also be inferred from analysis of the ^13^C NMR spectrum for the chloro derivative **2c** (see Supporting Information for more details). Due to overlapping signals, the two isomers cannot be distinguished in the relatively crowded ^1^H NMR spectrum. The characteristic ^11^B NMR signal for the borepin derivative **3c** is found at *δ*=56.9 ppm (in CD_2_Cl_2_). While we were able to assign the NMR signals to each individual isomer, only **2c** proved to be an isolable product. For the perphenylated derivative, **2a**, the borepin derivative **3a** is not readily apparent in the NMR spectra, suggesting that a possible equilibrium lies strongly on the side of the boranorbornadiene. However, by addition of the Lewis base *I*Me (*I*Me=1,3‐dimethylimidazol‐2‐ylidene), the two isomers in equilibrium can be trapped in the form of their *I*Me adducts. We are well aware that base addition might affect the equilibrium, but we have not found any evidence that the boranorbornadiene adduct gets transformed into the more stable borepin adduct at room temperature. The initial ratios between the base adducts remain virtually unchanged for many hours and only start to change in favor of the borepin adduct after heating at 80 °C, in line with our previous studies.^[8]^ After addition of *I*Me to **2a** in benzene solution at room temperature, the ratio is about 17 : 1 in favor of the boranorbornadiene adduct **2a(*I*Me)**, thus implying a similar equilibrium distribution of the uncomplexed isomers.

In addition, this approach allowed us to characterize the *I*Me adducts of **2b** and **2c** spectroscopically, the latter only partially by ^11^B NMR and characteristic ^1^H NMR resonances. Their ^11^B NMR signals (**2b(*I*Me)**: *δ*=10.1 ppm; **2c(*I*Me)**: *δ*=10.2 ppm) are significantly different from those of the corresponding borepin derivatives (**3b(*I*Me)**: *δ*=−13.7 ppm; **3c(*I*Me)**: *δ*=−5.3 ppm) into which they are converted upon heating at 80 °C. While the boranorbornadiene adducts **2b(*I*Me)** and **2c(*I*Me)** proved elusive to isolation, the corresponding borepin adducts could be isolated as colorless solids in good yields (64 % and 68 %, respectively). X‐ray diffraction revealed their nonplanar boat‐shaped geometry with alternating C=C double and C−C single bonds (see Supporting Information for details). The metric parameters pertaining to the seven‐membered rings are very similar to those in **3a(*I*Me)** ^[8]^ and in the pyridine adduct of the perfluorinated analog.^[6]^ Consistent with the change in coordination of the boron atom from three to four, the bond lengths around the boron atom become longer, as illustrated by the B−Cl bond change of 1.769(2) in **2c** to 1.917(4) Å in **3c(*I*Me)**.

**Scheme 4:**
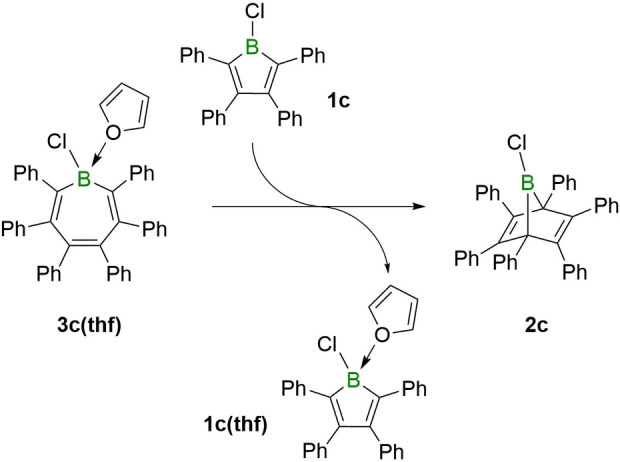
Conversion of borepin **3c(thf)** to boranorbornadiene **2c** via thf abstraction with chloroborole **1c**.

A base‐induced transformation of a boranorbornadiene into a borepin was also observed with the weak Lewis base thf. While none of the three boranorbornadiene derivatives **2a**‐**c** form Lewis acid‐base adducts with thf, the chloro derivative **2c** was nevertheless found to convert quantitatively into the corresponding borepin adduct **3c(thf)**. The reaction is likely driven by adduct formation of the borepin isomer, thus removing it from the equilibrium with the boranorbornadiene. Thf adducts of the other borepins were not observed. Borepin **3c(thf)** was characterized by multinuclear NMR spectroscopy, X‐ray crystallography and by its molecular ion peak, with the loss of thf or the chloride anion, respectively, in the same high‐resolution mass spectrum (see Supporting Information for details). Having **3c(thf)** in hand, we treated it with 1‐chloro‐2,3,4,5‐tetraphenyborole, a strong Lewis acid,^[12]^ to see if the displacement of thf re‐forms the boranorbornadiene isomer **2c**, which was expected to be the more stable isomer of the two. As anticipated, alongside the formation of the corresponding borole‐thf adduct, the formation of **2c** is readily verified by its distinct ^11^B and ^13^C NMR resonances (see Supporting Information for NMR spectra), thus further supporting the notion that borepins can readily isomerize into boranorbornadienes at room temperature (Scheme 4).

### DFT Investigations of the isomerization pathways

We rationalized the experimental results using DFT calculations, including the mechanism of interconversion between the two isomers and their relative energies. Moreover, we carried out calculations to study the effect of the *I*Me base on facilitating the ring‐opening reactions to the borepin adducts. Computed structures are labelled with a prime symbol throughout.

**Figure 3:**
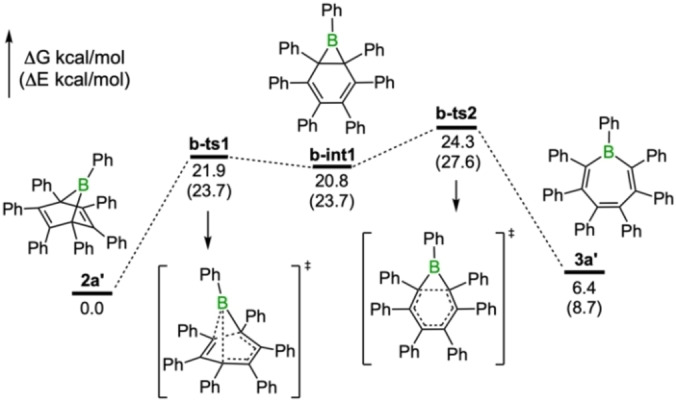
Energy profile for the interconversion of **2a’** and **3a’**. Relative free energies and electronic energies (in parentheses) are given in kcal/mol.

Our previous quantum chemical investigations into the mechanisms of formation of **2a’** and **3a’** have shown that the activation energies of the cycloaddition (for **2a’**) and insertion pathways (for **3a’**) are similar (ca. 14–15 kcal/mol) and readily surmountable at room temperature.^[10]^ These findings suggest that the ratio of the two isomers is therefore determined by the barriers for isomerization and their relative stabilities. In line with previous analysis and assumptions, we found that the two isomers, **2a’** and **3a’**, are connected through a boranorcaradiene intermediate (**b**‐**int1**, Figure 3). Two transition states are closely associated with this intermediate, which resides in a very shallow well (less than 4 kcal/mol). The respective barriers for isomerization are therefore largely governed by the relative energies of the isomers. Our calculations at the M062X‐D3/6‐31+G(d,p) level of theory indicate that the boranorbornadiene isomer **2a’** is 6.4 kcal/mol lower in energy than the borepin isomer **3a’**. Although at room temperature the calculated barriers for interconversion are slightly high (24.3 kcal/mol for **2a’**→**3a’** and 17.9 kcal/mol for **3a’**→**2a’**), they are in qualitative agreement with experimental observations that these isomers exist in equilibrium and that this equilibrium lies strongly on the side of the boranorbornadiene **2a**. This indicates that borepin **3a’**, if preferentially formed through the insertion mechanism, would be readily converted into the more stable boranorbornadiene **2a’**, thus giving a false appearance of cycloaddition reactivity of the borole. Our experimental conclusion that heptaphenylborepin **3a** is not isolable from the reaction of pentaphenylborole **1a** and diphenylacetylene, even at elevated temperatures (see below), is therefore consistent with the results from the DFT calculations. The choice of the appropriate functional proved to be crucial for these studies as the estimated energies vary greatly. Results from previous calculations with the M05‐2x‐D3 functional predicted a reversed order of stabilities for the isomers, which would thus be in conflict with the experimental data.^[10]^ A detailed overview with a brief discussion of the effects of the functionals on the relative isomer energies is given in section E in the Supporting Information.

While analogous calculations for the chloro derivative also indicate thermally accessible barriers for isomerization, we found differences in its mechanism. For **2c’** and **3c’**, the interconversion between the isomers is only a one‐step process as the putative boranorcaradiene intermediate does not correspond to a local minimum (see Supporting Information for details). Similar to the phenyl derivative **2a’**, the boranorbornadiene **2c’** is found to be more stable than its corresponding borepin isomer **3c’**, consistent with the experimental observation that only the former can be isolated.

As was previously reported and seen in the experiments, addition of the carbene *I*Me induces the transformation of the boranorbornadienes into the corresponding borepins.^[8]^ In all cases, the carbene‐borepin adducts were found to be thermodynamically more stable than the boranorbornadiene counterparts, in reversed order of stability compared to the uncomplexed forms. While the reactions follow a similar mechanism compared to that of the uncomplexed derivatives, the energy barriers involved are significantly higher. The conversion of the carbene‐boranorbornadiene adduct **2a(IMe)’** proceeds through a high‐energy transition state with a barrier of 27.3 kcal/mol to the base‐stabilized boranorcaradiene intermediate, indicating that the reaction should not readily occur at room temperature, as experimentally observed. For R=Cl, the barrier for borepin formation is even higher at 34.4 kcal/mol. The difference mainly results from greater stabilization of **2c’** versus **2a’** upon adduct formation.

Overall, the calculated barriers for interconversion and relative energies between the boranorbornadienes and borepins are consistent with the two isomers being in an equilibrium lying on the side of the boranorbornadienes. Similarly, the energetics of the base‐induced reactions are in agreement that heating is required in order to convert the boranorbornadienes **2a‐c(*I*Me)** into the thermodynamically more stable borepin isomers **3a**‐**c(*I*Me)**. The calculations are also consistent in that the boranorcaradiene intermediates are not isolable isomers in these reactions, or in their base‐stabilized forms.[^13^, ^14^, ^15^, ^16^]

### Synthesis of the tricyclic boracyclohexene isomers

**Figure 4:**
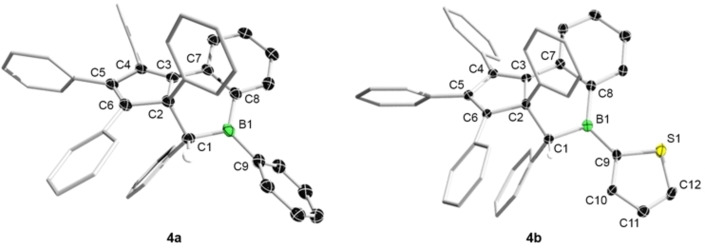
Molecular structures of the tricyclic boracyclohexenes **4a** (left) and **4b** (right) showing 50 % probability ellipsoids. Hydrogen atoms (except for the hydrogen on the methine carbon C1) and ellipsoids for the phenyl substituents are omitted for clarity.

According to the generally accepted picture of transformations,^[5]^ the initial Diels‐Alder adduct **2a** converts first to borepin **3a** and eventually to the tricyclic isomer **4a** on heating. Thus, to probe thermal reactions of the isomeric boranorbornadiene and borepin mixtures, we closely monitored their ^1^H and ^11^B NMR spectra as a function of temperature. For **2a**, even prolonged heating at 80 °C in benzene solution leads to no discernable changes in the spectra (see Supporting Information for details). Only prolonged heating of a toluene solution in an oil bath at 140 °C in a sealed tube gave rise to a new species, **4a**, which was readily identified by its characteristic singlet resonance at *δ*=4.55 ppm in the ^1^H NMR spectrum (Scheme 2). The low frequency signal can be assigned to the methine proton of the benzo‐cyclopentadiene‐fused boracyclohexene **4a**, a tricyclic compound previously described as a “pseudoborepin” by Eisch.^[5e]^ **4a** was obtained as pale yellow solid in 88 % yield and fully characterized. The X‐ray structural, NMR and optical data for **4a** are consistent with the limited characterization data available in the literature; its full characterization data are provided in the Supporting Information, its photophysical properties are discussed below. In the case of **2b**, a structural transformation occurs only after heating in refluxing toluene. After 60 h, the ^1^H NMR spectrum showed quantitative conversion to compound **4b**, which was isolated as a pale‐yellow solid in 84 % yield (Scheme 2). Diagnostic NMR features include the methine resonance at 4.73 ppm in the ^1^H NMR spectrum and the ^11^B NMR signal at 60.2 ppm, thereby identifying **4b** as being analogous to **4a**. Compound **4b** was further unambiguously characterized by X‐ray diffraction studies (see Figure 4). Similar to the structure of **4a**,^[5e]^ the central six‐membered boracyclohexene ring adopts an envelope conformation with the boron atom residing in a virtually trigonal planar geometry (Σ(C−B−C)=359.9(4)°). Despite the presence of two adjacent stereogenic centers, **4b** only exists as one diastereomer as evidenced by both single crystal X‐ray diffraction and solution NMR spectroscopy.

In contrast to **2a** and **2b**, the isomeric mixture of the chloro‐substituted derivative was found to be more reactive, leading to a thermal rearrangement at much lower temperatures. Heating of **2c/3c** at 80 °C for 15 h in benzene results in clean conversion to a new species (**4c**) without any detectable intermediates. Although a determination of its molecular structure by X‐ray diffraction has thus far eluded us, analysis of its ^13^C NMR signals revealed key structural features. Two low‐frequency ^13^C NMR spectroscopic signals at 69.7 and 80.3 ppm, indicative of saturated carbon atoms, along with the ^11^B NMR signal at 68.5 ppm, suggest a tricyclic boracyclohexene structure similar to that of **4a** and **4b**. However, due to the absence of a proton signal at lower frequency (*δ* ≈ 5 ppm) we assume that an elimination of HCl has taken place, resulting in a dimeric structure with a central four‐membered C_2_B_2_ ring (for further details, see Supporting Information). The high‐resolution mass spectrum of **4c**, which shows a monoisotopic peak at 1106.4825 m/z ([M+H_2_O]^+^), is consistent with the proposed structure.

In summary, the thermal transformations of the isomeric mixtures **2a**‐**c/3a**‐**c** to the tricyclic isomers **4a**‐**c** proceed cleanly, without detectable intermediates. We can thus conclude that borepins, including heptaphenylborepin **3a**, are not isolable products from the thermal reaction of boroles **1a**‐**c** with diphenylacetylene (Scheme 2). In addition, they do not appear to play a role in the formation of the tricyclic isomers since the decrease in intensity of the boranorbornadiene NMR signals is directly related to the increase in signal intensity for the tricyclic products. NMR spectra showing the progress of the reaction for **2a**→**4a** can be seen in Figure S86 in the Supporting Information.

### Mechanistic DFT investigations into the formation of the tricyclic isomers

**Figure 5:**
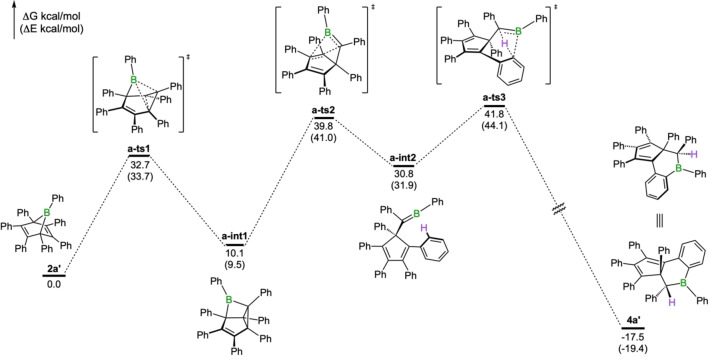
Energy profile for the formation of **4a’** from **2a’**. Computed structures and relative energies of minima and transition state structures are shown. Relative free energies and electronic energies (in parentheses) are given in kcal/mol.

To refine the conclusions drawn from our synthetic studies, we conducted DFT calculations on the mechanism of formation of the tricyclic compound **4a**. The proposed mechanism for the formation of **4a’** is shown in Figure 5. We first looked into the free energy barriers that would be required for formation of the tricyclic compound **4a’** from borepin **3a’**, in the concerted one‐step process originally proposed by Eisch and co‐workers. As the energy barrier for this transformation, which involves a boratricyclo[3.2.0.0]heptene intermediate (**a**‐**int1**) in the initial step, is predicted to be very high (Δ*G* ^≠^=44.0 kcal/mol, Figure S97), it appears unlikely. Instead, we found that the same intermediate, **a**‐**int1**, can be accessed more favorably by the boranorbornadiene **2a’**, with a free energy barrier of 32.7 kcal/mol. From this intermediate, a retro‐Diels‐Alder reaction results in an alkylideneborane intermediate (**a**‐**int2**) by passing through a transition state of high potential energy (39.8 kcal/mol). A similarly high energy transition state (41.8 kcal/mol) is involved in the product‐forming step. The tricyclic product **4a’** forms via a strongly exergonic process through intramolecular C−H activation of a peripheral phenyl ring. The stereochemistry of **4a’** is the result of a *si*‐attack of the C−H bond on the prochiral C=B double bond of the alkylideneborane.

While the activation barriers for the transformation of **2a’** to **4a’** are high, they are consistent with the reaction occurring only in toluene at 140 °C in a sealed vessel. Some support for the proposed reaction pathway is found in the dimeric product **4c** featuring a four‐membered C_2_B_2_ ring, conceivably formed through a formal cyclodimerization of two alkylideneboranes in the final step.^[17]^

### Photophysical properties of the tricyclic isomers

**Figure 6:**
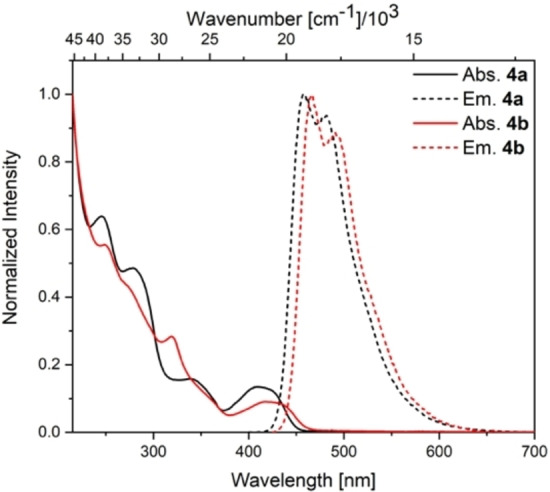
Combined absorption (solid line) and emission spectra (dashed line) of **4a** and **4b** in hexane.

The fluorescent nature of the tricyclic borane **4a** was previously observed but not further studied,^[5e]^ prompting us to investigate its photophysical properties together with those of **4b**. To this end, we measured their absorption and emission spectra, including their fluorescence quantum yield and lifetimes, and verified the results by time‐dependent density functional theory (TD‐DFT) calculations. The photophysical data of **4a** and **4b** are summarized in Table 1. The absorption spectra of **4a** and **4b** exhibit lowest energy absorption maxima at 412 and 422 nm, respectively, in hexane (see Figure 6). Increasing the solvent polarity from hexane to toluene does not influence the lowest energy absorption. In both cases, TD‐DFT calculations (see Supporting Information) suggest a HOMO to LUMO transition for S_1_←S_0_, with the largest contributions to the HOMO from cyclopentadiene and the largest contribution to the LUMO from the boron atom. The calculated orbital overlap coefficients^[18]^ (Λ=0.61 (**4a**) and 0.54 (**4b**)) suggest a weak to moderate charge transfer character. Both compounds are highly emissive, with emission maxima at 457 (**4a**) and 466 nm (**4b**), fluorescence lifetimes on the order of 10 ns and quantum yields of 0.61 (**4a**) and 0.67 (**4b**) in hexane. Both compounds exhibit a distinct vibrational progression of the emission, which is more pronounced in hexane than in toluene. The apparent Stokes shifts in hexane are 2400 and 2200 cm^−1^, respectively, and the emission maxima are slightly bathochromically shifted in more polar toluene (∼800–900 cm^−1^). This indicates a weak charge transfer character, resulting in a larger dipole moment in the excited state and an increased solvent reorganization energy in the more polar solvent. Interestingly, the quantum yields increase from hexane to toluene.

**Table 1:** Photophysical data for **4a** and **4b**.

Compound	Solvent	*λ*_abs_ [nm] (ϵ [M^−1^ cm^−1^])	*λ*_em_ [nm]	Stokes shift [cm^−1^]	τ_f_ [ns] (rel%)	*Φ*_f_
**4a**	hexane	412	457	2400	11.8	0.61
**4a**	toluene	414 (9000)	474	3100	11.3	0.75
**4b**	hexane	422	466	2200	3.3 (9 %) 9.0 (91 %)	0.67
**4b**	toluene	423 (8200)	482	2900	3.4 (7 %) 10.5 (93 %)	0.76

## Conclusion

In conclusion, our combined experimental and theoretical investigations into the thermal reaction of three different boroles with diphenylacetylene revealed that the isomerization mechanisms between boranorbornadienes, borepins and tricyclic boracyclohexenes, originally proposed by Eisch, might not be valid. While the sterically encumbered borepins were found to exist in equilibrium with the more stable boranorbornadiene isomers in solution, they are not isolable products and unlikely to play a role in the formation of the most stable, tricyclic isomers. Based on computational data, a mechanism proceeding via C−H activation by an intermediate alkylideneborane is favored over a purely pericyclic pathway. The herein established equilibrium between the boranorbornadienes and borepins supports the presence of an isomerization process that connects the products resulting from Diels‐Alder and insertion reactivity of the borole. If the barriers for this process are thermally accessible, the product distribution will be determined by the relative stability of the two isomers, thereby obscuring the mechanism of their formation. According to DFT calculations, the interconversion of the isomers proceeds via a boranorcaradiene intermediate, a well‐established isomer that is consistent with the previously proposed pericyclic mechanism.

## Experimental

Full details of synthesis, NMR characterization, crystal structure determination, photophysical studies and DFT calculations can be found in the Supporting Information.

Deposition Numbers 2050168 (for **2b**), 2050169 (for **2c**), 2050170 (for **3b(** ***I*** **Me)**), 2050171 (for **3c(** ***I*** **Me)**), 2050172 (for **3c(thf)**), 2050173 (for **4a**), and 2050174 (for **4c**) contain the supplementary crystallographic data for this paper. These data are provided free of charge by the joint Cambridge Crystallographic Data Centre and Fachinformationszentrum Karlsruhe Access Structures service www.ccdc.cam.ac.uk/structures.

## Conflict of interest

The authors declare no conflict of interest.

## Supporting information

As a service to our authors and readers, this journal provides supporting information supplied by the authors. Such materials are peer reviewed and may be re‐organized for online delivery, but are not copy‐edited or typeset. Technical support issues arising from supporting information (other than missing files) should be addressed to the authors.

Supporting Information
