# Supplementary material for: Rethinking Borole Cycloaddition Reactivity
Source: Chemistry. 2021 Jun 21;27(43):11226–33. doi: 10.1002/chem.202101290 (PMC8453799; doi:10.1002/chem.202101290)
Supplement: Supplementary file 1 — Supporting Information [file CHEM-27-11226-s001.pdf]

Chemistry–A European Journal

Supporting Information

Rethinking Borole Cycloaddition Reactivity

Felix Lindl[+], Xueying Guo[+], Ivo Krummenacher, Florian Rauch, Anna Rempel, Valerie Paprocki, Theresa Dellermann, Tom E. Stennett, Anna Lamprecht, Tobias Brückner, Krzysztof Radacki, Guillaume Bélanger-Chabot, Todd B. Marder, Zhenyang Lin,* and Holger Braunschweig*

Table of Contents

A. Experimental Section 3

A.1. General Considerations 3

A.2. Synthesis of 2b 4

A.3. Synthesis of 2c 5

A.4. Synthesis of 3b(*I*Me) 7

A.5. Synthesis of 3c(*I*Me) 9

A.6. Synthesis of 3c(thf) 10

A.7. Synthesis of 4a 11

A.8. Synthesis of 4b 13

A.9. Synthesis of 4c 14

B. NMR Spectroscopy 16

B.1. ^{1}H NMR data of isomers 2b/3b 16

B.2. ^{1}H NMR data of isomers 2c/3c 18

B.3. ^{1}H NMR data of 3b(*I*Me) 20

B.4. ^{1}H NMR data of 3c(*I*Me) 21

B.5. ^{1}H NMR data of 3c(thf) 23

B.6. ^{1}H NMR data of 4a 25

B.7. ^{1}H NMR data of 4b 26

B.7. ^{1}H NMR data of 4c 27

B.8. ^{13}C NMR data of 2b/3b 28

B.9. ^{13}C NMR data of 2c/3c 30
B.10. ^{13}C NMR data of 3b(*I*Me) 34
B.11. ^{13}C NMR data of 3c(*I*Me) 36
B.12. ^{13}C NMR data of 3c(thf) 37
B.13. ^{13}C NMR data of 4a 39
B.14. ^{13}C NMR data of 4b 41
B.15. ^{13}C NMR data of 4c 43
B.16. ^{11}B NMR data of 2b/3b 45
B.17. ^{11}B NMR data of 2c/3c 45
B.18. ^{11}B NMR data of 3b(*I*Me) 47
B.19. ^{11}B NMR data of 3c(*I*Me) 47
B.20. ^{11}B NMR data of 3c(thf) 48
B.21. ^{11}B NMR data of 4a 48
B.22. ^{11}B NMR data of 4b 49
B.23. ^{11}B NMR data of 4c 49
B.24. Chemical Equilibrium of 2b/3b 50
B.25. NMR Studies, Part One: Carbene Adduct Formation 53
a) Reaction of 2a/3a with *I*Me 53
b) Reaction of 2b/3b with *I*Me 56
c) Reaction of 2c/3c with *I*Me 62
B.26. NMR Studies, Part Two: THF Adduct Formation 64
a) Reaction of 2a/3a with THF 64
b) Reaction of 2b/3b with THF 66
c) Reaction of 2c/3c with THF 68
B.27. NMR Studies, Part Three: Irreversible Transformations into the Tricyclic Products 73
a) Conversion of 2a/3a into 4a 73
b) Conversion of 2b/3b into 4b 74
c) Conversion of 2c/3c into 4c 75
C. Crystal Structure Determination 76
D. Photophysical Studies 88
D.1. Methods 88
D.2. UV-vis Spectra of 4a and 4b 89

D.3. Combined Absorption/Excitation and Emission Spectra 90
D.4. DFT Calculations 93
a) TD-DFT results 93
b) Cartesian coordinates 98
E. Mechanistic DFT Calculations 101
a) Computational Method 101
b) Figure S97 102
c) Energy Profiles 102
c) Cartesian Coordinates 104
d) Comparative Test of Different Density Functionals 141
F. References 143

A. Experimental Section

A.1. General Considerations

All syntheses were carried out with standard Schlenk and glovebox techniques under an argon atmosphere. Solvents were dried by distillation from suitable desiccants (benzene (K), toluene (Na), dichloromethane (P_2O_5), pentane (Na/K alloy), hexane (Na/K alloy) under argon and were stored over molecular sieves. 1,2,3,4,5-pentaphenylborole,[1] 1-thienyl-2,3,4,5-tetraphenylborole,[2] 1-chloro-2,3,4,5-tetraphenylborole,[3] heptaphenyl-7-borabicyclo[2.2.1]hepta-2,5-diene[4] were synthesized according to published procedures. NMR spectra were recorded on a Bruker Avance 400 FT NMR spectrometer (^{1}H: 400 MHz; ^{11}B: 128 MHz; ^{13}C{^{1}H}: 101 MHz; ^{13}C{^{1}H},^{1}H HSQC: 101/400 MHz; ^{13}C{^{1}H},^{1}H HMBC: 101/400 MHz) and/or a Bruker Avance 500 FT NMR spectrometer (^{1}H: 500 MHz; ^{11}B: 160 MHz; ^{13}C{^{1}H}: 126 MHz; ^{13}C{^{1}H},^{1}H HSQC: 126/500 MHz; ^{13}C{^{1}H},^{1}H HMBC: 126/500 MHz) at 296 K unless otherwise specified. Chemical shifts (δ) are given in ppm and referenced against external Me_4Si (^{1}H, ^{13}C) and $BF_3{\cdot}OEt_2$ (^{11}B). IR spectra were recorded on a JASCO FT/IR-6200 spectrometer equipped with an ATR module. UV-vis absorption spectra were recorded on a JASCO V-660 UV/Vis spectrometer. Elemental analyses were obtained from an Elementar Vario MICRO cube instrument. High-resolution mass spectrometry data were obtained using a Thermo Scientific Exactive Plus

spectrometer in LIFDI mode. Solvents and reagents were purchased from Sigma-Aldrich, Alfa Aesar or Acros Organics.

A.2. Synthesis of 2b

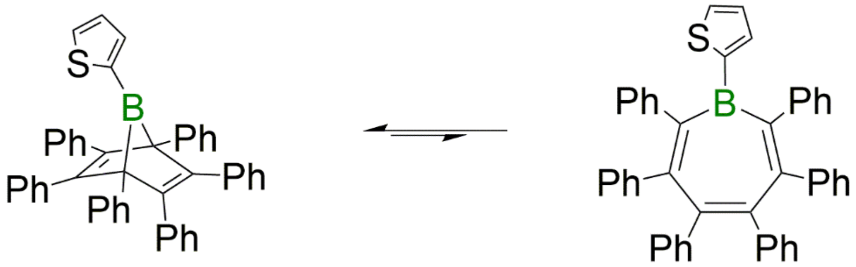

$C_{46}H_{33}BS$ (628.644 gmol^{-1})

A solution of 1-thienyl-2,3,4,5-tetraphenylborole (500 mg, 1.11 mmol) in 5 mL of benzene was treated with a solution of diphenylacetylene (297 mg, 1.67 mmol) in 5 mL of benzene at room temperature. The reaction mixture was stirred for 16 h. Over this period, a color change from deep red to yellow was observed. All volatiles were removed *in vacuo* and the resulting pale-yellow residue was washed with pentane (8 x 10 mL). **2b** was isloated as a pale yellow solid in 88% yield (616 mg, 0.98 mmol). Single crystals suitable for X-ray diffraction were grown by gas-phase diffusion of pentane into a saturated solution of **2b** in benzene.

In solution, compound **2b** (E2) exists in equilibrium with the corresponding borepin **3b** (E1).

^{1}H NMR (400 MHz, CD_2Cl_2): δ = 8.46–8.34 (m, 1H, C*H*, C_4H_3S, [E1]), 8.34–8.17 (m, 1H, C*H*, C_4H_3S, [E1]), 7.64–7.51 (m, 1H, C*H*, C_4H_3S, [E1]), 7.46–7.35 (m, 2H, C*H*, C_4H_3S, [E2]), 7.29–6.86 (m, 80H, C*H*, C_6H_5 [E1+E2] + 2H, C*H*, C_4H_3S, [E2]), 6.86–6.72 (m, 6H, C*H*, C_6H_5 [E1+E2] + 2H, C*H*, C_4H_3S, [E2]), 6.72–6.58 (m, 4H, C*H*, C_6H_5 [E1+E2]) ppm.

Comment: Two thiophene resonances of the boranorbornadiene (E2) overlap with resonances of the phenyl ring substituents. These were identified by means of ^{1}H,^{1}H COSY NMR experiments.

^{1}H,^{1}H COSY NMR (400 MHz, CD_2Cl_2): δ = 6.97 (C*H*, C_4H_3S, [E2]) ppm.

^{1}H,^{1}H COSY NMR (400 MHz, CD_2Cl_2): δ = 6.77 (C*H*, C_4H_3S, [E2]) ppm.

Comment: The sum of the reported integrals corresponds to three equivalents of compounds E1/E2 in chemical equilibrium in CD_2Cl_2 at room temperature; two for E2 and one for borepin E1. The position of the equilibrium was estimated by integration of characteristic thienyl proton resonances.

^{13}C{^{1}H} NMR (101 MHz, CD_2Cl_2): δ = 149.2 (C_q, C_B, C_4H_3S, [E2])$^{\#}$, 145.1 (*C*H, 1C, C_4H_3S, [E1]), 144.3 (C_q, [E1]), 142.6 (C_q, C_B, C_4H_3S, [E1])$^{\#}$, 141.2 (C_q, [E2]), 140.7 (*C*H, 1C, C_4H_3S, [E1]), 140.1 (C_q, [E1]), 138.4 (C_q, C_B, [E1]), 138.3 (C_q, [E2]), 138.1 (C_q, [E1]), 137.5 (*C*H, 1C, C_4H_3S, [E2]), 137.3 (C_q, [E1]), 137.1 (C_q, [E1] + C_q, [E2]), 132.7 (*C*H), 131.2 (*C*H), 130.9 (*C*H), 130.8 (*C*H), 130.5 (*C*H, 1C, C_4H_3S, [E2]), 130.3 (*C*H, 1C, C_4H_3S, [E1]), 130.1 (*C*H), 128.2 (*C*H), 128.0 (*C*H), 128.0 (*C*H), 127.6 (*C*H, 1C, C_4H_3S, [E2]), 127.6 (*C*H), 127.1 (*C*H), 126.9 (*C*H), 126.8 (*C*H), 126.6 (*C*H), 126.1 (*C*H), 125.9 (*C*H), 72.6 (C_q, C_B, [E2]) ppm.

Comment: The signals marked with a hash symbol (#) can be assigned to resonances of the boron-bound quaternary carbon atoms of the thienyl units.

^{11}B NMR (128 MHz, CD_2Cl_2): δ = 59.8 [E1], –5.5 [E2] ppm.

HRMS (LIFDI): *m/z* calcd. for $C_{46}H_{33}B_1S_1$ ($[M]^+$): 627.2427, 628.2391, 629.2424, 630.2458, 631.2382; found: 627.2426, 628.2389, 629.2419, 630.2450, 631.2376.

A.3. Synthesis of 2c

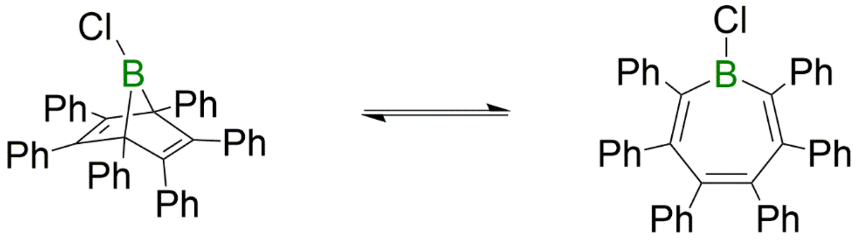

$C_{42}H_{30}BCl$ (580.963 gmol^{-1})

A solution of 1-chloro-2,3,4,5-tetraphenylborole (2.14 g, 5.32 mmol) in 10 mL of toluene was treated at −55 °C with a solution of diphenylacetylene (1.14 g, 6.38 mmol) in 10 mL of toluene. The reaction mixture was warmed to room temperature and stirred for 16 h. A color change from deep purple to red was observed concomitant with the formation of a colorless precipitate. The solvent was removed to about one-third *in vacuo* followed by the addition of 20 mL of pentane. The resulting suspension was filtered and the colorless precipitate washed with pentane (5 x 10 mL). **2c** was isolated as a colorless solid in 83% yield (1.36 g, 2.34 mmol). Single crystals suitable for X-ray diffraction were grown by gas-phase diffusion of pentane into a saturated solution of **2c** in benzene.

Alternative method

1-Chloro-2,3,4,5-tetraphenylborole (500 mg, 1.24 mmol) and diphenylacetylene (266 mg, 1.49 mmol) were dissolved in benzene (4 mL) at room temperature and stirred for 7 h. A color change from deep purple to red was observed. In addition, a colorless solid precipitated after ca. 2 h. The solvent was removed *in vacuo* and the residue washed with pentane (8 x 10 mL), dissolved in benzene and filtered. The solvent was evaporated and **2c** was isolated in 86% yield (630 mg, 1.07 mmol).

In solution, compound **2c** exists in equilibrium with the corresponding borepin **3c**.

<u>^{1}H NMR (500 MHz, C_6D_6):</u> δ = 7.35–7.20 (m, 7H, C*H*, C_6H_5), 7.20–7.09 (m, 13H, C*H*, C_6H_5 + solvent residue)†, 7.01–6.48 (m, 40H, C*H*, C_6H_5) ppm.

Comment: The set of signals marked with a cross (†) includes the solvent resonance of C_6D_6. The resonances cannot be unambiguously assigned to the individual isomers.

<u>^{1}H NMR (400 MHz, CD_2Cl_2):</u> δ = 7.13–6.94 (m, 88H, C*H*, C_6H_5), 6.94–6.83 (m, 18H, C*H*), 6.83–6.68 (m, 44H, C*H*, C_6H_5) ppm.

<u>^{13}C{^{1}H} NMR (126 MHz, C_6D_6):</u> δ = 150.5 (*C*$_q$, 2C)*, 143.8 (*C*$_q$, 2C)*, 141.2 (*C*$_q$, 2C)*, 141.1 (*C*$_q$, 2C)*, 139.7 (*C*$_q$, 2C)*, 137.6 (*C*$_q$, 4C)$^\#$, 136.4 (*C*$_q$, 2C)$^\#$, 136.4 (*C*$_q$, 4C)$^\#$, 135.2 (*C*$_q$, 2C$_B$)*†, 132.9 (*C*H), 131.2 (*C*H), 130.4 (*C*H), 130.4 (*C*H), 129.8 (*C*H), 128.0 (*C*H), 127.9

(CH), 127.8 (CH), 127.3 (CH), 127.2 (CH), 127.0 (CH), 126.5 (CH), 126.5 (CH), 126.5 (CH), 126.0 (CH) 72.5 (C_q, 2C_B)$^{\#\dagger}$.

<u>$^{13}C\{^1H\}$ NMR (101 MHz, CD_2Cl_2):</u> δ = 152.3 (C_q, 2C)*, 144.1 (C_q, 2C)*, 141.7 (C_q, 2C)*, 141.5 (C_q, 2C)*, 140.9 (C_q, 2C)*, 137.3 (C_q, 4C)$^\#$, 136.4 (C_q, 2C)$^\#$, 136.3 (C_q, 4C)$^\#$, 134.6 (C_q, 2C_B)*†, 132.9 (CH), 131.3 (CH), 130.4 (CH), 130.3 (CH), 129.7 (CH), 127.9 (CH), 127.6 (CH), 127.2 (CH), 127.1 (CH), 126.8 (CH), 126.4 (CH), 126.1 (CH), 126.1 (CH), 126.1 (CH), 126.4 (CH) 72.2 C_q, 2C_B)$^{\#\dagger}$.

Comment: The signals marked with a cross (†) are resonances of boron-bound quaternary carbon atoms. These signals were detected by means of ^{13}C DEPT-135/$^{13}C\{^1H\}$,^{1}H HMBC NMR experiments. The signals marked with an asterisk (*) can unambiguously be assigned to the borepin **3c**, whereas the signals marked with a hash symbol (#) can unambiguously be assigned to the boranorbornadiene **2c**. The expected total number of 10 quaternary carbon atom and 15 CH-bound carbon atom resonances were found for the two isomers.

<u>^{11}B NMR (128 MHz, C_6D_6):</u> δ = 60.3 (**3c**), –2.1 (**2c**) ppm.

<u>^{11}B NMR (128 MHz, CD_2Cl_2):</u> δ = 56.9 (**3c**), –2.8 (**2c**) ppm.

<u>Anal.</u> calcd. for $C_{42}H_{30}BCl$: C 86.83, H 5.20; found: C 86.92, H 5.40.

<u>HRMS (LIFDI):</u> *m/z* calcd. for $C_{42}H_{30}B_1Cl_1$ ($[M]^+$): 579.2160, 580.2124, 581.2157, 582.2094, 583.2128; found: 579.2159, 580.2120, 581.2154, 582.2089, 583.2125.

A.4. Synthesis of 3b(*I*Me)

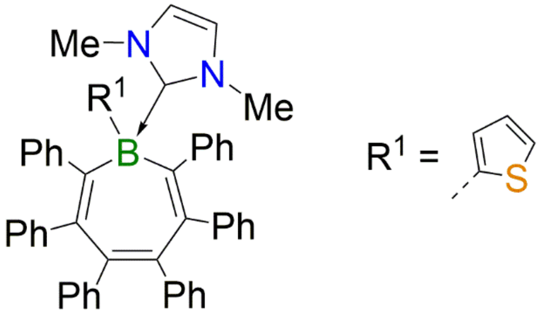

$C_{51}H_{41}N_2BS$ (724.775 $gmol^{-1}$)

A suspension of **2b** (43.0 mg, 0.07 mmol) in 0.5 mL of benzene was treated dropwise at room temperature with a solution of *I*Me (7.1 mg, 0.07 mmol) in 0.5 mL of benzene. The mixture was stirred for 7 d at 80 °C, during which a colorless solid precipitated. The resulting suspension was filtered and the colorless precipitate was washed with pentane (5 x 3 mL). **3b(*I*Me)** was isolated as a colorless solid in 64% yield (31.8 mg, 0.04 mmol). Single crystals suitable for X-ray diffraction were grown by gas-phase diffusion of pentane into a saturated solution of **3b(*I*Me)** in benzene.

^{1}H NMR (400 MHz, C_6D_6): δ = 7.14–6.76 (m, 20H, C*H*, C_6H_5 + 1H, C*H*, C_4H_3S), 6.76–6.68 (m, 4H, C*H*, C_6H_5), 6.68–6.61 (m, 2H, C*H*, C_6H_5 + 1H, C*H*, C_4H_3S), 6.58–6.49 (m, 4H, C*H*, C_6H_5), 5.83 (s, 2H, C*H*, NCN(C*H*)$_2$), 5.15–5.05 (m, 1H, C*H*, C_4H_3S), 3.70 (s, 6H, CH_3, (NCH_3)$_2$) ppm.

^{1}H,^{1}H COSY NMR (400 MHz, C_6D_6): δ = 6.99 (C*H*, C_4H_3S) ppm.

^{1}H, ^{1}H COSY NMR (400 MHz, C_6D_6): δ = 6.64 (C*H*, C_4H_3S) ppm.

^{13}C{^{1}H} NMR (101 MHz, C_6D_6): δ = 170.3 (C_q, 1C, N*C*N(CH)$_2$)†, 144.3 (C_q, 2C), 144.7 (C_q, 2C), 144.4 (C_q, 2C), 143.3 (C_q, 2C), 141.7 (C_q, 2C_B)†, 137.7 (C_q, 2C), 134.1 (*C*H, 1C, C_4H_3S), 132.6 (*C*H, 4C), 132.2 (C_q, 1C, C_4H_3S), 132.1 (*C*H, 4C), 130.9 (*C*H, 4C), 128.4 (*C*H, 1C, C_4H_3S), 127.2 (*C*H, 4C), 126.8 (*C*H, 4C), 125.6 (*C*H, 4C), 125.5 (*C*H, 2C), 125.3 (*C*H, 2C), 124.7 (*C*H, 2C), 122.9 (*C*H, 1C, C_4H_3S), 122.0 (*C*H, 2C, NCN(*C*H)$_2$), 38.4 (*C*H$_3$, 2C, (N*C*H$_3$)$_2$) ppm.

Comment: The signals marked with a cross (†) are resonances of boron- and nitrogen-bound quaternary carbon atoms. These signals were detected by means of ^{13}C DEPT-135/^{13}C{^{1}H},^{1}H HMBC NMR experiments.

^{11}B NMR (128 MHz, C_6D_6): δ = –13.7 ppm.

HRMS (LIFDI): *m/z* calcd. for $C_{51}H_{41}N_2BS$ ($[M]^+$): 723.3114, 724.3078, 725.3112, 726.3145, 727.3179; found: 723.3102, 724.3061, 725.3096, 726.3142, 727.3170.

A.5. Synthesis of 3c(*I*Me)

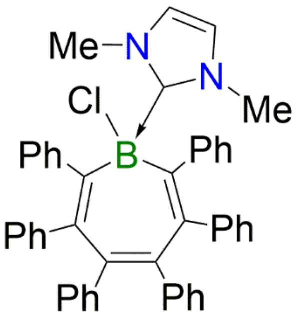

$C_{47}H_{38}N_2BCl$ (677.095 gmol^{-1})

A suspension of **2c** (43.0 mg, 0.07 mmol) in 0.5 mL of benzene was treated dropwise at room temperature with a solution of *I*Me (7.1 mg, 0.07 mmol) in 0.5 mL of benzene whereupon the formation of a clear solution was observed. The mixture was stirred for 14 h at room temperature, during which a colorless solid precipitated. The suspension was filtered and the colorless precipitate was washed with pentane (5 x 3 mL). **3c(*I*Me)** was isolated as a colorless solid in 68% yield (34.0 mg, 0.05 mmol). Single crystals suitable for X-ray diffraction were grown by gas-phase diffusion of pentane into a saturated solution of **3c(*I*Me)** in benzene.

^{1}H NMR (500 MHz, C_6D_6): δ = 7.29–7.19 (m, 5H, C*H*, C_6H_5), 7.07–6.97 (m, 6H, C*H*, C_6H_5), 6.94–6.76 (m, 6H, C*H*, C_6H_5), 6.74–6.67 (m, 5H, C*H*, C_6H_5), 6.67–6.60 (m, 3H, C*H*, C_6H_5), 6.50–6.36 (m, 5H, C*H*, C_6H_5), 5.64 (s, 2H, C*H*, NCN(C*H*)$_2$), 3.70 (s, 6H, C*H*$_3$, (NC*H*$_3$)$_2$) ppm.

^{13}C{^{1}H} NMR (126 MHz, C_6D_6): δ = 165.4 (C_q, 1C, N*C*N(CH)$_2$)†, 144.4 (C_q, 2C), 144.2 (C_q, 2C), 143.9 (C_q, 2C), 142.7 (C_q, 2C), 141.3 (C_q, 2C_B)†, 137.6 (C_q, 2C), 131.9 (*C*H, 6C),

131.8 (*C*H, 6C), 127.4 (*C*H, 6C), 126.9 (*C*H, 6C), 125.7 (*C*H, 2C), 125.5 (*C*H, 2C), 125.2 (*C*H, 2C), 121.6 (*C*H, 2C, NCN(*C*H)$_2$), 38.1 (*C*H$_3$, 2C, (N*C*H$_3$)$_2$) ppm.

Comment: The signals marked with a cross (†) are resonances of boron- and nitrogen-bound quaternary carbon atoms. These signals were detected by means of ^{13}C DEPT-135/^{13}C{^{1}H},^{1}H HMBC NMR spectroscopy.

<u>^{11}B NMR (128 MHz, C_6D_6):</u> δ = –5.3 ppm.

<u>HRMS (LIFDI):</u> *m/z* calcd. for $C_{47}H_{37}BN_2Cl$ ([M–H]$^+$): 674.2769, 675.2733, 676.2766, 677.2703, 678.2737, 679.2770; found: 674.2768, 675.2726, 676.2765, 677.2765, 678.2733, 679.2782; *m/z* calcd. for $C_{47}H_{38}BN_2$ ([M–Cl]$^+$): 640.3159, 641.3123, 642.3156, 643.3190, 644.3223; found: 640.3156, 641.3120, 642.3153, 643.3186, 644.3220.

A.6. Synthesis of 3c(thf)

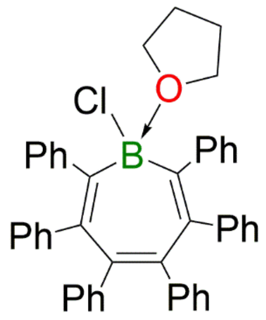

$C_{46}H_{38}BClO$ (653.070 gmol^{-1})

A solution of **2c** (30.0 mg, 0.05 mmol) in 0.6 mL of C_6D_6 was treated with tetrahydrofuran (8.00 mg, 0.12 mmol, 34 µL) at ambient temperature and stirred for 16 h at this temperature. ^{11}B NMR spectroscopy confirmed quantitative conversion to **3c(thf)**. Single crystals suitable for X-ray diffraction were grown by slow evaporation of the reaction mixture.

Comment: Due to decomposition of **3c(thf)** in vacuum, not all residual tetrahydrofuran could be removed.

<u>^{1}H NMR (500 MHz, C_6D_6):</u> δ = 7.22–7.10 (m, 8H, CH, C_6H_5 + solvent residue), 7.07–6.92 (m, 8H, CH, C_6H_5), 6.88–6.72 (m, 12H, CH, C_6H_5), 6.72–6.62 (m, 2H, CH, C_6H_5), 4.12–3.64 (m, 10H, CH, C_4H_8)$^{*\#}$, 1.61–1.15 (m, 10H, CH, C_4H_8)*.

Comment: The signals marked with an asterisk (*) correspond to the added tetrahydrofuran (ca. 2.5 equiv). Rapid exchange between bound and free tetrahydrofuran can be expected. The signal labeled with a hash symbol (#) is assigned to the 2,5-position of coordinated thf, which is shifted to lower frequencies compared to free thf.

<u>^{13}C{^{1}H} NMR (101 MHz, C_6D_6):</u> δ = 152.0 (C_q, 2C_B), 145.0 (C_q, 2C), 143.3 (C_q, 2C), 142.8 (C_q, 2C), 142.7 (C_q, 2C), 142.0 (C_q, 2C), 131.9 (*C*H, 4C), 131.6 (*C*H, 4C), 130.9 (*C*H, 4C), 127.3 (*C*H, 4C), 127.0 (*C*H, 4C), 126.9 (*C*H, 4C), 125.8 (*C*H, 2C), 125.7 (*C*H, 2C), 125.0 (*C*H, 2C), 70.5 (CH_2, C_4H_8O), 25.5 (CH_2, C_4H_8O) ppm.

<u>^{11}B NMR (160 MHz, C_6D_6):</u> δ = 11.6 ppm.

<u>HRMS (LIFDI):</u> *m/z* calcd. for $C_{46}H_{39}BO$ ($[M–Cl+H]^+$): 617.3125, 618.3088, 619.3122, 620.3156, 621.3189; found: 617.3118, 618.3077, 619.3112, 620.3144, 621.3178; *m/z* calcd. for $C_{42}H_{30}BCl$ ($[M–C_4H_8O]^+$): 579.2160, 580.2124, 581.2157, 582.2094, 583.2128, 584.2161; found: 579.2148, 580.2107, 581.2139, 582.2080, 583.2113, 584.2146.

A.7. Synthesis of 4a

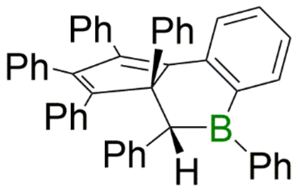

$C_{48}H_{35}B$ (622.616 gmol^{-1})

The synthesis of **4a** was previously reported but the characterization data was incomplete.[5]

A suspension of **2a** (60.0 mg, 0.10 mmol) in 1.5 mL of toluene was heated in an oil bath at 140 °C in a closed vessel for 60 h, during which the color gradually changed from colorless to fluorescent yellow-green. After cooling the reaction solution to room temperature, all volatiles were removed *in vacuo*. The remaining residue was washed with pentane (5 x 3 mL), yielding a pure, pale yellow solid (53.0 mg, 0.09 mmol, 88%). Single crystals suitable for X-ray diffraction were grown by gas-phase diffusion of pentane into a saturated solution of **4a** in benzene.

<u>^{1}H NMR (500 MHz, C_6D_6):</u> δ = 8.04–7.97 (m, 1H, C*H*, C_6H_4/C_6H_5), 7.77–7.66 (m, 4H, C*H*, C_6H_4/C_6H_5), 7.38–7.23 (m, 3H, C*H*, C_6H_4/C_6H_5), 7.23–7.18 (m, 3H, C*H*, C_6H_4/C_6H_5), 7.13–7.04 (m, 6H, C*H*, C_6H_4/C_6H_5), 7.04–6.96 (m, 4H, C*H*, C_6H_4/C_6H_5), 6.96–6.90 (m, 1H, C*H*, C_6H_4/C_6H_5), 6.90–6.66 (m, 12H, C*H*, C_6H_4/C_6H_5), 4.55 (s, 1H, C*H*Ph) ppm.

<u>^{13}C{^{1}H} NMR (126 MHz, C_6D_6):</u> δ = 154.4 (C_q, 1C), 150.3 (C_q, 1C), 146.5 (C_q, 1C), 143.9 (C_q, 1C_B)†, 143.9 (C_q, 1C), 143.3 (C_q, 1C), 142.2 (C_q, 1C), 139.9 (*C*H, 1C), 137.7 (*C*H, 1C_B), 137.0 (C_q, 1C), 136.7 (C_q, 1C), 136.4 (C_q, 1C), 136.2 (C_q, 1C), 134.2 (*C*H, 2C), 133.6 (*C*H, 1C), 131.6 (*C*H, 2C), 130.3 (*C*H, 2C), 130.1 (*C*H, 1C), 129.9 (*C*H, 2C), 129.9 (*C*H, 2C), 129.2 (*C*H, 2C), 128.8 (*C*H, 2C), 128.4 (*C*H, 1C), 128.2 (*C*H, 2C)$^\#$, 128.2 (*C*H, 2C)$^\#$, 128.1 (*C*H, 2C)$^\#$, 127.8 (*C*H, 2C)$^\#$, 127.5 (*C*H, 2C), 127.4 (*C*H, 1C), 127.3 (*C*H, 1C), 126.9 (*C*H, 1C), 126.8 (*C*H, 2C), 126.8 (*C*H, 1C), 69.6 (C_q, 1C), 47.6 (*C*H, 1C, *C*HPh) ppm.

Comment: The signal marked with a cross (†) is a resonance of a boron-bound quaternary carbon atom, which partly overlaps with another signal. However, this signal was identified with the help of an HMBC experiment. The signals marked with a hash symbol (#) overlap with resonances from the solvent but they could be identified using a ^{13}C DEPT-135 experiment.

<u>^{11}B NMR (128 MHz, C_6D_6):</u> δ = 69.1 ppm.

<u>UV-vis (Et_2O):</u> λ_{max} = 249 nm, 279 nm, 341 nm, 410 nm.

HRMS (LIFDI): *m/z* calcd. for $C_{48}H_{35}B$ ($[M]^+$): 624.2863, 622.2826, 623.2860, 624.2893, 625.2927; found: 621.2856, 622.2820, 623.2846, 624.2880, 625.2913.

A.8. Synthesis of 4b

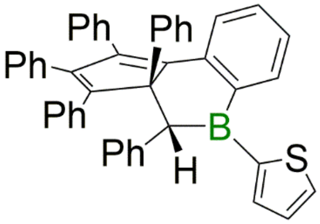

$C_{46}H_{33}BS$ (628.644 gmol^{-1})

A solution of **2b** (40.0 mg, 0.06 mmol) in 1.5 mL of toluene was warmed in an oil bath to 140 °C in a closed vessel and kept at this temperature for 60 h, upon which the color changed from colorless to fluorescent yellow-green. After cooling to room temperature, all volatiles were removed *in vacuo*. The remaining residue was washed with a 1:1 mixture of hexane and diethyl ether (5 x 3 mL), yielding a pure, pale yellow solid (34.0 mg, 0.05 mmol, 85%). Single crystals suitable for X-ray diffraction were grown by gas-phase diffusion of pentane into a saturated solution of **4b** in benzene.

^{1}H NMR (500 MHz, C_6D_6): δ = 8.66–8.50 (m, 1H, C*H*, Aryl), 7.97–7.83 (m, 1H, C*H*, Aryl), 7.83–7.64 (m, 2H, C*H*, Aryl), 7.38–7.19 (m, 4H, C*H*, Aryl), 7.13–7.04 (m, 6H, C*H*, Aryl), 7.04–6.92 (m, 4H, C*H*, C_6H_4/C_6H_5), 6.92–6.72 (m, 14H, C*H*, Aryl), 4.73 (s, 1H, C*H*Ph) ppm.
^{13}C{^{1}H} NMR (126 MHz, C_6D_6): δ = 154.0 (C_q, 1C), 150.5 (C_q, 1C), 146.6 (C_q, 1C), 143.6 (C_q, 1C), 143.6 (C_q, 1C_B)†, 143.4 (C_q, 1C), 142.2 (C_q, 1C), 140.3 (*C*H, 1C), 138.5 (*C*H, 1C) 137.7 (C_q, 1C), 137.2 (C_q, 1C_B), 136.8 (C_q, 1C), 136.8 (C_q, 1C), 136.3 (C_q, 1C), 135.8 (*C*H, 1C), 133.4 (*C*H, 1C), 131.3 (*C*H, 2C), 130.3 (*C*H, 2C), 130.0 (*C*H, 2C), 129.9 (*C*H, 2C), 129.3 (*C*H, 1C), 129.2 (*C*H, 2C), 128.8 (*C*H, 1C), 128.7 (*C*H, 2C), 128.3 (*C*H, 2C)$^\#$, 127.9 (*C*H, 2C)$^\#$, 127.8 (*C*H, 2C)$^\#$, 127.5 (*C*H, 2C), 127.4 (*C*H, 1C), 127.2 (*C*H, 1C), 126.9 (*C*H, 1C), 126.8 (*C*H, 1C), 126.8 (*C*H, 1C), 126.6 (*C*H, 1C), 68.9 (C_q, 1C), 46.6 (*C*H, 1C, *C*HPh) ppm.

Comment: The signal marked with a cross (†) is a resonance of a boron-bound quaternary carbon atom, which partly overlaps with another signal. However, this signal could be identified with the help of an HMBC experiment. The signals marked with a hash symbol (#) overlap with resonances of the solvent but they could be identified by means of a ^{13}C DEPT-135 experiment.
<u>^{11}B NMR (128 MHz, C_6D_6):</u> δ = 60.2 ppm.
<u>UV-vis (Et_2O):</u> λ_{max} = 251, 273, 321, 422.
<u>HRMS (LIFDI):</u> *m/z* calcd. for $C_{46}H_{33}B_1S_1$ ([M]$^+$): 627.2427, 628.2391, 629.2424, 630.2458, 631.2382; found: 627.2424, 628.2387, 629.2415, 630.2448, 631.2374.

A.9. Synthesis of 4c

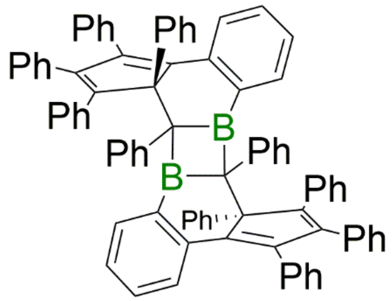

$C_{84}H_{58}B_2$ (1089.004 gmol^{-1})

A suspension of **2c** (40.0 mg, 0.07 mmol) in 0.8 mL of benzene was heated at 80 °C for 15 h, resulting in a slight yellow colored solution. After cooling to room temperature, all volatiles were removed *in vacuo*. The remaining pale yellow residue was extracted with 1.0 mL of hexane. The slightly yellow colored suspension was filtered and the filtrate dried under reduced pressure. The proposed dimeric constitution of **4c** was inferred from NMR spectroscopic data and high-resolution mass spectrometric analysis.

<u>^{1}H NMR (500 MHz, C_6D_6):</u> δ = 8.08–7.96 (m, 4H, C*H*, C_6H_4/C_6H_5), 7.49–7.35 (m, 12H, C*H*, C_6H_4/C_6H_5), 7.15–6.41 (m, 42H, C*H*, C_6H_4/C_6H_5) ppm.
<u>^{13}C{^{1}H} NMR (126 MHz, C_6D_6):</u> δ = 181.4 (C_q, 2C), 150.2 (C_q, 2C_B)†, 147.5 (C_q, 2C), 143.0 (C_q, 2C_B), 140.0 (C_q, 2C), 139.4 (C_q, 2C), 138.4 (C_q, 2C), 138.2 (*C*H, 2C), 135.9 (*C*H, 2C),

134.3 (C_q, 2C), 131.3 (CH, 4C), 131.0 (CH, 4C), 130.0 (CH, 4C), 129.7 (CH, 4C), 129.0 (CH, 4C), 128.7 (CH, 2C), 128.7 (CH, 2C), 128.7 (CH, 4C), 128.6 (CH, 6C), 128.5 (CH, 4C), 128.2 (CH, 2C)$^{\#}$, 128.0 (CH, 4C)$^{\#}$, 128.0 (CH, 4C)$^{\#}$, 127.8 (CH, 4C), 126.9 (CH, 2C), 126.7 (CH, 2C), 126.1 (CH, 2C), 80.3 (C_q, 2C), 69.8 (C_q, 2C_B), 127.3 (CH, 1C), 126.9 (CH, 1C), 126.8 (CH, 2C), 126.8 (CH, 1C), 69.6 (C_q, 1C), 47.6 (CH, 1C, CHPh) ppm.

Comment: The signals marked with a cross (†) are resonances of a boron-bound quaternary carbon atoms and were identified with the help of ^{1}H,^{13}C HMBC NMR spectroscopy. The signals marked with a hash symbol (#) overlap with solvent resonances and were therefore identified by means of a ^{13}C DEPT-135 experiment. One resonance for a quaternary carbon atom was not detected because it is likely obscured by the solvent signal.

^{11}B NMR (128 MHz, C_6D_6): δ = 69.1 ppm.

HRMS (LIFDI): *m/z* calcd. for $C_{84}H_{60}B_2O_1$ ([M+H_2O]$^+$): 1104.4897, 1105.4861, 1106.4825, 1107.4858, 1108.4892, 1109.4925, 1110.4959; found: 1104.4900, 1105.4854, 1106.4820, 1107.4850, 1108.4881, 1109.4920, 1110.4953. *m/z* calcd. for $C_{84}H_{62}B_2O_2$ ([M+2H_2O]$^+$): 1122.5003, 1123.4967, 1124.4930, 1125.4964, 1126.4998, 1127.5031, 1128.5065; found: 1122.5012, 1123.4964, 1124.4935, 1125.4960, 1126.4993, 1127.5034, 1128.4754.

B. NMR Spectroscopy

B.1. ^{1}H NMR data of isomers 2b/3b

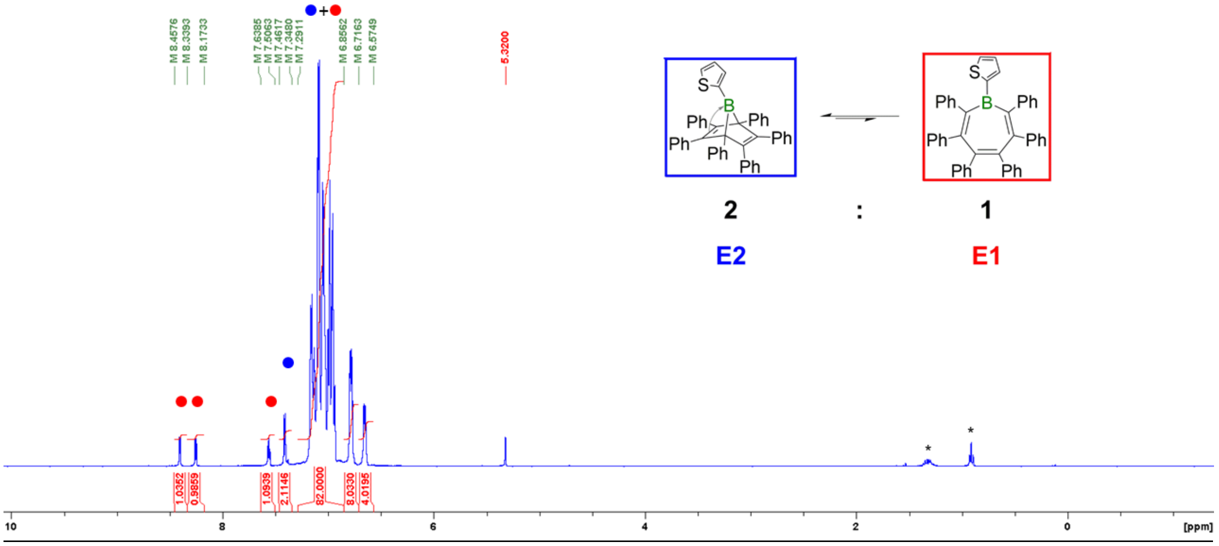

Figure S1. ^{1}H NMR spectrum in CD_2Cl_2 of the isomers **2b**/**3b** in chemical equilibrium at room temperature. **2b** is labeled as **E2** and **3b** as **E1** in the figure.

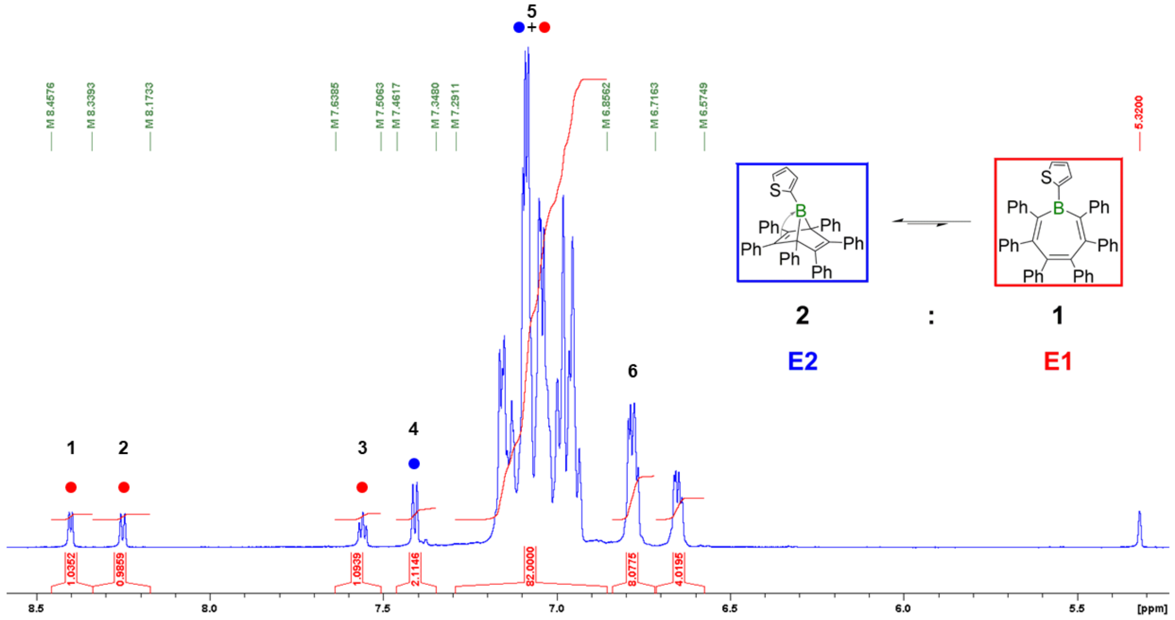

Figure S2. Expansion of the aromatic region of the ^{1}H NMR spectrum in CD_2Cl_2 of the isomers **2b**/**3b** in chemical equilibrium at room temperature. **2b** is labeled as **E2** and **3b** as **E1** in the figure.

Comment: The signals **1**–**3** can be assigned to the thienyl unit of compound **E1**, the signal **4** to the thienyl unit in **E2**. The set of resonances marked with the number **5** contains the second resonance for the thienyl unit of **E2**. The third signal of the thienyl unit of **E2** is part of the resonance marked with the number **6**. Moreover, all thienyl resonances can be detected by $^{1}H,^{1}H$ COSY NMR spectroscopy.

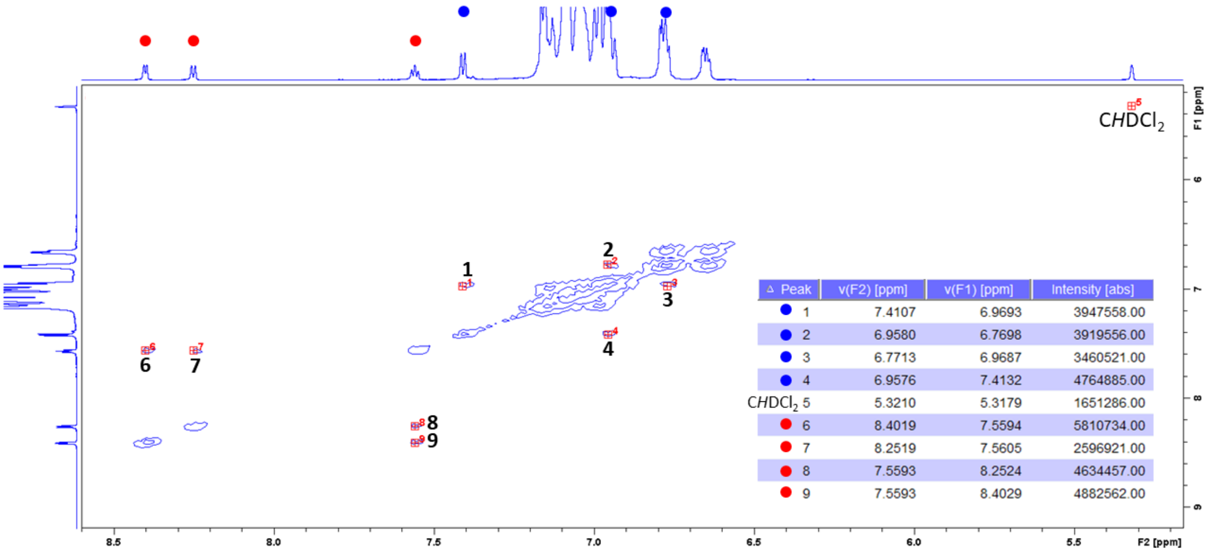

Figure S3. Excerpt of the aromatic region of the $^{1}H,^{1}H$ COSY NMR spectrum in CD_2Cl_2 of the isomers **2b**/**3b** at room temperature. The blue peaks (**E2**) correspond to **2b**, the red (**E1**) to borepin **3b**.

Comment: The signals with the numbers **1**–**4** can be assigned to the spin system of the thienyl unit of compound **E2** (boranorbornadiene). The signals with the numbers **6**–**9** can be assigned to the spin system of the thienyl unit in isomer **E1** (borepin).

B.2. ^{1}H NMR data of isomers 2c/3c

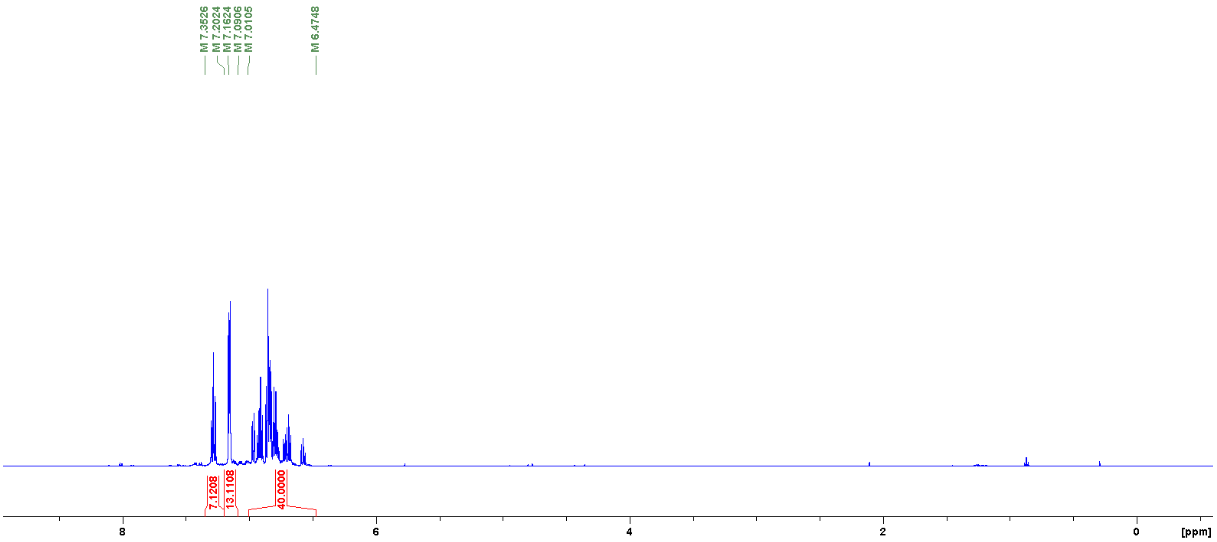

Figure S4. ^{1}H NMR spectrum of the mixture of isomers **2c** and **3c** (C_6D_6, 298 K).

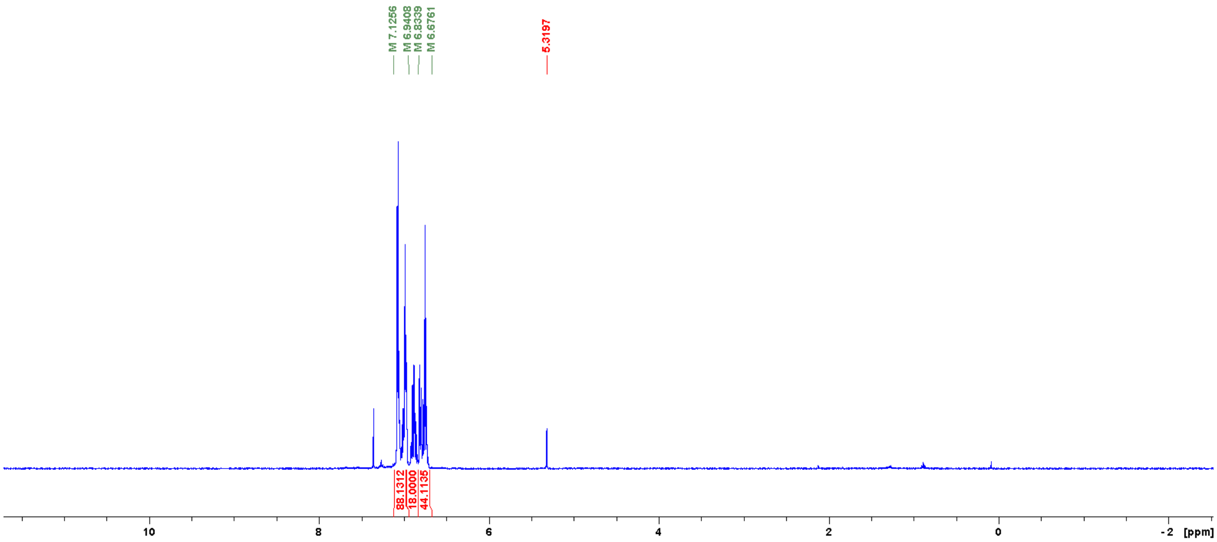

Figure S5. ^{1}H NMR spectrum of the mixture of isomers **2c** and **3c** (CD_2Cl_2, 298 K).

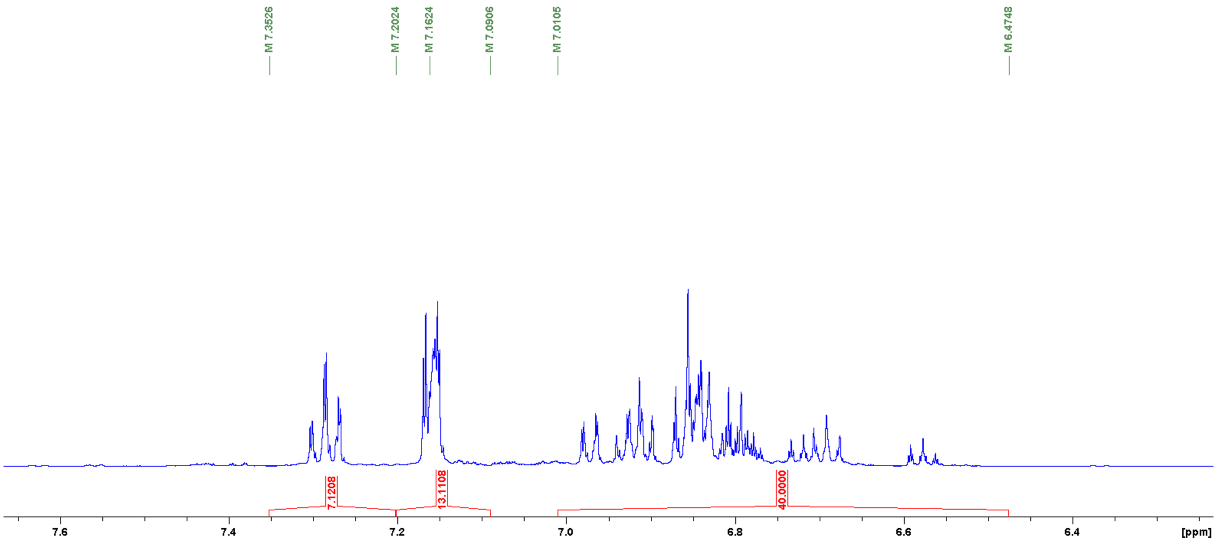

Figure S6. Expansion of the aromatic region of the ^{1}H NMR spectrum (C_6D_6, 298 K) of the mixture of isomers **2c** and **3c**.

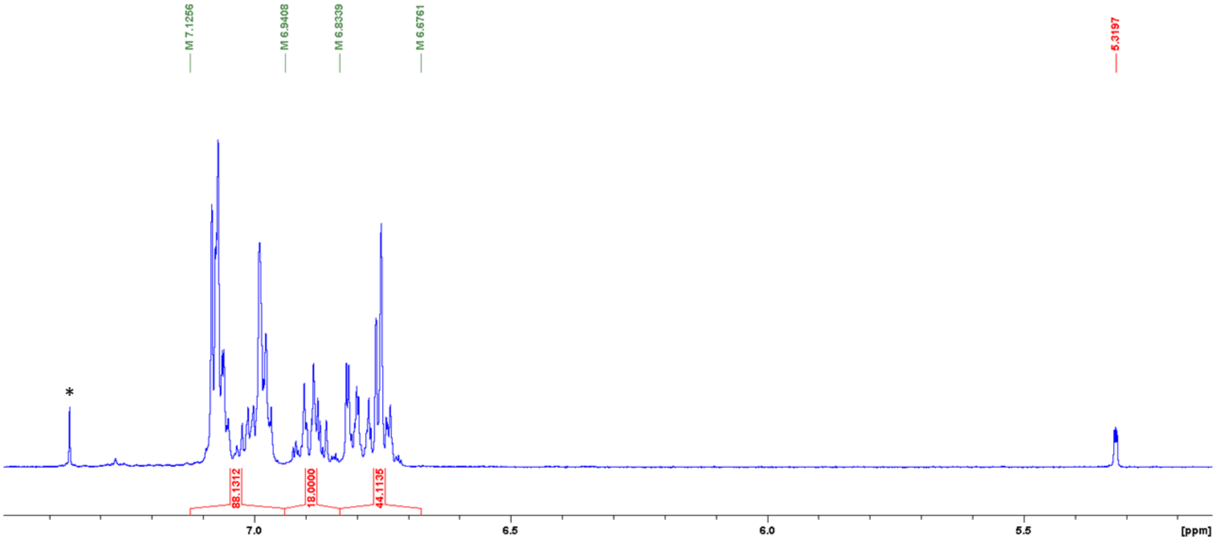

Figure S7. Expansion of the aromatic region of the ^{1}H NMR spectrum (CD_2Cl_2, 298 K) of the mixture of isomers **2c** and **3c**. The signal marked with an asterisk (*) is due to residual benzene.

B.3. ^{1}H NMR data of 3b(*I*Me)

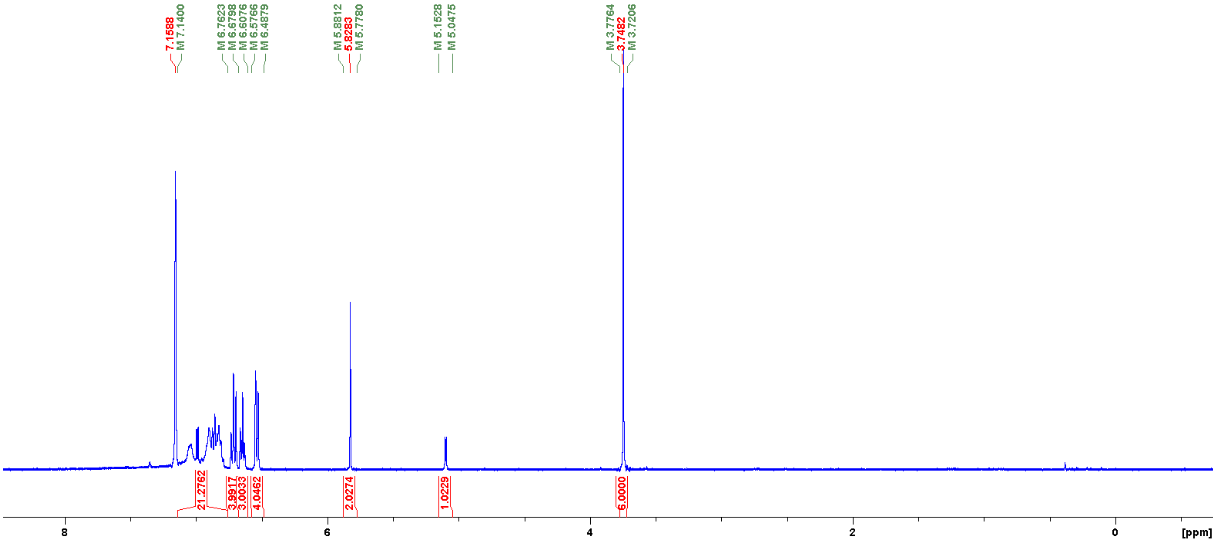

Figure S8. ^{1}H NMR spectrum of **3b(*I*Me)** in C_6D_6.

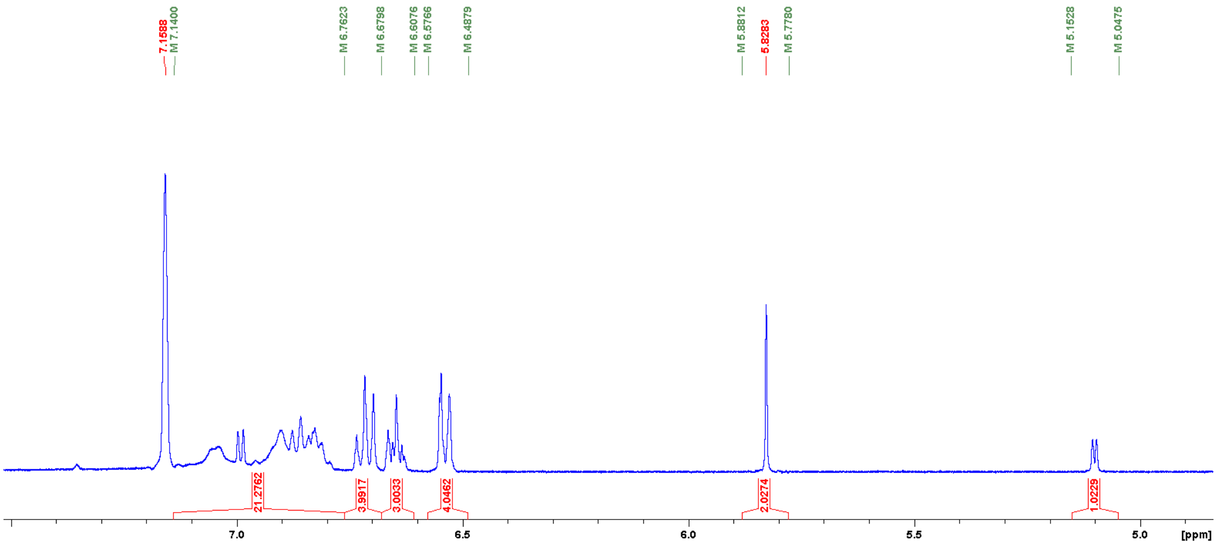

Figure S9. Expansion of the aromatic region of the ^{1}H NMR spectrum of **3b(*I*Me)** in C_6D_6.

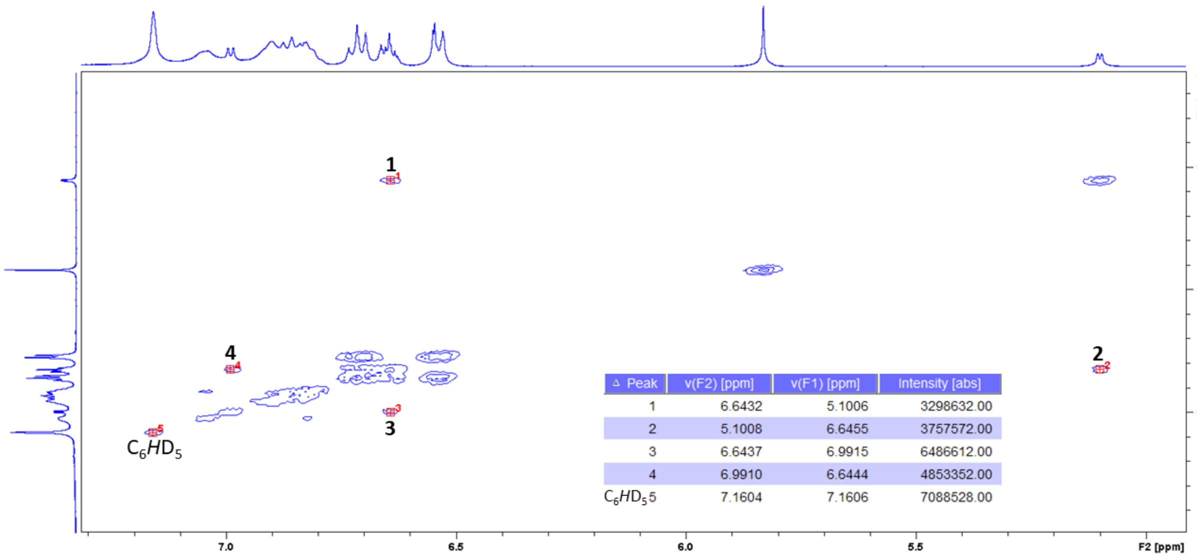

Figure S10. Excerpt of the aromatic region of the ^{1}H,^{1}H COSY NMR spectrum of **3b(*I*Me)** in C_6D_6.

Comment: The signals with the numbers **1–4** can be assigned to the thienyl spin system.

B.4. ^{1}H NMR data of 3c(*I*Me)

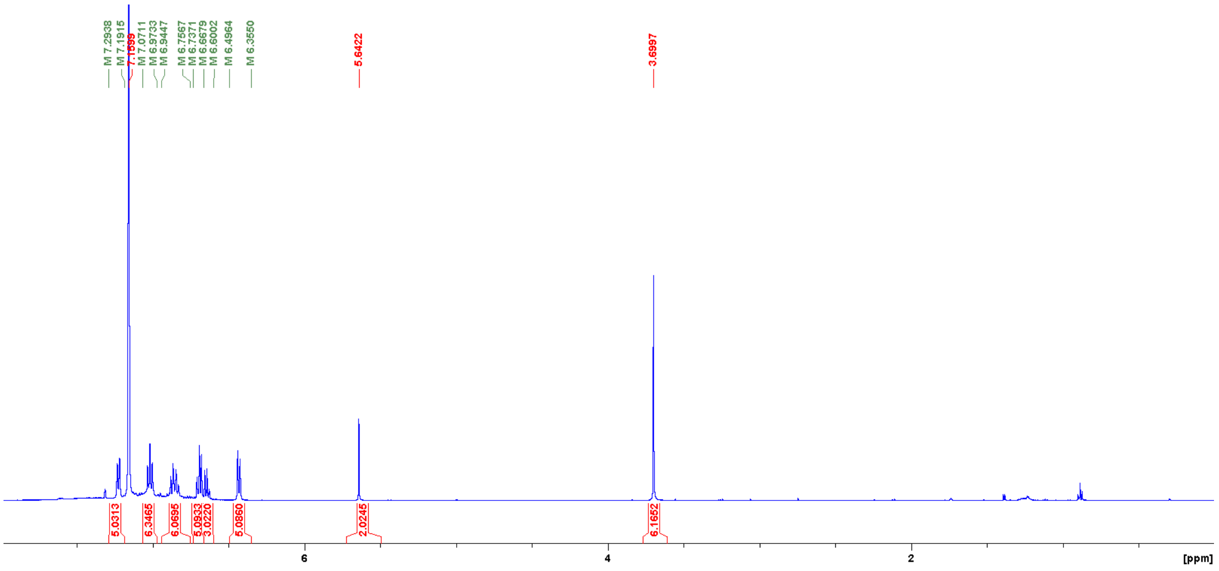

Figure S11. ^{1}H NMR spectrum of **3c(*I*Me)** in C_6D_6.

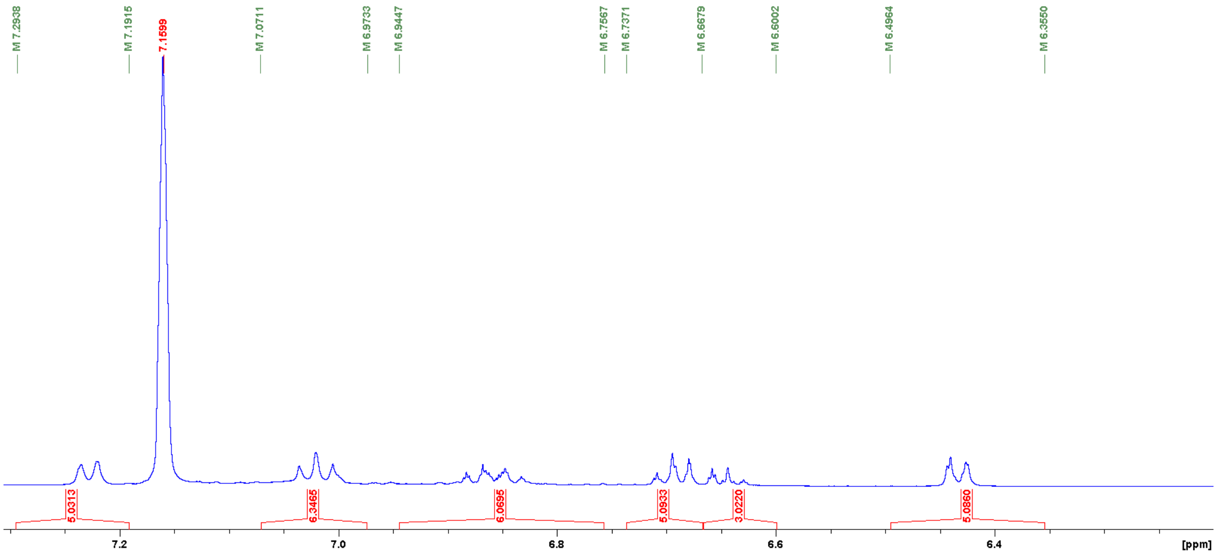

Figure S12. Expansion of the aromatic region of the ^{1}H NMR spectrum of **3c(*I*Me)** in C_6D_6.

B.5. ¹H NMR data of 3c(thf)

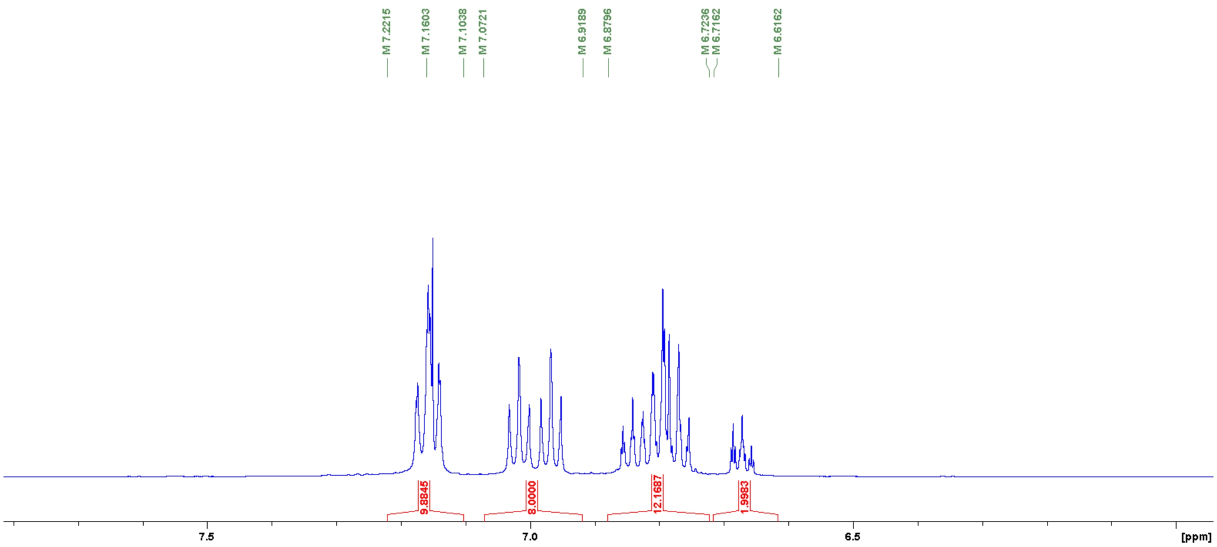

Figure S13. ^{1}H NMR spectrum of **3c(thf)** in C_6D_6.

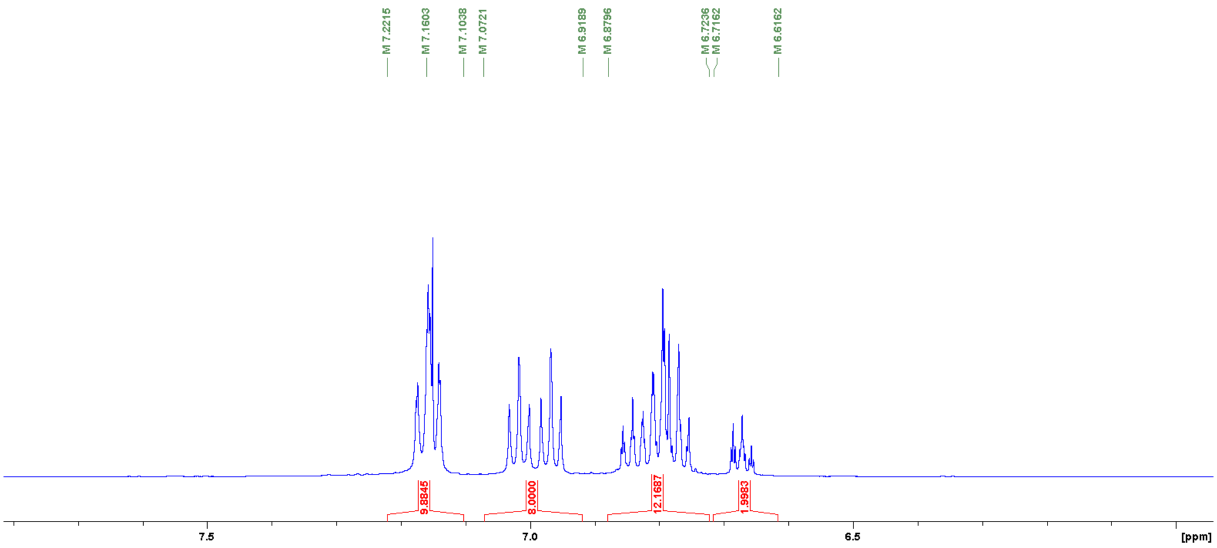

Figure S14. Expansion of the aromatic region of the ^{1}H NMR spectrum of **3c(thf)** in C_6D_6.

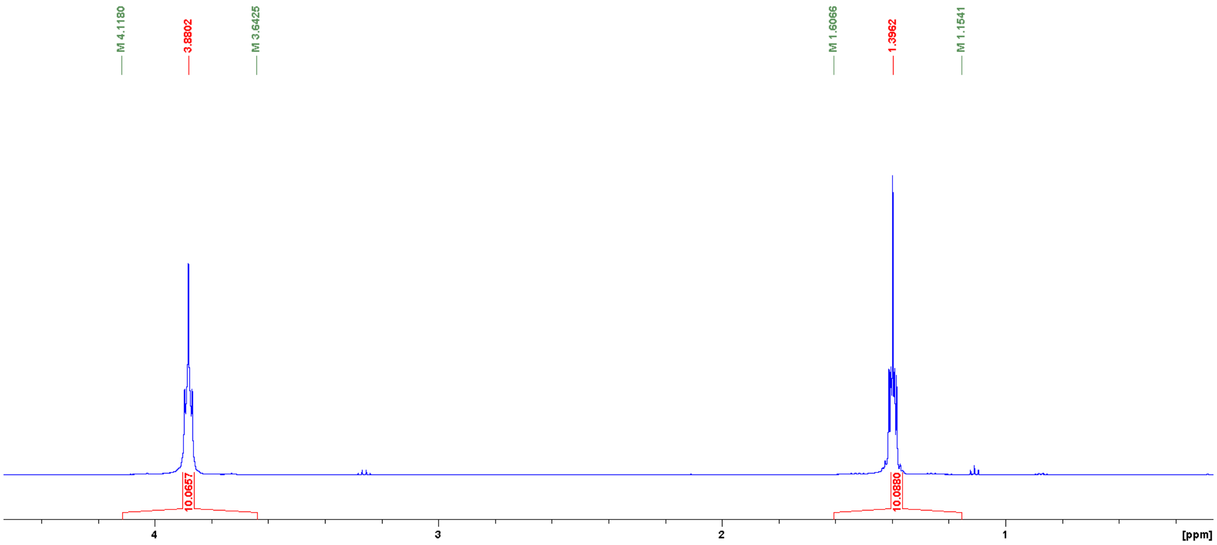

Figure S15. Expansion of the aliphatic region of the ¹H NMR spectrum of **3c(thf)** in C_6D_6.

B.6. ^{1}H NMR data of 4a

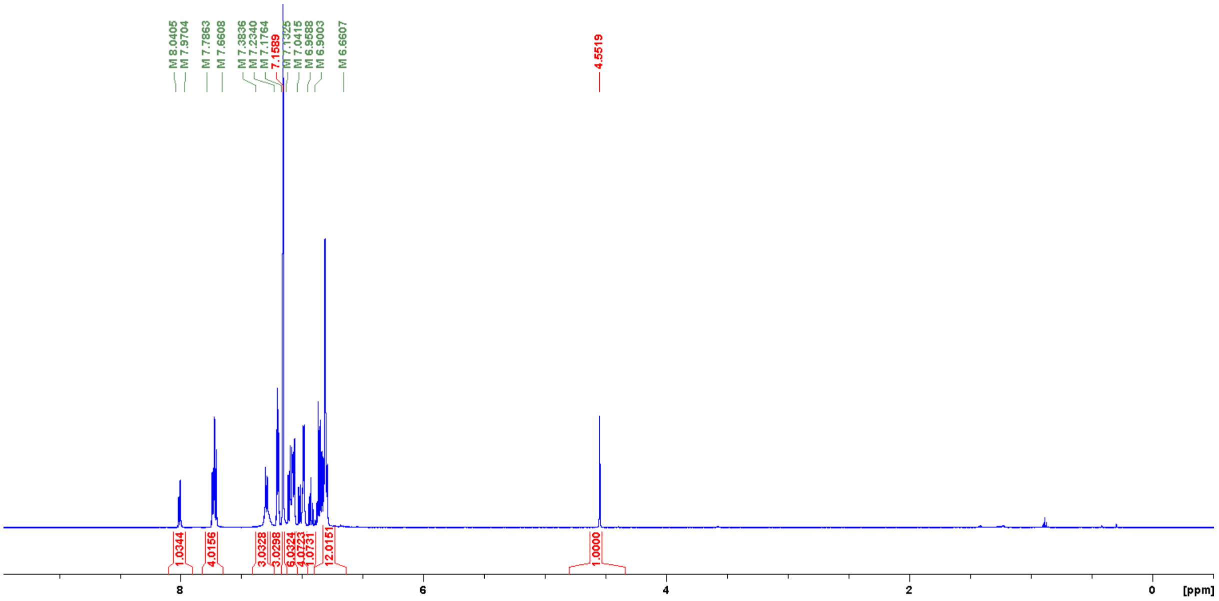

Figure S16. ^{1}H NMR spectrum of **4a** in C_6D_6.

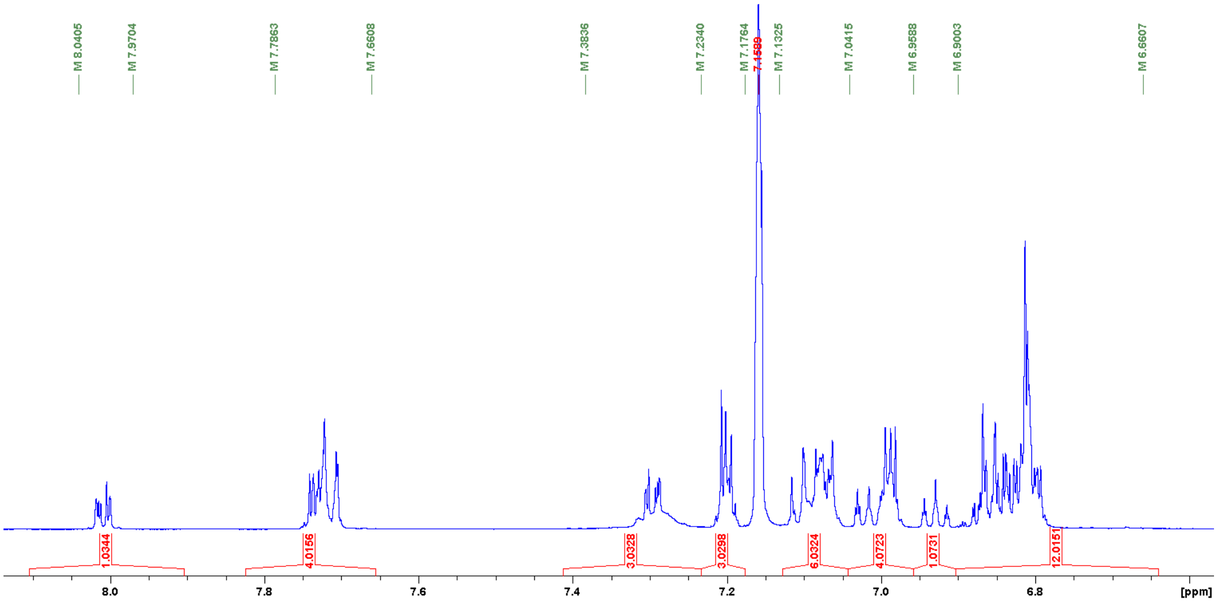

Figure S17. Expansion of the aromatic region of the ^{1}H NMR spectrum of **4a** in C_6D_6.

B.7. ¹H NMR data of 4b

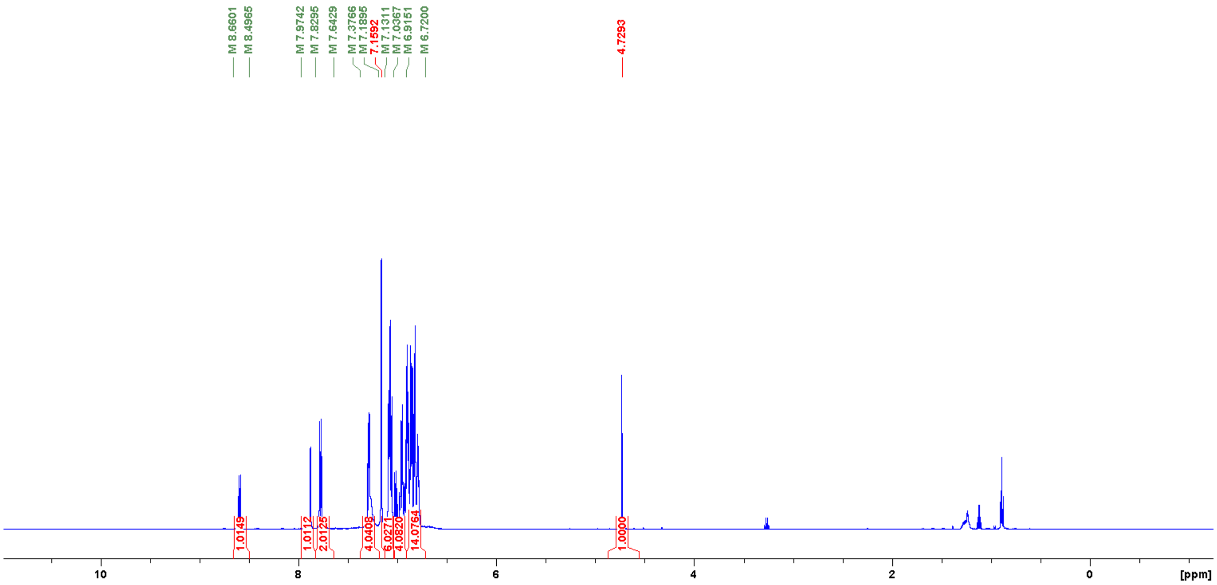

Figure S18. ^{1}H NMR spectrum of **4b** in C_6D_6.

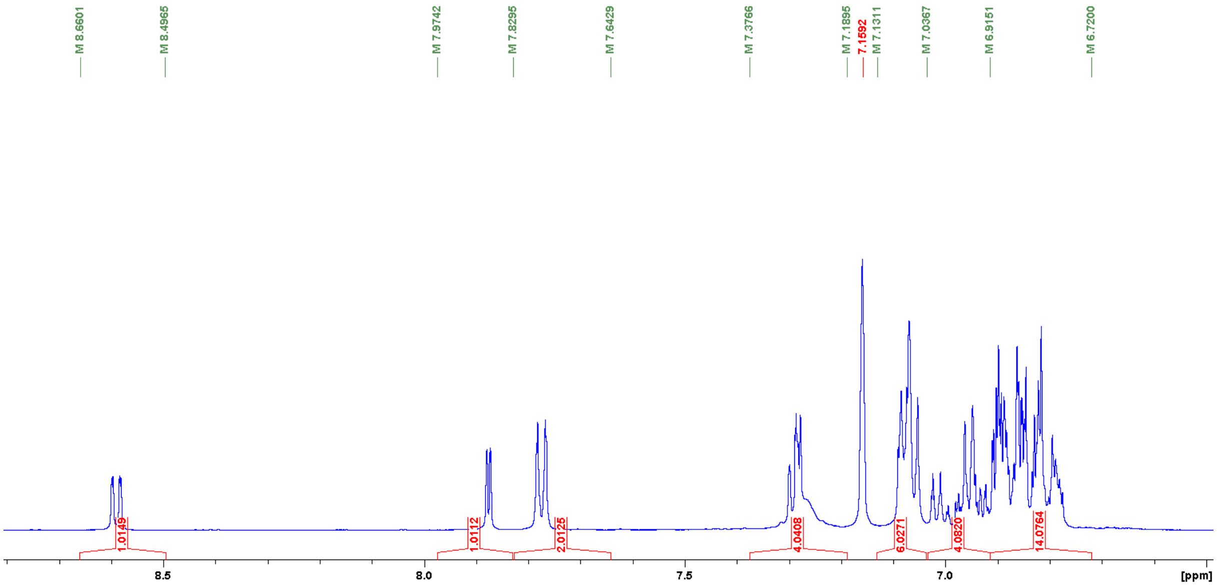

Figure S19. Expansion of the aromatic region of the ^{1}H NMR spectrum of **4b** in C_6D_6.

B.7. ^{1}H NMR data of 4c

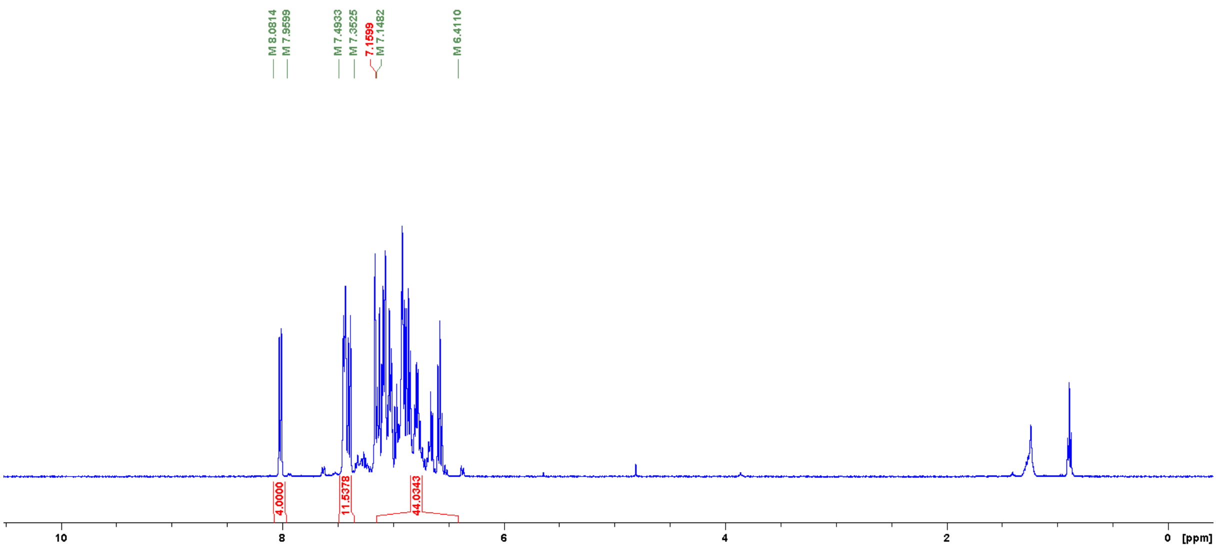

Figure S20. ^{1}H NMR spectrum of **4c** in C_6D_6.

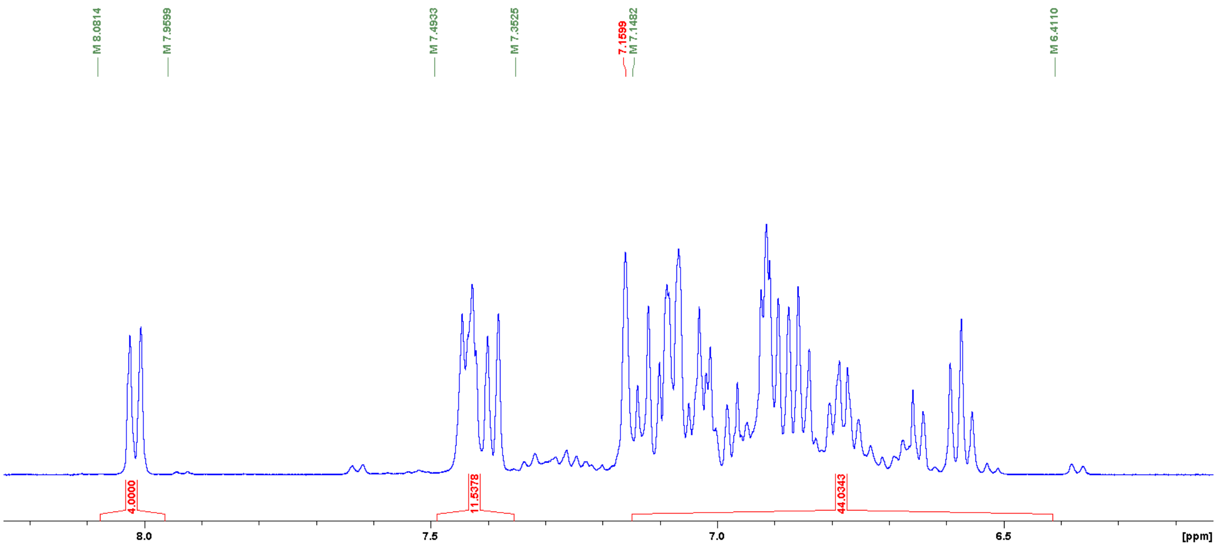

Figure S21. Expansion of the aromatic region of the ^{1}H NMR spectrum of **4c** in C_6D_6.

B.8. ^{13}C NMR data of 2b/3b

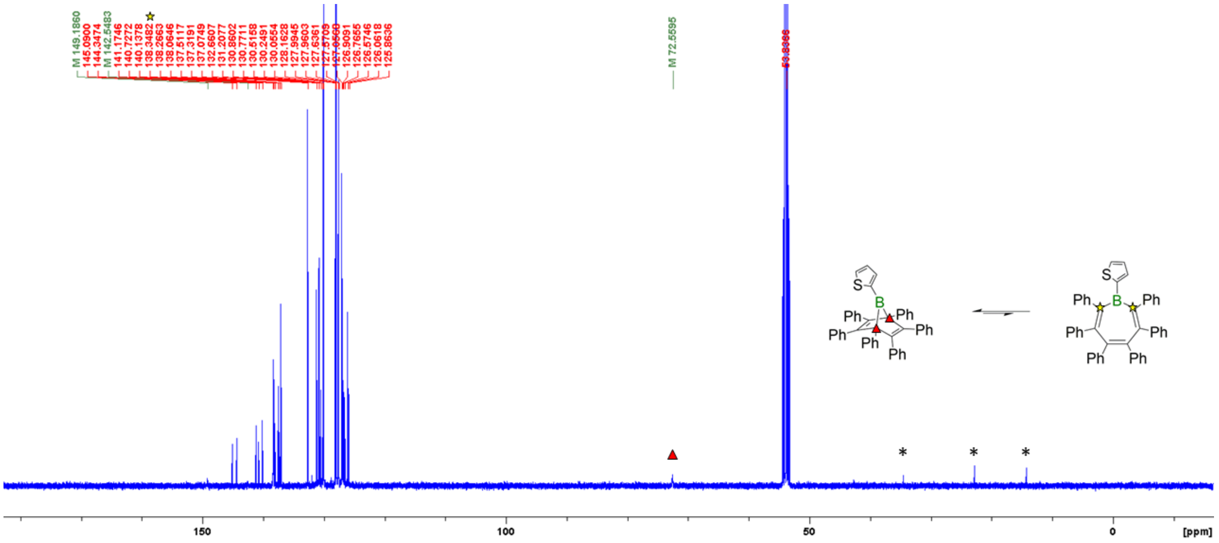

Figure S22. $^{13}C\{^{1}H\}$ NMR spectrum of **2b/3b** in CD_2Cl_2. The signal marked with a triangle is associated with the quaternary boron-bound carbon atom of **2b**. The signals marked with an asterisk (*) can be assigned to residual pentane.

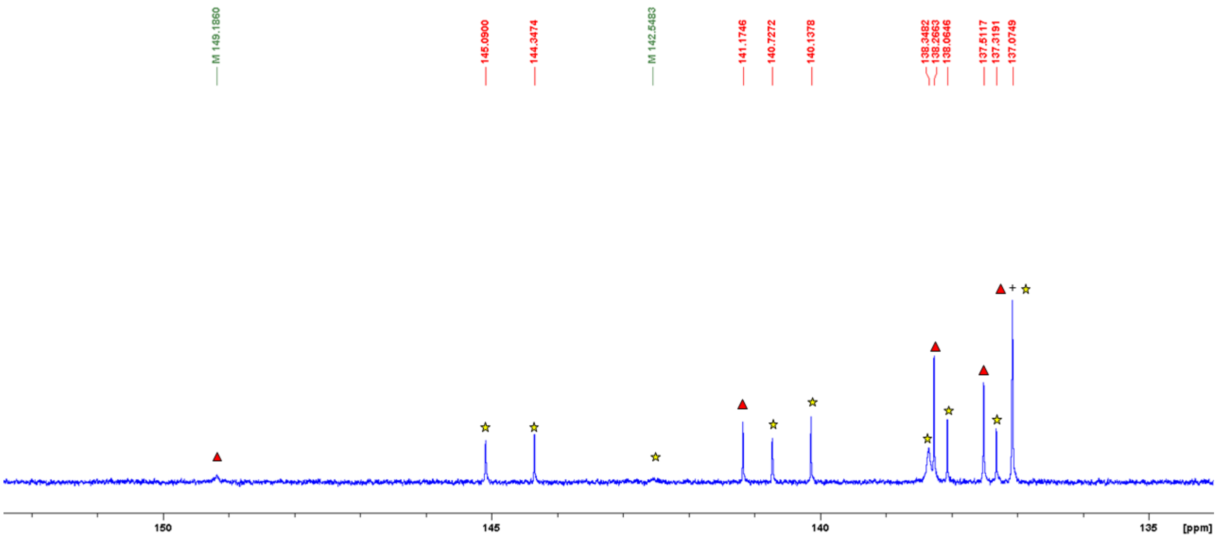

Figure S23. Expansion of the aromatic region (part 1 of 2) of the $^{13}C\{^{1}H\}$ NMR spectrum of **2b/3b** in CD_2Cl_2. The signals marked with a star can be assigned to borepin **3b**, whereas the signals marked with a triangle can be assigned to **2b**.

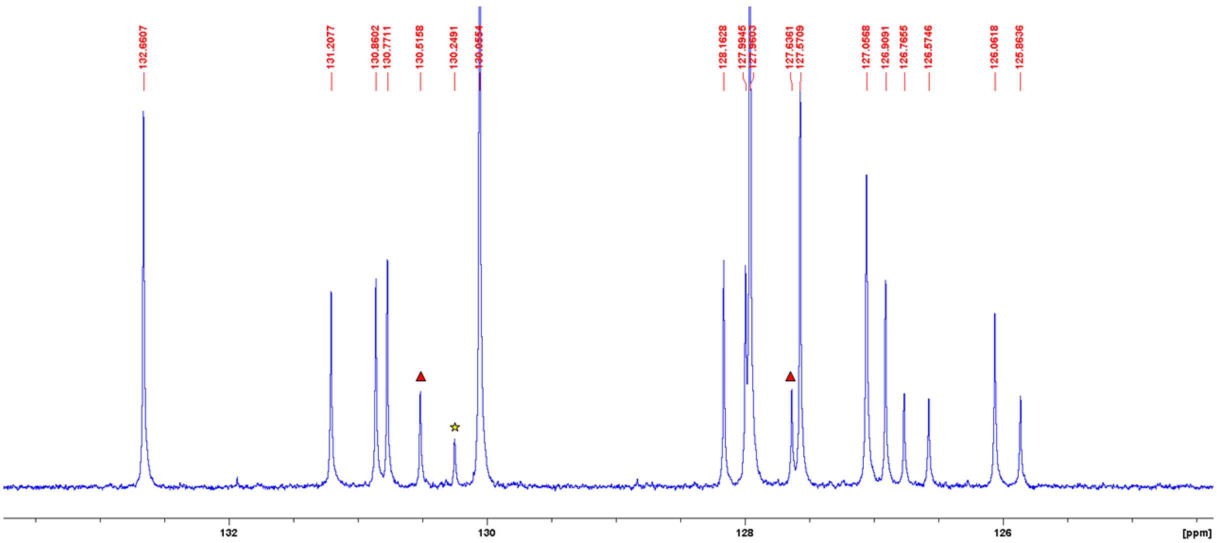

Figure S24. Expansion of the aromatic region (part 2 of 2) of the $^{13}C\{^{1}H\}$ NMR spectrum of **2b/3b** in CD_2Cl_2. The signal marked with a star can be assigned to borepin **3b**, whereas the signals marked with a triangle can be assigned to **2b**.

B.9. ^{13}C NMR data of 2c/3c

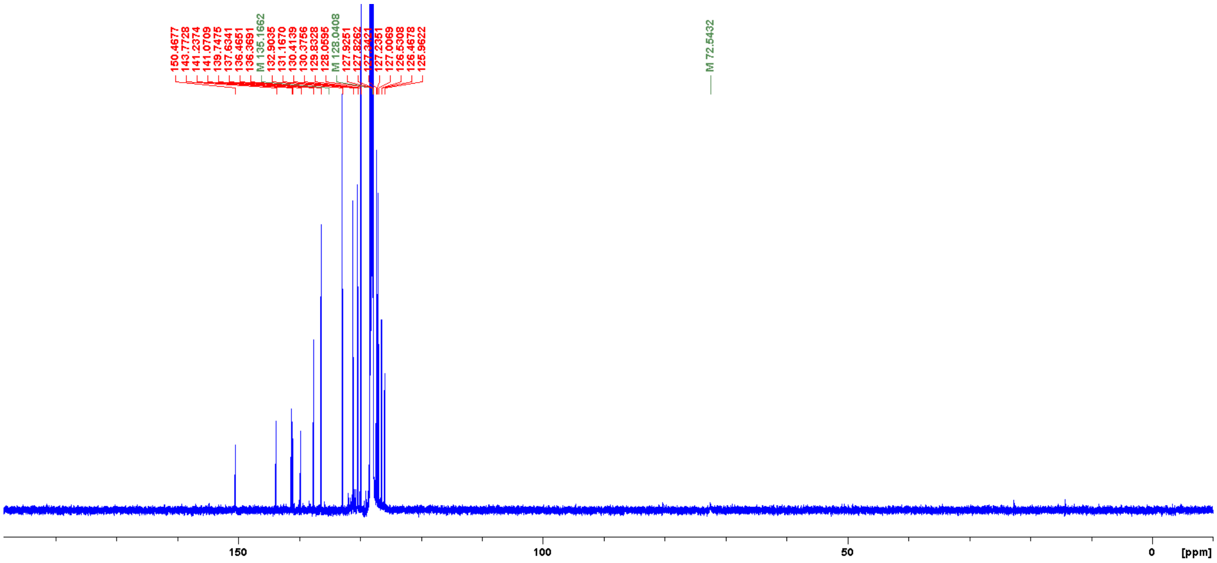

Figure S25. $^{13}C\{^1H\}$ NMR spectrum of the isomers **2c/3c** in C_6D_6 at room temperature.

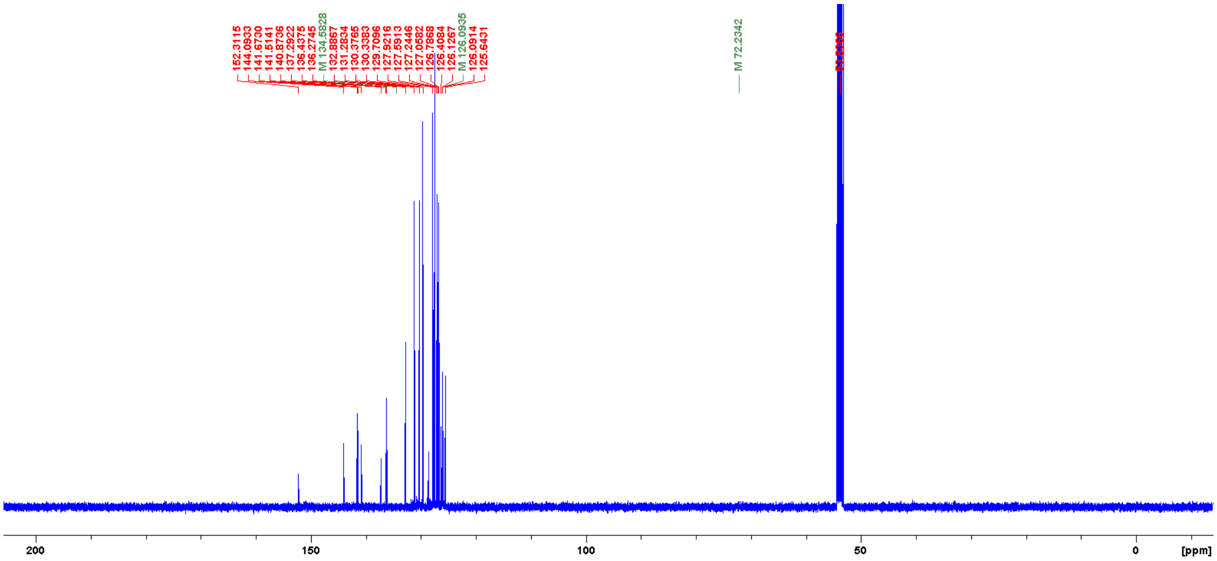

Figure S26. $^{13}C\{^1H\}$ NMR spectrum of the isomers **2c/3c** in CD_2Cl_2 at room temperature.

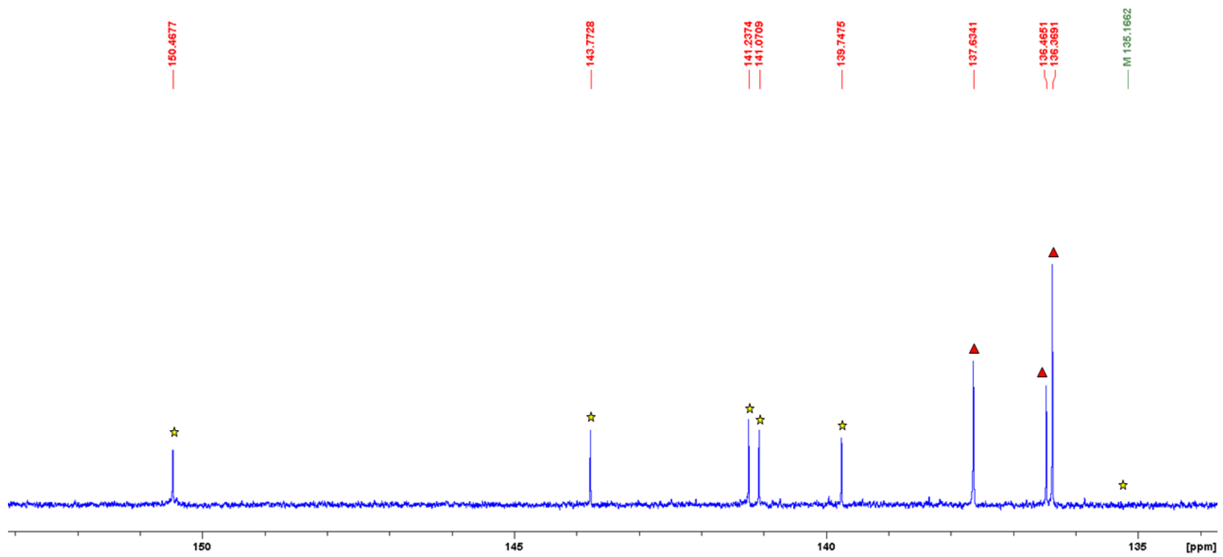

Figure S27. Expansion of the aromatic region (part 1 of 2) of the $^{13}C\{^{1}H\}$ NMR spectrum of **2c/3c** in C_6D_6. The signals with a star can be assigned to **3c**, whereas the signals with a triangle can be assigned to **2c**.

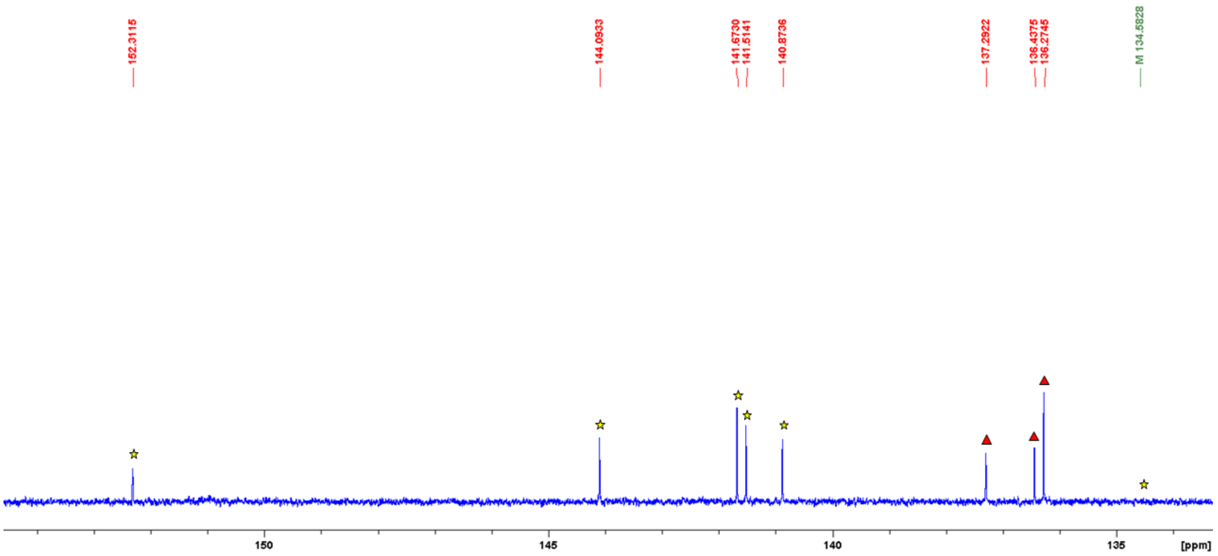

Figure S28. Expansion of the aromatic region (part 1 of 2) of the $^{13}C\{^{1}H\}$ NMR spectrum of **2c/3c** in CD_2Cl_2. The signals with a star can be assigned to **3c**, whereas the signals with a triangle can be assigned to **2c**.

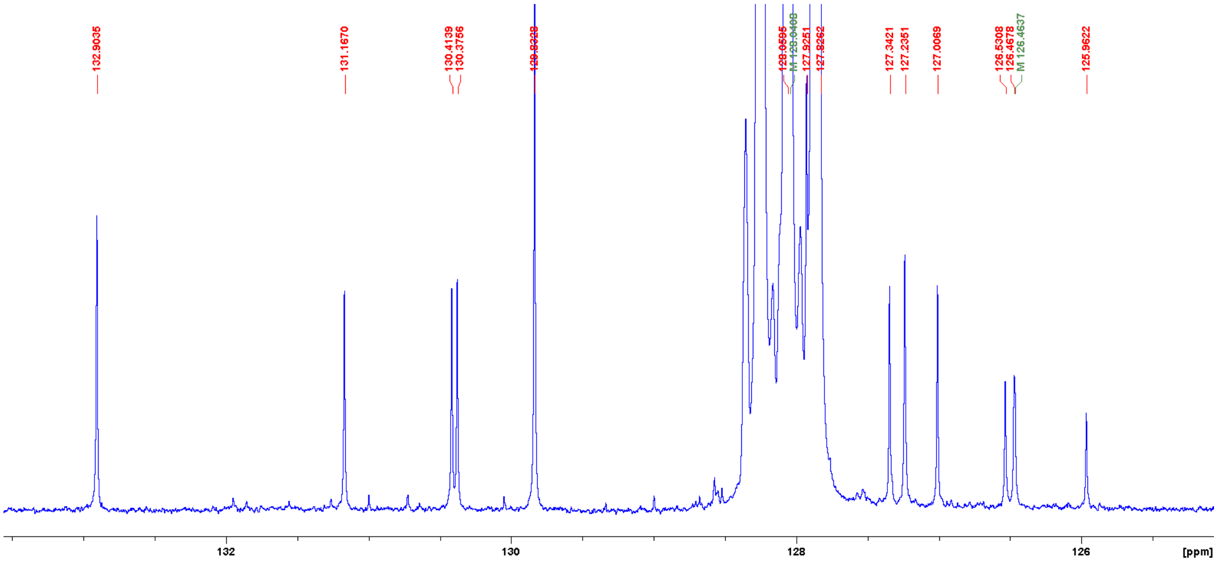

Figure S29. Expansion of the aromatic region (part 2 of 2) of the $^{13}C\{^{1}H\}$ NMR spectrum of **2c/3c** in C_6D_6.

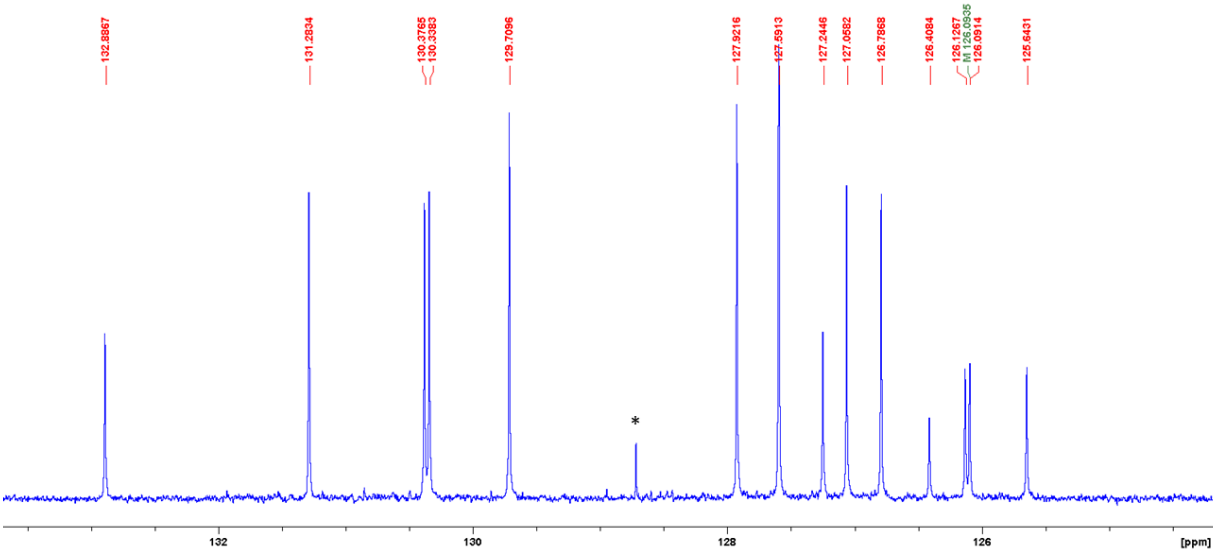

Figure S30. Expansion of the aromatic region (part 2 of 2) of the $^{13}C\{^{1}H\}$ NMR spectrum of **2c/3c** in CD_2Cl_2. The signal marked with an asterisk (*) can be assigned to residual benzene.

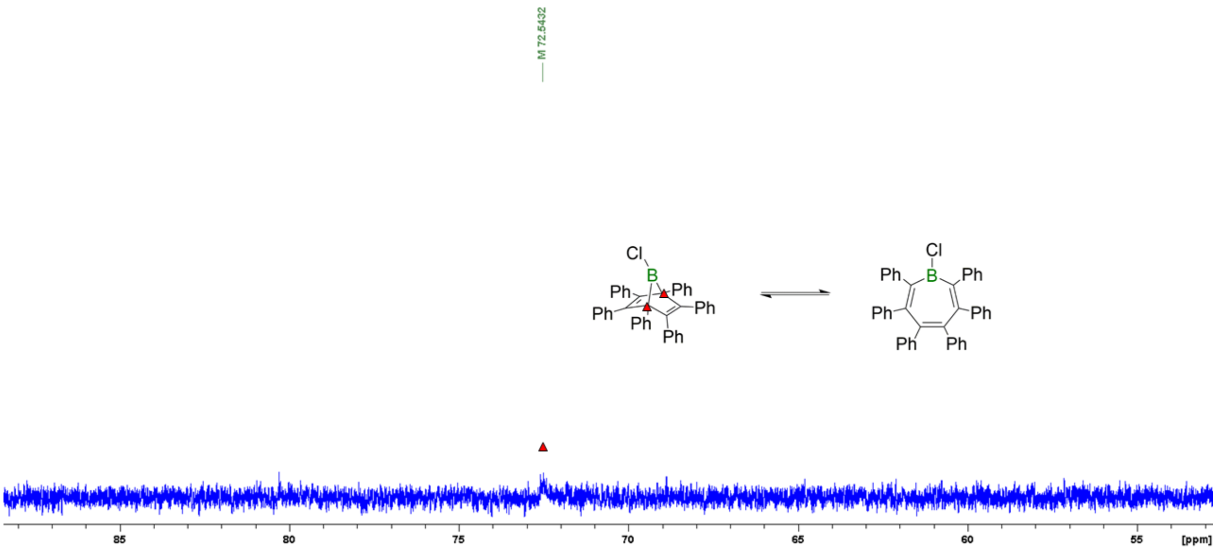

Figure S31. Expansion of the aliphatic region of the $^{13}C\{^{1}H\}$ NMR spectrum of **2c/3c** in C_6D_6. The signal with a triangle is associated with the quaternary boron-bound carbon atom of compound **2c**.

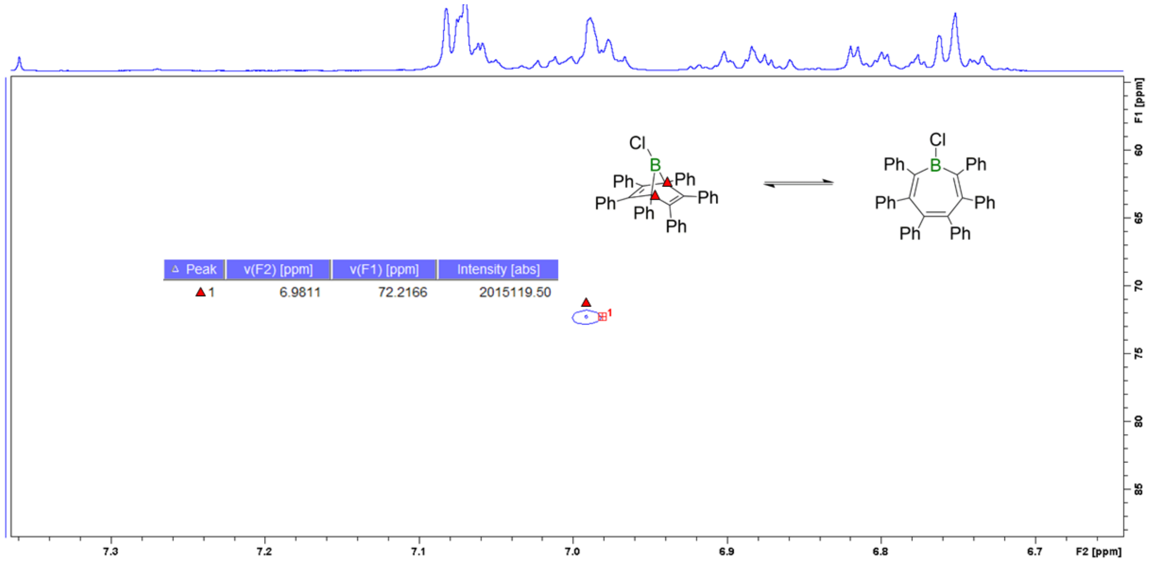

Figure S32. Excerpt of the aliphatic region of the $^{13}C,^{1}H$ HMBC NMR spectrum of **2c/3c** in CD_2Cl_2. The signal marked with a triangle is associated with the quaternary boron-bound carbon atom of **2c**.

B.10. ^{13}C NMR data of 3b(*I*Me)

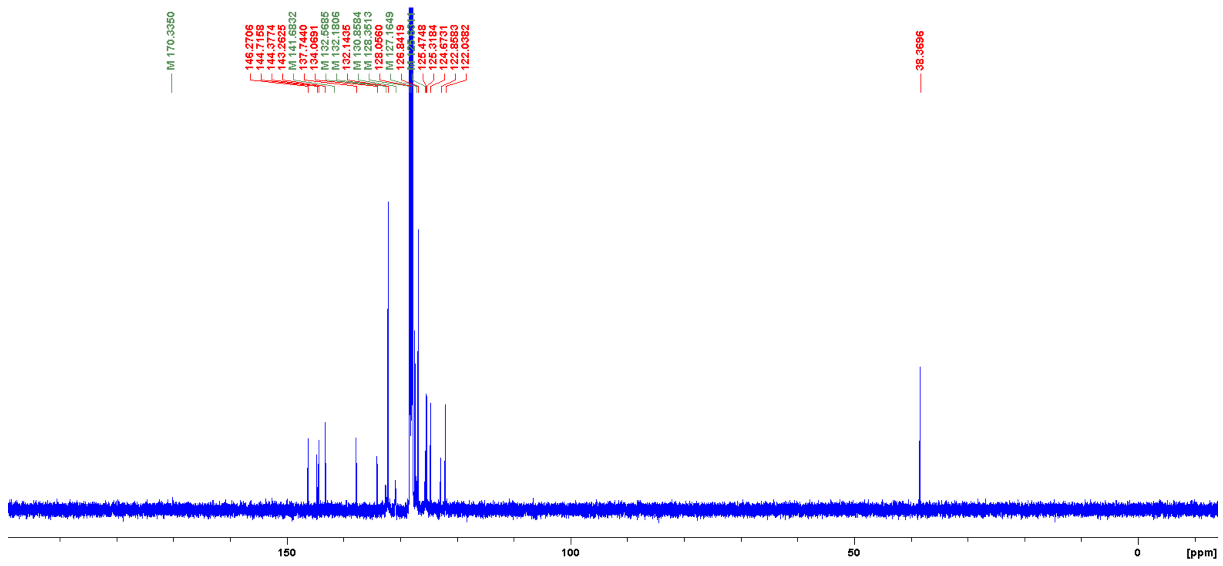

Figure S33. $^{13}C\{^1H\}$ NMR spectrum of **3b(*I*Me)** in C_6D_6.

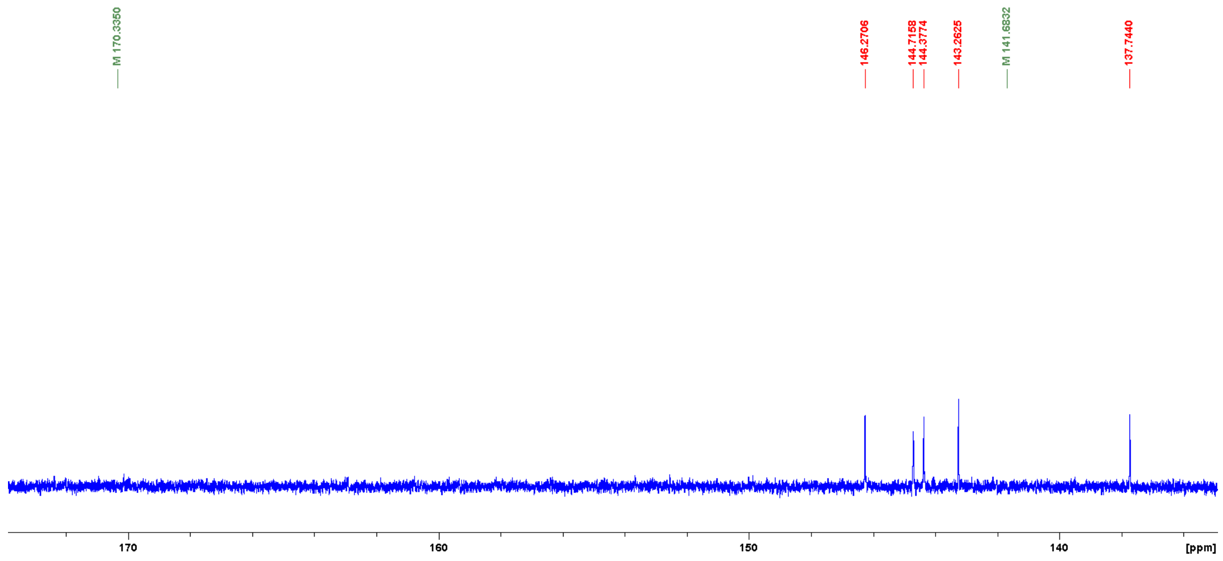

Figure S34. Expansion of the aromatic region (part 1of 2) of the $^{13}C\{^1H\}$ NMR spectrum of **3b(*I*Me)** in C_6D_6.

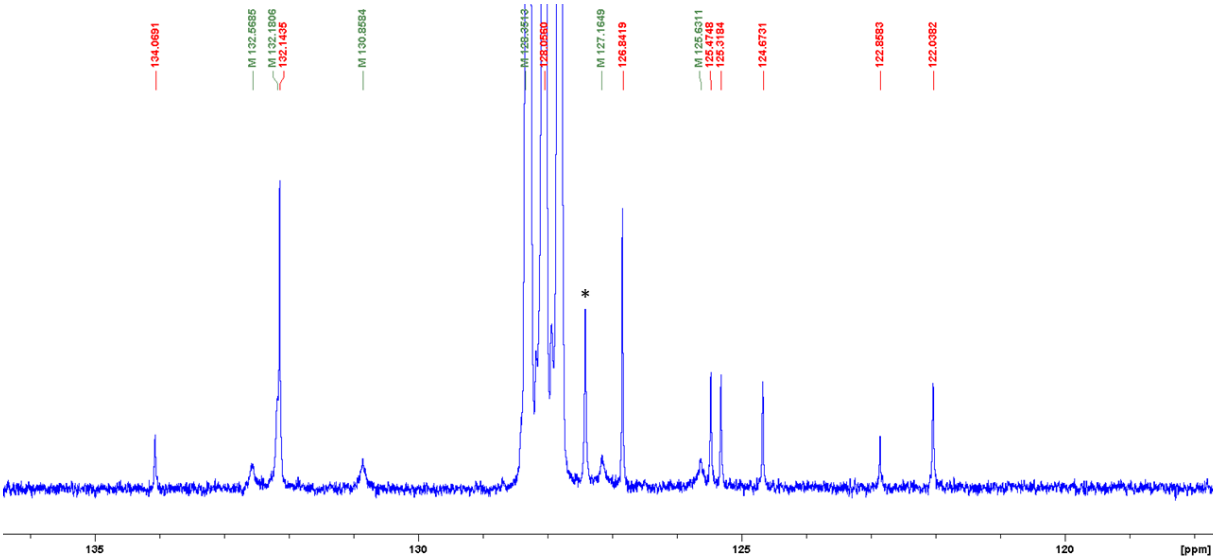

Figure S35. Expansion of the aromatic region (part 2 of 2) of the $^{13}C\{^{1}H\}$ NMR spectrum of **3b(*I*Me)** in C_6D_6. The signal marked with an asterisk (*) does not belong to **3b(*I*Me)**: the $^{13}C,^{1}H$ HSQC as well as the $^{13}C,^{1}H$ HMBC NMR experiment do not show correlations to the identified ^{1}H and ^{13}C NMR signals of **3b(*I*Me)**.

B.11. ^{13}C NMR data of 3c(*I*Me)

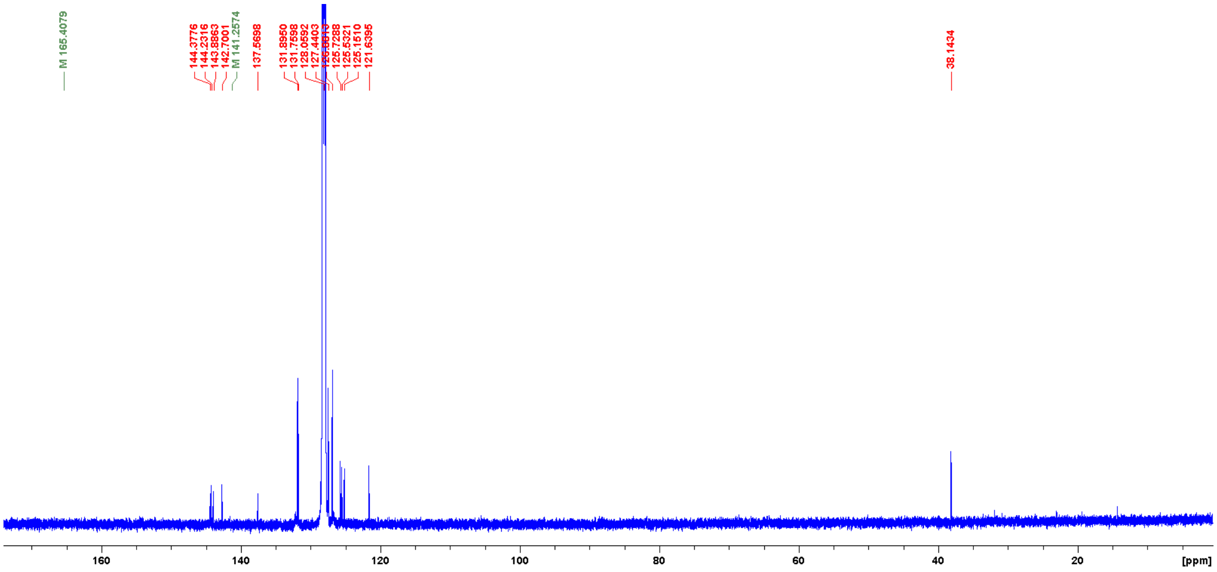

Figure S36. $^{13}C\{^{1}H\}$ NMR spectrum of **3c(*I*Me)** in C_6D_6.

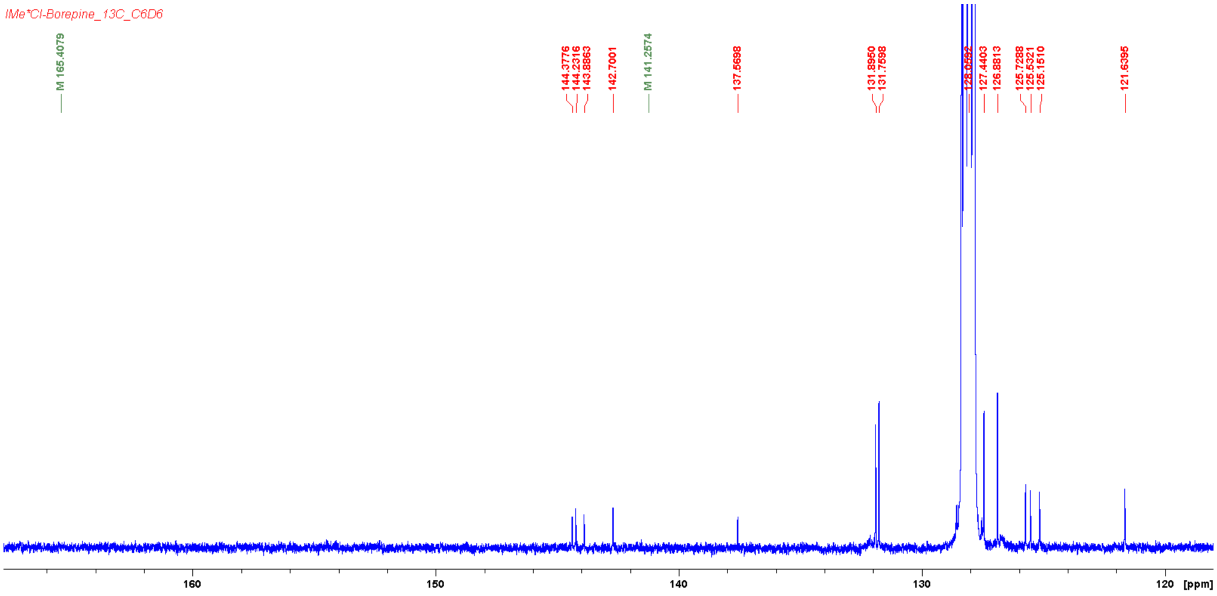

Figure S37. Expansion of the aromatic region of the $^{13}C\{^{1}H\}$ NMR spectrum of **3c(*I*Me)** in C_6D_6.

B.12. ^{13}C NMR data of 3c(thf)

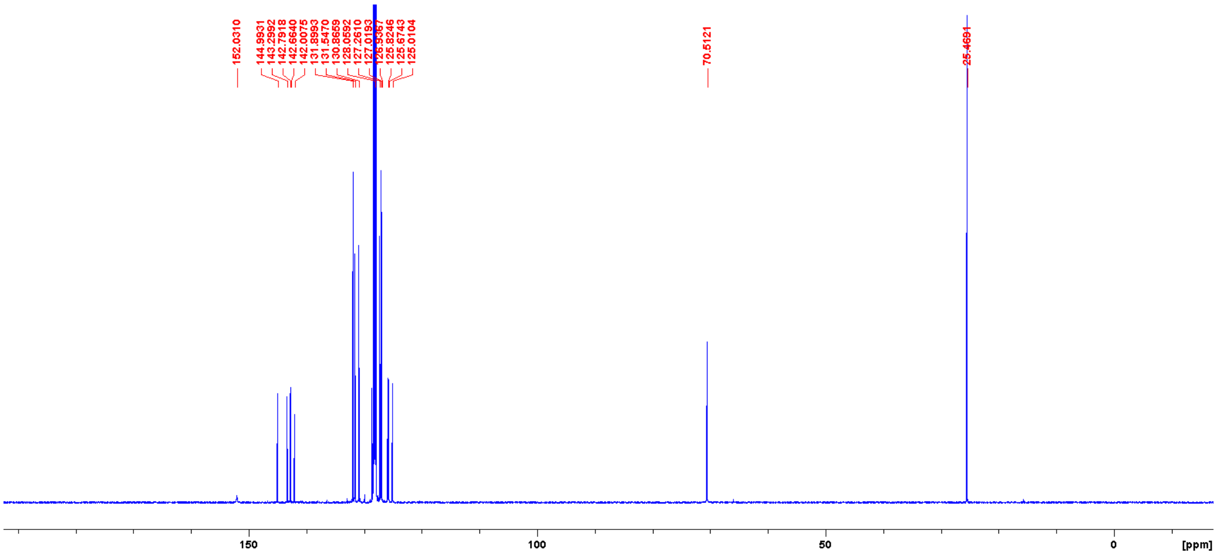

Figure S38. $^{13}C\{^1H\}$ NMR spectrum of **3c(thf)** in C_6D_6.

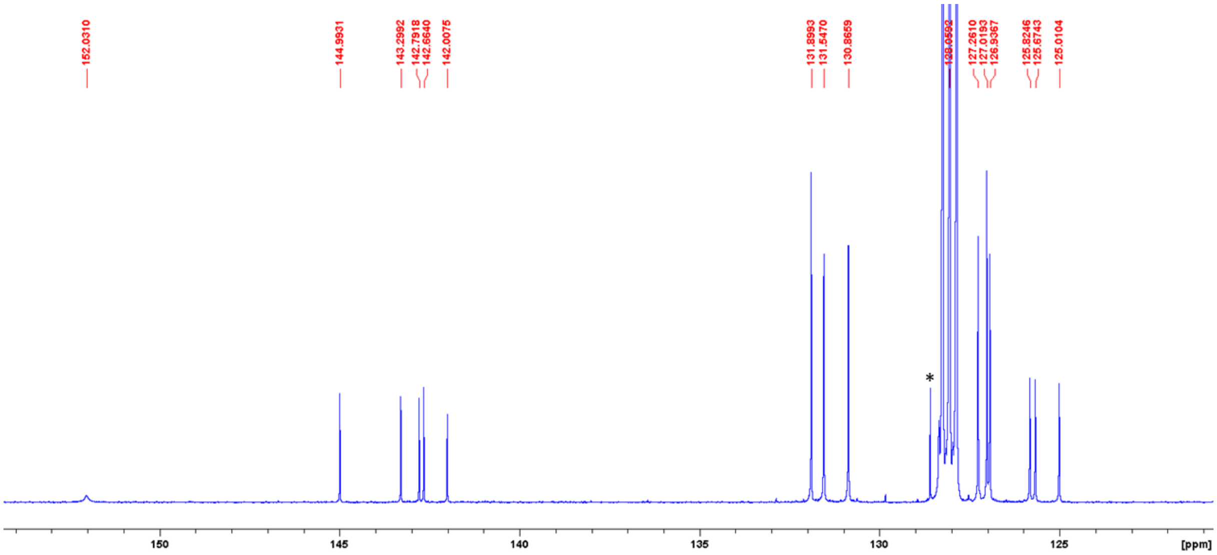

Figure S39. Expansion of the aromatic region of the $^{13}C\{^1H\}$ NMR spectrum of **3c(thf)** in C_6D_6. The signal marked with an asterisk (*) correspond to C_6H_6, which is generated via C/D exchange of C_4H_8O and C_6D_6.

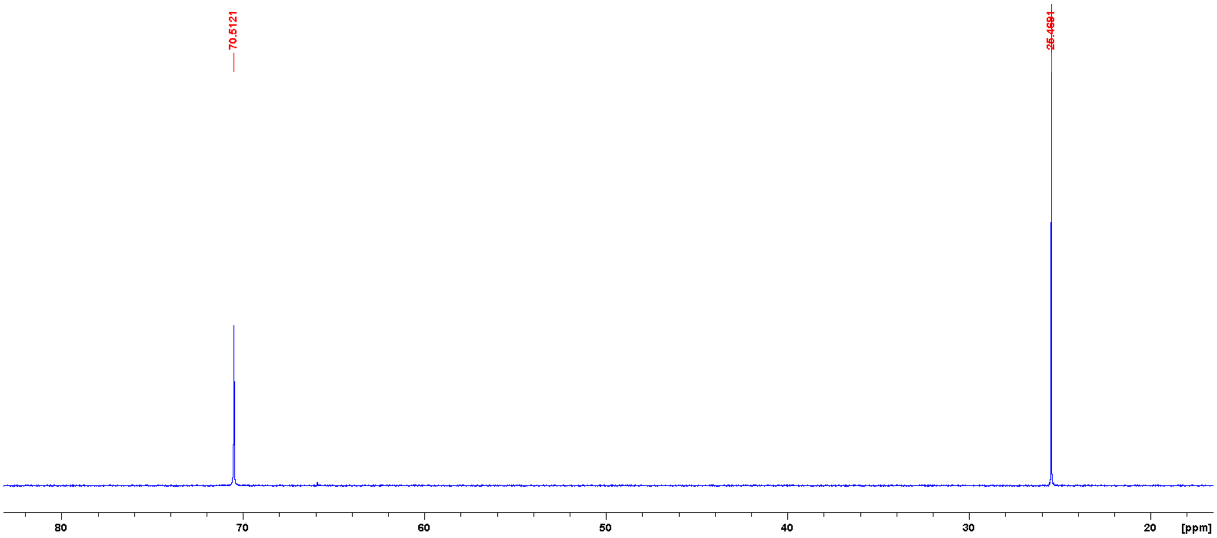

Figure S40. Expansion of the aliphatic region of the $^{13}C\{^{1}H\}$ NMR spectrum of **3c(thf)** in C_6D_6.

B.13. ^{13}C NMR data of 4a

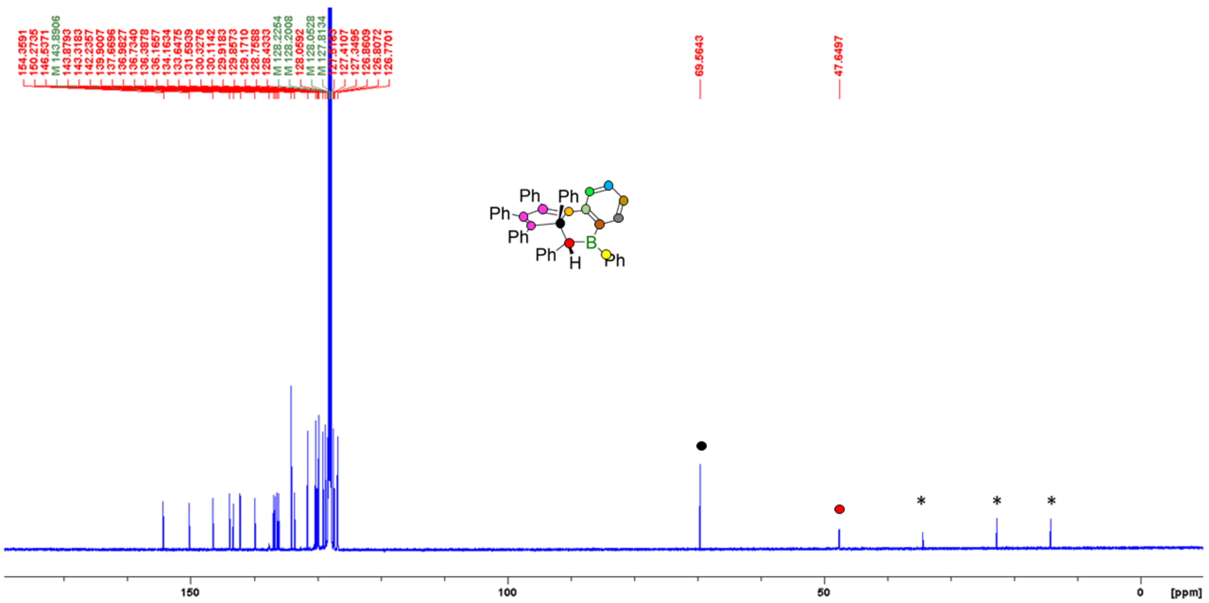

Figure S41. $^{13}C\{^1H\}$ NMR spectrum of **4a** in C_6D_6. The signal marked with an asterisk (*) can be assigned to residual pentane.

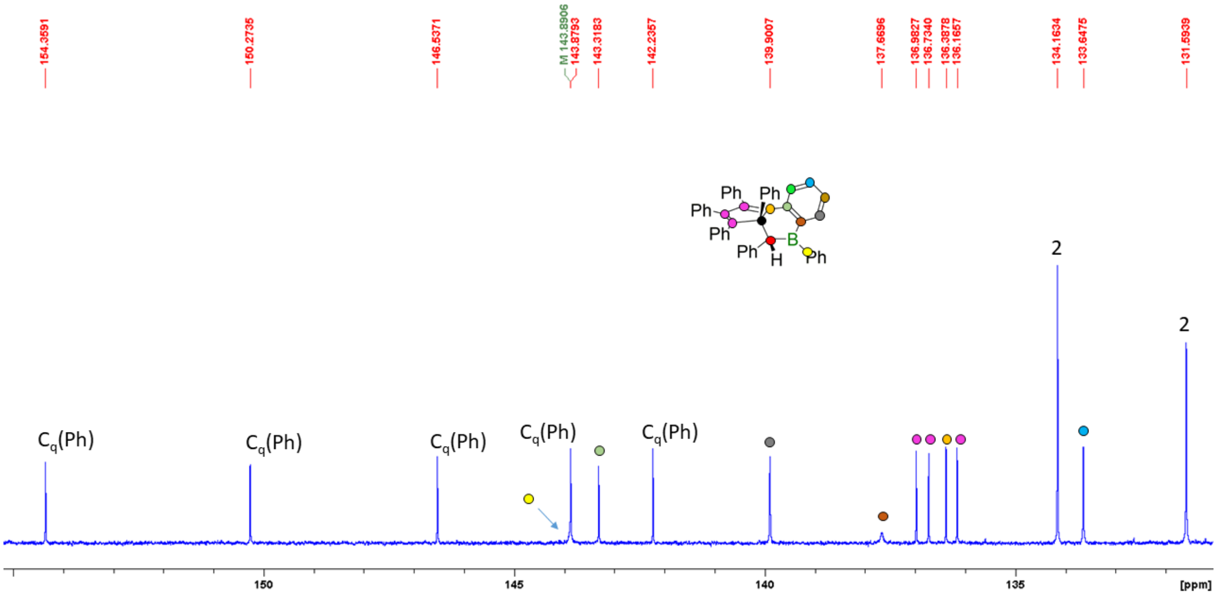

Figure S42. Expansion of the aromatic region (part 1 of 2) of the $^{13}C\{^1H\}$ NMR spectrum of **4a** in C_6D_6.

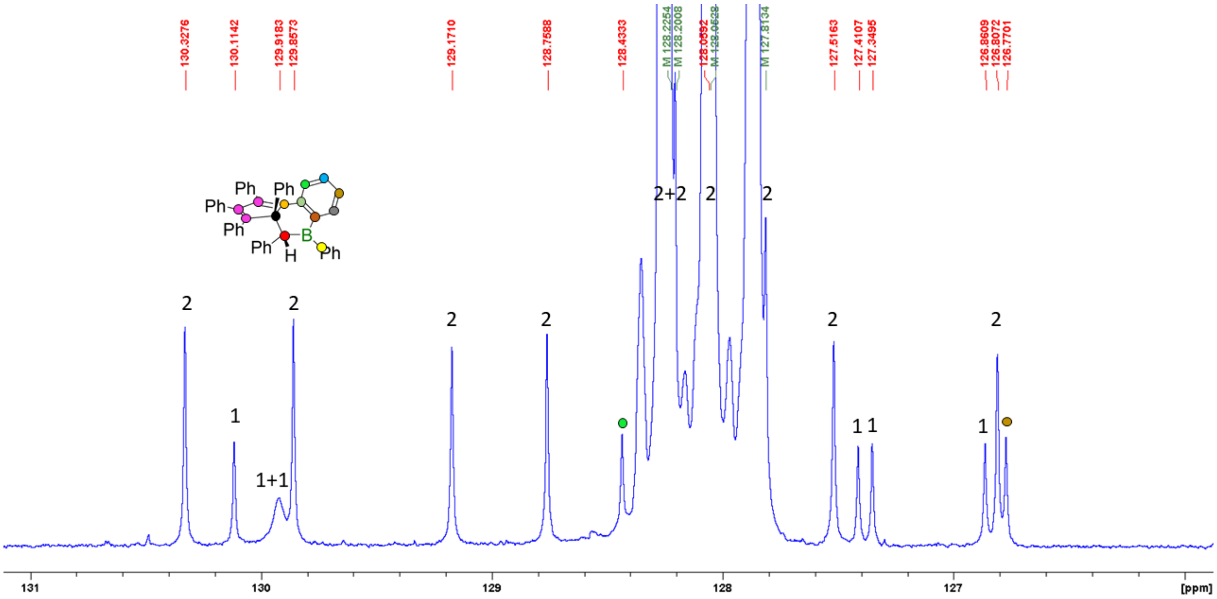

Figure S43. Expansion of the aromatic region (part 2 of 2) of the $^{13}C\{^{1}H\}$ NMR spectrum of **4a** in C_6D_6.

B.14. ^{13}C NMR data of 4b

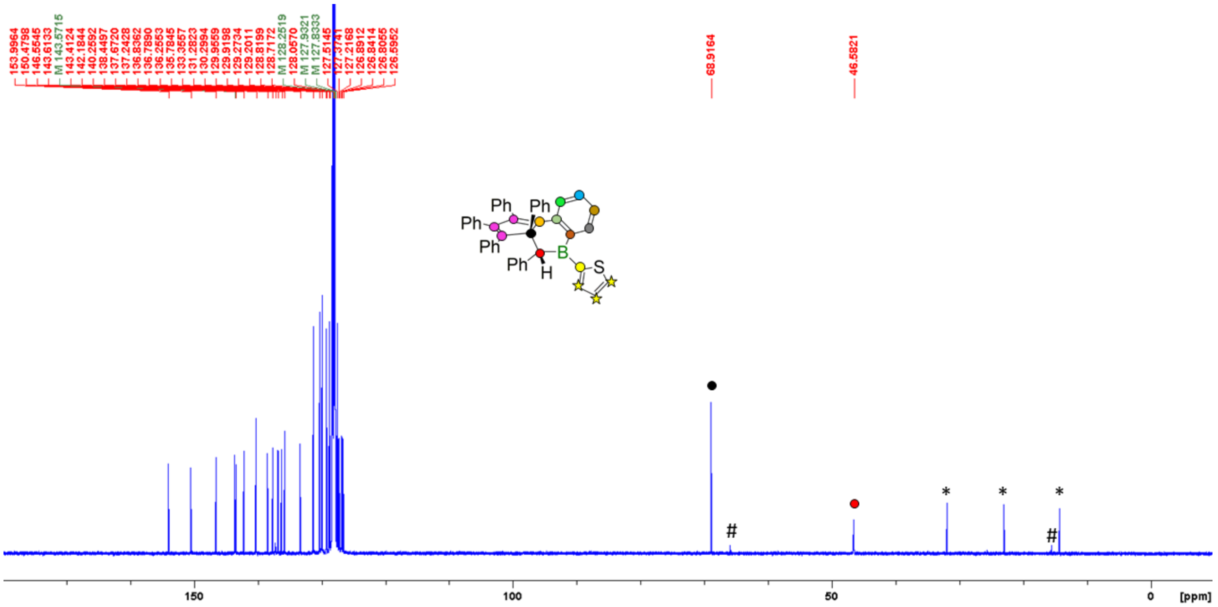

Figure S44. $^{13}C\{^1H\}$ NMR spectrum of **4b** in C_6D_6. The signals marked with a hash symbol (#) can be assigned to residual diethyl ether, the signals with an asterisk (*) to residual hexane.

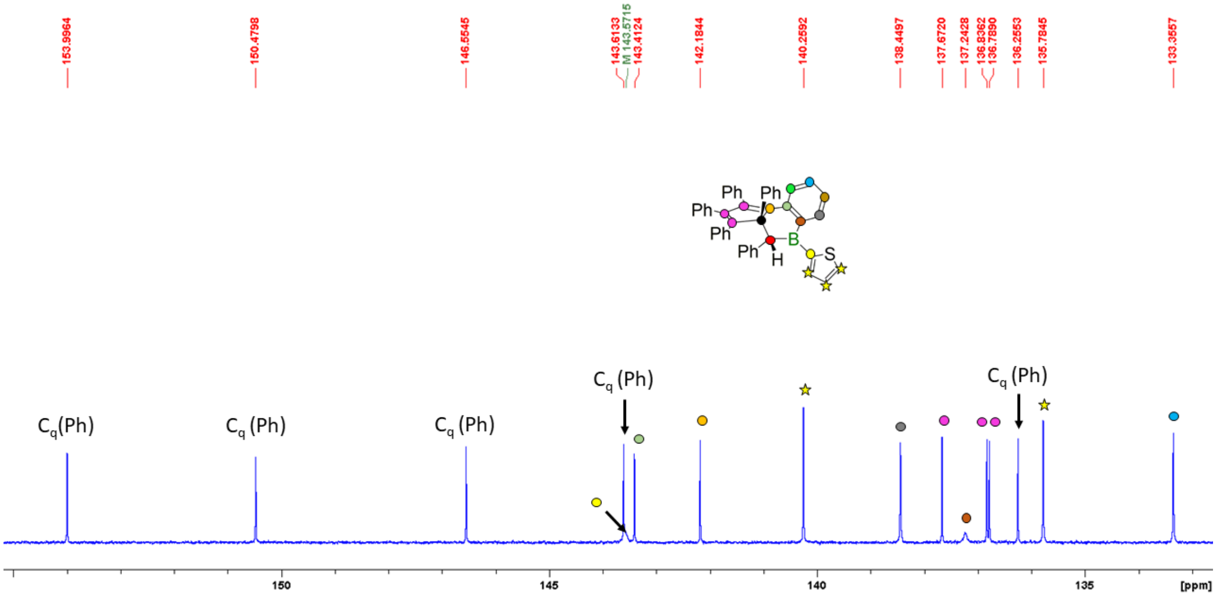

Figure S45. Expansion of the aromatic region (part 1of 2) of the $^{13}C\{^1H\}$ NMR spectrum of **4b** in C_6D_6.

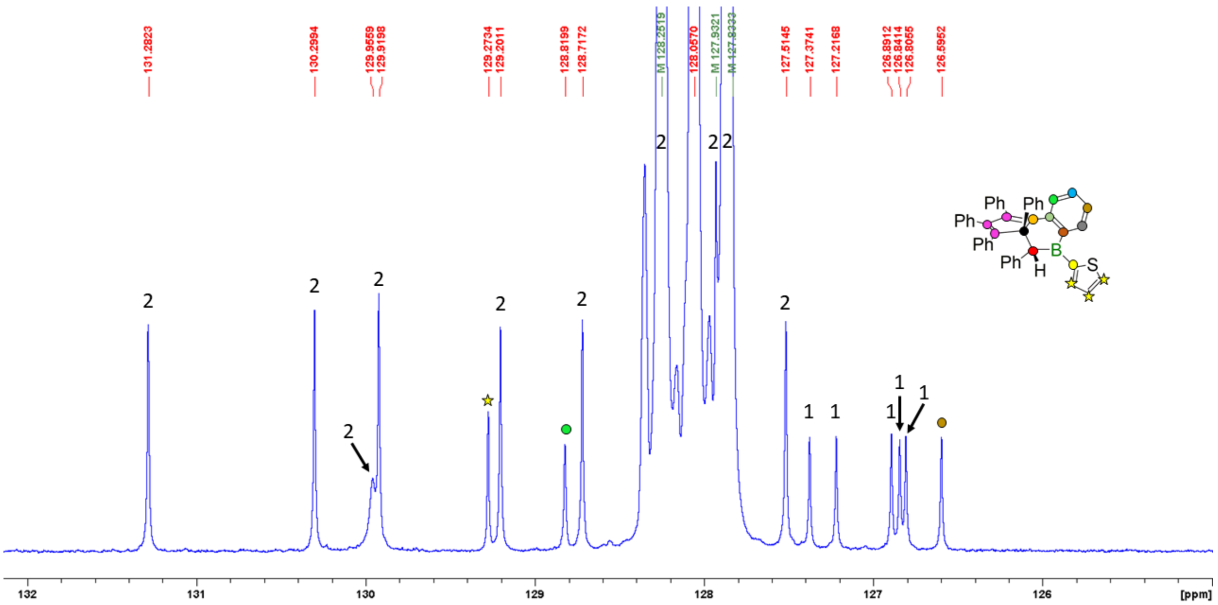

Figure S46. Expansion of the aromatic region (part 1 of 2) of the $^{13}C\{^{1}H\}$ NMR spectrum of **4b** in C_6D_6.

B.15. ^{13}C NMR data of 4c

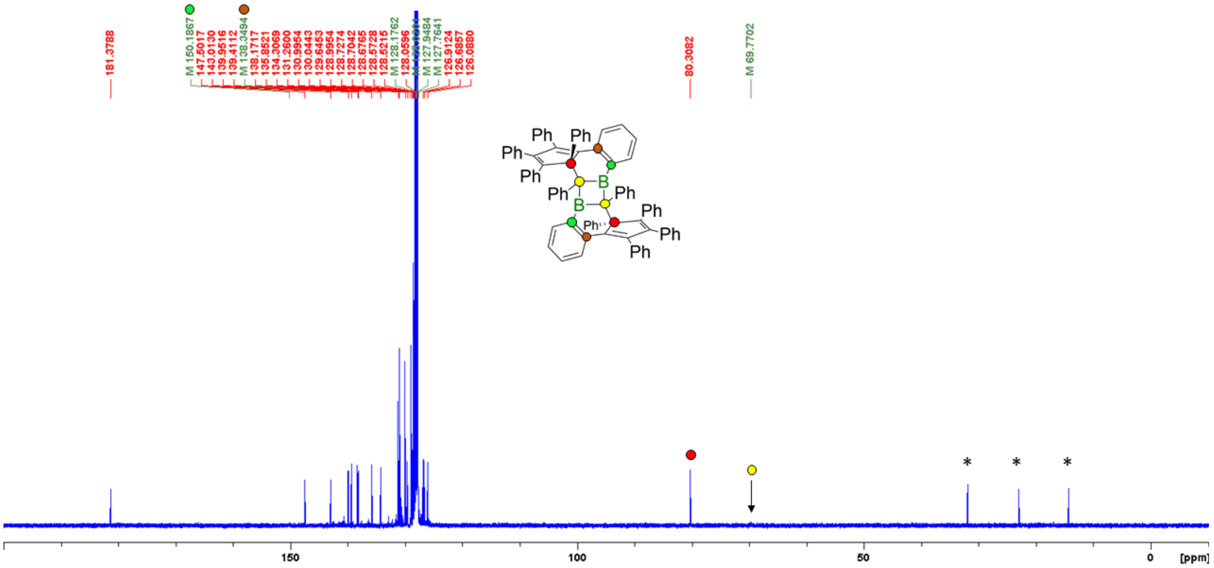

Figure S47. Expansion of the aromatic region of the $^{13}C\{^1H\}$ NMR spectrum of **4c** in C_6D_6. The signals marked with an asterisk (*) can be assigned to residual hexane.

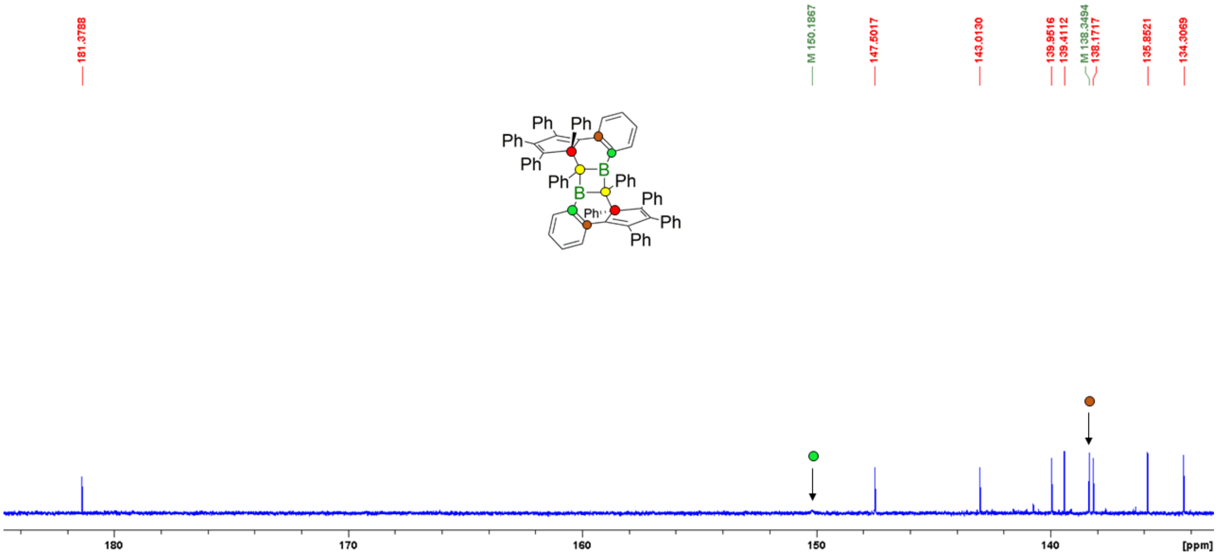

Figure S48. Expansion of the aromatic region (part 1 of 2) of the $^{13}C\{^1H\}$ NMR spectrum of **4c** in C_6D_6.

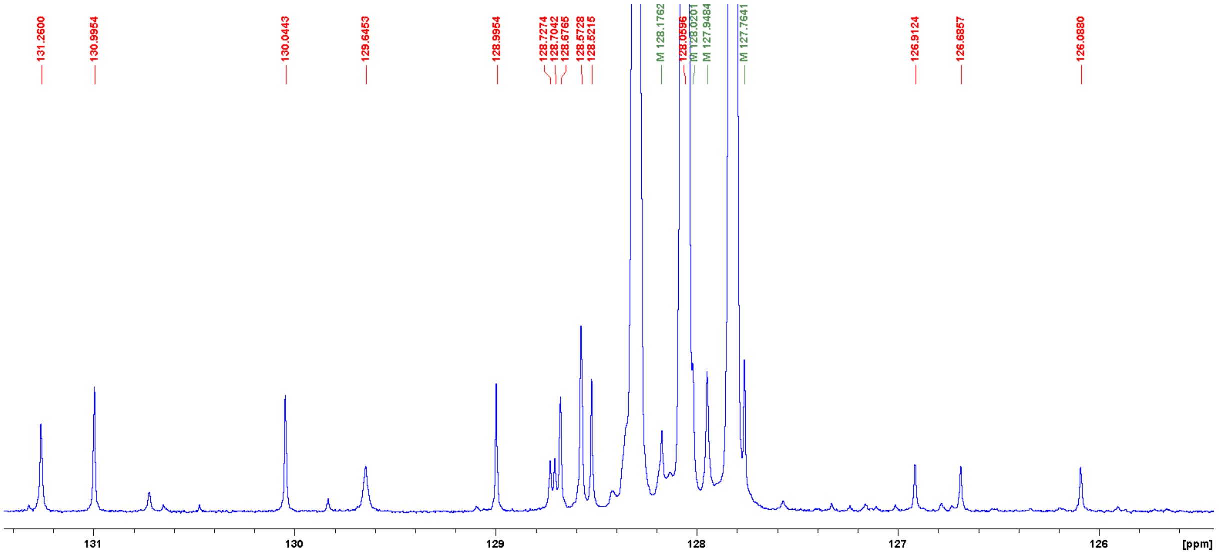

Figure S49. Expansion of the aromatic region (part 2 of 2) of the $^{13}C\{^{1}H\}$ NMR spectrum of **4c** in C_6D_6.

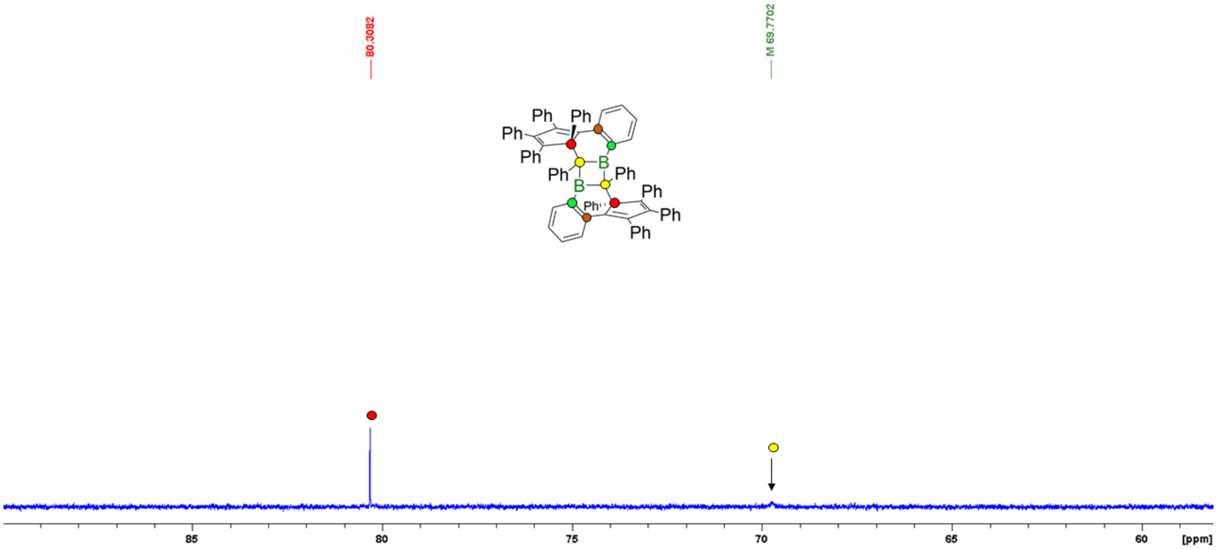

Figure S50. Expansion of the aliphatic region of the $^{13}C\{^{1}H\}$ NMR spectrum of **4c** in C_6D_6.

B.16. ^{11}B NMR data of 2b/3b

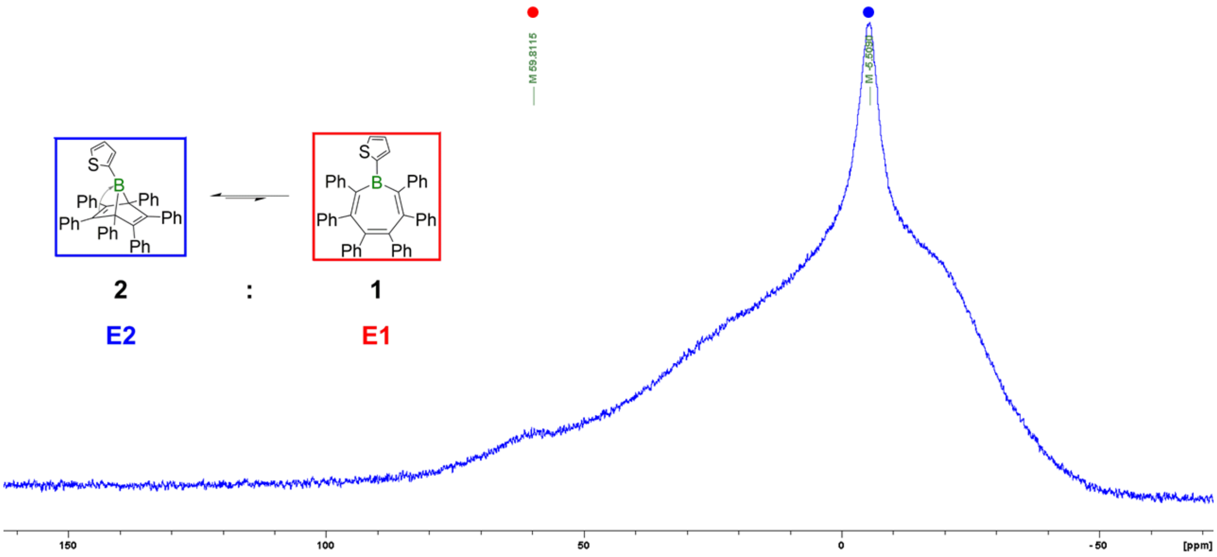

Figure S51. ^{11}B NMR spectrum of **2b/3b** (E2/E1) in CD_2Cl_2 at room temperature.

B.17. ^{11}B NMR data of 2c/3c

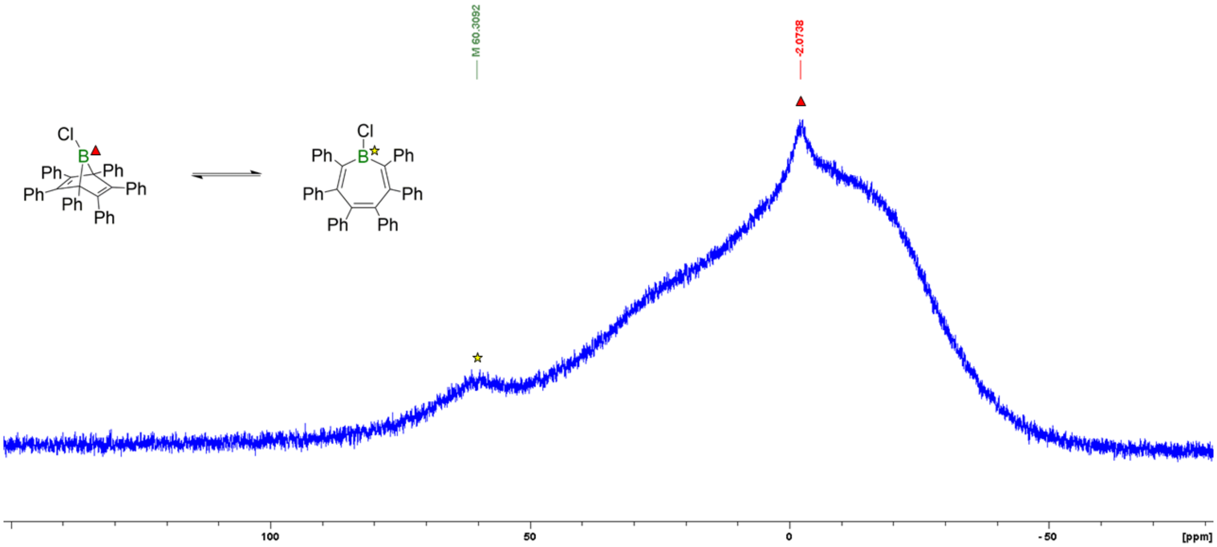

Figure S52. ^{11}B NMR spectrum of **2c/3c** in C_6D_6 at room temperature.

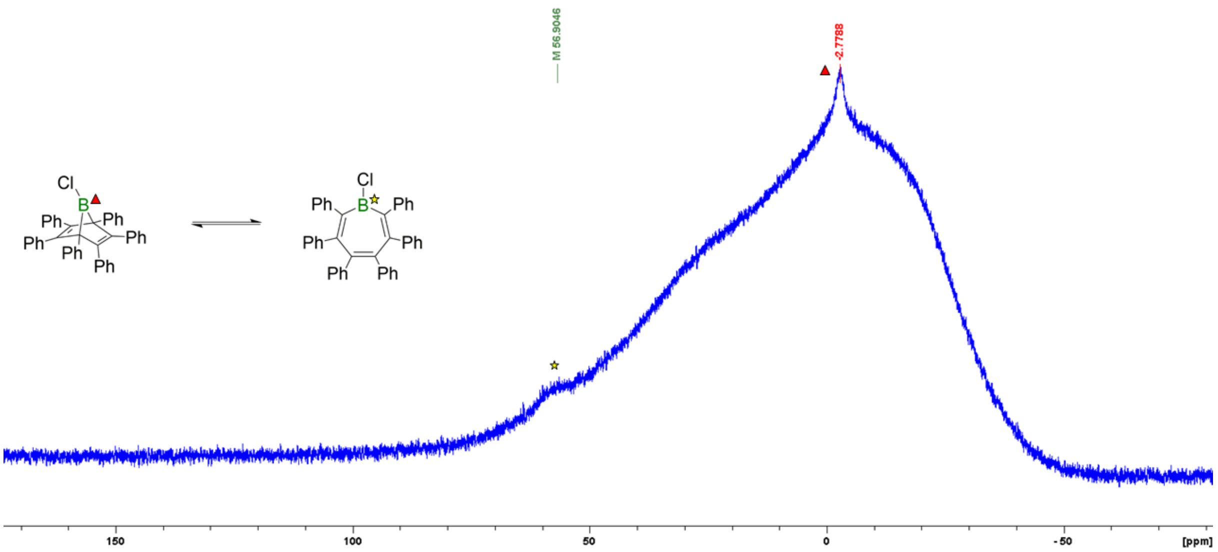

Figure S53. ^{11}B NMR spectrum of **2c/3c** in CD_2Cl_2 at room temperature.

B.18. ^{11}B NMR data of 3b(*I*Me)

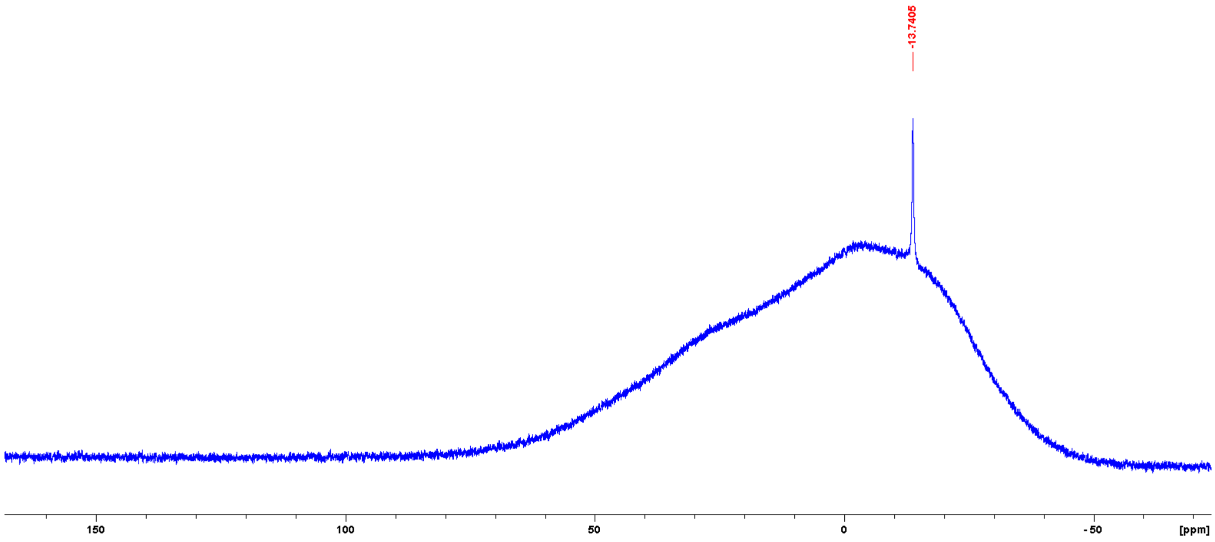

Figure S54. ^{11}B NMR spectrum of **3b(*I*Me)** in C_6D_6.

B.19. ^{11}B NMR data of 3c(*I*Me)

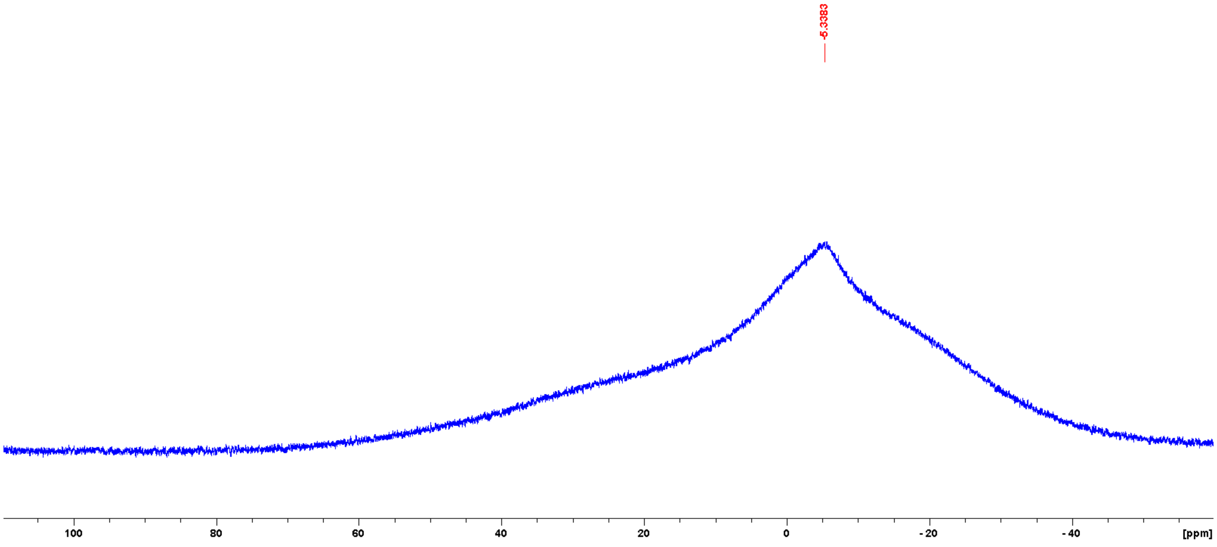

Figure S55. ^{11}B NMR spectrum of **3c(*I*Me)** in C_6D_6.

B.20. ^{11}B NMR data of 3c(thf)

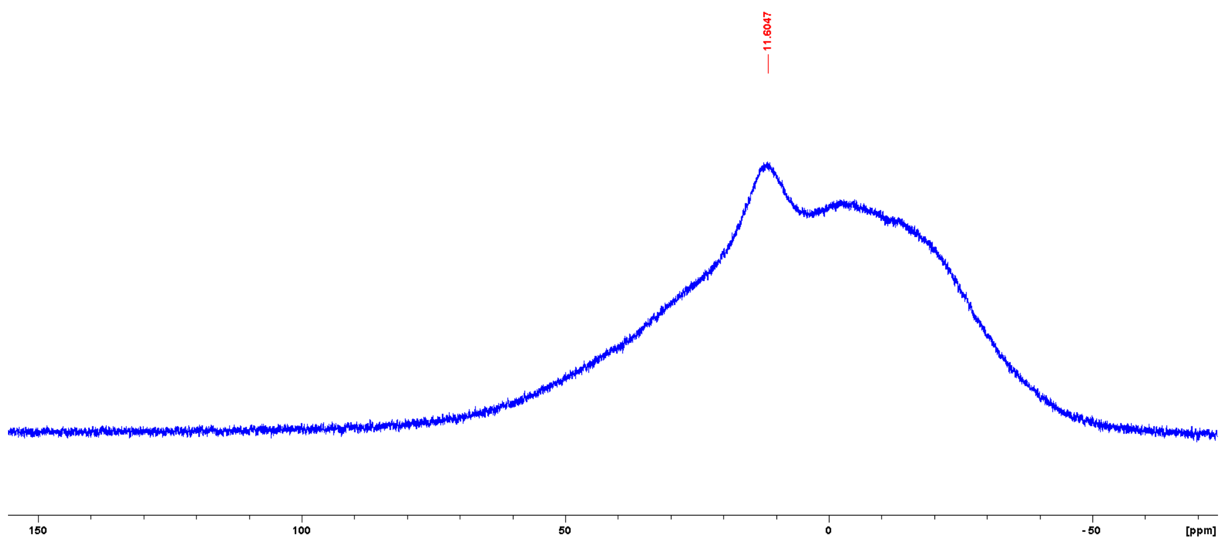

Figure S56. ^{11}B NMR spectrum of **3c(thf)** in C_6D_6.

B.21. ^{11}B NMR data of 4a

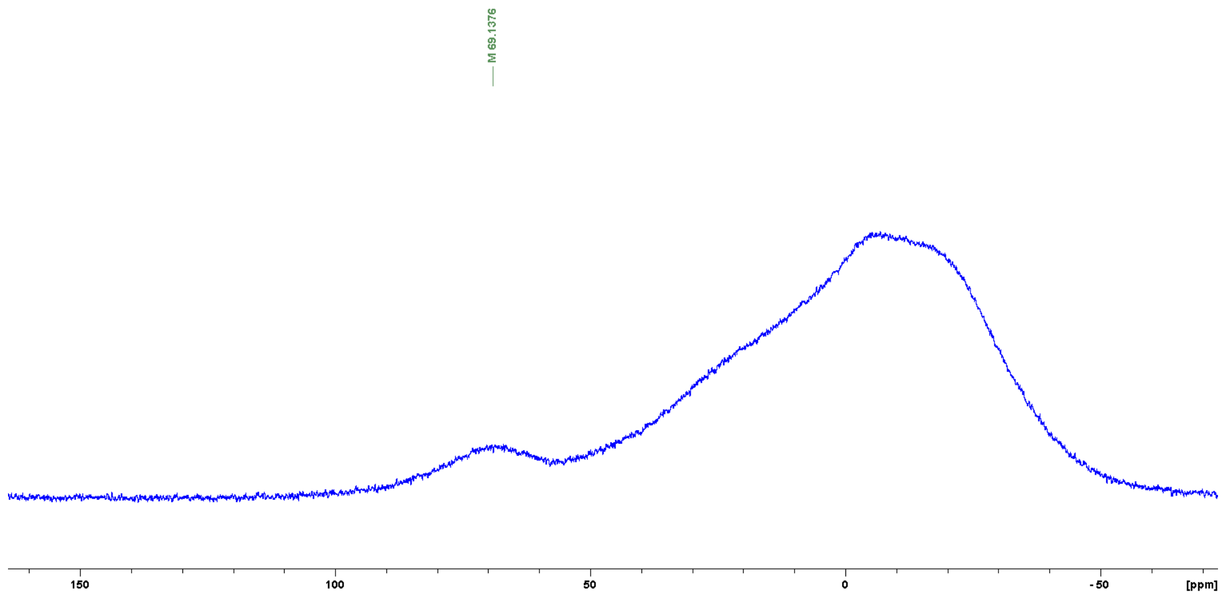

Figure S57. ^{11}B NMR spectrum of **4a** in C_6D_6.

B.22. ^{11}B NMR data of 4b

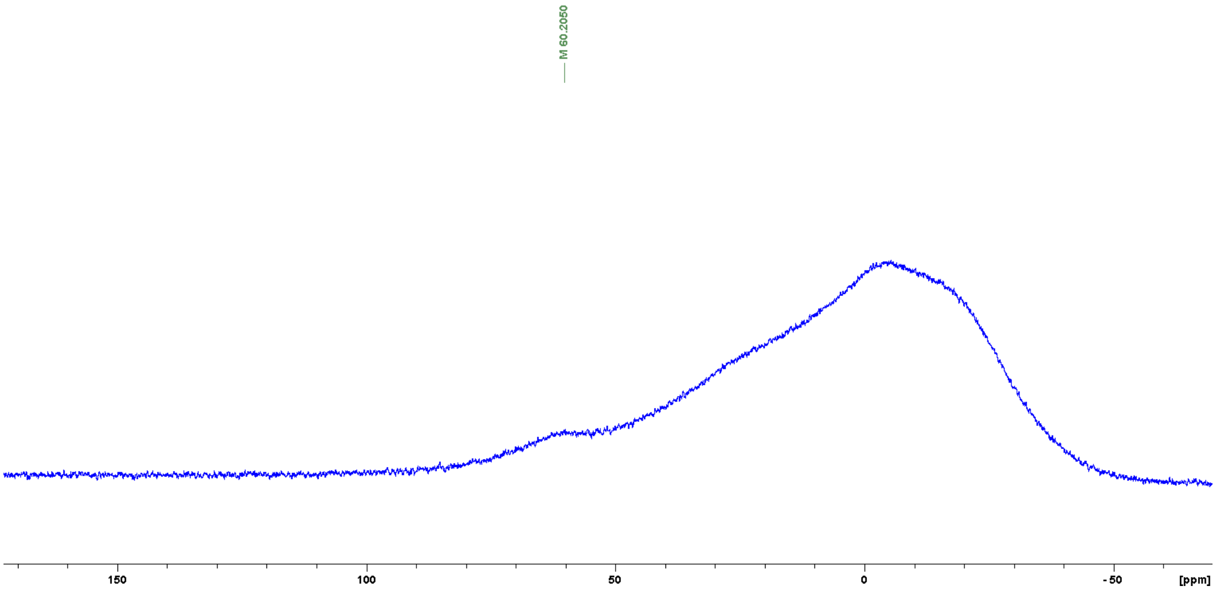

Figure S58. ^{11}B NMR spectrum of **4b** in C_6D_6.

B.23. ^{11}B NMR data of 4c

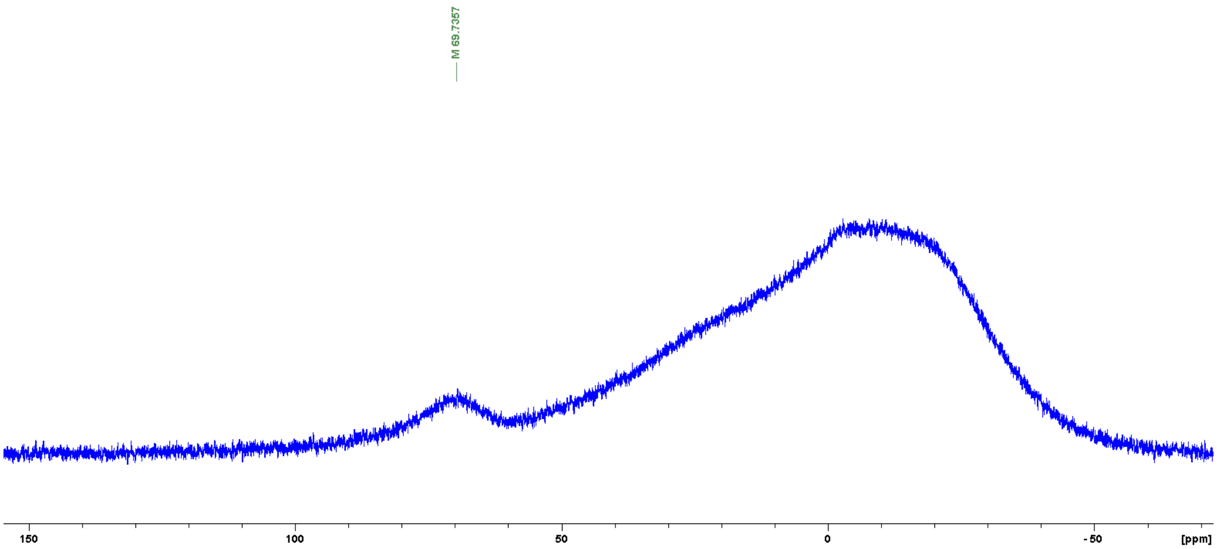

Figure S59. ^{11}B NMR spectrum of **4c** in C_6D_6.

B.24. Chemical Equilibrium of 2b/3b

The position of the equilibrium of **2b/3b** in CD_2Cl_2 solution at room temperature was determined to be 2:1. By cooling to –40 °C, the position of the equilibrium is changed in favor of the boranorbornadiene **2b**, resulting in an isomer distribution of 3:1. In C_6D_6 solution at room temperature, the position of the equilibrium was determined to be 3:1 (**2b/3b**). Therefore, isomer **2b** is slightly favored in d_6-benzene over CD_2Cl_2.

a) Variable emperature NMR spectra

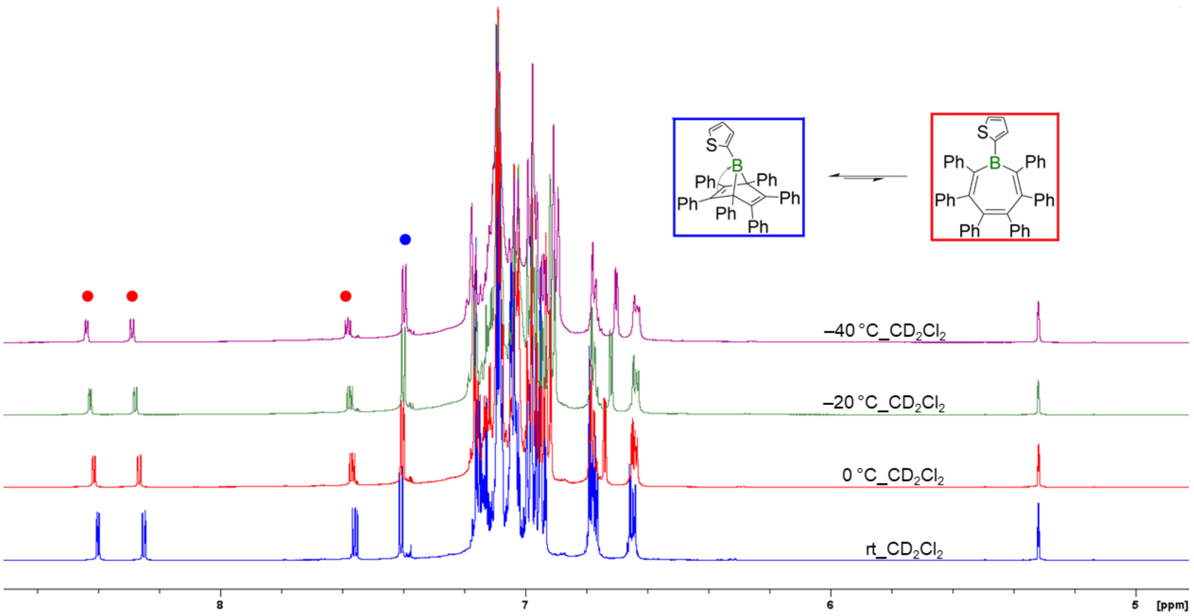

Figure S60. Variable-temperature ^{1}H NMR spectra (RT, 0 °C, –20 °C, –40 °C) of **2b/3b** in CD_2Cl_2.

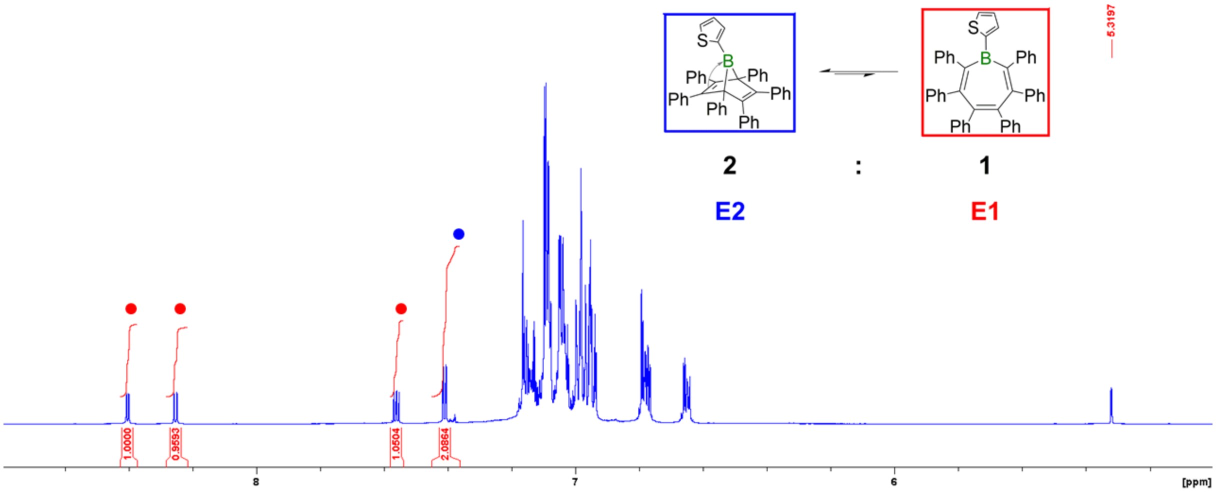

Figure S61. ^{1}H NMR spectrum of **2b/3b** (**E2**/**E1**) in CD_2Cl_2. The position of the equilibrium is estimated to be 2:1.

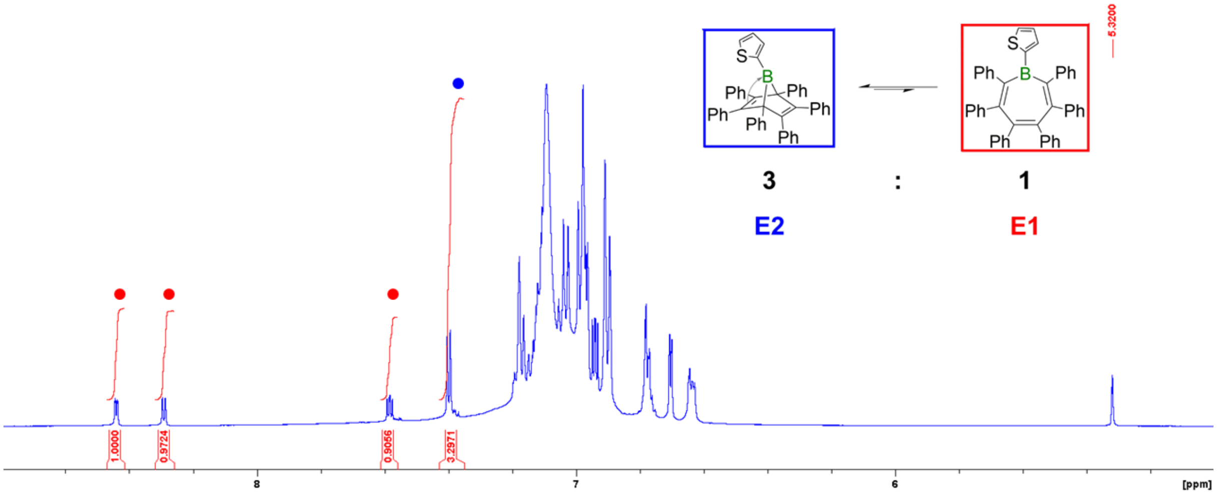

Figure S62. ^{1}H NMR spectrum of **2b/3b** (**E2**/**E1**) in CD_2Cl_2 at –40 °C. The position of the equilibrium between **2b/3b** is estimated to be ca. 3:1.

b) ^{1}H NMR spectra in C_6D_6

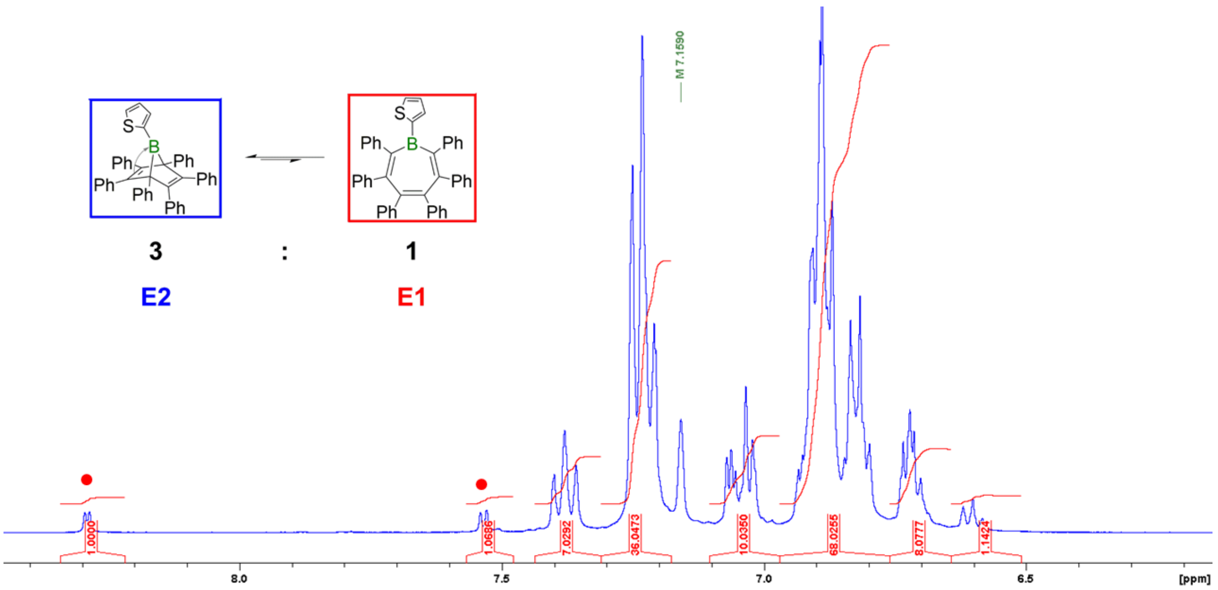

Figure S63. Excerpt of the aromatic region of the ^{1}H NMR spectrum of **2b/3b** (**E2**/**E1**) in C_6D_6 at room temperature. The sum of the integrals corresponds to 4 equivalents of molecules. The position of the equilibrium was estimated based on the characteristic resonances of the thienyl unit of borepin **3b** (**E1**). The equilibrium position is determined to be 3:1 (**2b/3b**).

B.25. NMR Studies, Part One: Carbene Adduct Formation

a) Reaction of 2a/3a with *I*Me

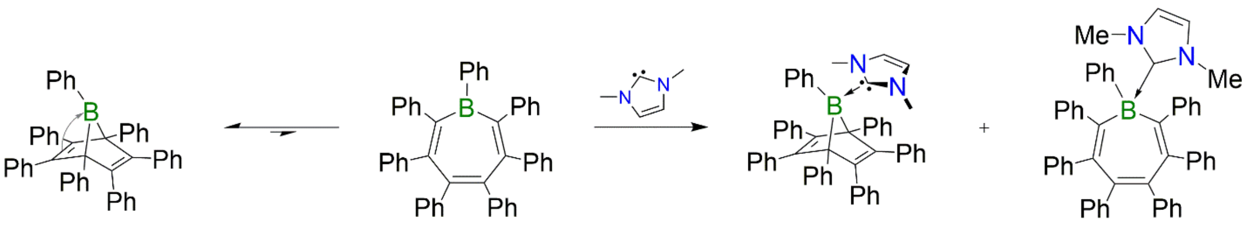

Q **X** **Y** **Z**

A solution of **Q** and **X** (27.3 mg, 0.04 mmol) in chemical equilibrium in 0.6 mL of benzene was treated with *I*Me (4.2 mg, 0.04 mmol) at room temperature. The reaction mixture was stirred at room temperature overnight and subsequently heated to 80 °C over a period of 5 h, whereupon **Y** was fully converted into borepin **Z**.

^{11}B NMR (129 MHz, C_6D_6): δ = –3.2 ppm. (**Q**)

^{11}B NMR (129 MHz, C_6D_6): δ = *not detected* (**X**)

^{11}B NMR (129 MHz, C_6D_6): δ = 12.6 ppm. (**Y**)

^{11}B NMR (129 MHz, C_6D_6): δ = –12.2 ppm. (**Z**)

^{1}H NMR spectra

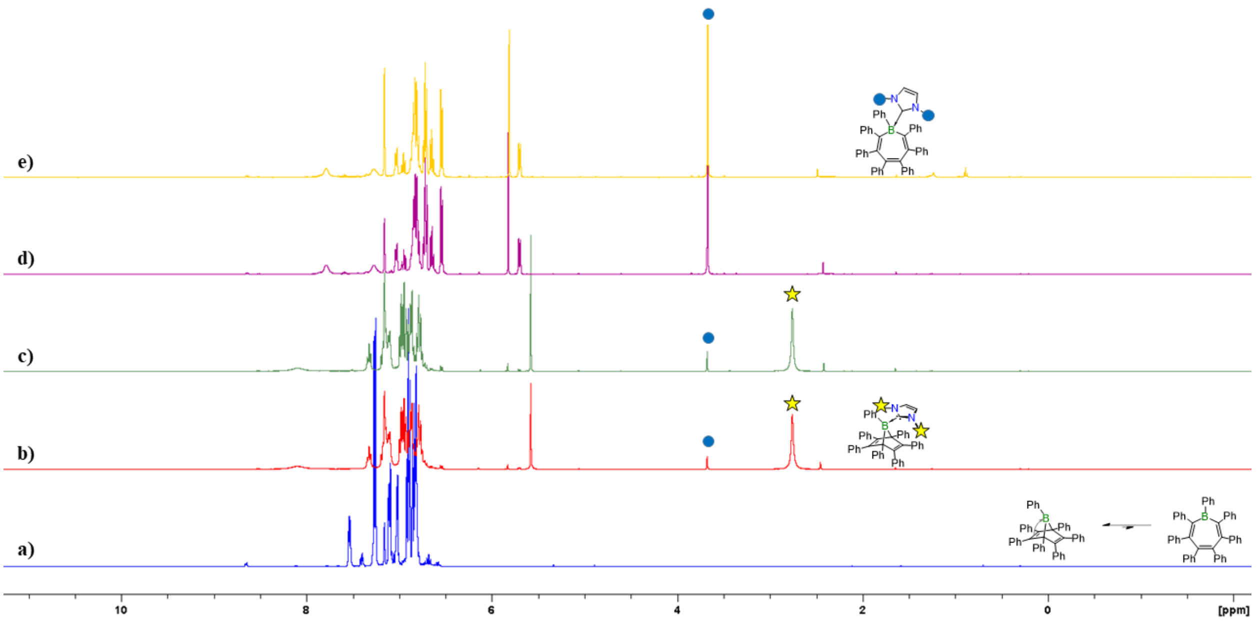

Figure S64. *In-situ* ^{1}H NMR spectra in C_6D_6 after various time points. a) Compounds **2a/3a** before addition. b) 15 min after addition of *I*Me at room temperature. c) 15 h at room temperature. d) After heating to 80 °C for 5 h. e) Isolated compound **Z**.

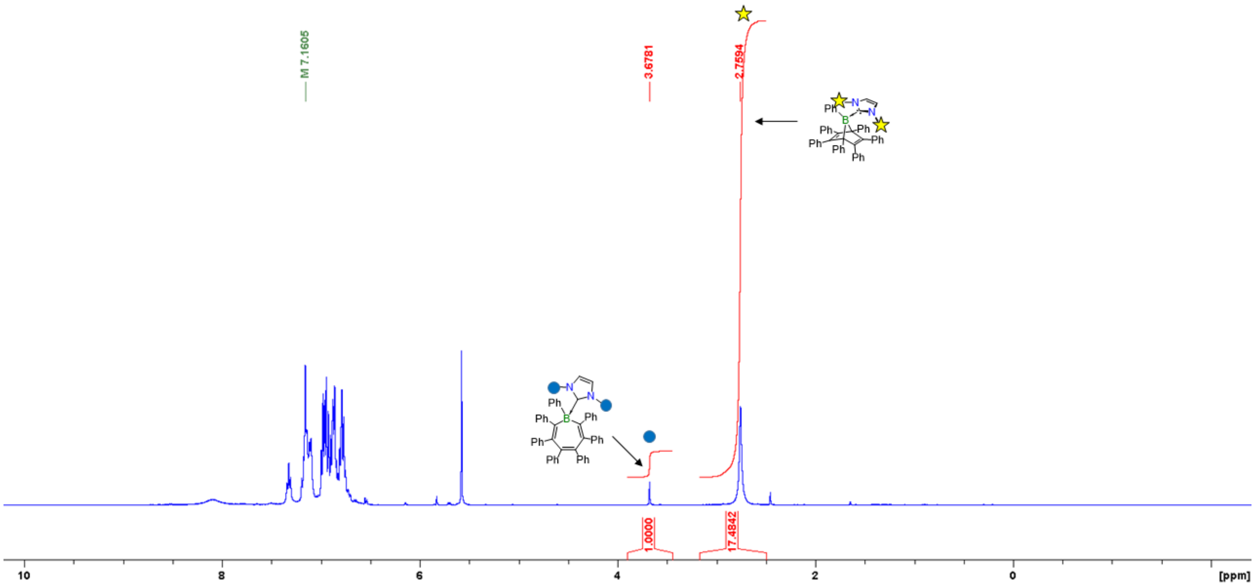

Figure S65. ^{1}H NMR spectrum of **2a/3a** immediately after addition of *I*Me at room temperature.

^{11}B NMR spectra

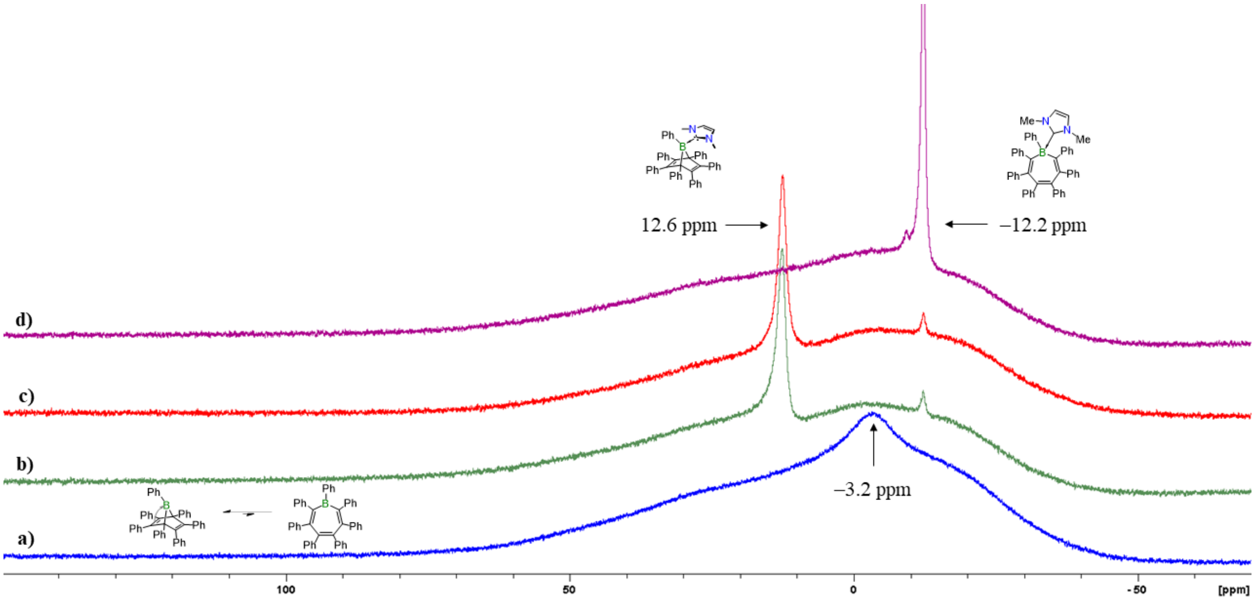

Figure S66. *In-situ* ^{11}B NMR spectra of **2a/3a** in C_6D_6 after various time points. a) Before addition. b) 15 min after addition of *I*Me at room temperature. c) 15 h at room temperature. d) After heating at 80 °C for 5 h.

b) Reaction of 2b/3b with *I*Me

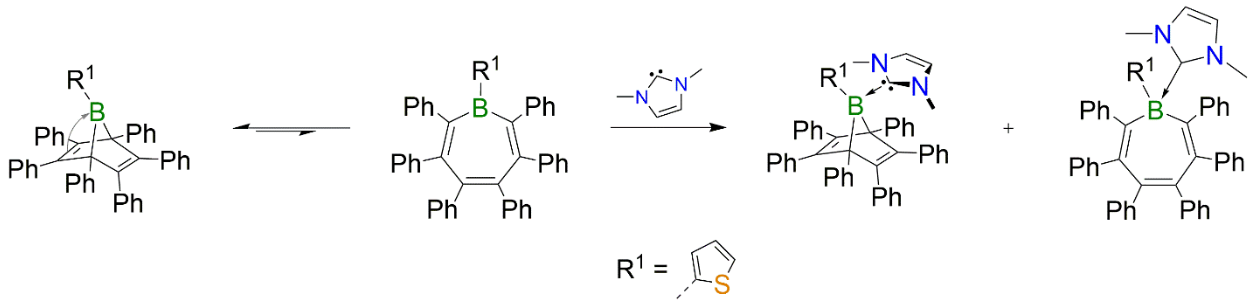

Q **X** **Y** **Z**

A solution of **Q** and **X** (43.0 mg, 0.07 mmol) in chemical equilibrium in 0.6 mL of benzene was treated with *I*Me (8.0 mg, 0.04 mmol) at room temperature. The reaction mixture was heated to 80 °C for 3 d. During this time, **Y** was not fully converted to compound **Z**.

^{11}B NMR (129 MHz, C_6D_6): δ = –4.3 ppm. (**Q**)

^{11}B NMR (129 MHz, C_6D_6): δ = *not detected* (**X**)

^{11}B NMR (129 MHz, C_6D_6): δ = 10.2 ppm. (**Y**)

^{11}B NMR (129 MHz, C_6D_6): δ = –13.7 ppm. (**Z**)

Comment: We were able to characterize compound **Y** (or **2b(*I*Me)**) via NMR spectroscopy, directly after addition of *I*Me (*vide infra*).

^{1}H NMR spectra

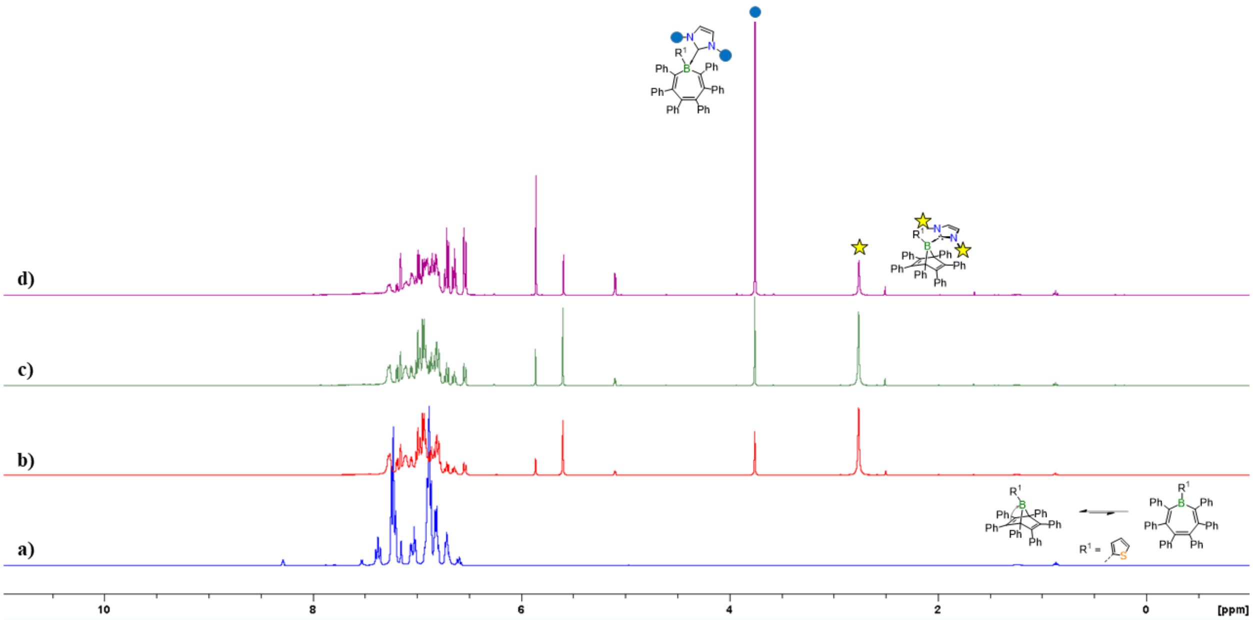

Figure S67. *In-situ* ^{1}H NMR spectroscopy of **2b/3b** in C_6D_6. a) Before addition. b) 15 min after addition of *I*Me at room temperature. c) After heating at 80 °C for 15 h. d) After 3 d of heating at 80 °C.

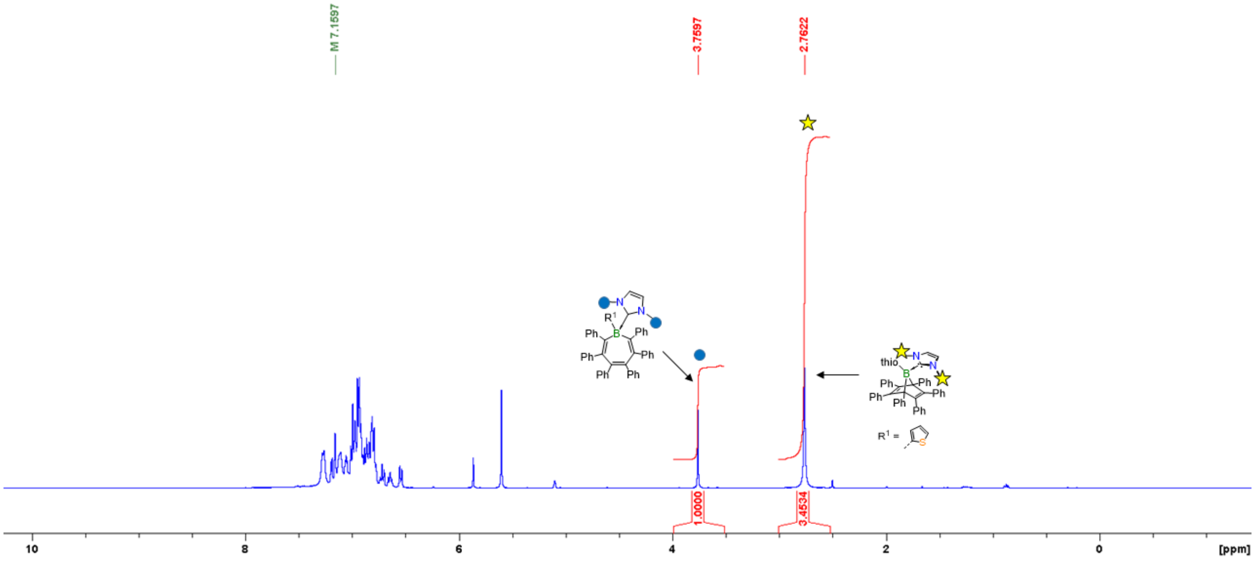

Figure S68. ^{1}H NMR spectrum of **2b/3b** in C_6D_6 immediately after addition of *I*Me at room temperature.

^{11}B NMR spectra

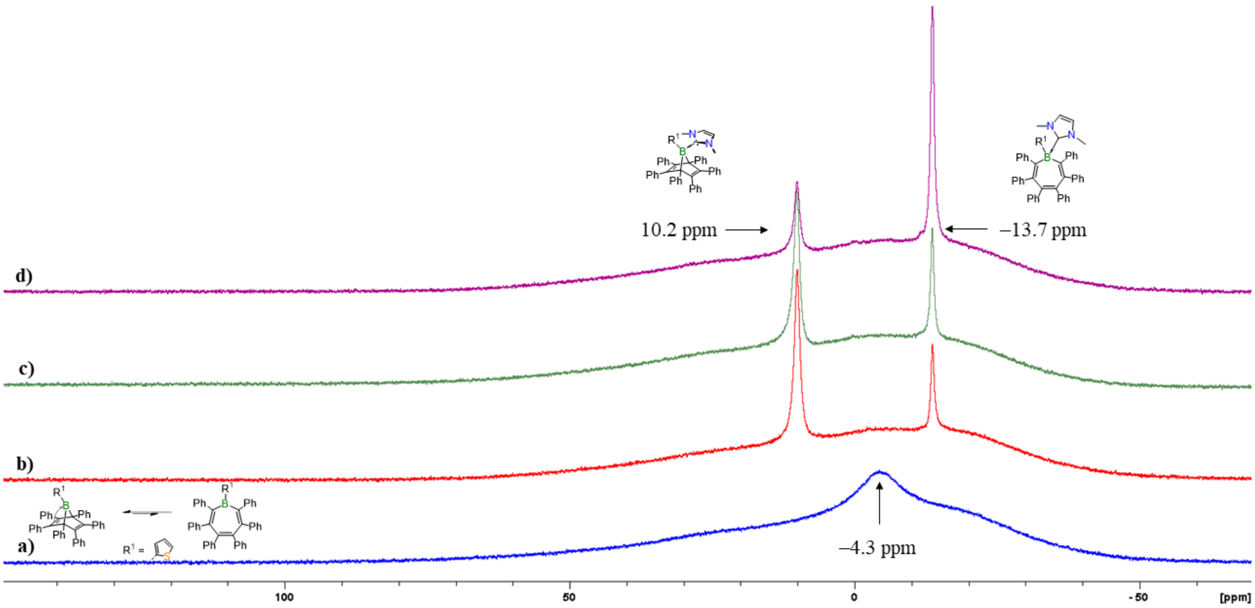

Figure S69. *In-situ* ^{11}B NMR spectra of **2b**/**3b** in C_6D_6. a) Before addition. b) 15 min after addition of *I*Me at room temperature. c) After 15 h of heating at 80 °C. d) After 3 d of heating at 80 °C.

NMR spectroscopic characterization of compound 2b(*I*Me)

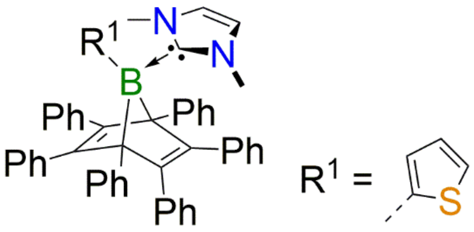

$C_{51}H_{41}N_2BS$ (724.775 gmol^{-1})

A suspension of **2b/3b** (40.0 mg, 0.06 mmol) in 0.5 mL of C_6D_6 was treated dropwise with a solution of *I*Me (6.20 mg, 0.06 mmol) in 0.2 mL of C_6D_6. NMR data was collected immediately after addition.

^{1}H NMR (500 MHz, C_6D_6): δ = 7.38–7.21 (m, 4H, C*H*, C_6H_5), 7.21–7.18 (m, 1H, C*H*, C_4H_3S), 7.15–7.07 (m, 4H, C*H*, C_6H_5), 7.07–7.04 (m, 1H, C*H*, C_4H_3S), 7.04–6.89 (m, 14H, C*H*, C_6H_5), 6.89–6.75 (m, 8H, C*H*, C_6H_5 + 1H, C*H*, C_4H_3S), 5.60 (s, 2H, C*H*, NCN(C*H*)$_2$), 2.77 (s, 6H, C*H*$_3$, (NC*H*$_3$)$_2$) ppm.

^{13}C{^{1}H} NMR (101 MHz, C_6D_6): δ = 164.6 (C_q, 1C, N*C*N(CH)$_2$)†, 155.3 (C_q, 2C), 153.7 (C_q, 2C), 142.8 (C_q, 2C), 141.2 (C_q, 2C), 140.7 (C_q, 2C), 133.4 (*C*H, 4C), 132.2 (C_q, 1C, C_4H_3S), 131.7 (*C*H, 4C), 130.9 (*C*H, 4C), 127.6 (*C*H, 4C), 127.6 (*C*H, 4C + *C*H, 1C, C_4H_3S), 126.7 (*C*H, 1C, C_4H_3S), 126.3 (*C*H, 4C), 125.8 (*C*H, 2C), 125.6 (*C*H, 1C, C_4H_3S), 125.4 (*C*H, 2C), 124.1 (*C*H, 2C), 121.9 (*C*H, 2C, NCN(*C*H)$_2$), 72.5 (C_q, 2C_B)†, 38.6 (*C*H$_3$, 2C, (N*C*H$_3$)$_2$).

Comment: The signals marked with a cross (†) are resonances of boron- and nitrogen-bound quaternary carbon atoms. These signals were detected by means of ^{13}C DEPT-135/^{13}C{^{1}H},^{1}H HMBC experiments.

^{11}B NMR (128 MHz, C_6D_6): δ = 10.2 ppm.

^{11}B NMR (161 MHz, C_6D_6): δ = 10.0 ppm.

^{1}H NMR spectrum

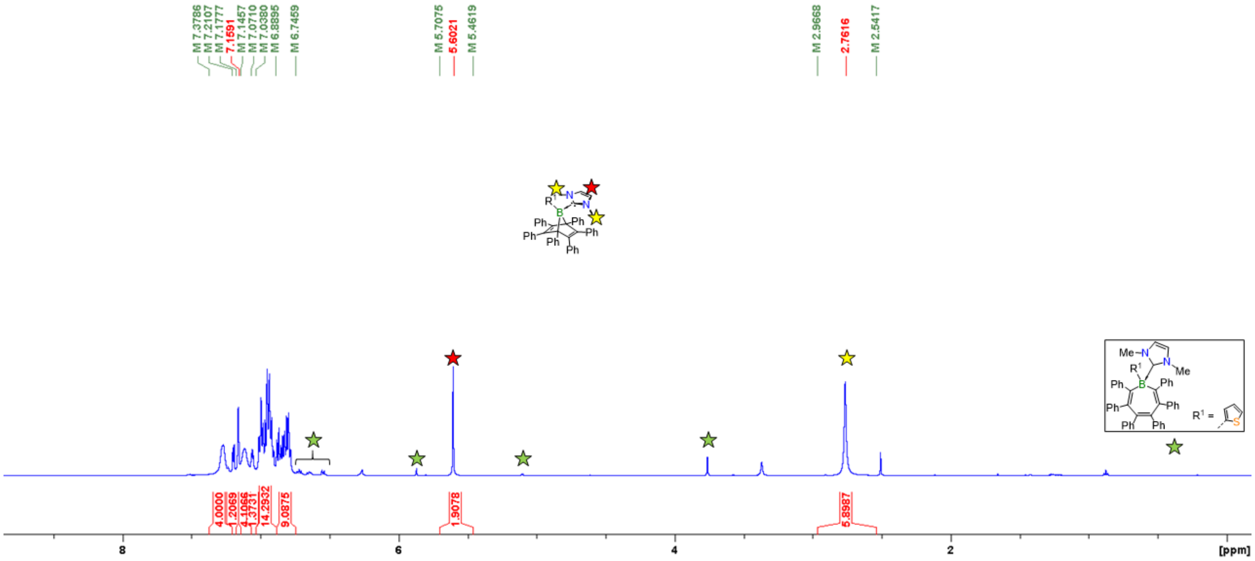

Figure S70. ^{1}H NMR spectrum of **2b(*I*Me)** in C_6D_6 immediately after addition of *I*Me to **2b/3b**.

^{13}C{^{1}H} NMR spectrum

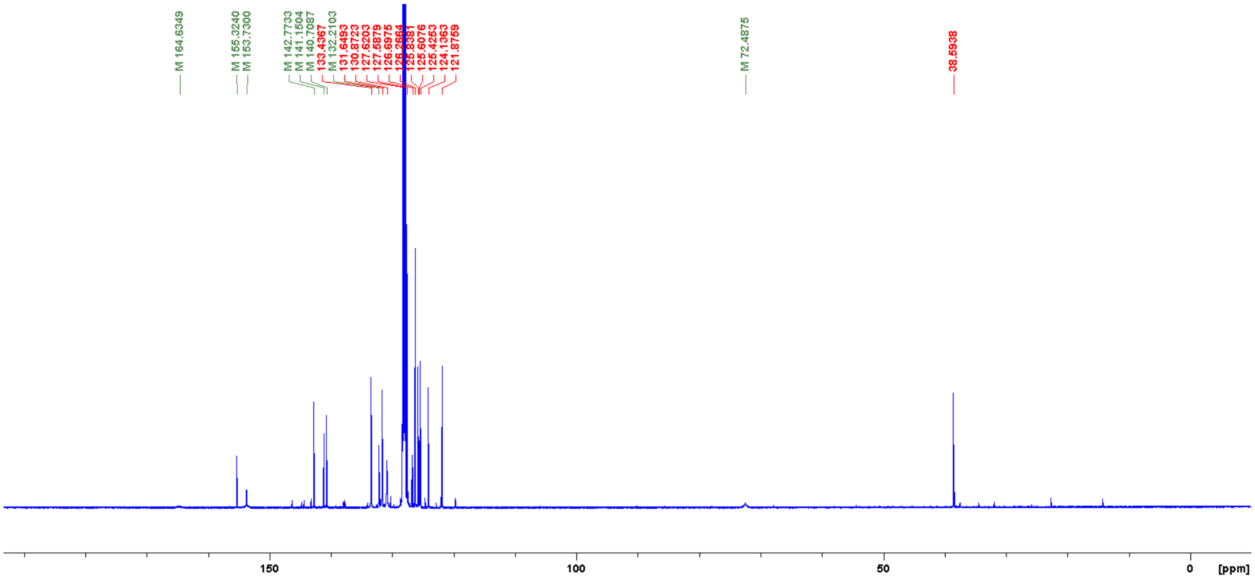

Figure S71. ^{13}C{^{1}H} NMR spectrum of **2b(*I*Me)** in C_6D_6 immediately after addition of *I*Me to **2b/3b**.

^{11}B NMR spectrum

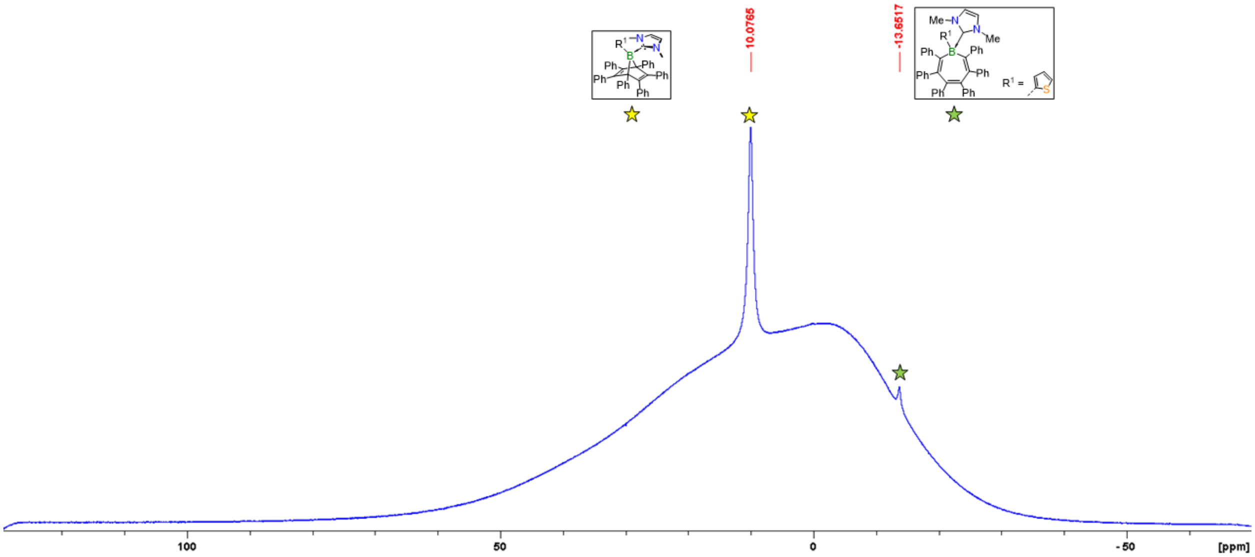

Figure S72. ^{11}B NMR spectrum of **2b(*I*Me)** in C_6D_6 immediately after addition of *I*Me to **2b/3b**.

c) Reaction of 2c/3c with *I*Me

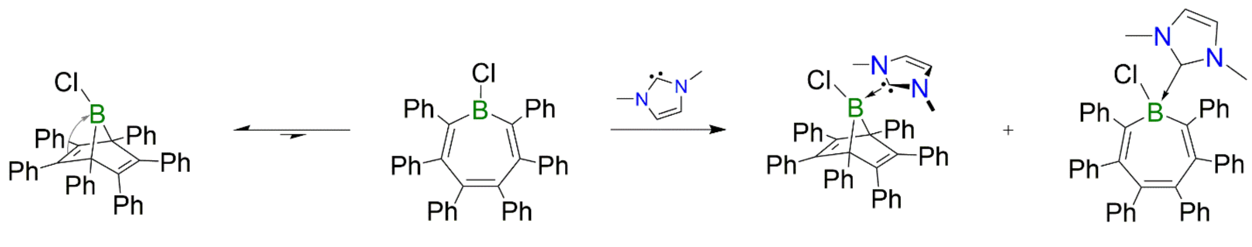

Q X Y Z

A suspension of **Q** and **X** (43.0 mg, 0.07 mmol) in chemical equilibrium in 0.6 mL of benzene was treated with *I*Me (8.0 mg, 0.04 mmol) at room temperature. Immediately after the addition, the product distribution between **Y** and **Z** was determined. The reaction mixture was heated at 80 °C for 15h. During this time, **Y** was not fully converted to **Z**. Due to the poor solubility of borepin **Z** in benzene, large quantities of it precipitate over the course of the reaction.

^{11}B NMR (129 MHz, C_6D_6): δ = –2.1 ppm. (**Q**)

^{11}B NMR (129 MHz, C_6D_6): δ = 60.8 ppm. (**X**)

^{11}B NMR (129 MHz, C_6D_6): δ = 10.2 ppm. (**Y**)

^{11}B NMR (129 MHz, C_6D_6): δ = –5.3 ppm. (**Z**)

^{1}H NMR spectra

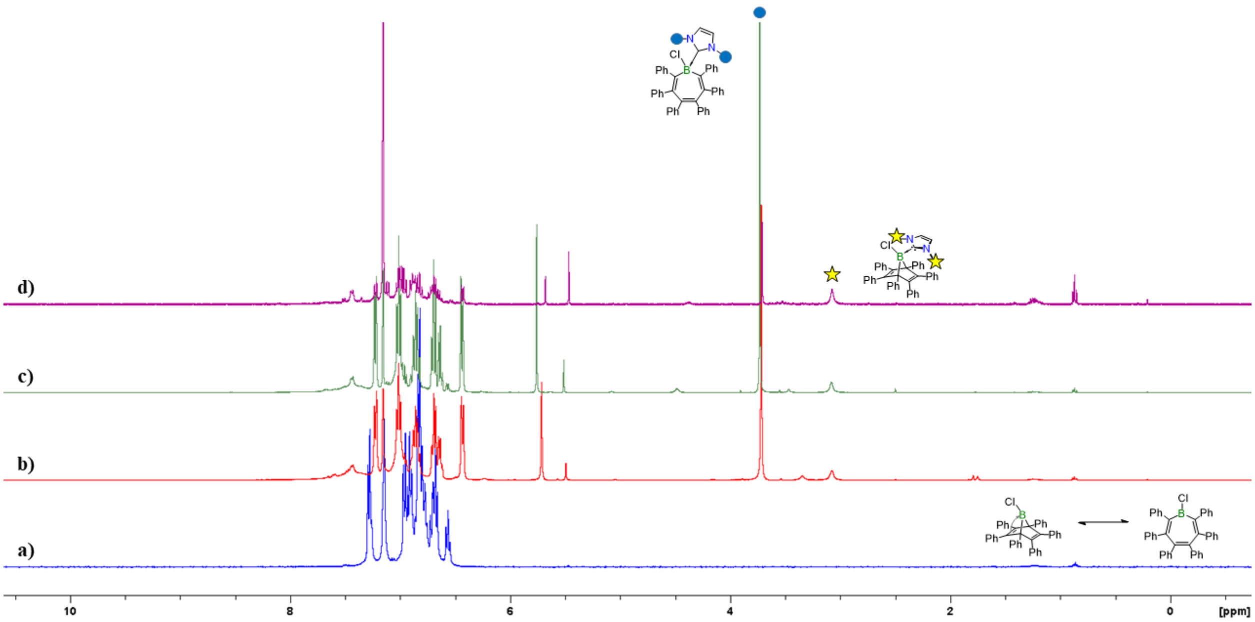

Figure S73. *In-situ* ^{1}H NMR spectra of **2c/3c** in C_6D_6. a) Before addition of *I*Me. b) 15 min after addition of *I*Me at room temperature. c) After 15 h of heating at 80 °C. d) After 15 h of heating at 80 °C and storage at room temperature for 90 h.

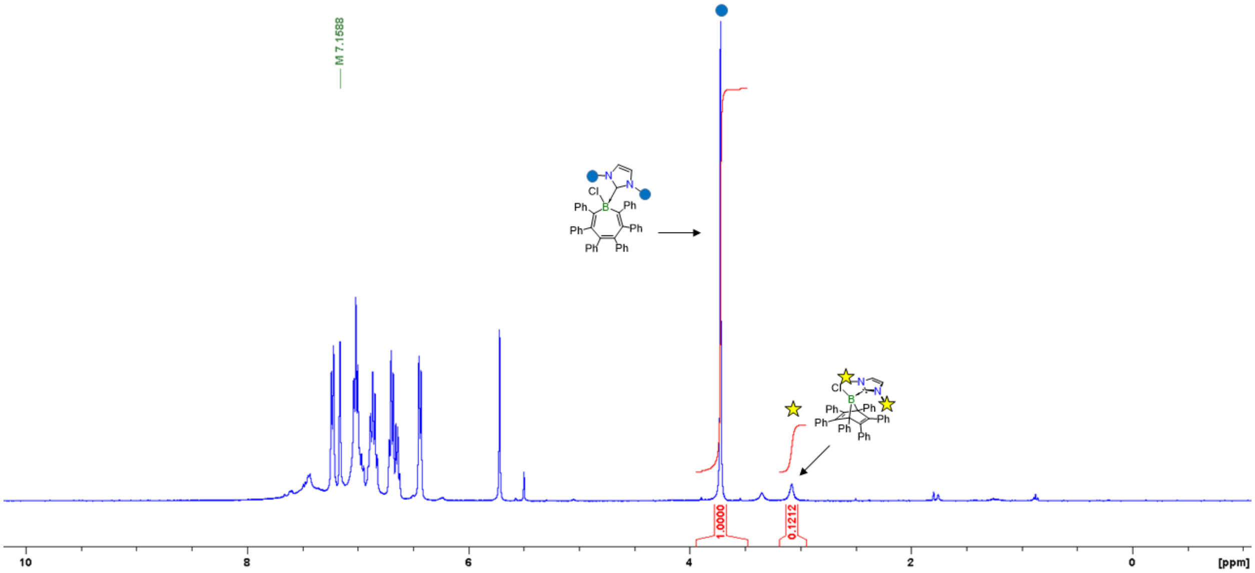

Figure S74. ^{1}H NMR spectrum of **2c/3c** in C_6D_6 immediately after addition of *I*Me at room temperature.

^{11}B NMR spectra

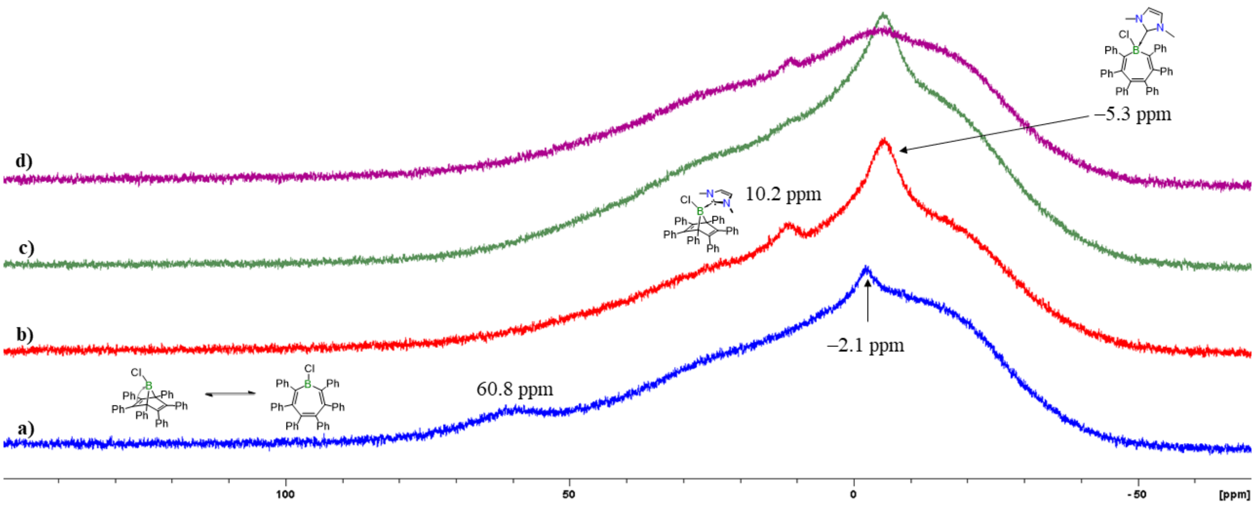

Figure S75. *In-situ* ^{11}B NMR spectra of **2c/3c** in C_6D_6. a) Before addition of *I*Me. b) 15 min after addition of *I*Me at room temperature. c) After 15 h of heating at 80 °C. d) After 15 h of heating at 80 °C, followed by storage at room temperature for 90 h.

B.26. NMR Studies, Part Two: THF Adduct Formation

a) Reaction of 2a/3a with THF

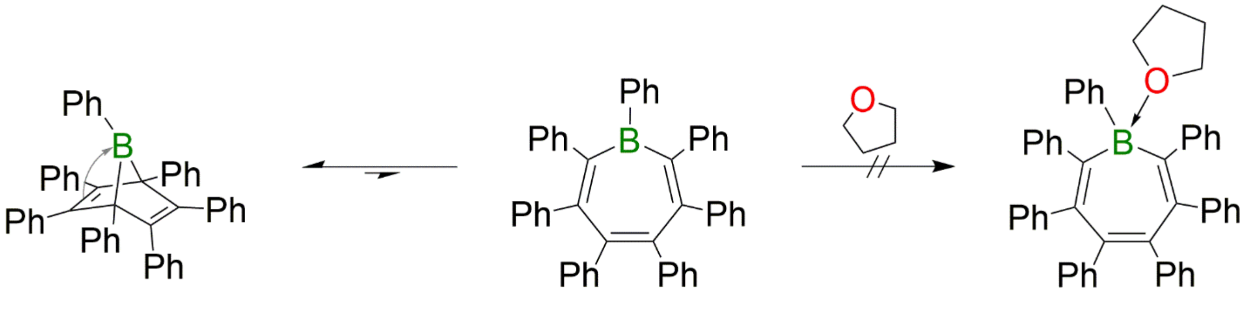

Q **X** **Y**

A solution of **Q** and **X** (30.0 mg, 0.05 mmol) in chemical equilibrium in 0.6 mL of C_6D_6 was treated with THF (8.3 mg, 0.12 mmol) and stirred at room temperature for 15 h. No adduct formation was observed according to ^{1}H and ^{11}B NMR spectroscopy.

^{11}B NMR (129 MHz, C_6D_6): δ = –2.1 ppm. (**Q**)

^{11}B NMR (129 MHz, C_6D_6): δ = *not detected* (**X**)

^{1}H NMR spectra

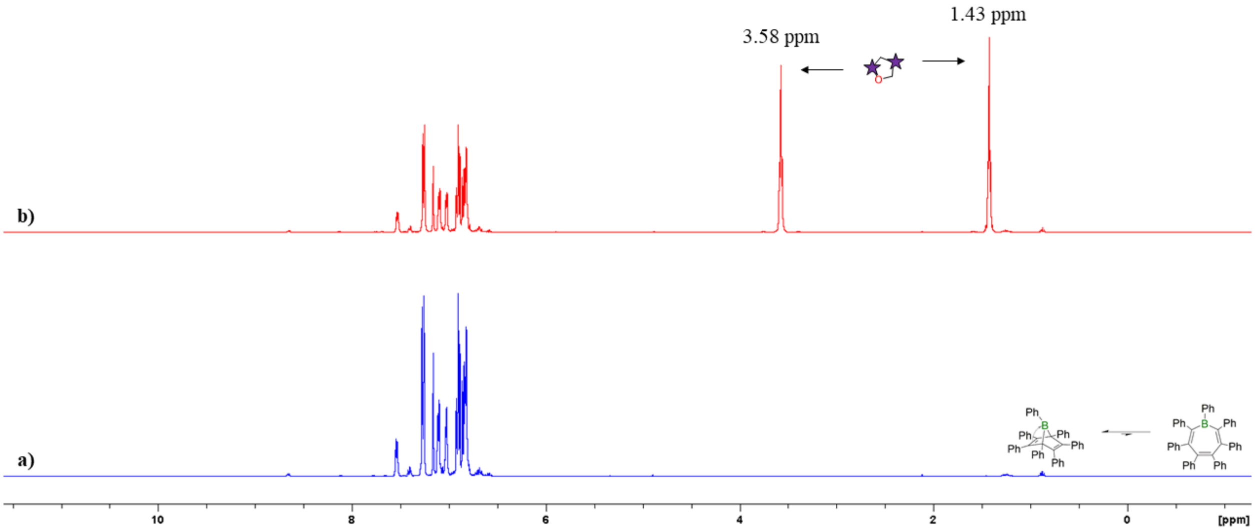

Figure S76. *In-situ* ^{1}H NMR spectra of **2a/3a** in C_6D_6. a) Before THF addition. b) 15 h after addition of THF at room temperature.

^{11}B NMR spectra

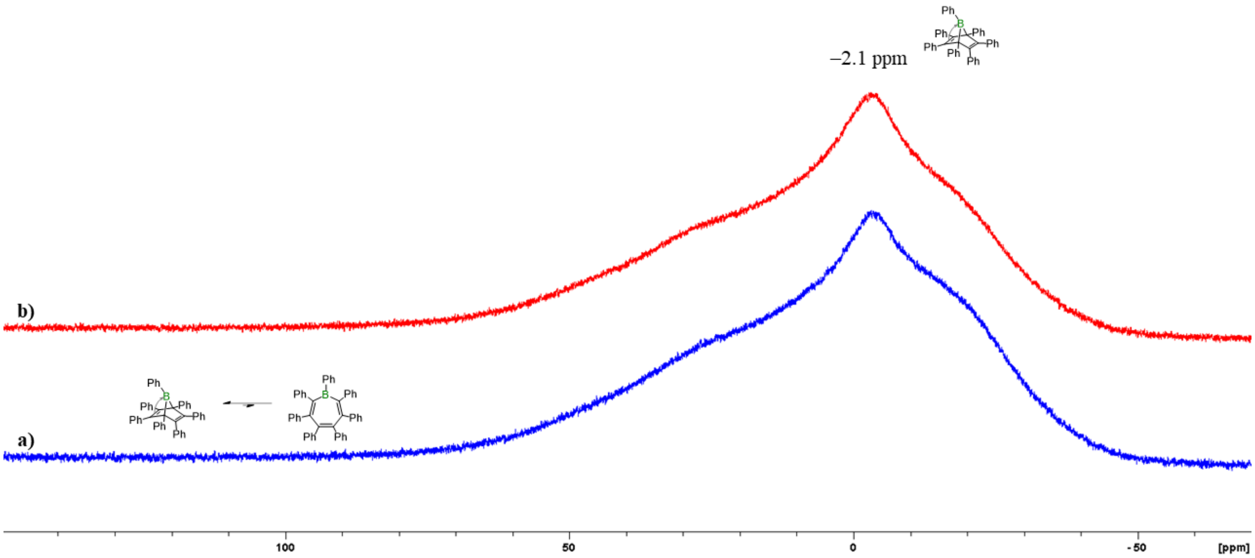

Figure S77. *In-situ* ^{11}B NMR spectra of **2a/3a** in C_6D_6. a) Before THF addition. b) 15 h after addition of THF at room temperature.

b) Reaction of 2b/3b with THF

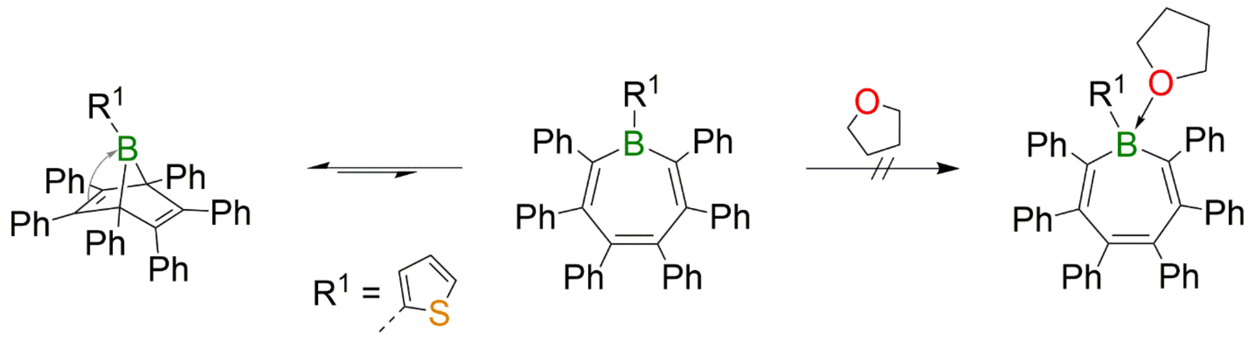

Q **X** **Y**

A solution of **Q** and **X** (30.0 mg, 0.05 mmol) in chemical equilibrium in 0.6 mL of C_6D_6 was treated with THF (8.0 mg, 0.11 mmol) and stirred at room temperature for 15 h. No adduct formation was observed according to ^{1}H and ^{11}B NMR spectroscopy.

^{11}B NMR (129 MHz, C_6D_6): δ = –4.1 ppm. (**Q**)

^{11}B NMR (129 MHz, C_6D_6): δ = *not detected* (**X**)

^{1}H NMR spectra

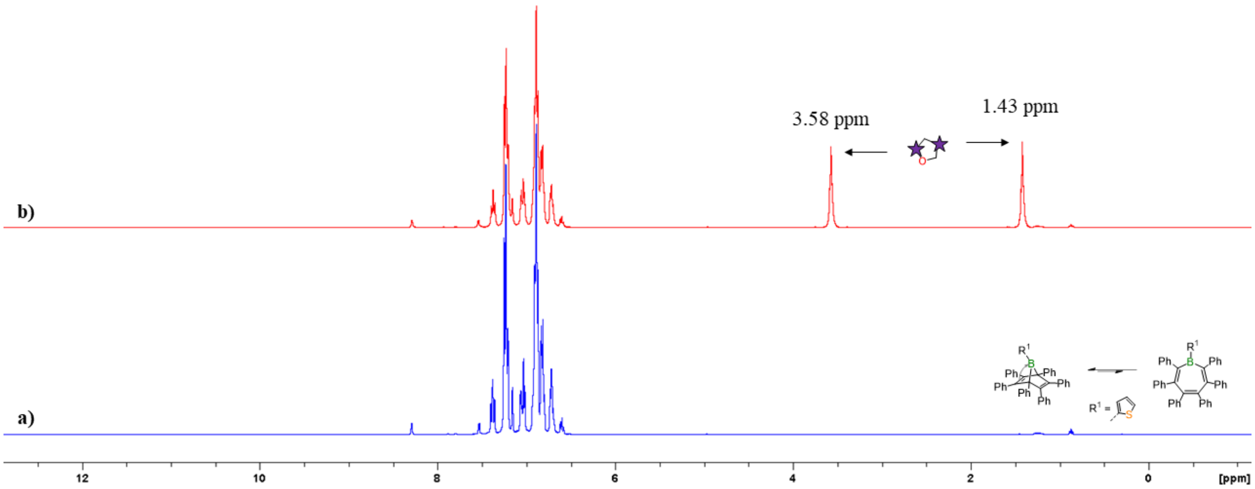

Figure S78. *In-situ* ^{1}H NMR spectra of **2b/3b** in C_6D_6. a) Before THF addition. b) 15 h after addition of THF at room temperature.

^{11}B NMR spectra

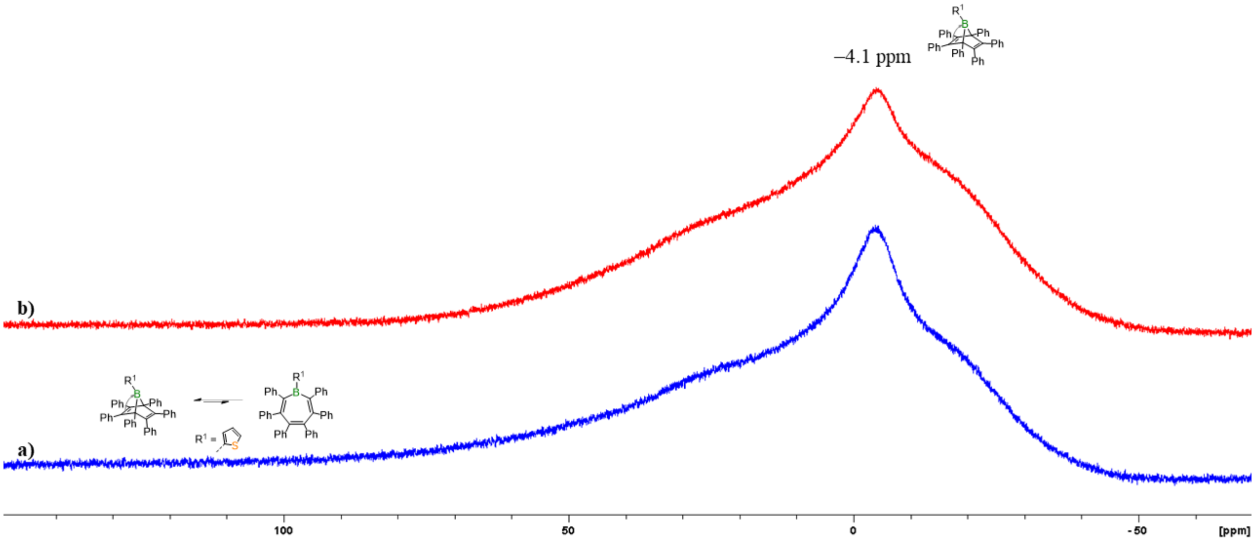

Figure S79. *In-situ* ^{11}B NMR spectra of **2b/3b** in C_6D_6. a) Before THF addition. b) 15 h after THF addition at room temperature.

c) Reaction of 2c/3c with THF

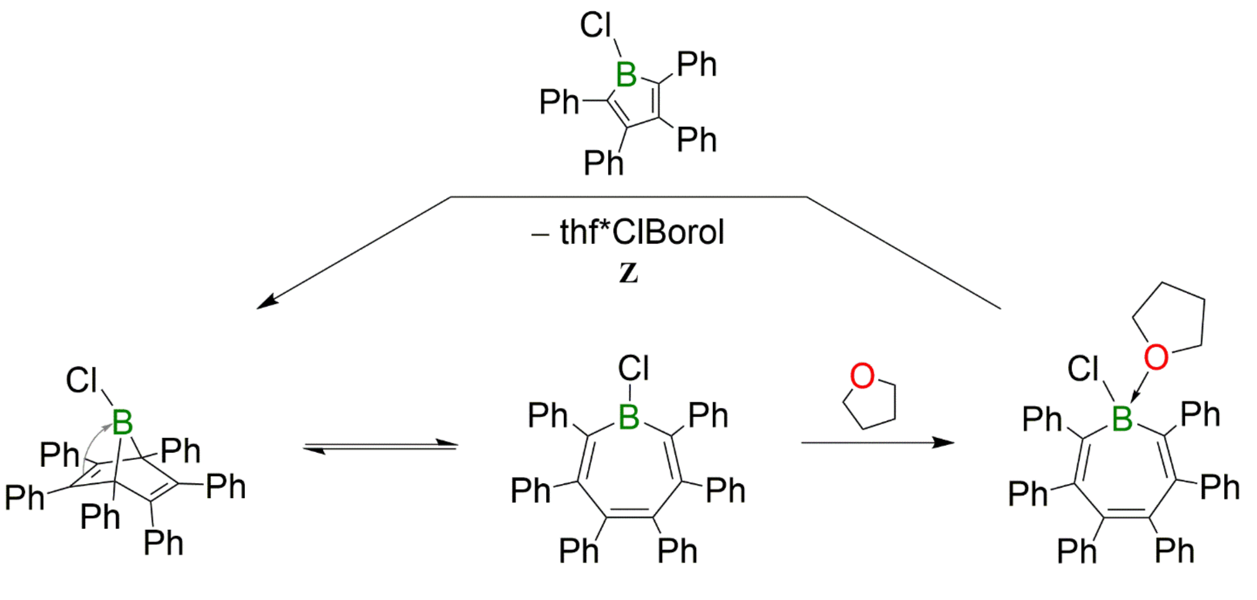

Q **X** **Y**

A suspension of **Q** and **X** (30.0 mg, 0.06 mmol) in chemical equilibrium in 0.6 mL of C_6D_6 was treated with THF (8.3 mg, 0.12 mmol) at room temperature and stirred for 15 h. The formation of borepin **Y** was determined by means of ^{1}H, ^{13}C{^{1}H} and ^{11}B NMR spectroscopy. Subsequently, 1-chloro-2,3,4,5-tetraphenylborole (46.4 mg, 0.12 mmol) was added, which caused the formation of **Q** and the borole adduct **Z**, as evidenced by ^{1}H, ^{13}C{^{1}H} and ^{11}B NMR spectroscopy. Collected NMR data for *in situ* generated **Z** agree well with the published values.[6]

^{11}B NMR (129 MHz, C_6D_6): δ = –2.1 ppm. (**Q**)

^{11}B NMR (129 MHz, C_6D_6): δ = 60.8 ppm. (**X**)

^{11}B NMR (129 MHz, C_6D_6): δ = 11.6 ppm. (**Y**)

^{11}B NMR (129 MHz, C_6D_6): δ = 13.4 ppm. (**Z**)

^{1}H NMR spectra

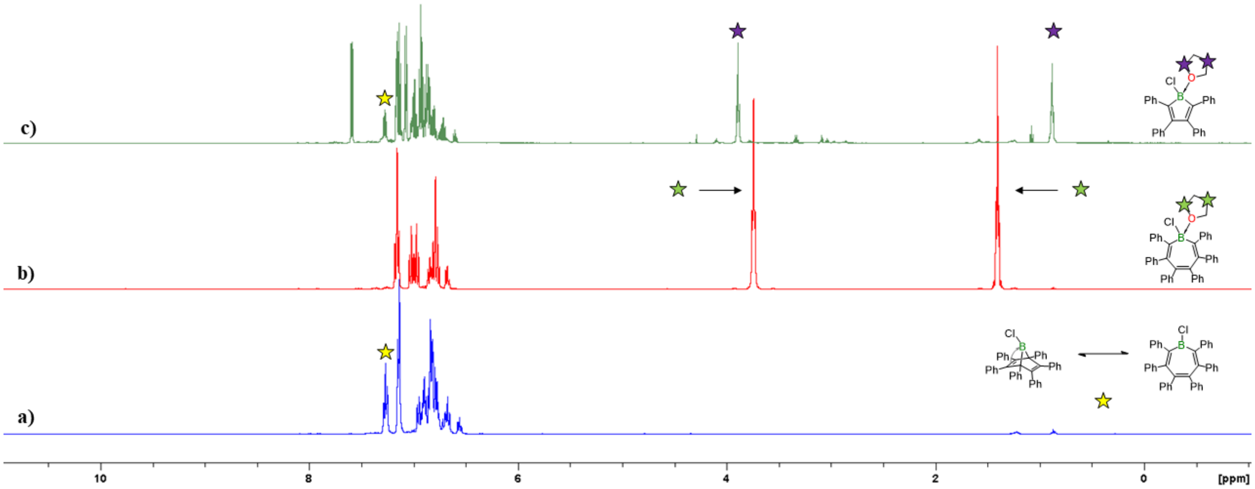

Figure S80. *In-situ* ^{1}H NMR spectra of **2c/3c** in C_6D_6. a) Before THF addition. b) 15 h after addition of THF at room temperature. c) After addition of 1-chloro-2,3,4,5-tetraphenylborole to the borepin THF adduct **Y**, showing regeneration of **Q** and formation of **Z**.

$^{13}C\{^{1}H\}$ NMR spectra

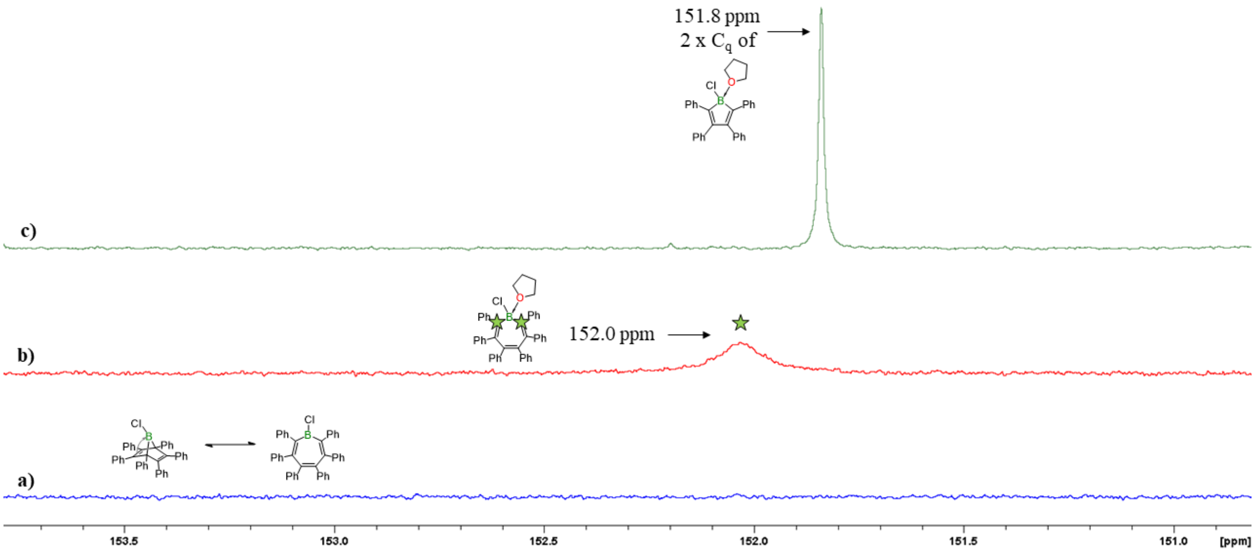

Figure S81. *In-situ* $^{13}C\{^{1}H\}$ NMR spectra of **2c/3c** in C_6D_6 showing an expansion of the aromatic region (part 1/2). a) Before THF addition. b) 15 h after addition of THF at room temperature. c) After addition of 1-chloro-2,3,4,5-tetraphenylborole to the borepin THF adduct **Y**, showing formation of **Z**.

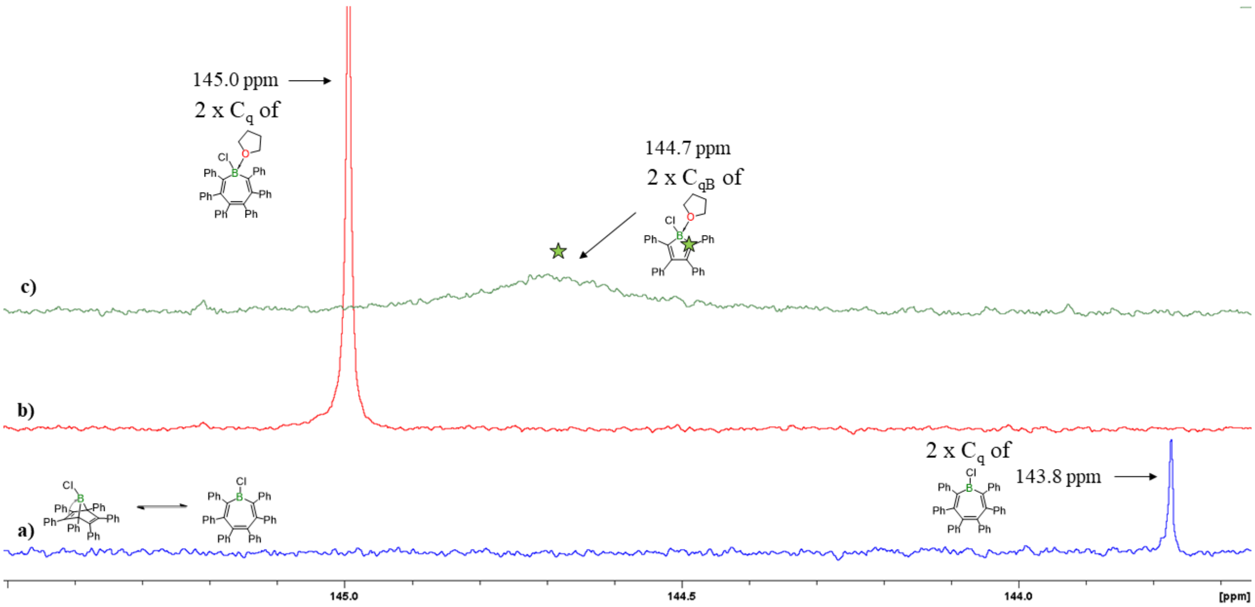

Figure S82. *In-situ* $^{13}C\{^{1}H\}$ NMR spectra of **2c/3c** in C_6D_6 showing an expansion of the aromatic region (part 2/2). a) Before THF addition. b) 15 h after addition of THF at room temperature. c) After addition of 1-chloro-2,3,4,5-tetraphenylborole to the borepin THF adduct **Y**, showing formation of **Z**.

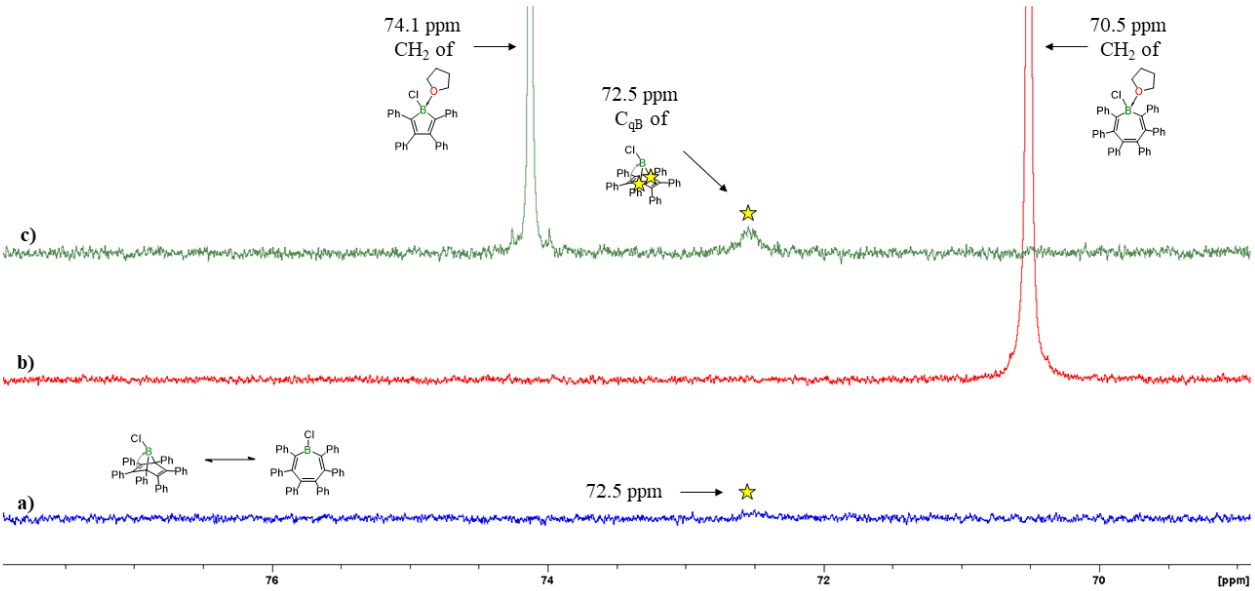

Figure S83. *In-situ* $^{13}C\{^{1}H\}$ NMR spectra of **2c/3c** in C_6D_6 showing an expansion of the aliphatic region (part 1/2). a) Before THF addition. b) 15 h after addition of THF at room temperature. c) After addition of 1-chloro-2,3,4,5-tetraphenylborole to the borepin THF adduct **Y**, showing regeneration of **Q** and formation of **Z.**

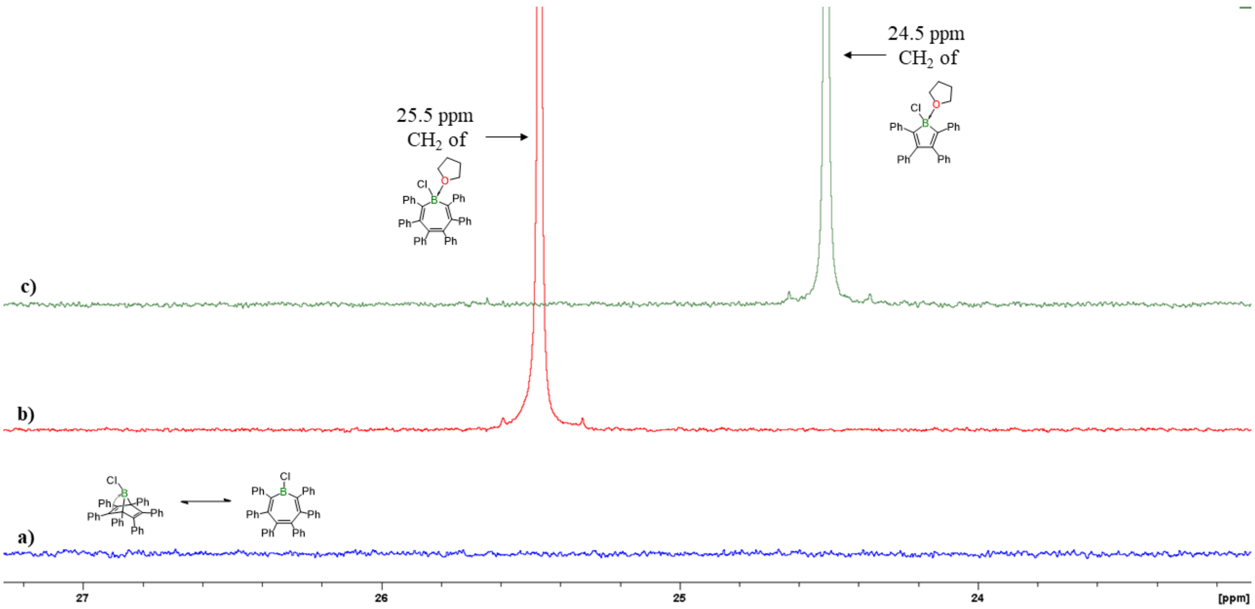

Figure S84. *In-situ* $^{13}C\{^{1}H\}$ NMR spectra in C_6D_6 showing an expansion of the aliphatic region (part 2/2). a) Before THF addition. b) 15 h after addition of THF at room temperature. c) After addition of 1-chloro-2,3,4,5-tetraphenylborole to the borepin THF adduct **Y**, showing formation of **Z**.

^{11}B NMR spectra

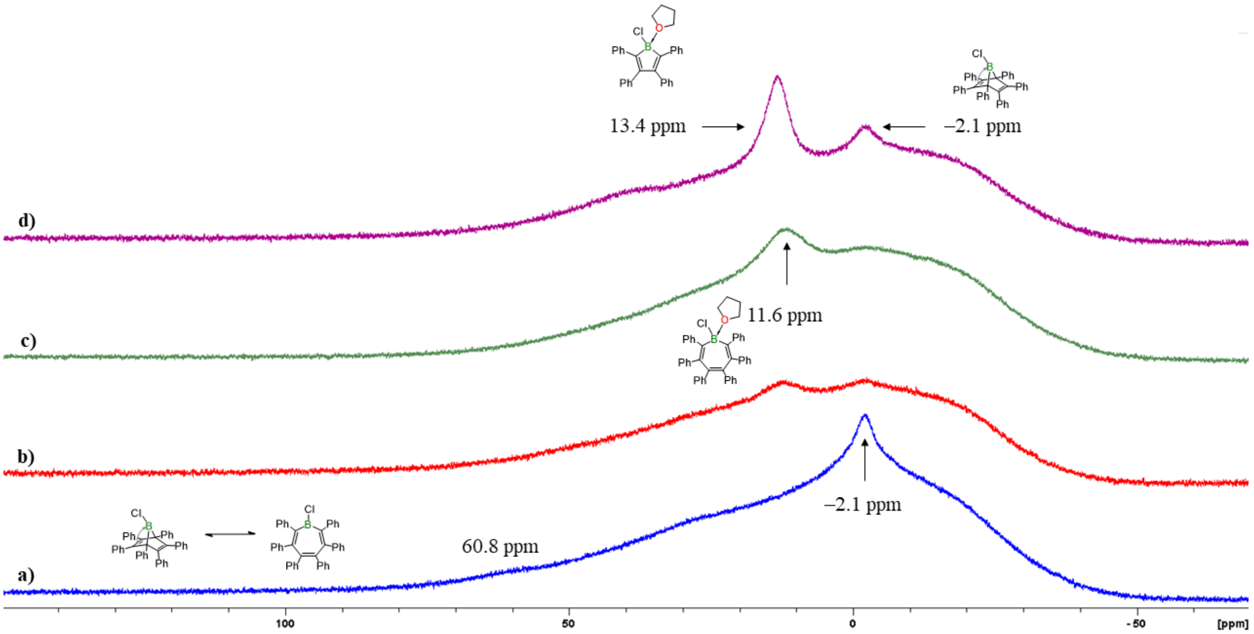

Figure S85. *In-situ* ^{11}B NMR spectra of **2c/3c** in C_6D_6. a) Before THF addition. b) 30 min after addition of THF at room temperature. c) 15 h after addition of THF at room temperature. d) After addition of 1-chloro-2,3,4,5-tetraphenylborole to **Y**, showing formation of **Z** and regeneration of **Q**.

B.27. NMR Studies, Part Three: Irreversible Transformations into the Tricyclic Products

a) Conversion of 2a/3a into 4a

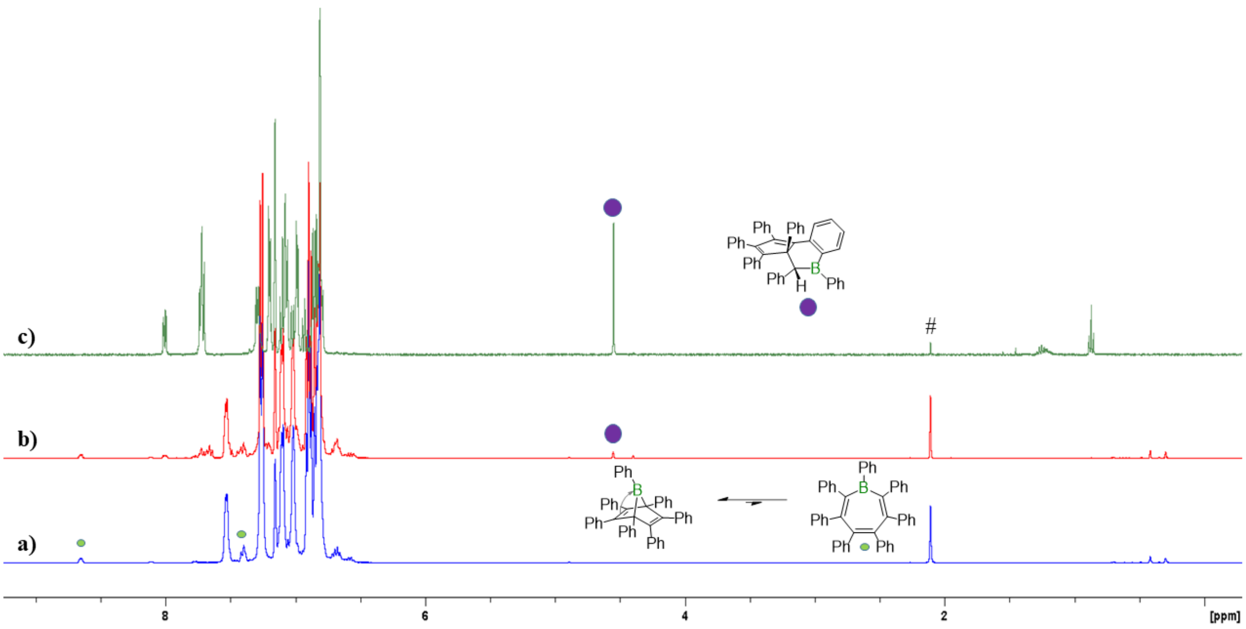

Figure S86. *In-situ* ^{1}H NMR spectra of **2a/3a** in C_6D_6 at various time points. a) At room temperature. The isomer ratio is 16:1 in favor of the boranorbornadiene. b) After heating for 6 d at 80 °C. c) After heating for 60 h in toluene at 140 °C and redissolution in C_6D_6. The signal labeled with a hash symbol (#) is due to residual toluene.

b) Conversion of 2b/3b into 4b

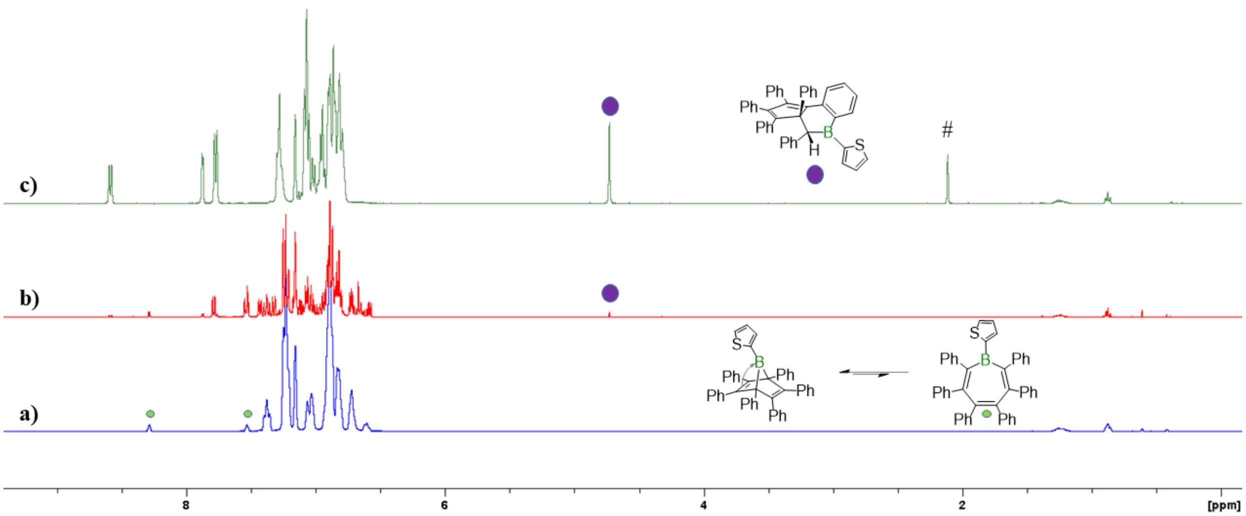

Figure S87. *In-situ* ^{1}H NMR spectra of **2b/3b** in C_6D_6 at various time points. a) At room temperature. **b)** After heating for 6 d at 80 °C. c) After heating in toluene for 60 h at 140 °C and redissolution in C_6D_6. The signal labeled with a hash symbol (#) is due to residual toluene.

c) Conversion of 2c/3c into 4c

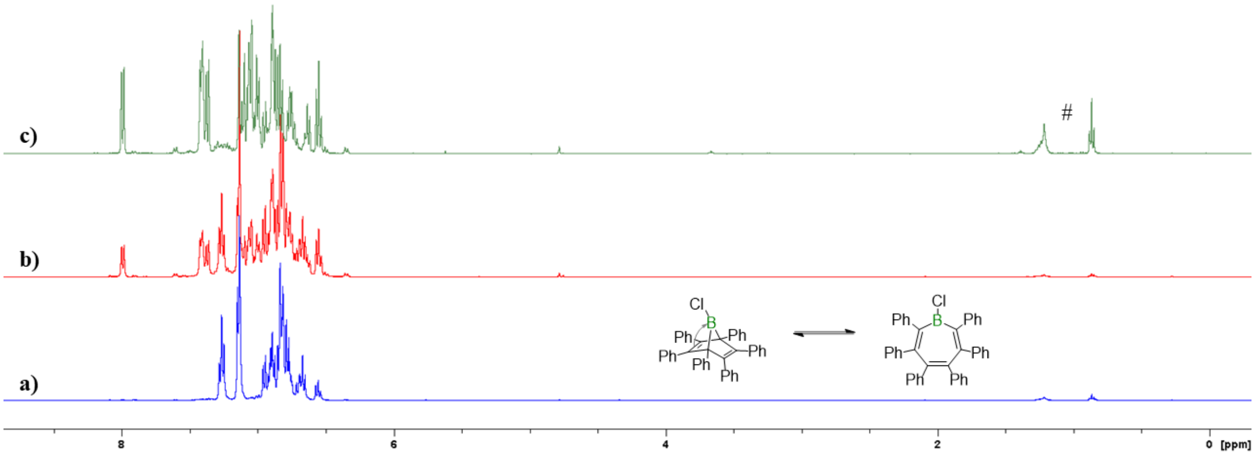

Figure S88. *In-situ* ^{1}H NMR spectra of **2c/3c** in C_6D_6 at various time points. a) At room temperature. b) After heating for 1.5 h at 80 °C in C_6D_6. c) After heating for 15 h at 80 °C in C_6D_6, extraction with hexane and redissolution in C_6D_6. The signals labeled with a hash symbol (#) are due to residual hexane.

C. Crystal Structure Determination

The crystal data of **2b** were collected on a BRUKER D8 QUEST diffractometer with a CMOS area detector and multi-layer mirror monochromated $Mo_{K\alpha}$ radiation. The structure was solved using the intrinsic phasing method,[7] refined with the SHELXL program,[8] and expanded using Fourier techniques. The thiophene substituent comprising atoms C43-46 and S1 (major component) and C47-50 and S2 (minor component) and associated hydrogen atoms is disordered and was modelled over two positions with an occupancy ratio of 87:13. The minor component was refined isotropically as the data do not support anisotropic refinement. Hydrogen atoms were included in structure factors calculations. All hydrogen atoms were assigned to idealized geometric positions. The displacement parameters of atoms C47-C50 and S2 were constrained to the same value with the EADP keyword. The distances between atoms B1 and C43, and B1 and C47, were restrained during refinement to the same value with the SADI restraint. The 1-2 and 1-3 distances in the ring comprising C47-C50 and S2 were restrained to the same values with the SAME keyword.

Crystal data for **2b**: $C_{46}H_{33}BS$, M_r = 628.59, colorless block, 0.2×0.15×0.1 mm^3, monoclinic space group $P2_1/n$, a = 16.068(3) Å, b = 11.246(3) Å, c = 19.010(4) Å, β = 104.907(11)°, V = 3319.5(12) Å^3, Z = 4, ρ_{calcd} = 1.258 g·cm^{-3}, μ = 0.131 mm^{-1}, F(000) = 1320, T = 100(2) K, R_1 = 0.0404, wR^2 = 0.0872, 6530 independent reflections [2θ≤52.042°] and 450 parameters.

Crystallographic data have been deposited with the Cambridge Crystallographic Data Center as supplementary publication no. CCDC 2050168. These data can be obtained free of charge from The Cambridge Crystallographic Data Centre *via* www.ccdc.cam.ac.uk/data_request/cif.

The crystal data of **2c** were collected on a BRUKER X8-APEX II diffractometer with a CCD area detector and multi-layer mirror monochromated $Mo_{K\alpha}$ radiation. The structure was solved using the intrinsic phasing method,[7] refined with the SHELXL program,[8] and expanded using Fourier techniques. All non-hydrogen atoms were refined anisotropically. Hydrogen atoms were included

in structure factors calculations. All hydrogen atoms were assigned to idealized geometric positions.

Crystal data for **2c**: $C_{42}H_{30}BCl$, M_r = 580.92, colorless block, 0.257×0.169×0.13 mm^3, triclinic space group $P\bar{1}$, a = 11.485(3) Å, b = 12.252(4) Å, c = 13.346(3) Å, α = 63.496(8)°, β = 66.018(19)°, γ = 81.739(10)°, V = 1533.8(7) Å^3, Z = 2, ρ_{calcd} = 1.258 g·cm^{-3}, μ = 0.155 mm^{-1}, F(000) = 608, T = 296(2) K, R_1 = 0.0542, wR^2 = 0.0949, 6275 independent reflections [2θ≤52.736°] and 397 parameters.

Crystallographic data have been deposited with the Cambridge Crystallographic Data Center as supplementary publication no. CCDC 2050169. These data can be obtained free of charge from The Cambridge Crystallographic Data Centre *via* www.ccdc.cam.ac.uk/data_request/cif.

The crystal data of **3b(*I*Me)** were collected on a RIGAKU OD XTALAB SYNERGY-S diffractometer with a HPAD area detector and multi-layer mirror monochromated CuK_α radiation. The structure was solved using the intrinsic phasing method,[7] refined with the SHELXL program[8] and expanded using Fourier techniques. All non-hydrogen atoms were refined anisotropically. Hydrogen atoms were included in the structure factors calculations. All hydrogen atoms were assigned to idealized geometric positions.

The solvent molecule (benzene) has shown a small disorder. The residue was divided into two parts. Their occupation was fixed to 50:50, and their atomic displacement parameters were restrained with the similarity restrain SIMU.

Crystal data for **3b(*I*Me)**: $C_{57}H_{47}BN_2S$, M_r = 802.83, clear colorless plate, 0.556×0.192×0.034 mm^3, space group $Pna2_1$, a = 25.8935(3) Å, b = 17.6320(2) Å, c = 9.63920(10) Å, α = 90°, β = 90°, γ = 90°, V = 4400.82(8) Å^3, Z = 4, ρ_{calcd} = 1.212 g·cm^{-3}, μ = 0.956 mm^{-1}, F(000) = 1696, T = 100.00(10) K, R_1 = 0.0529, wR^2 = 0.1432, Flack parameter = 0.028(17), 8220 independent reflections [2θ≤154.966°] and 523 parameters.

Crystallographic data have been deposited with the Cambridge Crystallographic Data Center as supplementary publication no. CCDC 2050170. These data can be obtained free of charge from The Cambridge Crystallographic Data Centre via www.ccdc.cam.ac.uk/data_request/cif.

The crystal data of **3c(*I*Me)** were collected on a BRUKER X8-APEX II diffractometer with a CCD area detector and multi-layer mirror monochromated $Mo_{K\alpha}$ radiation. The structure was solved using the intrinsic phasing method,[7] refined with the SHELXL program,[8] and expanded using Fourier techniques. All non-hydrogen atoms were refined anisotropically. Hydrogen atoms were included in the structure factors calculations. All hydrogen atoms were assigned to idealized geometric positions.

Crystal data for **3c(*I*Me)**: $C_{47}H_{38}BClN_2$, $M_r = 677.05$, colorless plate, 0.05×0.05×0.01 mm^3, monoclinic space group $P2_1/n$, $a = 11.5719(5)$ Å, $b = 18.7805(8)$ Å, $c = 16.7707(11)$ Å, $\beta = 100.988(4)°$, $V = 3577.9(3)$ Å^3, $Z = 4$, $\rho_{calcd} = 1.257$ g·cm^{-3}, $\mu = 0.144$ mm^{-1}, $F(000) = 1424$, $T = 296(2)$ K, $R_1 = 0.1147$, $wR^2 = 0.1393$, 6987 independent reflections [$2\theta \leq 52.038°$] and 462 parameters.

Crystallographic data have been deposited with the Cambridge Crystallographic Data Center as supplementary publication no. CCDC 2050171. These data can be obtained free of charge from The Cambridge Crystallographic Data Centre *via* www.ccdc.cam.ac.uk/data_request/cif

The crystal data of **3c(thf)** were collected on a BRUKER D8 QUEST diffractometer with a CMOS area detector and multi-layer mirror monochromated $Mo_{K\alpha}$ radiation. The structure was solved using the intrinsic phasing method,[7] refined with the SHELXL program,[8] and expanded using Fourier techniques. All non-hydrogen atoms were refined anisotropically. Hydrogen atoms were included in structure factors calculations. All hydrogen atoms were assigned to idealized geometric positions. The benzene and THF moieties showed disorder. The atomic displacement parameters (ADPs) of overlapping atoms from different PARTs (O1, C1, C2, C3, C4 of RESIs 8/18 and C1,

C2, C3, C4, C5 and C6 of RESI 9) were restrained using similarity restraint (SIMU) and rigid body restraint (RIGU). The distances between atoms of the disordered THF moieties of residue 8/18 were restrained during refinement to the same value (SAME). The geometry of the benzene molecule was constrained to an idealized hexagon. Its ADPs were additionally restrained using the isotropic restraint ISOR. Reflections [0 3 3], [0 2 2], [-1 2 1], [0 39 3], and [-2 39 3] were omitted in the refinement because of *I*(obs) << *I*(calc) (PLAT919).

Crystal data for **3c(thf)**: $C_{49}H_{41}BClO$, $M_r = 692.08$, colorless block, 0.357×0.165×0.076 mm^3, tetragonal space group $I\bar{1}2d$, $a = 31.872(8)$ Å, $b = 31.872(8)$ Å, $c = 14.766(6)$ Å, $\alpha = 90°$, $\beta = 90°$, $\gamma = 90°$, $V = 15000(10)$ Å^3, $Z = 16$, $\rho_{calcd} = 1.226$ g·cm^{-3}, $\mu = 0.139$ mm^{-1}, $F(000) = 5840$, $T = 100(2)$ K, $R_1 = 0.0537$, $wR^2 = 0.1109$, 7942 independent reflections [2θ≤53.572°] and 542 parameters.

Crystallographic data have been deposited with the Cambridge Crystallographic Data Center as supplementary publication no. CCDC 2050172. These data can be obtained free of charge from The Cambridge Crystallographic Data Centre *via* www.ccdc.cam.ac.uk/data_request/cif.

The crystal data of **4a** were collected on a BRUKER D8 QUEST diffractometer with a CMOS area detector and multi-layer mirror monochromated $Mo_{K\alpha}$ radiation. The structure was solved using the intrinsic phasing method,[7] refined with the SHELXL program,[8] and expanded using Fourier techniques. All non-hydrogen atoms were refined anisotropically. Hydrogen atoms were included in the structure factors calculations. All hydrogen atoms were assigned to idealized geometric positions.

Crystal data for **4a**: $C_{48}H_{35}BN_0$, $M_r = 622.57$, yellow block, 0.84×0.525×0.384 mm^3, triclinic space group $P\bar{1}$, $a = 9.842(4)$ Å, $b = 10.506(4)$ Å, $c = 16.706(5)$ Å, $\alpha = 78.254(18)°$, $\beta = 85.405(15)°$, $\gamma = 88.994(14)°$, $V = 1685.9(11)$ Å^3, $Z = 2$, $\rho_{calcd} = 1.226$ g·cm^{-3}, $\mu = 0.069$ mm^{-}

^{1}, $F(000) = 656$, $T = 100(2)$ K, $R_1 = 0.0678$, $wR^2 = 0.1618$, 6609 independent reflections [2θ≤52.042°] and 442 parameters.

Crystallographic data have been deposited with the Cambridge Crystallographic Data Center as supplementary publication no. CCDC 2050173. These data can be obtained free of charge from The Cambridge Crystallographic Data Centre *via* www.ccdc.cam.ac.uk/data_request/cif.

The crystal data of **4b** were collected on a BRUKER D8 QUEST diffractometer with a CMOS area detector and multi-layer mirror monochromated $Mo_{K\alpha}$ radiation. The structure was solved using the intrinsic phasing method,[7] refined with the SHELXL program,[8] and expanded using Fourier techniques. All non-hydrogen atoms were refined anisotropically. Hydrogen atoms were included in the structure factors calculations. All hydrogen atoms were assigned to idealized geometric positions.

Crystal data for **4b**: $C_{52}H_{39}BN_{0}S$, $M_r = 706.70$, yellow plate, 0.533×0.194×0.041 mm^3, triclinic space group $P\bar{1}$, $a = 9.7661(10)$ Å, $b = 10.5267(11)$ Å, $c = 19.744(2)$ Å, $\alpha = 92.467(3)°$, $\beta = 100.508(3)°$, $\gamma = 106.354(3)°$, $V = 1905.4(3)$ Å^3, $Z = 2$, $\rho_{calcd} = 1.232$ g·cm^{-3}, $\mu = 0.122$ mm^{-1}, $F(000) = 744$, $T = 100(2)$ K, $R_1 = 0.0910$, $wR^2 = 0.1362$, 7473 independent reflections [2θ≤52.044°] and 487 parameters.

Crystallographic data have been deposited with the Cambridge Crystallographic Data Center as supplementary publication no. CCDC 2050174. These data can be obtained free of charge from The Cambridge Crystallographic Data Centre *via* www.ccdc.cam.ac.uk/data_request/cif.

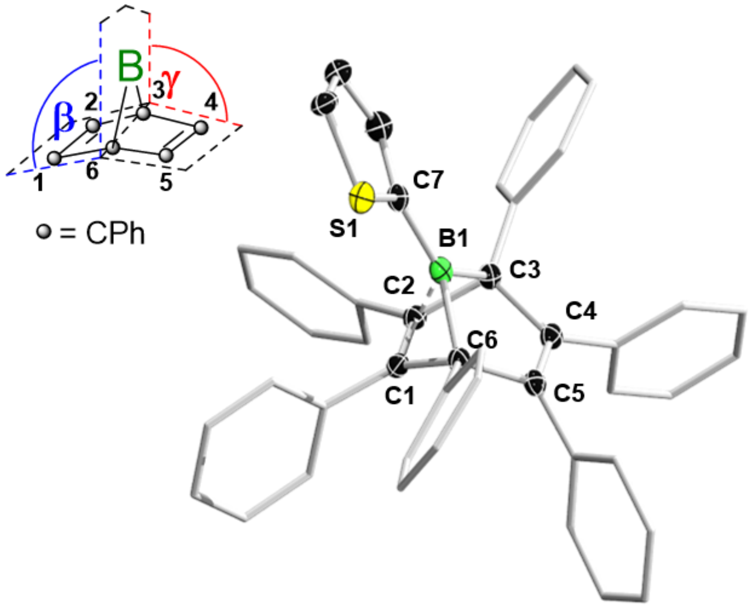

Figure S89. Molecular structure of **2b** as determined by single-crystal X-ray diffraction. Hydrogen atoms are omitted for clarity and thermal ellipsoids are set at 50% probability. Selected bond lengths [Å] and angles [°]: B1–C7 1.561(2), B1–C1 1.843(2), B1–C2 1.855(2), B1–C3 1.638(2), B1–C6 1.626(2), C1–C2 1.389(2), C2–C3 1.519(2), C3–C4 1.520(2), C4–C5 1.335(2), C5–C6 1.525(2), C6–C1 1.520(2); β 84.91, γ 151.80.

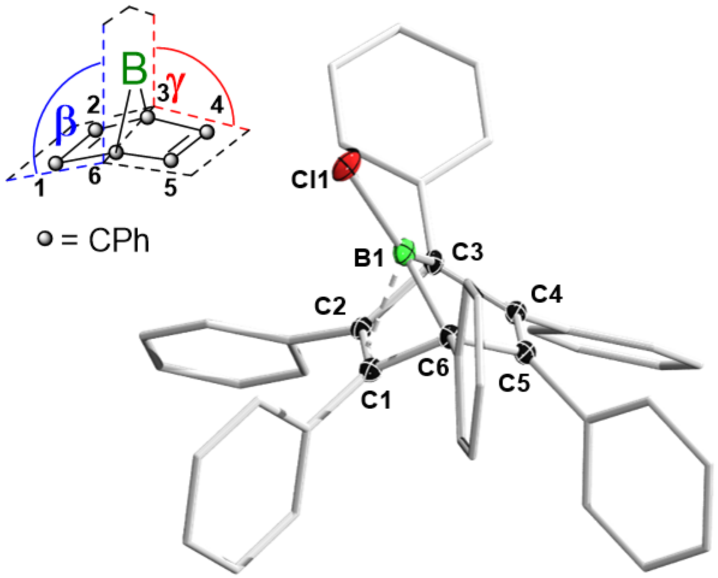

Figure S90. Molecular structure of **2c** as determined by single-crystal X-ray diffraction. Hydrogen atoms are omitted for clarity and thermal ellipsoids are set at 50% probability. Selected bond lengths [Å] and angles [°]: B1–Cl1 1.769(2), B1–C3 1.610(3), B1–C6 1.593(3), C1–C2 1.380(3), C2–C3 1.539(2), C3–C4 1.525(2), C4–C5 1.347(3), C5–C6 1.523(2), C6–C1 1.539(3); *β* 86.7, *γ* 152.4.

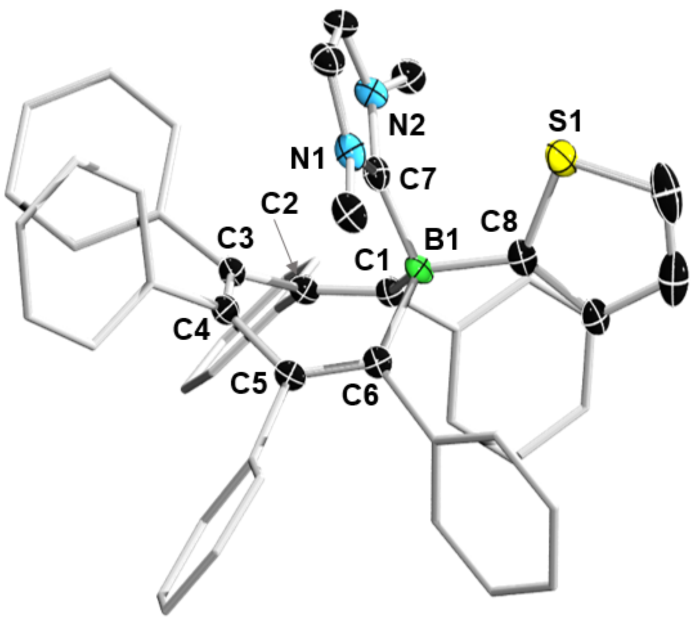

Figure S91. Molecular structure of **3b(*I*Me)** as determined by single-crystal X-ray diffraction. Hydrogen atoms are omitted for clarity and thermal ellipsoids are set at 50% probability. Selected bond lengths [Å] and angles [°]: B1–C8 1.623(5), B1–C1 1.651(4), B1–C6 1.648(4), C1–C2 1.368(4), C2–C3 1.475(4), C3–C4 1.373(4), C4–C5 1.479(4), C5–C6 1.360(4), B1–C7 1.626(5); C1–B1–C6 97.47, C1–B1–C8 115.65, C1–B1–C7 111.39, C6–B1–C8 114.00, C6–B1–C7 113.83.

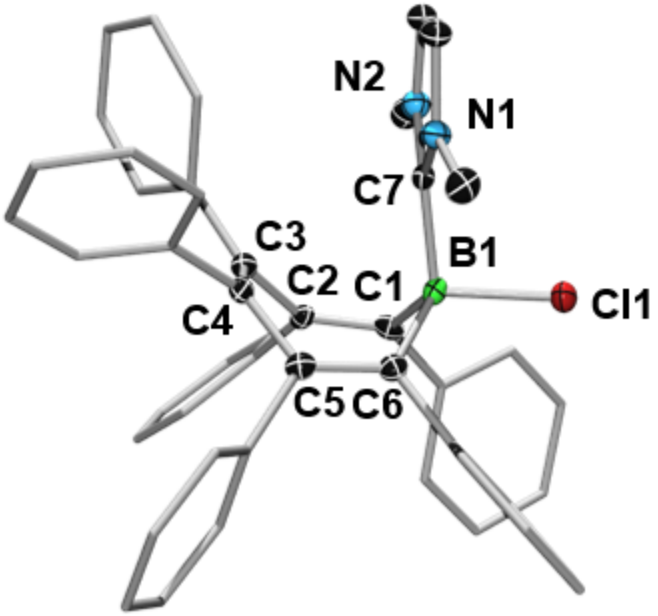

Figure S92. Molecular structure of **3c(*I*Me)** as determined by single-crystal X-ray diffraction. Hydrogen atoms are omitted for clarity and thermal ellipsoids are set at 50% probability. Selected bond lengths [Å] and angles [°]: B1–Cl1 1.917(4), B1–C1 1.633(4), B1–C6 1.642(4), C1–C2 1.355(4), C2–C3 1.507(3), C3–C4 1.358(4), C4–C5 1.480(4), C5–C6 1.360(4), B1–C7 1.631(4); C1–B1–C6 101.83, C1–B1–Cl1 112.95, C1–B1–C7 116.38, C6–B1–Cl1 111.33, C6–B1–C7 116.05.

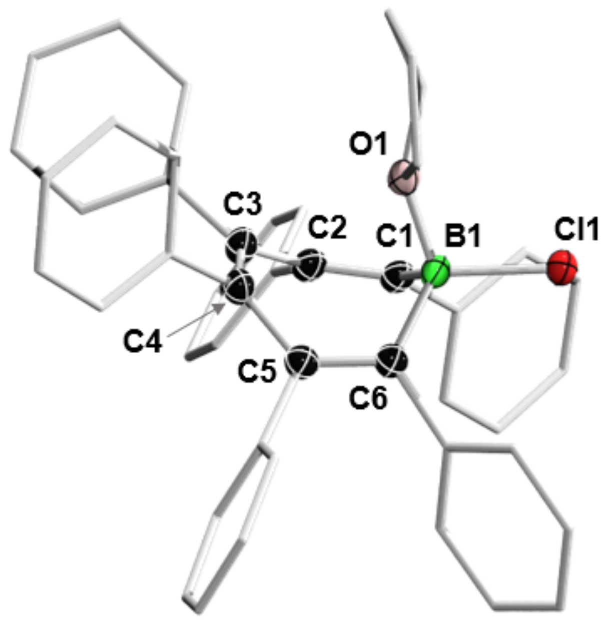

Figure S93. Molecular structure of **3c(thf)** as determined by single-crystal X-ray diffraction. Hydrogen atoms are omitted for clarity and thermal ellipsoids are set at 50% probability. Selected bond lengths [Å] and angles [°]: B1–Cl1 1.881(3), B1–C1 1.601(4), B1–C6 1.599(4), C1–C2 1.353(4), C2–C3 1.475(4), C3–C4 1.366(4), C4–C5 1.485(4), C5–C6 1.354(4), B1–O1 1.55(2); C1–B1–C6 105.4(2), C1–B1–Cl1 114.5(2), C1–B1–O1 109.3(6), C6–B1–Cl1 113.8(2), C6–B1–O1 109.4(6).

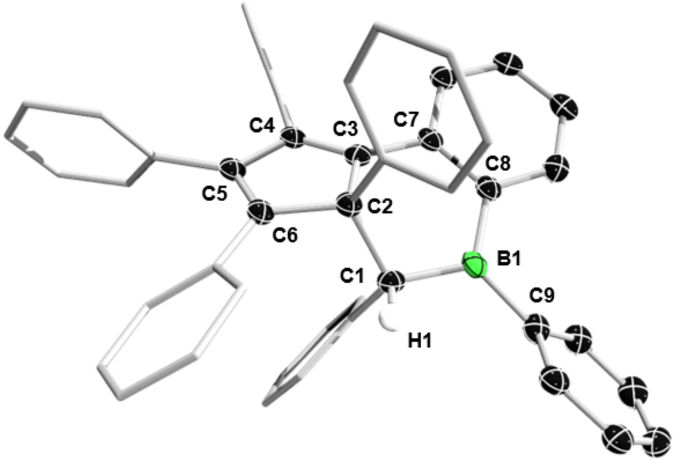

Figure S94. Molecular structure of **4a** as determined by single-crystal X-ray diffraction. Hydrogen atoms are omitted for clarity and thermal ellipsoids are set at 50% probability. Selected bond lengths [Å]: B1−C1 1.590(2), B1−C8 1.564(3), B1−C9 1.570(2), C1−C2 1.564(2), C2−C3 1.518(3), C3−C4 1.357(2), C4−C5 1.476(2), C5−C6 1.359(3), C2−C6 1.527(2), C3−C7 1.463(2), C7−C8 1.424(2).

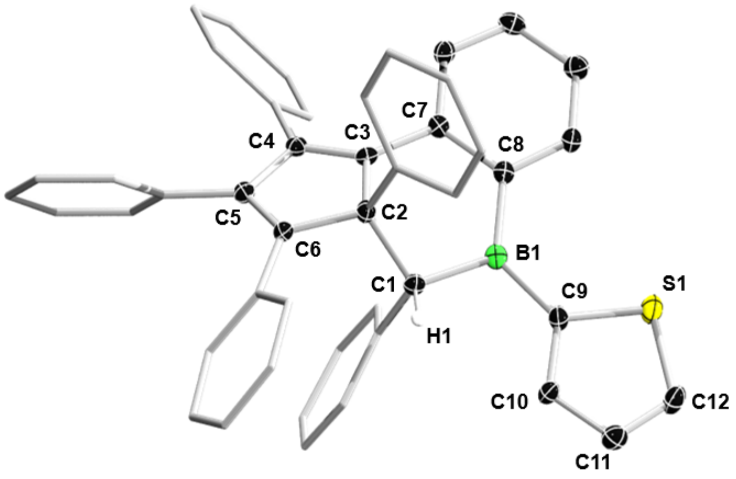

Figure S95. Molecular structure of **4b** as determined by single-crystal X-ray diffraction. Hydrogen atoms are omitted for clarity and thermal ellipsoids are set at 50% probability. Selected bond lengths [Å]: B1−C1 1.602(3), B1−C8 1.567(4), B1−C9 1.548(4), C1−C2 1.567(3), C2−C3 1.512(3), C3−C4 1.357(3), C4−C5 1.492(3), C5−C6 1.355(3), C2−C6 1.526(2), C3−C7 1.464(2), C7−C8 1.428(3), S1−C9 1.736(2), S1−C12 1.692(2), C9−C10 1.405(3), C10−C11 1.407(3), C11−C12 1.357(2).

D. Photophysical Studies

D.1. Methods

All measurements were performed in standard quartz cuvettes (1 cm × 1 cm cross section). UV–visible absorption spectra were recorded using an Agilent 8453 diode array UV-visible spectrophotometer. The emission spectra were recorded using an Edinburgh Instruments FLSP920 spectrometer equipped with a double monochromator for both excitation and emission, operating in right-angle geometry mode. The spectra were fully corrected for the spectral response of the instrument. The solutions used for photophysical measurements had a concentration lower than 10^{-5} M to minimize inner filter effects during fluorescence measurements. The fluorescence quantum yields were measured using a calibrated integrating sphere (inner diameter: 150 mm) from Edinburgh Instruments combined with the FLSP920 spectrometer described above. For solution- and solid-state measurements, the longest-wavelength absorption maximum of the compound in the respective solvent was chosen as the excitation wavelength. Fluorescence lifetimes were recorded using the time-correlated single-photon counting (TCSPC) method in combination with the FLSP920 spectrometer described above. Solutions were excited with a picosecond pulsed diode laser at an emission maximum of 418.6 nm or 376 nm. The full width at half maximum (FWHM) of the laser pulses were ca. 50–100 ps, whereas the instrument response function (IRF) had a FWHM of ca. 1.0 ns, which was measured from the scatter of a Ludox solution at the excitation wavelength. Decays were recorded to at least 10000 counts in the peak channel with a record length of at least 1000 channels. The band pass of the monochromator was adjusted to give a signal count rate of <50 kHz. Iterative reconvolution of the IRF with one decay function and non-linear least-squares analysis were used to analyze the data. The quality of the fit was judged by the calculated value of the reduced χ^2 and visual inspection of the weighted residuals.

D.2. UV-vis Spectra of 4a and 4b

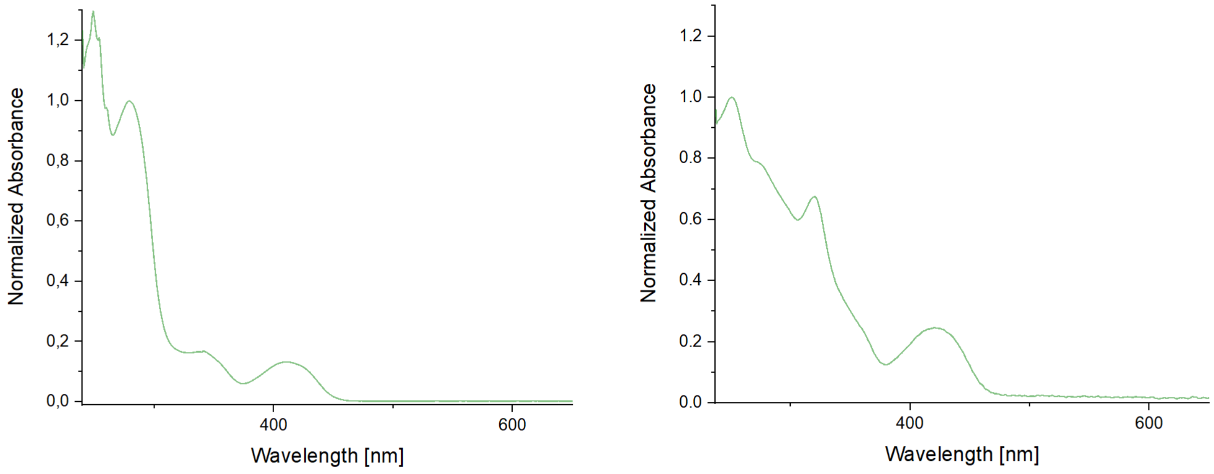

Figure S96. Qualitative UV−vis spectra of **4a** (left) and **4b** (right) in Et_2O. **4a**: λ_{max} = 249 nm, 279 nm, 341 nm, 410 nm; **4b**: λ_{max} = 251 nm, 273 nm, 321 nm, 422 nm.

D.3. Combined Absorption/Excitation and Emission Spectra

4a in hexane:

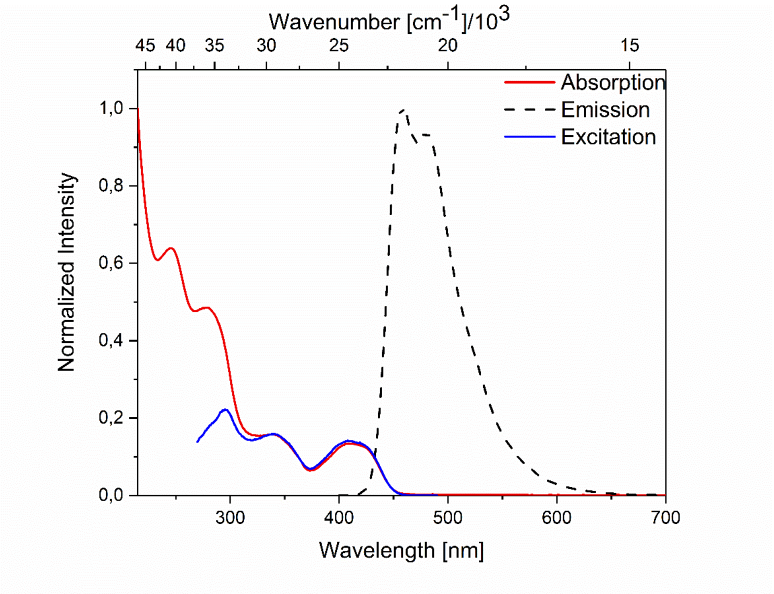

4a in toluene:

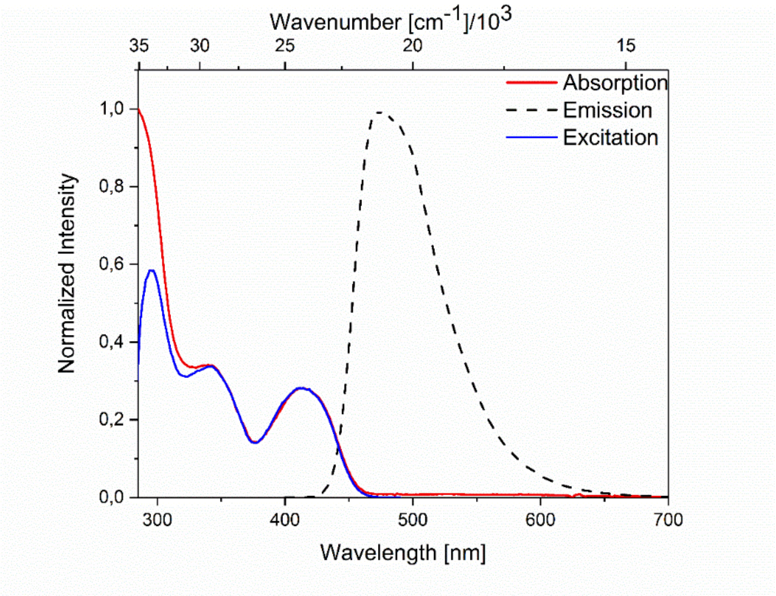

4b in hexane:

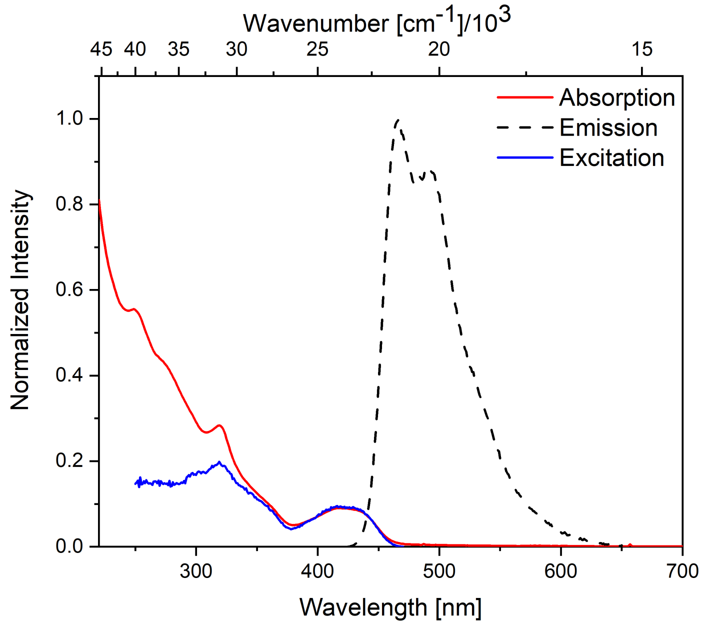

4b in toluene:

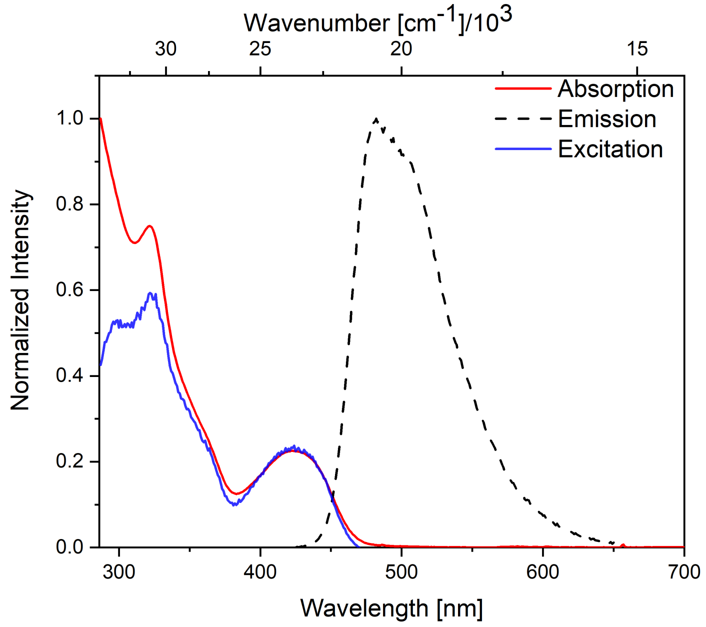

The extinction coefficients of **4a** and **4b** were calculated from three independently prepared samples in toluene.

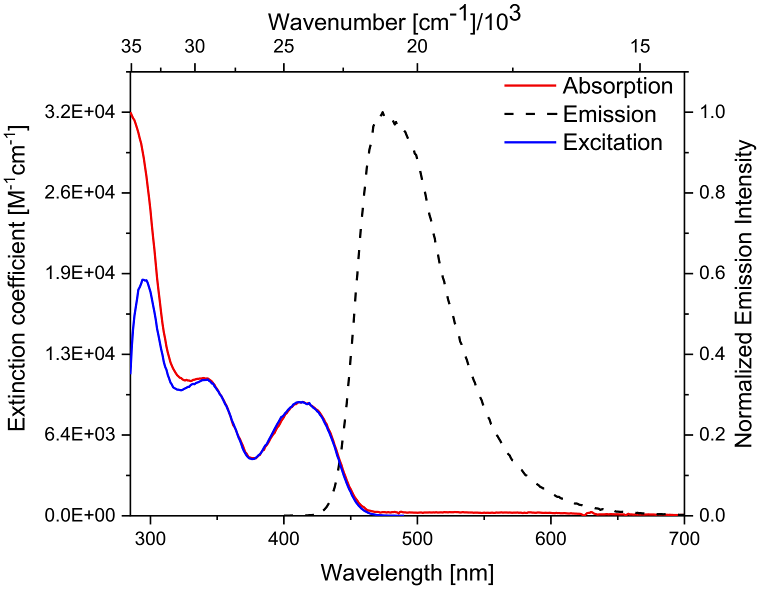

The molar extinction coefficient (ε) of **4a** at 414 nm is 9000 $M^{-1}cm^{-1}$.

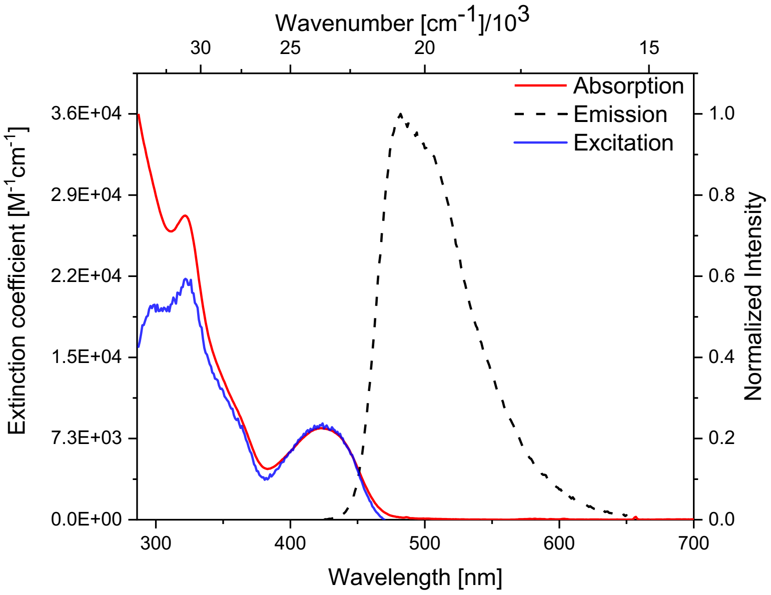

The molar extinction coefficient (ε) of **4b** at 423 nm is 8200 $M^{-1}cm^{-1}$.

D.4. DFT Calculations

DFT and TD-DFT calculations were carried out with the Gaussian 09 (9.E.01)[9] program package and performed on a parallel cluster system. GaussView (6.0.16), Avogadro (1.2.0)[10] and multiwfn[11] were used to visualize the results, to measure calculated structural parameters, and to plot orbital surfaces (isovalue: ± 0.030 [e a_0^{-3}]$^{1/2}$). The ground-state geometries were optimized using the B3LYP functional[12] in combination with the 6-31G+(d) basis set.[13] The ultrafine integration grid and no symmetry constraints were used. Frequency calculations on the optimized structures were performed to ensure that no negative (imaginary) frequencies existed. Based on these optimized structures, the lowest-energy vertical transitions (gas-phase) were calculated (singlets, 25 states) by TD-DFT, using B3LYP in combination with the 6-31G+(d,p) basis set.[13]

a) TD-DFT results

Compound 4a

<table>
<tr><th>Calculated absorption spectra</th><th>Orbital</th><th>Energy [eV]</th><th>Symmetry</th></tr>
<tr><td rowspan="10">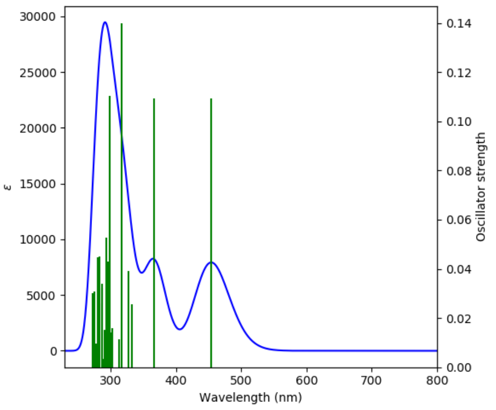

TD-DFT B3LYP/6-31G+(d), gas phase</td><td>L+4</td><td>-0.54</td><td>A</td></tr>
<tr><td>L+3</td><td>-0.59</td><td>A</td></tr>
<tr><td>L+2</td><td>-0.84</td><td>A</td></tr>
<tr><td>L+1</td><td>-1.47</td><td>A</td></tr>
<tr><td>LUMO</td><td>-2.06</td><td>A</td></tr>
<tr><td>HOMO</td><td>-5.35</td><td>A</td></tr>
<tr><td>H-1</td><td>-6.37</td><td>A</td></tr>
<tr><td>H-2</td><td>-6.49</td><td>A</td></tr>
<tr><td>H-3</td><td>-6.66</td><td>A</td></tr>
<tr><td>H-4</td><td>-6.72</td><td>A</td></tr>
</table>

Orbitals relevant to the $S_1 \leftarrow S_0$ transition	other relevant orbitals	
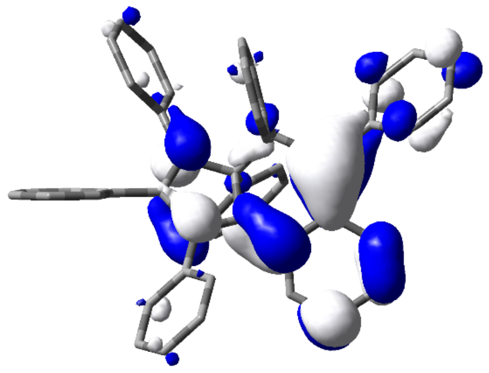<br>**LUMO**<br>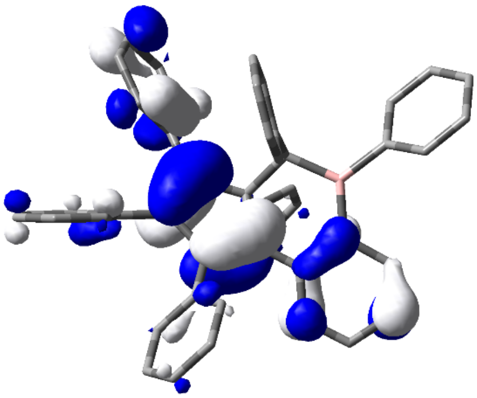<br>**HOMO**	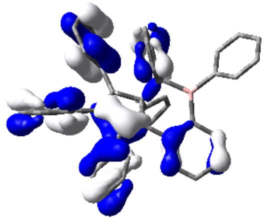<br>**HOMO-1**<br>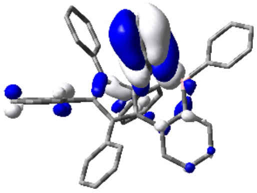<br>**HOMO-2**<br>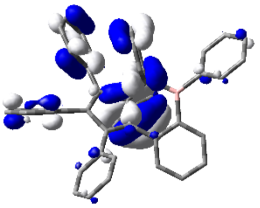<br>**HOMO-3**	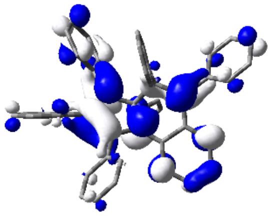<br>**LUMO+1**<br>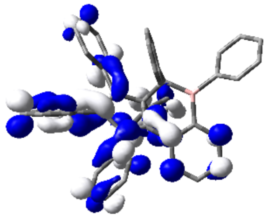<br>**LUMO+2**<br>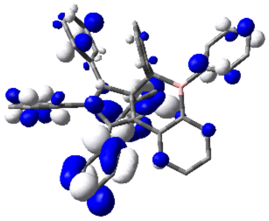<br>**LUMO+3**

Table S1: Lowest-energy singlet electronic transition of **4a** (TD-DFT CAM-B3LYP/6-31G+(d,p), gas phase).

State	E [eV]	λ [nm]	*f*	Symmetry	Major contributions	Λ
**1**	2.73	454.32	0.1094	A	HOMO->LUMO (98%)	0.61
**2**	3.38	367.02	0.1094	A	HOMO->L+1 (97%)	0.77
**3**	3.73	332.29	0.0257	A	H-2->LUMO (43%), H-1->LUMO (50%)	0.49
**4**	3.78	327.78	0.0391	A	H-2->LUMO (53%), H-1->LUMO (37%)	0.48
**5**	3.92	316.50	0.14	A	H-1->LUMO (10%), HOMO->L+2 (83%)	0.65
**6**	3.97	312.65	0.0113	A	H-3->LUMO (92%)	0.42
**7**	4.09	303.31	0.0159	A	H-4->LUMO (68%)	0.50
**8**	4.13	300.55	0.0143	A	HOMO->L+3 (75%)	0.51

**9**	4.14	299.43	0.1105	A	H-6->LUMO (16%), H-5->LUMO (56%), HOMO->L+4 (12%)	0.52
**10**	4.18	296.32	0.0429	A	H-6->LUMO (48%), HOMO->L+4 (22%)	0.45
**11**	4.23	293.21	0.0528	A	H-5->LUMO (24%), HOMO->L+4 (43%)	0.50
**12**	4.26	291.38	0.0035	A	H-9->LUMO (29%), H-8->LUMO (46%)	0.41
**13**	4.26	290.85	0.0152	A	H-9->LUMO (20%), H-8->LUMO (16%), H-7->LUMO (32%)	0.41
**14**	4.28	289.46	0.0035	A	H-9->LUMO (42%), H-7->LUMO (28%)	0.38
**15**	4.31	287.63	0.0321	A	H-8->LUMO (17%), H-1->L+1 (10%), HOMO->L+5 (53%)	0.42
**16**	4.32	286.75	0.0341	A	H-2->L+1 (24%), H-1->L+1 (48%), HOMO->L+5 (10%)	0.54
**17**	4.37	283.75	0.0259	A	H-1->L+1 (10%), HOMO->L+6 (43%), HOMO->L+7 (20%)	0.47
**18**	4.38	282.83	0.045	A	H-2->L+1 (50%), HOMO->L+6 (19%)	0.45
**19**	4.40	282.05	0.0206	A	H-10->LUMO (34%), HOMO->L+6 (15%), HOMO->L+7 (18%)	0.44
**20**	4.43	280.09	0.0447	A	H-10->LUMO (21%), HOMO->L+7 (34%), HOMO->L+8 (19%)	0.44
**21**	4.46	278.05	0.0095	A	H-11->LUMO (28%), HOMO->L+8 (25%), HOMO->L+9 (13%)	0.45
**22**	4.50	275.58	0.0309	A	H-11->LUMO (13%), H-10->LUMO (13%), HOMO->L+8 (22%), HOMO->L+9 (18%), HOMO->L+10 (13%)	0.46
**23**	4.51	274.62	0.0039	A	H-12->LUMO (15%), H-11->LUMO (37%), HOMO->L+10 (27%)	0.51
**24**	4.53	273.84	0.0042	A	HOMO->L+8 (15%), HOMO->L+9 (46%)	0.44
**25**	4.55	272.24	0.03	A	H-3->L+1 (75%)	0.48

Compound 4b

Calculated absorption spectra

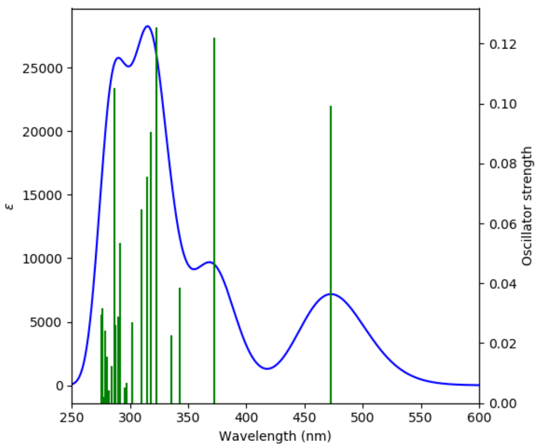

TD-DFT B3LYP/6-31G+(d), gas phase

Orbital	Energy [eV]	Symmetry
L+4	-0.54	A
L+3	-0.63	A
L+2	-0.83	A
L+1	-1.53	A
LUMO	-2.17	A
HOMO	-5.34	A
H-1	-6.36	A
H-2	-6.47	A
H-3	-6.57	A
H-4	-6.67	A

Orbitals relevant to the $S_1 \leftarrow S_0$ transition

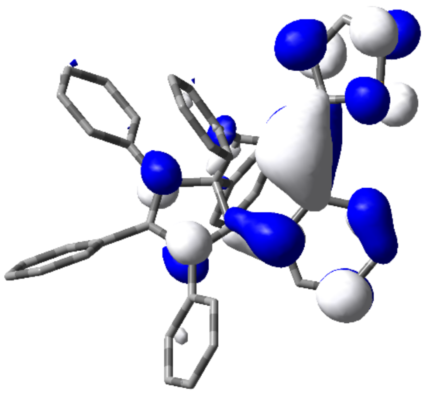

LUMO

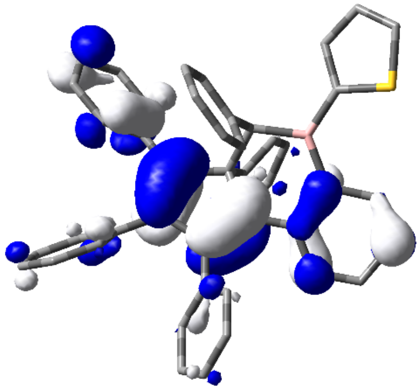

HOMO

other relevant orbitals

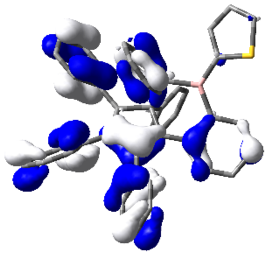

HOMO-1

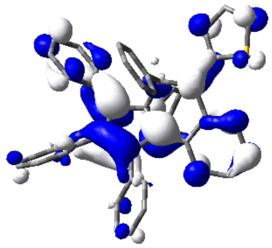

LUMO+1

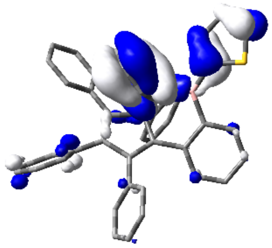

HOMO-2

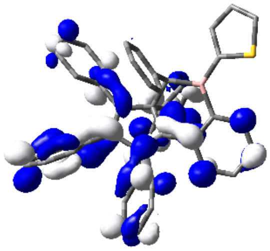

LUMO+2

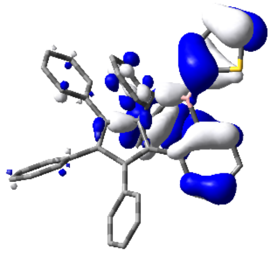

HOMO-3

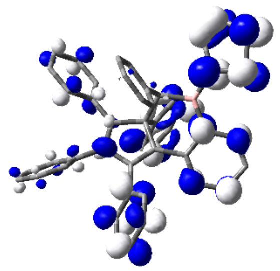

LUMO+3

Table S2: Lowest-energy singlet electronic transition of **4b** (TD-DFT B3LYP/6-31G+(d), gas phase).

**State**	**E [eV]**	**λ [nm]**	***f***	**Symmetry**	**Major contributions**	**Λ**
**1**	2.62	472.77	0.0991	A	HOMO->LUMO (99%)	0.54
**2**	3.33	372.37	0.1219	A	HOMO->L+1 (97%)	0.79
**3**	3.62	342.48	0.0385	A	H-2->LUMO (47%), H-1->LUMO (42%)	0.50
**4**	3.69	335.73	0.0226	A	H-2->LUMO (41%), H-1->LUMO (51%)	0.49
**5**	3.84	323.08	0.1255	A	H-3->LUMO (74%)	0.62
**6**	3.90	317.97	0.0905	A	H-4->LUMO (28%), HOMO->L+2 (62%)	0.60
**7**	3.94	314.87	0.0757	A	H-4->LUMO (50%), H-3->LUMO (11%), HOMO->L+2 (24%)	0.54
**8**	4.00	309.60	0.0648	A	H-6->LUMO (22%), H-5->LUMO (67%)	0.37
**9**	4.10	302.23	0.0247	A	H-8->LUMO (10%), HOMO->L+3 (61%)	0.52
**10**	4.11	301.67	0.0271	A	H-6->LUMO (50%), H-5->LUMO (12%), HOMO->L+4 (14%)	0.42
**11**	4.17	297.30	0.0069	A	H-8->LUMO (12%), H-7->LUMO (48%), HOMO->L+4 (16%)	0.41
**12**	4.18	296.71	0.004	A	H-8->LUMO (53%), H-6->LUMO (11%), HOMO->L+4 (17%)	0.46
**13**	4.20	295.26	0.0053	A	H-7->LUMO (31%), HOMO->L+3 (14%), HOMO->L+4 (36%)	0.45
**14**	4.26	291.25	0.0536	A	H-2->L+1 (17%), H-1->L+1 (57%)	0.61
**15**	4.28	290.01	0.015	A	H-10->LUMO (40%), H-9->LUMO (46%)	0.50
**16**	4.28	289.75	0.029	A	H-10->LUMO (13%), HOMO->L+5 (74%)	0.49
**17**	4.32	287.33	0.0262	A	H-10->LUMO (13%), H-9->LUMO (18%), H-2->L+1 (40%)	0.48
**18**	4.33	286.30	0.1051	A	H-10->LUMO (14%), H-9->LUMO (13%), H-2->L+1 (28%), H-1->L+1 (17%)	0.51
**19**	4.36	284.26	0.0124	A	HOMO->L+6 (85%)	0.45
**20**	4.40	281.94	0.0043	A	H-11->LUMO (58%), H-9->LUMO (10%)	0.40
**21**	4.43	279.85	0.0155	A	H-12->LUMO (17%), HOMO->L+7 (56%)	0.48
**22**	4.45	278.70	0.0241	A	H-11->LUMO (15%), H-3->L+1 (21%), HOMO->L+7 (27%)	0.48

**23**	4.47	277.32	0.0022	A	H-12->LUMO (23%), H-4->L+1 (10%), H-3->L+1 (16%), HOMO->L+8 (28%)	0.54
**24**	4.48	276.47	0.0316	A	H-4->L+1 (10%), HOMO->L+8 (30%), HOMO->L+9 (36%)	0.53
**25**	4.51	275.06	0.0296	A	H-12->LUMO (14%), H-4->L+1 (17%), HOMO->L+8 (12%), HOMO->L+9 (29%)	0.51

b) Cartesian coordinates

Compound **4a**

DFT B3LYP/6-31G+g(d), gas phase, S_0

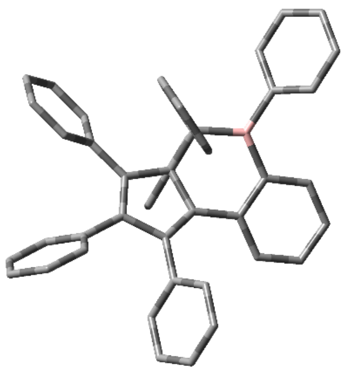

Point group: C_1

Total energy: -1,176,650.13 kcal mol^{-1}

Dipole moment: 0.73 D

Imaginary frequencies: 0

C	-3.996019	-0.162509	-1.403140
H	-3.403294	0.376284	-2.137132
C	1.490253	-0.674690	-0.154369
H	1.878985	-1.641448	0.187651
B	2.637848	0.442723	-0.105741
C	-0.139437	1.138400	0.462571
C	0.275534	-0.297275	0.761947
C	-1.993729	-0.212783	0.123613
C	-1.001996	-1.088864	0.468648
C	-1.456512	1.170936	0.108311
C	0.863081	2.199061	0.598872
C	2.237349	1.880163	0.353436
C	3.196904	2.891696	0.575033
H	4.248105	2.659898	0.431381
C	2.840868	4.171559	0.994189
H	3.604691	4.926603	1.162687
C	1.490902	4.471040	1.210394
H	1.199033	5.462403	1.549359
C	0.514814	3.495373	1.020376
H	-0.523517	3.730976	1.228009
C	-2.261916	2.361728	-0.266684
C	-1.887183	3.176928	-1.348125
H	-0.996063	2.928758	-1.917650
C	-2.643690	4.297198	-1.697659
H	-2.335906	4.913027	-2.539300
C	-3.791428	4.625500	-0.970052
H	-4.380765	5.497922	-1.240816
C	-3.424519	2.696314	0.448956
H	-3.738111	2.072418	1.281560
C	-3.409106	-0.542859	-0.183993
C	-4.178358	3.820428	0.105205
H	-5.071400	4.063764	0.675556
C	-5.326245	-0.479708	-1.685658
H	-5.759105	-0.182198	-2.637676
C	-6.099163	-1.173262	-0.749739
H	-7.135850	-1.416814	-0.968545
C	-5.529701	-1.549726	0.470331
H	-6.121793	-2.088541	1.206141
C	-4.197577	-1.238127	0.749049
H	-3.757532	-1.539091	1.696141
C	-1.076027	-2.565890	0.489849
C	-1.772588	-3.256395	-0.524220
H	-2.247331	-2.693183	-1.320375

C	-1.840567	-4.648424	-0.536924
H	-2.382344	-5.149904	-1.335347
C	-1.207877	-5.396983	0.460988
H	-1.260110	-6.482879	0.451026
C	-0.503932	-4.731949	1.467242
H	-0.006854	-5.297886	2.251683
C	-0.435586	-3.336437	1.481105
H	0.104040	-2.845015	2.282175
C	0.631498	-0.379936	2.273498
C	-0.357220	-0.057799	3.220693
H	-1.347311	0.228116	2.877374
C	-0.095379	-0.099411	4.588350
H	-0.883628	0.153448	5.293488
C	1.173925	-0.463169	5.053365
H	1.381405	-0.497109	6.119805
C	2.167450	-0.778525	4.129059
H	3.161934	-1.059552	4.467212
C	1.897301	-0.736501	2.754617
H	2.698080	-0.997275	2.072588
C	4.087299	0.063511	-0.581901
C	4.827565	0.897586	-1.448477
H	4.390597	1.831815	-1.792134
C	6.096072	0.538713	-1.909553
H	6.630324	1.196558	-2.591152
C	6.679104	-0.661158	-1.492844
H	7.671521	-0.937983	-1.840490
C	5.973086	-1.507306	-0.632933
H	6.414536	-2.446469	-0.307694
C	4.692210	-1.154677	-0.203886
H	4.157355	-1.843171	0.447072
C	1.139804	-0.879320	-1.631772
C	0.801900	0.186008	-2.484654
H	0.750689	1.196621	-2.092272
C	0.534833	-0.027955	-3.839910
H	0.275615	0.816130	-4.475015
C	0.611048	-1.313653	-4.379726
H	0.407211	-1.480269	-5.434536
C	0.959920	-2.382170	-3.549226
H	1.026572	-3.389604	-3.953098
C	1.222084	-2.163659	-2.195507
H	1.486677	-3.006084	-1.561440

Compound **4b**

DFT B3LYP/6-31G+g(d), gas phase, S_0

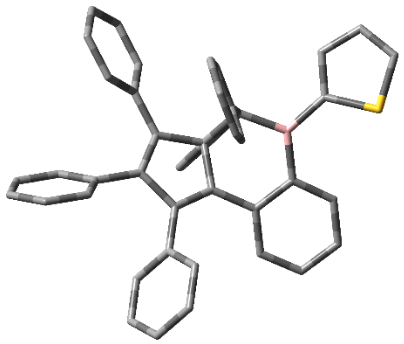

Point group: C_1

Total energy: -1,377,930.98 kcal mol^{-1}

Dipole moment: 0.14 D

Imaginary frequencies: 0

S	5.34468868	0.79640198	-1.21525745
C	4.04659413	-0.21298386	-0.58517924
B	2.65006806	0.29718990	-0.12434251
C	4.46992813	-1.53566432	-0.62855321
C	5.78441984	-1.73302297	-1.12749039
C	6.37981764	-0.55167970	-1.49966762
C	2.32045902	1.74421047	0.37030596
C	3.31378761	2.71924699	0.60780415
C	3.00827765	4.00642978	1.04261966
C	1.67239302	4.35579696	1.26733344
C	0.66249401	3.41866986	1.06729909
C	0.95971069	2.11617885	0.62470336
C	-0.09107921	1.10530979	0.48199629
C	-1.41073852	1.20459221	0.14951917

C -2.16710940 2.43596626 -
0.19564706
C -1.77892449 3.24242194 -
1.27881561
C -2.48885300 4.40084151 -
1.60056903
C -4.00252363 3.98023197
0.23385526
C -3.60229966 4.77643370 -
0.84312794
C -2.01109105 -0.15234884
0.15291276
C -3.29556154 2.81815776
0.55002664
C -3.44668293 -0.41016530 -
0.12939917
C -4.24872126 -1.07804181
0.81196047
C -5.59974344 -1.32172864
0.55747340
C -6.17476881 -0.90348117 -
0.64625822
C -5.38829421 -0.23639757 -
1.59009873
C -4.03885599 0.01308435 -
1.33171801
C -1.05486312 -1.07970293
0.46291537
C -1.20077299 -2.55174444
0.46094881
C -0.57911663 -3.37169722
1.42420145
C -0.71682941 -4.76174622
1.38710426
C -1.47402264 -5.37258738
0.38522590
C -2.09017068 -4.57502497 -
0.58474463
C -1.95265354 -3.18853606 -
0.54905464
C 0.26385105 -0.35175640
0.74229373
C 1.43509281 -0.75276744 -
0.21970581
H 1.76479036 -1.75579362
0.07415488
C 1.03316535 -0.87173515 -
1.69340604
C 0.79911472 0.24980676 -
2.50785489
C 0.47800979 0.10833555 -
3.86086338
C 0.39250701 -1.16083651 -
4.43724863
C 0.63529372 -2.28647823 -
3.64547430
C 0.95327998 -2.13933449 -
2.29389124
C 0.65846936 -0.49509536
2.23850983
C 1.90584523 -0.96275563
2.67143343
C 2.20947461 -1.06175603
4.03599945
C 1.27002892 -0.69454271
4.99712207
C 0.02048684 -0.22099759
4.57983127
C -0.27544491 -0.12281160
3.22201101
H 3.80271032 4.72745155
1.21795663
H 4.35805732 2.45976678
0.47041299
H 1.41881403 5.35248944
1.62092907
H -0.36576121 3.68803972
1.28289344
H 7.36092449 -0.40786206 -
1.93511215
H 6.26222297 -2.70286741 -
1.21951452
H 3.84040204 -2.35737534 -
0.30307859
H 1.13281751 -3.02576421 -
1.69065116
H 0.57574778 -3.28256536 -
4.07775449
H 0.14661766 -1.27129942 -
5.49047722
H 0.30475237 0.99528291 -
4.46608598

H 0.87740614 1.24796273 -2.08864787
H -2.41596398 -2.58818646 -1.32442971
H 0.00033963 -2.92301506 2.22227552
H -0.23232418 -5.36583265 2.15069524
H -1.58057152 -6.45419959 0.35745535
H -2.67384366 -5.03395771 -1.37924706
H -3.80462492 -1.41116347 1.74630961
H -6.20227084 -1.84025196 1.29937063
H -7.22623385 -1.09416694 -0.84625380
H -5.82541208 0.09331305 -2.52947759
H -3.43579866 0.53184655 -2.07167993
H -3.61922831 2.20171834 1.38436541
H -4.86952357 4.26028952 0.82709967
H -4.15519694 5.67865387 -1.09228779
H -2.17161583 5.00943949 -2.44395935
H -0.91472971 2.95631238 -1.87141460
H -1.24931356 0.24810244 2.91511322
H -0.72521241 0.07360176 5.31443916
H 1.50420325 -0.77253820 6.05572208
H 3.18771812 -1.42846055 4.33754096
H 2.66446169 -1.26711661 1.95933940

E. Mechanistic DFT Calculations

a) Computational Method

DFT calculations were carried out at the M06-2X level of theory, which is well established for studying the main-group thermochemistry, kinetics, and noncovalent interaction computations.[14-17] All the molecular geometries were fully optimized in toluene solvent employing the SMD model.[18] The ultrafine integration grid was used to enhance calculation accuracy. Grimme's D3 dispersion correction was also introduced taking account of the dispersion effect.[19] The 6-31G(d,p) Pople basis set[20] was employed for H, B, C and N atoms while 6-311+G(d,p) was employed for Cl. Vibrational frequency calculations were performed at the same level of theory to ensure that a transition state has only one imaginary frequency and a local minimum has no imaginary frequency. Intrinsic reaction coordinate (IRC)[21] calculations were run to ensure that transition states indeed connect two relevant minima. All of the DFT calculations were performed with the Gaussian 09 v.D.01 package.[22]

b) Figure S97

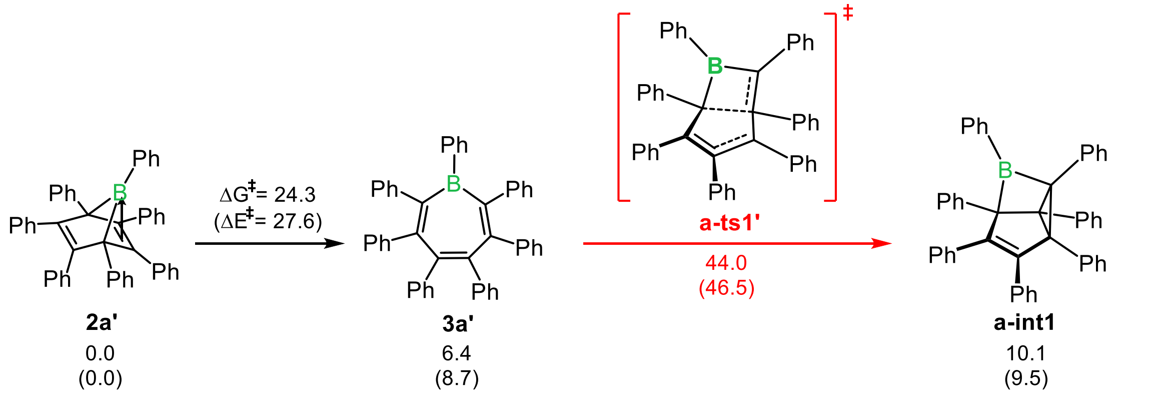

Figure S97. Calculated energetics for the unfavorable pathway from **3a'** to **a-int1**. Relative free energies and electronic energies (in parenthesis) are given in kcal/mol.

c) Energy Profiles

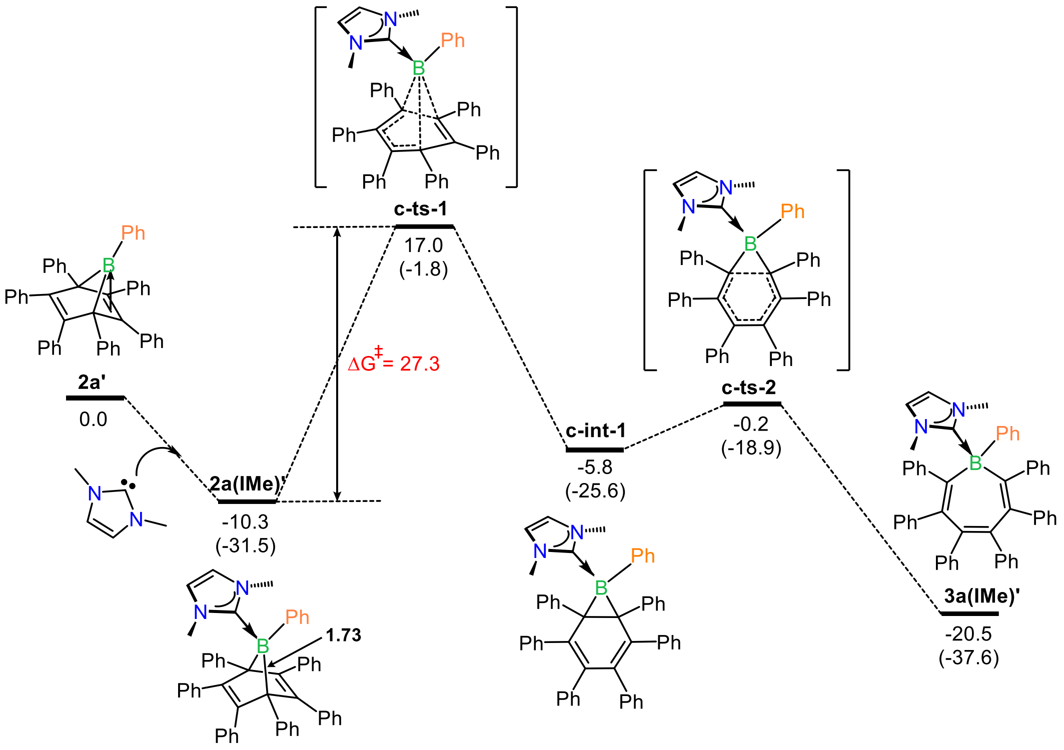

Figure S98. Energy profile calculated for the pathway to form the borepin base adduct **3a(*I*Me)'** from the reaction of **2a'** and *I*Me.

(a)

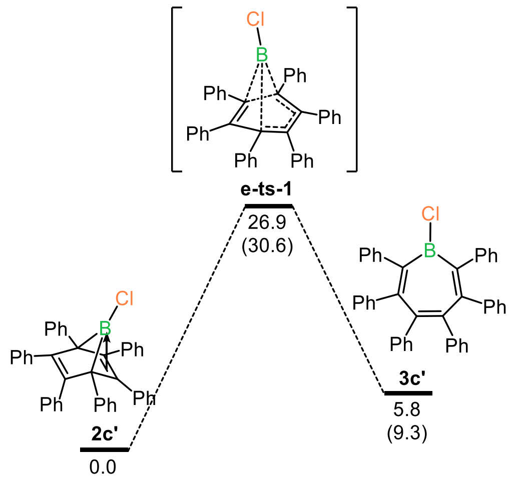

(b)

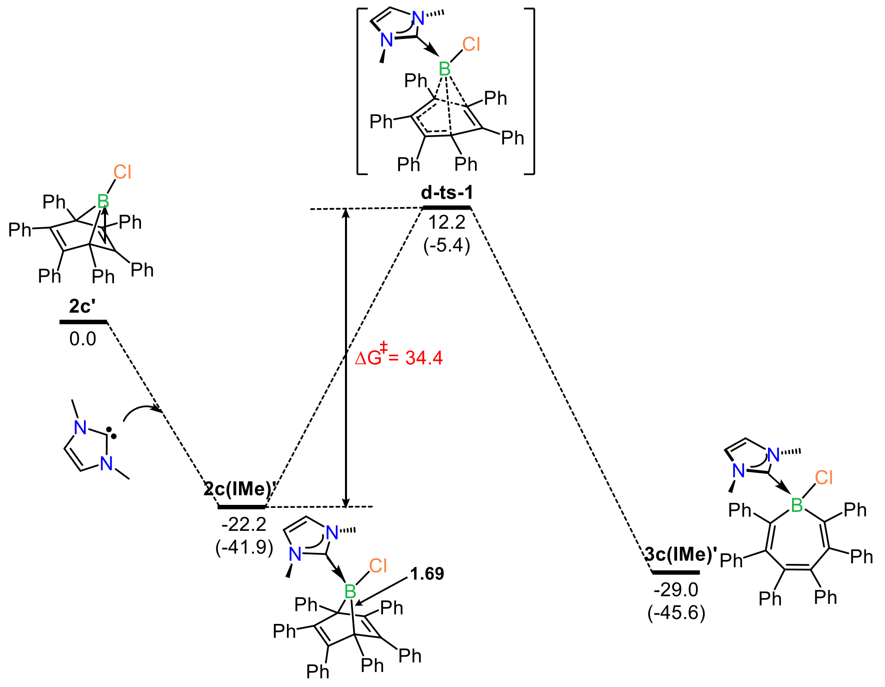

Figure S99. Energy profiles calculated for a) the interconversion between **2c’** and **3c’**, and b) formation of the borepin base adduct **3c(IMe)’** from the reaction of **2c’** and *I*Me.

c) Cartesian Coordinates

2a’

E(M062X/6-31G**) = -1874.335888 a.u.

B	-0.4221660	-0.6261710	-0.8552510
C	-1.0270360	0.7755450	-0.2403760
C	0.1120800	1.7712310	-0.1786550
C	1.3005340	1.1500420	-0.2972290
C	1.1264760	-0.3466280	-0.4535850
C	-2.3647290	1.2075060	-0.7751130
C	-3.5499250	0.4822830	-0.5900850
C	-4.7358640	0.8716710	-1.2080470
C	-4.7713950	1.9845880	-2.0408560
C	-3.5960530	2.6972750	-2.2616360
C	-2.4126910	2.3120490	-1.6412340
C	-0.0839390	3.1951860	0.1775890
C	-1.1299630	3.5871230	1.0235450
C	-1.3079170	4.9219400	1.3722090
C	-0.4520570	5.8981180	0.8687140
C	0.5762160	5.5262090	0.0052930
C	0.7561870	4.1915900	-0.3410730
C	2.6414350	1.7376380	-0.0533550
C	2.9663230	2.2489840	1.2085010
C	4.2268460	2.7861610	1.4522100
C	5.1787910	2.8228450	0.4358610
C	4.8664920	2.3134540	-0.8226190
C	3.6088080	1.7687850	-1.0646700
C	2.2837610	-1.1373640	-0.9873050
C	3.4342050	-1.3408600	-0.2144250
C	4.5150580	-2.0509660	-0.7240740
C	4.4646750	-2.5672810	-2.0185630
C	3.3314240	-2.3621720	-2.7994030
C	2.2484480	-1.6483440	-2.2871040
C	-1.1259850	-1.6431790	-1.8223510
C	-1.7184900	-1.1468590	-2.9953750
C	-2.3239080	-1.9959440	-3.9175860
C	-2.3553530	-3.3692320	-3.6858550
C	-1.7779540	-3.8844080	-2.5282970
C	-1.1705850	-3.0288700	-1.6118460
C	-0.8901190	-0.2831600	0.8242680
C	0.3513780	-0.9210250	0.7127580
C	-1.9629030	-0.7509610	1.7405150
C	-2.5386780	0.1636820	2.6257330
C	-2.4045710	-2.0777750	1.7424770
C	-3.5431010	-0.2406440	3.5001660
H	-2.1895490	1.1912570	2.6336350
C	-3.4167500	-2.4767800	2.6085390
H	-1.9665790	-2.7886890	1.0493200
C	-3.9877900	-1.5600380	3.4895080
H	-3.9813170	0.4777450	4.1853740
H	-3.7597060	-3.5062270	2.5945970
H	-4.7772830	-1.8738960	4.1647210
C	0.7737440	-2.0722130	1.5518910
C	0.7050810	-1.9275040	2.9435170
C	1.2775850	-3.2654850	1.0198770
C	1.1168020	-2.9557960	3.7839470
H	0.3225680	-1.0025410	3.3644020

C	1.6805190	-4.2956270	1.8648360
H	1.3605150	-3.3893480	-0.0544740
C	1.6025500	-4.1453980	3.2467950
H	1.0567840	-2.8256730	4.8596200
H	2.0634120	-5.2161880	1.4363950
H	1.9219690	-4.9496450	3.9017700
H	-1.8225160	2.8389190	1.3948780
H	-2.1234470	5.1992510	2.0328790
H	-0.5918960	6.9405020	1.1368490
H	1.2394190	6.2795740	-0.4082780
H	1.5512870	3.9150100	-1.0256600
H	2.2188900	2.2253050	1.9965140
H	4.4644700	3.1784320	2.4361690
H	5.6052740	2.3353000	-1.6176710
H	3.3702200	1.3609760	-2.0429120
H	6.1612180	3.2442570	0.6245020
H	3.4758460	-0.9378820	0.7941680
H	1.3651960	-1.4879330	-2.8981620
H	3.2862260	-2.7556460	-3.8101070
H	5.3076600	-3.1236570	-2.4165000
H	5.3981180	-2.2017900	-0.1111160
H	-0.7237740	-3.4516440	-0.7164090
H	-1.7125940	-0.0758500	-3.1827230
H	-2.7754920	-1.5837350	-4.8148190
H	-2.8293870	-4.0335300	-4.4019210
H	-1.7996800	-4.9530810	-2.3373600
H	-3.5587840	-0.4145280	0.0155100
H	-5.6346960	0.2861420	-1.0409920
H	-5.6966810	2.2838660	-2.5227260
H	-3.5934300	3.5570880	-2.9245540
H	-1.5074130	2.8723680	-1.8468660

a-ts1

E(M062X/6-31G**) = -1874.282133 a.u.

B	0.9971140	-0.5404460	-0.8436830
C	0.5761500	0.9382380	-0.6756710
C	0.8319010	0.1133350	0.6527770
C	0.1020450	-1.1531280	0.4151380
C	-1.2753500	-1.0199740	-0.0550070
C	-1.7460380	0.2828220	-0.1479750
C	-0.7441440	1.2968740	-0.1387900
C	0.5136290	-2.3549090	1.2112770
C	0.3135790	-2.3415370	2.5973910
C	0.6392750	-3.4494620	3.3721930
C	1.1681030	-4.5903020	2.7692690
C	1.3715800	-4.6116020	1.3929380
C	1.0494070	-3.4985490	0.6178370
C	-2.0771600	-2.1976320	-0.4824900
C	-2.6441030	-2.1939620	-1.7648410
C	-3.4113610	-3.2646750	-2.2089080
C	-3.6330260	-4.3566280	-1.3723490
C	-3.0821330	-4.3693990	-0.0936980
C	-2.3050830	-3.3022030	0.3487780
C	-3.1896640	0.6156810	-0.2683380
C	-4.1422730	-0.0271240	0.5329440
C	-5.4945920	0.2756510	0.4145980
C	-5.9201460	1.2353880	-0.5011660

C	-4.9831670	1.8860100	-1.3005940
C	-3.6318390	1.5759110	-1.1876280
C	-1.0778720	2.6691890	0.3257090
C	-0.8036020	3.8119340	-0.4372350
C	-1.1865850	5.0708970	0.0174430
C	-1.8381980	5.2093040	1.2399890
C	-2.1241150	4.0792800	2.0043300
C	-1.7542140	2.8210830	1.5452420
C	2.1673500	-1.1524230	-1.6869610
C	1.8914760	-2.1828330	-2.5992610
C	2.8908180	-2.7312920	-3.3992260
C	4.1960050	-2.2537250	-3.3063920
C	4.4903520	-1.2233520	-2.4161560
C	3.4863950	-0.6773370	-1.6201050
C	1.5400420	1.9741810	-1.1713450
C	2.2291070	2.8654190	-0.3431530
C	1.7328070	2.0560940	-2.5555220
C	3.0889050	3.8157090	-0.8899770
C	2.5823020	3.0125700	-3.1022500
H	1.2143010	1.3517410	-3.2001190
C	3.2655390	3.8954560	-2.2684730
H	3.6165120	4.4996520	-0.2325130
H	2.7185110	3.0606630	-4.1780540
H	3.9340340	4.6389480	-2.6910700
C	1.7659750	0.3974020	1.7690030
C	2.9582430	-0.3242120	1.9010650
C	1.4464780	1.3646960	2.7292330
C	3.8152360	-0.0752300	2.9694920
H	3.2023120	-1.0889300	1.1690640
C	2.3147460	1.6231940	3.7858140
H	0.5204100	1.9246650	2.6347070
C	3.5004070	0.9024370	3.9100790
H	4.7339960	-0.6455790	3.0624520
H	2.0587490	2.3836880	4.5169370
H	4.1738990	1.0996570	4.7380260
H	-0.3088850	3.7157280	-1.3974790
H	-1.9872460	1.9336920	2.1280780
H	-2.6409540	4.1778670	2.9534980
H	-0.9747340	5.9457850	-0.5887620
H	-2.1302220	6.1934810	1.5925360
H	-2.9051860	2.0784250	-1.8198450
H	-3.8119360	-0.7687730	1.2545080
H	-6.2167340	-0.2345750	1.0444820
H	-6.9749730	1.4753730	-0.5904520
H	-5.3051120	2.6336340	-2.0188640
H	-2.4756210	-1.3363190	-2.4098700
H	-1.8893280	-3.3212080	1.3503500
H	-0.1000010	-1.4506900	3.0636060
H	-3.2569190	-5.2133650	0.5660900
H	-4.2353640	-5.1923430	-1.7146380
H	-3.8388320	-3.2451360	-3.2063470
H	0.4811850	-3.4233800	4.4458690
H	1.4211940	-5.4571710	3.3716560
H	1.7834060	-5.4958870	0.9167300
H	1.2080610	-3.5205510	-0.4548360
H	5.5038710	-0.8404410	-2.3435800

H	4.9785220	-2.6788890	-3.9273880
H	2.6498050	-3.5286010	-4.0959410
H	0.8741730	-2.5605650	-2.6836040
H	3.7283300	0.1375970	-0.9417400
H	2.0860630	2.8304370	0.7307550

a-int1

E(M062X/6-31G**) = -1874.320800 a.u.

B	-1.0418800	-0.0883230	-1.0119770
C	1.5527100	0.8909620	0.0823630
C	1.0861490	-0.5242420	0.2881390
C	-0.3857380	-0.4826560	0.6473710
C	0.1185730	-1.0419510	-0.7859960
C	2.9611460	1.2047430	-0.2542790
C	3.9925180	1.0094070	0.6728010
C	5.3104310	1.3040360	0.3364820
C	5.6180390	1.7910350	-0.9318930
C	4.6003910	1.9825400	-1.8636980
C	3.2817970	1.6919770	-1.5273230
C	2.0656370	-1.4999460	0.8725530
C	2.1006670	-1.7872390	2.2377010
C	3.0766630	-2.6380940	2.7557920
C	4.0335560	-3.1991710	1.9162100
C	4.0144310	-2.9051710	0.5536000
C	3.0377050	-2.0623870	0.0372590
C	-1.1318920	-1.1989650	1.7182940
C	-1.9726200	-2.2768480	1.4396080
C	-2.7236650	-2.8651060	2.4559550
C	-2.6524720	-2.3696010	3.7536230
C	-1.8352570	-1.2744960	4.0343110
C	-1.0852750	-0.6889800	3.0222370
C	0.2223570	-2.4237130	-1.3300210
C	0.2923600	-3.5886550	-0.5559920
C	0.3954210	-4.8357290	-1.1668860
C	0.4482060	-4.9402230	-2.5546300
C	0.3990440	-3.7860840	-3.3330230
C	0.2855660	-2.5403510	-2.7245560
C	-2.3685430	-0.3585140	-1.8121800
C	-3.1352410	0.7436520	-2.2256470
C	-4.3012340	0.5889430	-2.9684710
C	-4.7455800	-0.6884720	-3.3019120
C	-4.0134640	-1.8001240	-2.8927250
C	-2.8370460	-1.6349640	-2.1657140
C	0.4904560	1.7136410	0.0102260
C	-0.8054720	0.9608620	0.1757320
C	-1.9264050	1.6043620	0.9516750
C	-1.6877080	2.7314380	1.7489840
C	-3.2071050	1.0337900	1.0008320
C	-2.6942820	3.2871390	2.5349640
H	-0.7020490	3.1828620	1.7663450
C	-4.2107200	1.5820360	1.7922590
H	-3.4213960	0.1370650	0.4306480
C	-3.9633530	2.7184150	2.5578990
H	-2.4771120	4.1647070	3.1362550
H	-5.1894780	1.1127590	1.8107240
H	-4.7477090	3.1484550	3.1728270
C	0.5362450	3.1398390	-0.3790390

C	-0.4110140	3.6675850	-1.2639890
C	1.5209460	3.9938090	0.1369530
C	-0.3675980	5.0074900	-1.6364330
H	-1.1776420	3.0162070	-1.6713850
C	1.5607690	5.3334970	-0.2327880
H	2.2515340	3.6001350	0.8370580
C	0.6179860	5.8459790	-1.1221370
H	-1.1080880	5.3965080	-2.3282890
H	2.3294340	5.9803710	0.1784690
H	0.6492120	6.8924410	-1.4082660
H	-2.2790840	-2.5187590	-1.8720220
H	-4.3555920	-2.7996280	-3.1431320
H	-5.6599590	-0.8170010	-3.8731530
H	-4.8689370	1.4621760	-3.2745840
H	-2.8243970	1.7431960	-1.9343170
H	3.7569860	0.6232970	1.6604810
H	6.0983650	1.1511730	1.0673230
H	6.6466740	2.0183750	-1.1937210
H	4.8327710	2.3587310	-2.8551290
H	2.4853180	1.8439950	-2.2501360
H	1.3663370	-1.3467530	2.9023860
H	3.0874840	-2.8572810	3.8188520
H	4.7938700	-3.8600600	2.3204370
H	4.7608290	-3.3327480	-0.1080840
H	3.0230560	-1.8320860	-1.0245820
H	-2.0498640	-2.6457090	0.4218500
H	-3.3713780	-3.7051130	2.2265400
H	-0.4713390	0.1838260	3.2300690
H	-1.7924270	-0.8695280	5.0401740
H	-3.2410300	-2.8250370	4.5435960
H	0.2699810	-3.5219260	0.5272240
H	0.2306400	-1.6391690	-3.3286890
H	0.4433180	-3.8540140	-4.4154470
H	0.5309490	-5.9143090	-3.0261250
H	0.4415910	-5.7285270	-0.5512660

a-ts2

E(M062X/6-31G**) = -1874.270511 a.u.

B	0.4794560	-0.8606850	1.2356060
C	-0.9364380	1.2001240	-0.5076010
C	-0.9812610	-0.3216640	-0.5606540
C	0.4728850	-0.6847720	-0.8705840
C	-0.9012400	-0.8532670	0.9107400
C	-2.1264770	2.0111530	-0.1756830
C	-3.0775170	2.3482070	-1.1457230
C	-4.1965900	3.0990950	-0.7996170
C	-4.3831770	3.5111620	0.5186480
C	-3.4426780	3.1765360	1.4895140
C	-2.3169450	2.4335150	1.1442980
C	-2.0727410	-0.8206640	-1.4898570
C	-1.7932330	-1.2248740	-2.7995510
C	-2.8193850	-1.5879240	-3.6690700
C	-4.1449700	-1.5452410	-3.2482510
C	-4.4379020	-1.1192970	-1.9551510
C	-3.4132390	-0.7522040	-1.0880790
C	0.8889380	-2.0026580	-1.4174110
C	0.3103070	-3.2005320	-0.9764970

C	0.6456180	-4.4109910	-1.5749520	H	6.5112040	0.8736480	-1.6788050
C	1.5595270	-4.4485310	-2.6248300	C	0.7995610	3.0031690	-0.2508250
C	2.1308010	-3.2624530	-3.0791170	C	1.8344420	3.2297620	0.6660430
C	1.7996460	-2.0500800	-2.4830160	C	0.1885190	4.1035170	-0.8628800
C	-1.9839710	-1.3702650	1.7730390	C	2.2393170	4.5247060	0.9708360
C	-2.8121360	-2.4426900	1.3974080	H	2.3255900	2.3820320	1.1351140
C	-3.7657320	-2.9518580	2.2721940	C	0.5966650	5.3987220	-0.5585690
C	-3.9067190	-2.4194780	3.5526970	H	-0.6089660	3.9372830	-1.5804360
C	-3.0803500	-1.3716250	3.9490050	C	1.6216230	5.6138990	0.3595330
C	-2.1355500	-0.8513530	3.0689750	H	3.0401360	4.6825640	1.6864050
C	1.7033350	-1.2116580	2.0990910	H	0.1128040	6.2414700	-1.0421620
C	2.3857210	-0.2228510	2.8259320	H	1.9393360	6.6244620	0.5958280
C	3.5154270	-0.5351360	3.5756380	H	1.6640180	-3.3121240	1.6093770
C	3.9829910	-1.8475010	3.6105070	H	3.6705480	-3.8714210	2.9434650
C	3.3116630	-2.8475800	2.9085030	H	4.8680500	-2.0924170	4.1897940
C	2.1797640	-2.5322320	2.1627790	H	4.0333530	0.2421920	4.1285130
C	0.3454230	1.6251690	-0.5372720	H	2.0224410	0.8011990	2.7992980
C	1.2416640	0.4516280	-0.7054910	H	-2.9354440	2.0156560	-2.1702050
C	2.7061260	0.5611080	-0.9141970	H	-4.9257080	3.3598540	-1.5602820
C	3.1956230	1.6135800	-1.7027700	H	-5.2591810	4.0931480	0.7870780
C	3.6142520	-0.3847690	-0.4217880	H	-3.5823120	3.4960510	2.5173860
C	4.5540130	1.7204700	-1.9807440	H	-1.5733690	2.1754030	1.8928250
H	2.5059930	2.3457580	-2.1098320	H	-0.7681200	-1.2565120	-3.1518670
C	4.9738550	-0.2673950	-0.6914160	H	-2.5758580	-1.9030800	-4.6788870
H	3.2579610	-1.2218480	0.1655490	H	-4.9440420	-1.8325350	-3.9244730
C	5.4500380	0.7853980	-1.4685370	H	-5.4679810	-1.0630490	-1.6175220
H	4.9108580	2.5388040	-2.5978560	H	-3.6581840	-0.3988310	-0.0924980
H	5.6602560	-1.0075510	-0.2927250	H	-0.3972120	-3.1774120	-0.1540240

H	0.1879500	-5.3276610	-1.2168070
H	2.2344300	-1.1297370	-2.8591910
H	2.8350530	-3.2763900	-3.9049240
H	1.8202430	-5.3939940	-3.0895960
H	-2.7020000	-2.8805390	0.4098100
H	-1.4970190	-0.0284490	3.3777290
H	-3.1743020	-0.9500160	4.9453500
H	-4.6499300	-2.8206540	4.2343690
H	-4.3948510	-3.7781330	1.9546830

a-int2

E(M062X/6-31G**) = -1874.285094 a.u.

B	0.0842490	-1.0594380	1.7402840
C	0.7022920	-0.7712830	-0.7057650
C	1.2868020	0.4588820	-0.7201040
C	0.2428590	1.5015850	-0.6331230
C	-0.9749620	0.9121120	-0.5628840
C	1.3424730	-2.0710540	-1.0090300
C	2.4118330	-2.1324620	-1.9187200
C	3.0150810	-3.3425620	-2.2372830
C	2.5567420	-4.5298240	-1.6697270
C	1.4788600	-4.4917340	-0.7911530
C	0.8740540	-3.2796000	-0.4687740
C	2.7325480	0.7635520	-0.8132600
C	3.2028450	1.7046150	-1.7375860
C	4.5613220	1.9941490	-1.8214770
C	5.4662220	1.3594590	-0.9733380
C	5.0060790	0.4269530	-0.0457710
C	3.6500100	0.1276590	0.0328890
C	0.5079850	2.9549710	-0.5409440
C	-0.2139990	3.8647350	-1.3224010
C	0.0176960	5.2324350	-1.2142550
C	0.9767510	5.7122900	-0.3255410
C	1.7040420	4.8155560	0.4542150
C	1.4749560	3.4479420	0.3454940
C	-2.2584700	1.6172690	-0.3421600
C	-3.2663110	1.6271990	-1.3137750
C	-4.4763960	2.2696060	-1.0660650
C	-4.6941680	2.9094320	0.1519850
C	-3.6913580	2.9150370	1.1188500
C	-2.4805350	2.2771600	0.8717570
C	-1.1071800	-0.9987570	0.9948780
C	-0.8170220	-0.6073400	-0.4841790
C	-1.5658680	-1.3794780	-1.5694210
C	-2.2315830	-2.5797130	-1.3223890
C	-1.4938750	-0.9127060	-2.8872040
C	-2.8394270	-3.2811190	-2.3631510
H	-2.2873840	-2.9636610	-0.3089770
C	-2.0970060	-1.6109750	-3.9268730
H	-0.9529830	0.0088210	-3.0920160
C	-2.7787750	-2.7989240	-3.6660380
H	-3.3615210	-4.2084590	-2.1490340
H	-2.0318950	-1.2303410	-4.9414580
H	-3.2537730	-3.3452440	-4.4746690
C	-2.4431290	-1.1231900	1.6168660
C	-3.6575300	-0.9035020	0.9493260
C	-2.5202200	-1.4844200	2.9776490

C	-4.8774610	-1.0109240	1.6136970
H	-3.6570350	-0.6353140	-0.0990490
C	-3.7336360	-1.5899310	3.6398320
H	-1.6009080	-1.6878640	3.5259510
C	-4.9289370	-1.3500490	2.9608780
H	-5.7937100	-0.8211610	1.0623170
H	-3.7476110	-1.8652840	4.6902720
H	-5.8809820	-1.4319150	3.4752070
C	1.3684270	-1.0517820	2.5449390
C	1.8307510	0.1652790	3.0810470
C	2.1712950	-2.1992390	2.6752290
C	3.0630990	0.2354880	3.7199020
C	3.4076820	-2.1261400	3.3076120
C	3.8526420	-0.9095580	3.8252020
H	0.0326170	-3.2692370	0.2140040
H	1.0964200	-5.4099610	-0.3560060
H	3.0268490	-5.4752060	-1.9214790
H	3.8386800	-3.3579650	-2.9443210
H	2.7635840	-1.2225540	-2.3919700
H	2.4983500	2.2084500	-2.3930230
H	4.9117900	2.7211010	-2.5472330
H	6.5247190	1.5920530	-1.0343880
H	5.7030020	-0.0711650	0.6213100
H	3.2952010	-0.6083320	0.7483960
H	-0.9617330	3.4913840	-2.0156840
H	-0.5513130	5.9240990	-1.8274950
H	1.1583870	6.7791490	-0.2422660
H	2.4541340	5.1808680	1.1486060
H	2.0496220	2.7518540	0.9503000
H	1.8308170	-3.1414930	2.2566270
H	4.0263200	-3.0132850	3.3944640
H	4.8203480	-0.8540230	4.3144780
H	3.4148060	1.1790720	4.1238850
H	1.2186020	1.0571680	2.9775390
H	-3.1050600	1.1201960	-2.2602540
H	-5.2509530	2.2682570	-1.8268350
H	-5.6403960	3.4048780	0.3456810
H	-3.8538430	3.4123070	2.0698590
H	-1.6993690	2.2674100	1.6267050

a-ts3

E(M062X/6-31G**) = -1874.265619 a.u.

B	2.4288180	0.7890480	-0.2434070
C	-1.0464370	-0.9148560	0.2538330
C	-1.9955600	-0.0686140	-0.2110470
C	-1.5187100	1.3316900	-0.0856210
C	-0.2933400	1.3194310	0.4839310
C	-1.1567760	-2.3920080	0.2697590
C	-1.0668660	-3.1366410	1.4529190
C	-1.1565870	-4.5259690	1.4191550
C	-1.3399860	-5.1909720	0.2099180
C	-1.4462330	-4.4566260	-0.9701610
C	-1.3600310	-3.0700790	-0.9398690
C	-3.3222510	-0.4447440	-0.7502480
C	-4.1509110	-1.3257350	-0.0446430
C	-5.3977850	-1.6806500	-0.5483540
C	-5.8350610	-1.1609840	-1.7647660

C	-5.0189630	-0.2823410	-2.4735970
C	-3.7731280	0.0773920	-1.9691000
C	-2.2819250	2.5191250	-0.5274980
C	-3.5909140	2.7313490	-0.0787130
C	-4.3027970	3.8545820	-0.4879240
C	-3.7202750	4.7733100	-1.3584420
C	-2.4219410	4.5637730	-1.8187990
C	-1.7064430	3.4437650	-1.4072280
C	0.6223500	2.4202790	0.7816660
C	0.2103770	3.6651850	1.2607810
C	1.1496610	4.6500650	1.5518600
C	2.5125280	4.4023520	1.3748090
C	2.9369380	3.1690230	0.8997560
C	2.0023480	2.1738430	0.5640040
C	1.4653900	-0.3164810	-0.0535940
C	0.1610300	-0.1064210	0.7529220
C	1.7178230	-1.6433440	-0.6713230
C	1.8277910	-2.8322330	0.0652610
C	1.8631620	-1.7095740	-2.0638630
C	2.0645060	-4.0453010	-0.5722700
H	1.7116290	-2.8117190	1.1435700
C	2.0817880	-2.9258980	-2.7036000
H	1.7951670	-0.7918960	-2.6409190
C	2.1840130	-4.0984290	-1.9598390
H	2.1407850	-4.9537200	0.0174360
H	2.1782580	-2.9545570	-3.7845640
H	2.3570270	-5.0476720	-2.4572340
C	0.3048090	-0.3168690	2.2799020

C	-0.7954920	-0.0172650	3.0956270
C	1.4681520	-0.7935110	2.8819810
C	-0.7363010	-0.2029060	4.4695850
H	-1.7073220	0.3589100	2.6385140
C	1.5263620	-0.9887610	4.2645550
H	2.3413450	-1.0197140	2.2781110
C	0.4276910	-0.6978540	5.0615450
H	-1.5999860	0.0367350	5.0820270
H	2.4397640	-1.3697680	4.7103660
H	0.4733630	-0.8487950	6.1352480
C	3.8449350	0.7348130	-0.9415100
C	4.7162160	-0.3539880	-0.7831300
C	4.2679070	1.7799940	-1.7768150
C	5.9522450	-0.3964440	-1.4222560
C	5.4988470	1.7433300	-2.4280760
C	6.3471070	0.6533530	-2.2491140
H	-1.4261700	-2.4971840	-1.8601380
H	-1.5875230	-4.9653380	-1.9187030
H	-1.4032750	-6.2744470	0.1865570
H	-1.0842480	-5.0888090	2.3448910
H	-0.9250100	-2.6311050	2.4026590
H	-3.8080260	-1.7324540	0.9021950
H	-6.0286450	-2.3641770	0.0111200
H	-6.8081590	-1.4387810	-2.1573960
H	-5.3529650	0.1259690	-3.4222480
H	-3.1415330	0.7660660	-2.5228700
H	-4.0493190	2.0100440	0.5919640
H	-5.3155320	4.0093310	-0.1295940

H	-4.2782070	5.6468430	-1.6804400
H	-1.9656020	5.2724300	-2.5026820
H	-0.6953230	3.2751970	-1.7663230
H	3.6186790	2.6420050	-1.9169790
H	5.7964610	2.5651200	-3.0727460
H	7.3096490	0.6218830	-2.7506760
H	6.6073370	-1.2503120	-1.2769670
H	4.4161680	-1.1890330	-0.1545470
H	-0.8467700	3.8529050	1.4197410
H	0.8180130	5.6100300	1.9344750
H	3.2408070	5.1693890	1.6171000
H	3.9956150	2.9674790	0.7662890
H	2.3238700	0.8869710	1.0555770

4a’

E(M062X/6-31G**) = -1874.366733 a.u.

C	-3.9519200	-0.0545070	-1.2849470
H	-3.3493560	0.5230380	-1.9804810
C	1.4528890	-0.6959910	-0.1713590
H	1.8383040	-1.6837990	0.1126260
B	2.6118190	0.3968080	-0.1554640
C	-0.0975870	1.1152770	0.5557860
C	0.3057550	-0.3238110	0.8108050
C	-1.9622830	-0.2032940	0.2233090
C	-0.9807510	-1.0874580	0.5405060
C	-1.4087420	1.1718940	0.2188940
C	0.9224900	2.1620930	0.6806120
C	2.2685260	1.8305930	0.3649070
C	3.2527250	2.8131740	0.5645680
H	4.2914050	2.5677930	0.3634830
C	2.9336380	4.0831220	1.0296270
H	3.7130450	4.8235140	1.1785860
C	1.6045440	4.3959690	1.3149370
H	1.3460530	5.3824910	1.6878300
C	0.6065760	3.4429920	1.1498690
H	-0.4200670	3.6813740	1.4082420
C	-2.1893720	2.3731120	-0.1529480
C	-1.7325500	3.2316000	-1.1602490
H	-0.7989710	3.0048010	-1.6673860
C	-2.4633900	4.3620040	-1.5114780
H	-2.0975570	5.0180290	-2.2951520
C	-3.6629280	4.6488820	-0.8630310
H	-4.2337570	5.5305120	-1.1369710
C	-3.4012890	2.6637020	0.4846330
H	-3.7704420	1.9956990	1.2576630
C	-3.3753070	-0.5093060	-0.0929880
C	-4.1295770	3.7968440	0.1353240
H	-5.0647260	4.0116910	0.6427610
C	-5.2784300	-0.3501810	-1.5833910
H	-5.7118040	0.0038360	-2.5134950
C	-6.0474800	-1.0956390	-0.6922480
H	-7.0830950	-1.3228070	-0.9248850
C	-5.4828310	-1.5480890	0.4985270
H	-6.0771400	-2.1285100	1.1972880
C	-4.1554620	-1.2565510	0.7969620
H	-3.7098340	-1.6124640	1.7217930
C	-1.0403340	-2.5613200	0.5080490

C	-1.8137080	-3.2159210	-0.4666100
H	-2.3777720	-2.6257500	-1.1810280
C	-1.8308520	-4.6017580	-0.5527610
H	-2.4313180	-5.0823690	-1.3189060
C	-1.0689700	-5.3718350	0.3256570
H	-1.0797600	-6.4549390	0.2550920
C	-0.2874960	-4.7383360	1.2865000
H	0.3138650	-5.3251620	1.9739930
C	-0.2699100	-3.3482040	1.3770990
H	0.3416940	-2.8740690	2.1366360
C	0.7394570	-0.4443840	2.2830180
C	-0.2195920	-0.2352950	3.2830680
H	-1.2429530	-0.0111990	2.9937440
C	0.1175410	-0.3138840	4.6273620
H	-0.6454200	-0.1528010	5.3826290
C	1.4296840	-0.5987330	5.0069730
H	1.6950800	-0.6617470	6.0573800
C	2.3923570	-0.7986310	4.0263840
H	3.4187540	-1.0174440	4.3038080
C	2.0499880	-0.7222550	2.6746980
H	2.8280460	-0.8983790	1.9379830
C	4.0112960	0.0121530	-0.7435220
C	4.7053540	0.8784100	-1.6057420
H	4.2738190	1.8461580	-1.8480130
C	5.9192470	0.5124600	-2.1799140
H	6.4272630	1.1938850	-2.8553980
C	6.4819580	-0.7270740	-1.8845690
H	7.4339990	-1.0113030	-2.3222350
C	5.8147910	-1.6054070	-1.0331560
H	6.2460420	-2.5753390	-0.8056380
C	4.5869130	-1.2437530	-0.4881070
H	4.0696170	-1.9504210	0.1571630
C	0.9708920	-0.8179570	-1.6108010
C	0.6806890	0.3102840	-2.3891890
H	0.8215490	1.3037140	-1.9720130
C	0.2190020	0.1789870	-3.6966810
H	-0.0034770	1.0683440	-4.2786340
C	0.0488660	-1.0844090	-4.2557370
H	-0.3118860	-1.1878590	-5.2742170
C	0.3563040	-2.2144340	-3.5013320
H	0.2343230	-3.2054870	-3.9276140
C	0.8154830	-2.0801980	-2.1944120
H	1.0401630	-2.9666840	-1.6067230

b-ts1

E(M062X/6-31G**) = -1874.298080 a.u.

B	-0.2821870	1.2074280	0.9755250
C	-0.8278830	-1.2366510	-0.1737400
C	0.6051270	-1.5572900	-0.1524280
C	1.4989570	-0.5401140	-0.0782620
C	0.9809430	0.8251840	0.1750030
C	-1.8332090	-2.3019560	0.1254130
C	-2.6940330	-2.1221090	1.2135050
C	-3.6549220	-3.0790810	1.5267310
C	-3.7706040	-4.2303530	0.7527140
C	-2.9188590	-4.4185960	-0.3340380
C	-1.9543880	-3.4650850	-0.6436500

C	1.0530230	-2.9760500	-0.2853110	C	-3.4233930	1.3133290	-0.3024180
C	1.7017820	-3.4101670	-1.4449200	C	-4.6405600	-0.2647710	-2.2408530
C	2.1300190	-4.7295320	-1.5669060	H	-2.7645840	-1.3101910	-2.3441430
C	1.9135320	-5.6332100	-0.5297180	C	-4.7446820	1.5537090	-0.6671860
C	1.2692460	-5.2097140	0.6309390	H	-2.9508370	1.9314020	0.4534530
C	0.8416810	-3.8911840	0.7515300	C	-5.3580570	0.7656170	-1.6368240
C	2.9670890	-0.7618400	-0.1945170	H	-5.1065210	-0.8789350	-3.0049560
C	3.6840160	-0.2734510	-1.2919040	H	-5.2942120	2.3604780	-0.1924060
C	5.0552290	-0.4866450	-1.3927630	H	-6.3875180	0.9554310	-1.9237050
C	5.7299170	-1.1884060	-0.3960750	C	-0.5807100	2.2723120	-1.5850270
C	5.0245090	-1.6770710	0.7003970	C	-0.7023940	1.9089690	-2.9302330
C	3.6529250	-1.4633990	0.8014920	C	-0.5972620	3.6283770	-1.2467690
C	1.9879060	1.9425150	0.3271750	C	-0.8383960	2.8810720	-3.9164400
C	2.4386250	2.7022090	-0.7597370	H	-0.6903200	0.8545800	-3.1958770
C	3.3995990	3.6928190	-0.5825980	C	-0.7286040	4.6026750	-2.2317160
C	3.9285410	3.9397540	0.6827060	H	-0.4884210	3.9142100	-0.2031120
C	3.4946880	3.1870420	1.7699250	C	-0.8498660	4.2308370	-3.5690140
C	2.5286700	2.2000160	1.5912370	H	-0.9341490	2.5862840	-4.9568640
C	-0.8322840	1.5701280	2.3687880	H	-0.7376070	5.6523830	-1.9553520
C	-0.5206870	0.7375910	3.4581340	H	-0.9541840	4.9898810	-4.3379140
C	-0.9888850	1.0223580	4.7366640	H	1.8752120	-2.7020430	-2.2502630
C	-1.7559690	2.1659320	4.9533910	H	2.6344020	-5.0500860	-2.4731860
C	-2.0635230	3.0145010	3.8912890	H	2.2459980	-6.6622060	-0.6241240
C	-1.6135310	2.7115950	2.6101420	H	1.0982280	-5.9075040	1.4446970
C	-1.2706170	0.0196470	-0.5306640	H	0.3301290	-3.5645060	1.6529520
C	-0.3957790	1.2230480	-0.5470940	H	3.1576340	0.2670180	-2.0735020
C	-2.7003940	0.2703800	-0.8882190	H	5.5970090	-0.1053760	-2.2527670
C	-3.3233100	-0.5091020	-1.8697640	H	5.5421670	-2.2261910	1.4807500

H 3.1003140 -1.8484270 1.6537400
H 6.7996980 -1.3542030 -0.4745170
H 2.0368540 2.5132040 -1.7503300
H 2.1855520 1.6136400 2.4395710
H 3.9035780 3.3689150 2.7588330
H 4.6780330 4.7132410 0.8181370
H 3.7373590 4.2718010 -1.4365020
H -1.8590770 3.3753450 1.7848480
H 0.0912270 -0.1457800 3.2890410
H -0.7493180 0.3625270 5.5644900
H -2.1130700 2.3972370 5.9522820
H -2.6559310 3.9076200 4.0628440
H -2.6066230 -1.2184740 1.8115720
H -4.3141570 -2.9219720 2.3746880
H -4.5203000 -4.9775450 0.9932650
H -3.0039440 -5.3122890 -0.9443000
H -1.2910240 -3.6216410 -1.4893010

b-int1

E(M062X/6-31G**) = -1874.298044 a.u.

B -1.0877310 -0.8121340 1.0038140
C -1.1569800 0.5055720 0.2166940
C -0.8449900 -0.9209050 -0.5023700
C 0.6245490 -1.1675070 -0.4893940
C 1.5426230 -0.2045930 -0.1568360
C 1.1695130 1.2138690 -0.1001650
C -0.1346590 1.5627340 0.0157150
C 1.0467630 -2.5591090 -0.8289400
C 2.0247860 -2.7792950 -1.8064390
C 2.4074170 -4.0700320 -2.1525640
C 1.8166170 -5.1663720 -1.5274170
C 0.8386240 -4.9598330 -0.5585980
C 0.4517590 -3.6667630 -0.2184140
C 2.9465740 -0.6003070 0.1808740
C 4.0553970 -0.2326710 -0.5891300
C 5.3370550 -0.6310370 -0.2222640
C 5.5344000 -1.3982260 0.9234730
C 4.4388600 -1.7703020 1.6975400
C 3.1568870 -1.3746300 1.3274570
C 2.2493410 2.2470660 -0.1585800
C 2.7259730 2.7297600 -1.3811310
C 3.7482410 3.6736260 -1.4230820
C 4.3085850 4.1472950 -0.2388210
C 3.8400620 3.6736380 0.9839040
C 2.8159790 2.7311290 1.0226080
C -0.5804840 2.9841990 -0.0133390
C -1.1618910 3.5536000 1.1253060
C -1.5872560 4.8776100 1.1182920
C -1.4487560 5.6497630 -0.0332750
C -0.8812010 5.0904300 -1.1748260
C -0.4487540 3.7671450 -1.1635820
C -1.3318220 -1.5012830 2.3555200
C -0.9579080 -0.8429440 3.5403800
C -1.1669860 -1.4314340 4.7831940
C -1.7832320 -2.6795800 4.8615910
C -2.1810020 -3.3417290 3.7008770
C -1.9473780 -2.7603590 2.4592560

C	-2.6034710	0.9279300	0.0779090
C	-3.0900480	1.3407170	-1.1696680
C	-3.4735430	0.9311820	1.1701660
C	-4.4082880	1.7514370	-1.3173810
H	-2.4207050	1.3336400	-2.0259350
C	-4.8007220	1.3357810	1.0225250
H	-3.1092080	0.6230220	2.1463880
C	-5.2699930	1.7465240	-0.2197670
H	-4.7667220	2.0728300	-2.2902050
H	-5.4628010	1.3329000	1.8827470
H	-6.3017130	2.0634600	-0.3356810
C	-1.6781510	-1.4720220	-1.6065550
C	-2.9754240	-1.9389160	-1.3712650
C	-1.1891750	-1.4799400	-2.9173810
C	-3.7658860	-2.4004410	-2.4192150
H	-3.3710810	-1.9207140	-0.3580370
C	-1.9744880	-1.9486390	-3.9657560
H	-0.1835660	-1.1137250	-3.1088220
C	-3.2664780	-2.4091470	-3.7196110
H	-4.7723800	-2.7544040	-2.2192490
H	-1.5789310	-1.9515110	-4.9767230
H	-3.8809750	-2.7720040	-4.5374850
H	-1.2775050	2.9490290	2.0210180
H	-0.0129390	3.3289150	-2.0562310
H	-0.7744240	5.6838870	-2.0775430
H	-2.0300790	5.3067700	2.0116780
H	-1.7845420	6.6819140	-0.0408750
H	2.4522910	2.3558390	1.9750910
H	2.2955370	2.3510650	-2.3048360
H	4.1067210	4.0397880	-2.3802440
H	5.1063720	4.8827370	-0.2693800
H	4.2712740	4.0394080	1.9105560
H	3.9138180	0.3598880	-1.4867920
H	2.3001570	-1.6689290	1.9282220
H	2.4824580	-1.9259500	-2.2979180
H	4.5799060	-2.3706420	2.5909340
H	6.5355510	-1.7053910	1.2090510
H	6.1842120	-0.3410190	-0.8360510
H	3.1659260	-4.2195480	-2.9145260
H	2.1151860	-6.1746330	-1.7964530
H	0.3730540	-5.8074200	-0.0652730
H	-0.3057270	-3.5115590	0.5420080
H	-2.6679200	-4.3094140	3.7663600
H	-1.9566440	-3.1373170	5.8307770
H	-0.8614210	-0.9175090	5.6889300
H	-0.4940200	0.1385370	3.4750970
H	-2.2567350	-3.2792190	1.5552410

b-ts2

E(M062X/6-31G**) = -1874.291860 a.u.

B	-1.0354200	-0.5974580	1.2238280
C	-1.2489260	0.6253230	0.3399920
C	-0.8352300	-1.1800620	-0.1748760
C	0.5855840	-1.2457990	-0.4463440
C	1.4821680	-0.2120580	-0.1666420
C	1.0902550	1.1760310	-0.1138270
C	-0.2384440	1.5526100	-0.0868080

C	1.0892110	-2.5381950	-1.0068700	C	-0.9943960	-2.7452390	4.4135390
C	1.9492390	-2.5560440	-2.1122030	C	-1.0474910	-2.3745680	3.0746930
C	2.4057100	-3.7582830	-2.6402990	C	-2.7015390	0.8935980	0.0938340
C	2.0079840	-4.9681110	-2.0743100	C	-3.1829220	1.0829480	-1.2089900
C	1.1462730	-4.9650740	-0.9808140	C	-3.6123970	0.8964450	1.1543240
C	0.6892620	-3.7601520	-0.4558450	C	-4.5369240	1.2834430	-1.4406290
C	2.9327950	-0.5649210	-0.0248600	H	-2.4823320	1.0561960	-2.0385210
C	3.9296220	-0.0945330	-0.8870260	C	-4.9725580	1.0985100	0.9228100
C	5.2589250	-0.4595380	-0.7034690	H	-3.2525030	0.7456250	2.1687180
C	5.6186020	-1.2985990	0.3494000	C	-5.4364520	1.2913910	-0.3735530
C	4.6369060	-1.7740430	1.2138620	H	-4.8947490	1.4266140	-2.4552860
C	3.3063580	-1.4101140	1.0254210	H	-5.6665510	1.1034390	1.7574440
C	2.1590620	2.2273190	-0.0984600	H	-6.4954030	1.4442890	-0.5564660
C	2.5140110	2.9686010	-1.2285970	C	-1.8518970	-1.8753360	-0.9917050
C	3.5265620	3.9226820	-1.1640350	C	-3.0292330	-2.3500670	-0.4001420
C	4.2031850	4.1470890	0.0317410	C	-1.7085020	-1.9831830	-2.3819750
C	3.8591520	3.4128270	1.1643670	C	-4.0318650	-2.9272770	-1.1729060
C	2.8435970	2.4642330	1.0981430	H	-3.1601410	-2.2566680	0.6759720
C	-0.6746830	2.9388810	-0.4396340	C	-2.7064030	-2.5653150	-3.1536350
C	-1.1381960	3.8052000	0.5548130	H	-0.8070670	-1.5991630	-2.8515410
C	-1.5680960	5.0898340	0.2354170	C	-3.8721100	-3.0386070	-2.5517540
C	-1.5540180	5.5219150	-1.0883650	H	-4.9388350	-3.2876440	-0.6979310
C	-1.1079890	4.6630560	-2.0899600	H	-2.5794290	-2.6433600	-4.2289160
C	-0.6696640	3.3823360	-1.7659760	H	-4.6539080	-3.4869200	-3.1566970
C	-1.0118720	-1.0215630	2.6981010	H	-1.1569310	3.4658070	1.5867910
C	-0.9137440	-0.0486800	3.7077470	H	-0.3327460	2.7070710	-2.5480030
C	-0.8486230	-0.4132550	5.0478490	H	-1.1026330	4.9896260	-3.1252880
C	-0.8913540	-1.7624790	5.3980260	H	-1.9174820	5.7526180	1.0209580

H	-1.8928110	6.5220780	-1.3395060
H	2.5812160	1.8837650	1.9787140
H	2.0047630	2.7845240	-2.1691790
H	3.7890630	4.4886190	-2.0526610
H	4.9947590	4.8882720	0.0803780
H	4.3825030	3.5776080	2.1011080
H	3.6593690	0.5563250	-1.7125360
H	2.5373610	-1.7835690	1.6971070
H	2.2527030	-1.6149450	-2.5603440
H	4.9045180	-2.4301310	2.0363960
H	6.6570510	-1.5806640	0.4924480
H	6.0163820	-0.0880430	-1.3866960
H	3.0708740	-3.7507780	-3.4982000
H	2.3659560	-5.9068980	-2.4850110
H	0.8287790	-5.9021450	-0.5341380
H	0.0170630	-3.7607710	0.3970520
H	-1.0288910	-3.7933260	4.6929290
H	-0.8441540	-2.0497460	6.4441500
H	-0.7659140	0.3463580	5.8185600
H	-0.8794380	1.0015470	3.4274050
H	-1.1269580	-3.1374110	2.3042360

3a’

E(M062X/6-31G**) = -1874.322052 a.u.

B	-1.0067210	0.2314070	1.1102460
C	0.9500380	-0.9592960	-0.2854410
C	1.3293490	0.3566190	-0.2837060
C	0.4055070	1.5143610	-0.4333660
C	-0.7681620	1.5213690	0.2525930
C	1.9277430	-2.0204780	0.1357230
C	2.3714740	-3.0562610	-0.6941870
C	3.2531370	-4.0206740	-0.2114480
C	3.6979390	-3.9745920	1.1065670
C	3.2599020	-2.9499320	1.9421150
C	2.3839170	-1.9832060	1.4607750
C	2.7663920	0.7323230	-0.0997710
C	3.7812240	0.1802710	-0.8877990
C	5.1062710	0.5608640	-0.7046550
C	5.4373310	1.4999910	0.2698740
C	4.4335620	2.0632300	1.0538500
C	3.1073700	1.6883580	0.8626350
C	0.8814930	2.6388250	-1.2890300
C	1.4175490	2.3815700	-2.5552080
C	1.8580310	3.4234410	-3.3664030
C	1.7810500	4.7389980	-2.9164730
C	1.2665030	5.0052580	-1.6486990
C	0.8238520	3.9636460	-0.8410380
C	-1.8696260	2.5039470	0.1143540
C	-2.2387570	3.0453290	-1.1271070
C	-3.3106980	3.9227050	-1.2255780
C	-4.0423280	4.2737520	-0.0907310
C	-3.7020050	3.7281180	1.1428310
C	-2.6324630	2.8420340	1.2416160
C	-0.6290710	0.0738040	2.6091730
C	-0.0873360	1.1484180	3.3350730
C	0.3367440	0.9881230	4.6500520
C	0.2253350	-0.2594840	5.2634180

C	-0.3070520	-1.3423920	4.5644380
C	-0.7270360	-1.1737030	3.2491500
C	-0.4373920	-1.4586480	-0.5256530
C	-1.4741970	-0.9329750	0.1715060
C	-2.9104940	-1.2569340	0.0072900
C	-3.4882440	-1.5363490	-1.2408410
C	-3.7476040	-1.2133340	1.1321650
C	-4.8492210	-1.7947420	-1.3490270
H	-2.8665110	-1.5368180	-2.1296030
C	-5.1089330	-1.4814410	1.0238840
H	-3.3224140	-0.9730860	2.1033370
C	-5.6641510	-1.7765540	-0.2176280
H	-5.2779730	-2.0052770	-2.3238810
H	-5.7356910	-1.4516150	1.9096290
H	-6.7264480	-1.9805690	-0.3068680
C	-0.5776900	-2.5803020	-1.4987520
C	-1.0694040	-3.8265950	-1.1010670
C	-0.2081800	-2.3908160	-2.8351670
C	-1.1927050	-4.8623060	-2.0224660
H	-1.3497570	-3.9772050	-0.0624770
C	-0.3370440	-3.4246810	-3.7585830
H	0.1789260	-1.4235270	-3.1457640
C	-0.8276810	-4.6643600	-3.3524980
H	-1.5704080	-5.8275660	-1.7001890
H	-0.0532750	-3.2636340	-4.7940500
H	-0.9240150	-5.4729180	-4.0701620
H	-1.1223760	-2.0220570	2.6943010
H	-0.3854960	-2.3132730	5.0431560
H	0.5575540	-0.3886730	6.2891610
H	0.7560820	1.8271780	5.1961080
H	0.0051600	2.1186150	2.8504410
H	2.0429580	-3.1043330	-1.7266380
H	3.5936010	-4.8106240	-0.8738470
H	4.3831430	-4.7295610	1.4790510
H	3.5998910	-2.8998100	2.9718860
H	2.0474010	-1.1799030	2.1111620
H	3.5244280	-0.5510470	-1.6485230
H	5.8818220	0.1249300	-1.3265300
H	6.4720560	1.7950110	0.4132450
H	4.6824230	2.7985550	1.8126740
H	2.3205900	2.1314870	1.4680910
H	1.4853860	1.3540950	-2.9034430
H	2.2654960	3.2064040	-4.3489130
H	0.4266740	4.1705240	0.1487860
H	1.2157990	6.0269920	-1.2855900
H	2.1279840	5.5522340	-3.5460760
H	-1.6819730	2.7671670	-2.0159260
H	-2.3787310	2.4109160	2.2068430
H	-4.2731690	3.9835380	2.0298100
H	-4.8790470	4.9602410	-0.1724450
H	-3.5827770	4.3295130	-2.1945130

***I*Me’**

E(M062X/6-31G**) = -304.677749 a.u.

C	0.6767290	-1.2052460	0.0000870
C	-0.6767290	-1.2052460	-0.0000500
C	0.0000000	0.9902410	-0.0000930

H	1.3873300	-2.0178670	0.0001790
H	-1.3873300	-2.0178670	-0.0000850
N	1.0559530	0.1261900	0.0000090
N	-1.0559530	0.1261900	-0.0000370
C	2.4393580	0.5596560	0.0000810
H	2.9586440	0.1942060	-0.8899190
H	2.4458160	1.6483990	-0.0002460
H	2.9583970	0.1947480	0.8904500
C	-2.4393580	0.5596560	-0.0000370
H	-2.9585020	0.1945620	0.8901950
H	-2.4458160	1.6483990	-0.0001380
H	-2.9585390	0.1943920	-0.8901750

2a(*I*Me)'

E(M062X/6-31G**) = -2179.063881 a.u.

B	0.2603510	-0.5067260	1.2429800
C	1.1377720	-0.2285950	-0.2263590
C	0.8996740	1.2875160	-0.3796150
C	-0.3782590	1.5618040	-0.0289500
C	-1.0543890	0.2839180	0.5309300
C	2.5659240	-0.6973220	-0.1844250
C	3.0079220	-1.9250830	-0.7006900
C	4.3196210	-2.3623360	-0.5117580
C	5.2333010	-1.5870550	0.1932200
C	4.8186060	-0.3574780	0.7046200
C	3.5119560	0.0744890	0.5170320
C	1.8483540	2.2554560	-0.9826860
C	2.8398820	1.8646030	-1.8971700
C	3.7109670	2.7917300	-2.4622790
C	3.6313730	4.1374050	-2.1176480
C	2.6701560	4.5435770	-1.1944350
C	1.7985550	3.6173880	-0.6345350
C	-1.1498060	2.7589670	-0.4525130
C	-1.1608900	3.0792040	-1.8204800
C	-1.8873980	4.1617310	-2.3027020
C	-2.6254420	4.9549800	-1.4268110
C	-2.6378280	4.6442600	-0.0703900
C	-1.9158620	3.5548520	0.4110140
C	-2.3651130	0.5358460	1.2465050
C	-3.5208220	0.9213810	0.5465470
C	-4.7123710	1.1961590	1.2077240
C	-4.7850890	1.1044260	2.5967650
C	-3.6498200	0.7431960	3.3129390
C	-2.4608010	0.4627590	2.6395690
C	0.1055700	-0.9021880	-1.1651880
C	-1.1352890	-0.6464840	-0.7115860
C	0.3976270	-1.6566820	-2.4090390
C	1.1964060	-1.0900390	-3.4105700
C	-0.1329010	-2.9363640	-2.6307300
C	1.4761630	-1.7841510	-4.5836860
H	1.5789940	-0.0841100	-3.2745590
C	0.1486110	-3.6318010	-3.8029150
H	-0.7754600	-3.3815500	-1.8766740
C	0.9604900	-3.0628890	-4.7814760
H	2.0956050	-1.3220200	-5.3463330
H	-0.2711800	-4.6220970	-3.9512140
H	1.1817340	-3.6078110	-5.6938090

C	-2.3848860	-1.1863950	-1.2896720
C	-2.7674440	-0.8943240	-2.6038900
C	-3.1990600	-2.0274000	-0.5189960
C	-3.9368000	-1.4300480	-3.1364540
H	-2.1382270	-0.2446040	-3.2058390
C	-4.3651910	-2.5647760	-1.0544710
H	-2.9073640	-2.2551100	0.5035220
C	-4.7390070	-2.2667400	-2.3637950
H	-4.2208970	-1.1929670	-4.1572660
H	-4.9840920	-3.2176960	-0.4466400
H	-5.6509970	-2.6841540	-2.7793170
H	2.9488320	0.8188550	-2.1568990
H	4.4610960	2.4528510	-3.1701780
H	4.3132010	4.8592410	-2.5559120
H	2.5976860	5.5877930	-0.9057580
H	1.0505000	3.9598790	0.0723900
H	-0.5857310	2.4616920	-2.5046640
H	-1.8780550	4.3841740	-3.3652200
H	-3.2211860	5.2446450	0.6208980
H	-1.9776480	3.3186570	1.4679100
H	-3.1936390	5.8012570	-1.7999000
H	-3.4818020	1.0190740	-0.5337220
H	-1.5801890	0.1995910	3.2153120
H	-3.6838820	0.6737120	4.3961100
H	-5.7158320	1.3177230	3.1133260
H	-5.5860460	1.4891750	0.6337340
H	2.3302190	-2.5599060	-1.2583490
H	4.6216980	-3.3205730	-0.9237160
H	6.2533990	-1.9289370	0.3370770
H	5.5154490	0.2708630	1.2521820
H	3.2089430	1.0335430	0.9303330
C	2.7566400	0.2860960	4.0525830
C	2.1243340	1.4806010	4.0251070
C	1.1235170	0.1487400	2.5039780
H	3.5874840	-0.0665690	4.6422690
H	2.2832540	2.3874880	4.5858250
N	2.1348010	-0.5164300	3.1256300
N	1.1289700	1.3768580	3.0792880
C	2.5975310	-1.8885880	2.9078130
H	2.0261020	-2.5797110	3.5299220
H	2.4903390	-2.1652240	1.8635450
H	3.6539510	-1.9220740	3.1754640
C	0.2524570	2.5031910	2.7926090
H	0.6862840	3.1354190	2.0173570
H	-0.7126230	2.1302480	2.4702810
H	0.1253260	3.0750780	3.7132680
C	-0.0839230	-2.0433540	1.6858490
C	-0.6001980	-2.3146310	2.9659950
C	0.1079490	-3.1697700	0.8681930
C	-0.9436460	-3.5990130	3.3876010
H	-0.7193860	-1.5050840	3.6816100
C	-0.2198310	-4.4617000	1.2740940
H	0.5446240	-3.0432720	-0.1141540
C	-0.7621700	-4.6849390	2.5366600
H	-1.3484460	-3.7482440	4.3847180
H	-0.0459030	-5.2951940	0.5989320

H -1.0270210 -5.6881960 2.8562210

c-ts1

E(M062X/6-31G**) = -2179.016476 a.u.

99

symmetry c1

B	-0.6684580	-0.3934320	1.5044130
C	-0.9934740	0.6288020	0.3240810
C	-1.0781740	-0.4316290	-0.8119630
C	0.1219510	-1.0903920	-1.0608560
C	1.3725410	-0.5675840	-0.5502380
C	-2.2606100	1.4347940	0.5729520
C	-2.7666220	1.6146580	1.8637250
C	-3.8753810	2.4262480	2.1082010
C	-4.5039510	3.0795560	1.0551210
C	-4.0098280	2.9191520	-0.2390910
C	-2.9027920	2.1141900	-0.4735320
C	-2.2824560	-0.5969300	-1.6699690
C	-2.1499680	-0.5680750	-3.0697960
C	-3.2488100	-0.7032720	-3.9107600
C	-4.5273210	-0.8510400	-3.3778660
C	-4.6877780	-0.8480110	-1.9950650
C	-3.5853020	-0.7180500	-1.1560530
C	0.1375520	-2.2393610	-2.0177440
C	0.8690700	-2.1381180	-3.2090090
C	0.9006470	-3.1890230	-4.1169330
C	0.2111880	-4.3712360	-3.8432580
C	-0.5124770	-4.4866370	-2.6605560
C	-0.5531800	-3.4247110	-1.7582390
C	2.5664960	-1.3734380	-0.2391770
C	3.5195120	-0.8609710	0.6763760
C	4.6434900	-1.5709440	1.0760320
C	4.8818250	-2.8585380	0.5975180
C	3.9573570	-3.4011400	-0.2918530
C	2.8344750	-2.6872360	-0.6989150
C	0.1857530	1.5308760	-0.1223980
C	1.3715760	0.8747140	-0.3821240
C	-0.0120130	2.9524390	-0.5307730
C	0.3828390	3.3522070	-1.8191510
C	-0.5728690	3.9398720	0.2972060
C	0.2629460	4.6697850	-2.2467540
H	0.7926560	2.6023170	-2.4893820
C	-0.6990420	5.2589800	-0.1272250
H	-0.9436790	3.6786270	1.2838290
C	-0.2752810	5.6350200	-1.3991790
H	0.5828600	4.9402110	-3.2486150
H	-1.1367900	5.9947230	0.5410600
H	-0.3740440	6.6645910	-1.7289090
C	2.6223970	1.6467950	-0.6599140
C	3.4816100	1.2780780	-1.7027610
C	2.9784070	2.7540820	0.1212340
C	4.6454490	1.9947030	-1.9615820
H	3.2279220	0.4134200	-2.3089010
C	4.1405720	3.4740730	-0.1343300
H	2.3180280	3.0587600	0.9281690
C	4.9819790	3.0968290	-1.1786630
H	5.2924420	1.6900790	-2.7788010

H	4.3903960	4.3321540	0.4828380
H	5.8905060	3.6557770	-1.3803430
H	-1.1654390	-0.4202430	-3.4998130
H	-3.1048200	-0.6783200	-4.9867630
H	-5.3879910	-0.9529900	-4.0314520
H	-5.6785400	-0.9399930	-1.5603210
H	-3.7426450	-0.6915600	-0.0829610
H	1.4172400	-1.2204640	-3.4072820
H	1.4664120	-3.0902520	-5.0383960
H	-1.0494870	-5.4036700	-2.4379040
H	-1.1104930	-3.5234550	-0.8315180
H	0.2398850	-5.1954120	-4.5491470
H	3.3500980	0.1183750	1.1124050
H	2.1588120	-3.1818880	-1.3800940
H	4.1053090	-4.4037660	-0.6841730
H	5.7578900	-3.4194330	0.9067470
H	5.3314550	-1.1136040	1.7828370
H	-2.2943930	1.1179500	2.7077150
H	-4.2430790	2.5414470	3.1233220
H	-5.3687380	3.7097390	1.2382780
H	-4.4841510	3.4296310	-1.0713500
H	-2.5192120	2.0164190	-1.4841370
C	2.3593080	-1.1202940	3.6381560
C	2.2319180	0.2128360	3.8612550
C	0.6838150	-0.3821980	2.3651490
H	3.0696970	-1.8403450	4.0122610
H	2.7826650	0.8865800	4.4978760
N	1.3912630	-1.4671800	2.7295490
N	1.1993940	0.6494040	3.0620760
C	1.2010730	-2.8241750	2.2238370
H	0.6007240	-3.3984390	2.9328390
H	0.7012460	-2.7788950	1.2567860
H	2.1843470	-3.2771980	2.0871330
C	0.6569590	2.0013990	3.1086650
H	0.0572300	2.1609400	2.2164790
H	0.0523400	2.1294380	4.0098800
H	1.4822710	2.7142950	3.1156760
C	-1.6707110	-1.4400260	2.1318820
C	-1.7666300	-1.4832930	3.5377300
C	-2.4548670	-2.3548040	1.4137850
C	-2.6340000	-2.3539790	4.1885040
H	-1.1552120	-0.8139850	4.1426750
C	-3.3093200	-3.2461310	2.0590460
H	-2.3835150	-2.3795710	0.3338700
C	-3.4089330	-3.2439050	3.4470950
H	-2.6955500	-2.3478560	5.2723590
H	-3.8958800	-3.9454180	1.4711980
H	-4.0765570	-3.9367840	3.9496520

c-int1

E(M062X/6-31G**) = -2179.054419 a.u.

B	-1.1502280	-0.3281840	1.1089630
C	-0.8779370	0.9097490	0.1253470
C	-0.9822530	-0.5883690	-0.5039430
C	0.2876250	-1.2586280	-0.8676200
C	1.5003850	-0.7337470	-0.4942370
C	-2.0050690	1.8303520	-0.2885320

C	-2.9983760	2.2323600	0.6028350
C	-4.0160080	3.1031260	0.2078980
C	-4.0558380	3.5859330	-1.0937680
C	-3.0659690	3.1976650	-1.9983150
C	-2.0563660	2.3355770	-1.5964990
C	-2.0958960	-0.7022260	-1.5217990
C	-1.7586620	-0.6718300	-2.8846160
C	-2.7249110	-0.6776990	-3.8831120
C	-4.0766110	-0.6887920	-3.5472710
C	-4.4345080	-0.6747860	-2.2039500
C	-3.4599060	-0.6766480	-1.2081270
C	0.1500510	-2.5540830	-1.6026580
C	0.8279180	-2.7422870	-2.8148350
C	0.6470820	-3.8915900	-3.5776440
C	-0.2350410	-4.8804240	-3.1500400
C	-0.9479510	-4.6944330	-1.9685480
C	-0.7666720	-3.5392870	-1.2153570
C	2.7097870	-1.5577970	-0.1558230
C	3.2896280	-1.2928510	1.0997110
C	4.3631710	-2.0247890	1.5889070
C	4.9126670	-3.0541120	0.8272580
C	4.3738620	-3.3190400	-0.4258020
C	3.2943270	-2.5822440	-0.9131670
C	0.4828990	1.5046870	-0.0866580
C	1.5922540	0.7187410	-0.2320410
C	0.5887690	2.9892890	-0.2904120
C	1.2169940	3.4855270	-1.4418970
C	0.0293090	3.9211740	0.5929460
C	1.3107390	4.8506100	-1.6860230
H	1.6321610	2.7819690	-2.1566990
C	0.1244590	5.2894210	0.3557860
H	-0.5147830	3.5826220	1.4670380
C	0.7698200	5.7623500	-0.7827490
H	1.8014730	5.2016480	-2.5885930
H	-0.3192650	5.9863600	1.0601630
H	0.8388710	6.8290810	-0.9712670
C	2.9572610	1.3220320	-0.3495100
C	3.7937450	0.9903260	-1.4232570
C	3.4393250	2.2268430	0.6021140
C	5.0590190	1.5538270	-1.5478540
H	3.4393060	0.2866690	-2.1715620
C	4.7053670	2.7909480	0.4840730
H	2.8023660	2.5030280	1.4373710
C	5.5218600	2.4569570	-0.5933290
H	5.6849470	1.2871050	-2.3939550
H	5.0543870	3.4929850	1.2352130
H	6.5099540	2.8959660	-0.6885260
H	-0.7115350	-0.6150940	-3.1661630
H	-2.4196760	-0.6574330	-4.9251740
H	-4.8375680	-0.6880010	-4.3215550
H	-5.4820870	-0.6500940	-1.9189250
H	-3.7768500	-0.6144520	-0.1762620
H	1.4791370	-1.9510420	-3.1769640
H	1.1836550	-4.0059780	-4.5144550
H	-1.6606260	-5.4436150	-1.6380640
H	-1.3601840	-3.3863150	-0.3217120

H	-0.3828810	-5.7772510	-3.7432040
H	2.8807460	-0.4855830	1.7005070
H	2.9281790	-2.8141090	-1.9015820
H	4.7979040	-4.1028940	-1.0460860
H	5.7538520	-3.6300040	1.1997190
H	4.7717800	-1.7849190	2.5663410
H	-2.9852890	1.8557820	1.6189740
H	-4.7775640	3.3984290	0.9236310
H	-4.8482070	4.2594690	-1.4056560
H	-3.0840270	3.5679520	-3.0187980
H	-1.2886390	2.0369350	-2.3049810
C	1.3624270	-1.7524740	3.6308170
C	1.3668620	-0.4410630	3.9658000
C	0.0647570	-0.6750750	2.1422050
H	1.8609660	-2.6028160	4.0672780
H	1.8580990	0.0917690	4.7639860
N	0.5596530	-1.8734910	2.5192870
N	0.5685710	0.2011470	3.0432480
C	0.2843940	-3.1407790	1.8662350
H	-0.7855020	-3.2100530	1.6715700
H	0.8514770	-3.2194480	0.9370090
H	0.5768380	-3.9445970	2.5417040
C	0.2766970	1.6237880	3.1169150
H	-0.5869230	1.8280160	2.4896400
H	0.0534000	1.8865030	4.1526520
H	1.1301130	2.2050890	2.7633570
C	-2.4596680	-0.7476670	1.9743640
C	-2.8172060	-0.0929410	3.1673110
C	-3.1521760	-1.9405800	1.7038860
C	-3.8206450	-0.5650400	4.0090650
H	-2.2962490	0.8179940	3.4560230
C	-4.1577350	-2.4297020	2.5371660
H	-2.9148870	-2.4900290	0.7957020
C	-4.5009110	-1.7395240	3.6954300
H	-4.0684310	-0.0166910	4.9134890
H	-4.6730730	-3.3502160	2.2784870
H	-5.2846710	-2.1120540	4.3477270

c-ts2

E(M062X/6-31G**) = -2179.043774 a.u.

B	-1.1004890	-0.4304530	1.0741030
C	-1.0379550	0.9830250	0.3083500
C	-0.9412750	-1.0208590	-0.4329910
C	0.3684450	-1.2679880	-0.8932710
C	1.5068900	-0.5456780	-0.3993810
C	-2.3009820	1.6279500	-0.1715560
C	-3.4001530	1.8533860	0.6636280
C	-4.5800260	2.4082930	0.1708340
C	-4.6952950	2.7279690	-1.1772240
C	-3.6134730	2.5028630	-2.0281700
C	-2.4341390	1.9691610	-1.5280910
C	-2.1014070	-1.1730080	-1.3755080
C	-1.9190520	-0.9750860	-2.7592330
C	-2.9765210	-1.0005210	-3.6581290
C	-4.2798280	-1.1920570	-3.2053940
C	-4.4970820	-1.3311330	-1.8400470
C	-3.4302410	-1.3147390	-0.9443060

C	0.5950370	-2.2677110	-1.9819160
C	1.2962190	-1.9093580	-3.1440720
C	1.4754190	-2.8135240	-4.1839340
C	0.9581250	-4.1052940	-4.0824010
C	0.2537830	-4.4744950	-2.9405150
C	0.0709950	-3.5618510	-1.9039030
C	2.8062070	-1.2533840	-0.1009630
C	3.3755550	-0.9785370	1.1578300
C	4.5174460	-1.6218410	1.6156460
C	5.1482380	-2.5775210	0.8229880
C	4.6124650	-2.8628650	-0.4268990
C	3.4632130	-2.2173220	-0.8833830
C	0.1903460	1.5648710	-0.0818360
C	1.4200660	0.8424840	-0.1208390
C	0.2141100	2.9992890	-0.5206600
C	0.8451950	3.3770580	-1.7127710
C	-0.3873890	4.0018860	0.2493570
C	0.8752800	4.7062910	-2.1201380
H	1.3133530	2.6107860	-2.3249170
C	-0.3602830	5.3333480	-0.1533020
H	-0.8941580	3.7298230	1.1717890
C	0.2735730	5.6917630	-1.3403920
H	1.3679460	4.9729330	-3.0502200
H	-0.8348080	6.0918840	0.4618110
H	0.2970440	6.7299480	-1.6563840
C	2.6914610	1.6359750	-0.0506380
C	3.7182480	1.4616210	-0.9873180
C	2.8712600	2.6035340	0.9442050
C	4.8862070	2.2128100	-0.9212210
H	3.5939370	0.7265670	-1.7773470
C	4.0391730	3.3560160	1.0176830
H	2.0681940	2.7797820	1.6533020
C	5.0550350	3.1612870	0.0857250
H	5.6650560	2.0609310	-1.6621850
H	4.1524680	4.1005290	1.7998680
H	5.9669070	3.7477250	0.1378840
H	-0.9287530	-0.7607530	-3.1389200
H	-2.7806330	-0.8458950	-4.7150050
H	-5.1115000	-1.2038950	-3.9030730
H	-5.5067130	-1.4400430	-1.4557340
H	-3.6565170	-1.3771530	0.1087450
H	1.6909530	-0.8988420	-3.2230550
H	2.0151800	-2.5115870	-5.0762880
H	-0.1598880	-5.4746200	-2.8561710
H	-0.4996130	-3.8511460	-1.0263590
H	1.0967370	-4.8144220	-4.8922470
H	2.9015940	-0.2359790	1.7911920
H	3.1034180	-2.4734730	-1.8680580
H	5.0910890	-3.5953630	-1.0699710
H	6.0421480	-3.0849500	1.1715220
H	4.9103740	-1.3730550	2.5972560
H	-3.3396240	1.5973320	1.7123570
H	-5.4131630	2.5796600	0.8457250
H	-5.6193330	3.1455960	-1.5650630
H	-3.6907300	2.7390190	-3.0850220
H	-1.5979360	1.7896610	-2.1965950

C	1.4658060	-1.8820950	3.5359030
C	1.3962120	-0.6104160	3.9865710
C	0.1532480	-0.7346880	2.1094090
H	2.0009050	-2.7427530	3.9039610
H	1.8408300	-0.1276270	4.8421580
N	0.7007210	-1.9391360	2.3904430
N	0.5936630	0.0798090	3.0984560
C	0.5536730	-3.1637440	1.6184540
H	-0.3940050	-3.1393000	1.0902030
H	1.3733070	-3.2568900	0.9001480
H	0.5633350	-4.0068950	2.3106390
C	0.2266960	1.4680450	3.3413860
H	-0.3187600	1.8528100	2.4836500
H	-0.3918140	1.5290610	4.2398030
H	1.1349920	2.0537540	3.4920600
C	-2.2968710	-0.9439450	2.0757090
C	-2.7040670	-0.2010630	3.1973810
C	-2.7634100	-2.2699640	2.0295690
C	-3.5476340	-0.7177390	4.1783970
H	-2.3510060	0.8204570	3.3146730
C	-3.6040760	-2.8064500	3.0027210
H	-2.4877030	-2.8982330	1.1847870
C	-4.0054510	-2.0290660	4.0854020
H	-3.8433410	-0.0955470	5.0184630
H	-3.9469280	-3.8332070	2.9118220
H	-4.6632100	-2.4389830	4.8455240

3a(*I*Me)'

E(M062X/6-31G**) = -2179.073574 a.u.

B	-1.1867500	-0.0497360	0.9495040
C	-0.8465680	1.2499720	-0.0086530
C	0.4133340	1.4991990	-0.4761250
C	1.5762100	0.6431850	-0.1589380
C	1.5367280	-0.7260000	-0.1584030
C	0.3419090	-1.4835650	-0.6080690
C	-0.9164860	-1.2309460	-0.1580700
C	-0.1622880	-0.1642350	2.2311010
N	0.2784920	-1.2850050	2.8466480
C	0.9217690	-0.9772680	4.0279500
H	1.3693750	-1.7391360	4.6459310
C	0.8737670	0.3679200	4.1596790
H	1.2488490	1.0240560	4.9285570
N	0.2061990	0.8488120	3.0505010
C	0.0946980	-2.6630380	2.4042710
H	-0.8975110	-2.7781820	1.9734610
H	0.1861620	-3.3053960	3.2804340
H	0.8519540	-2.9338110	1.6658290
C	-0.2574960	2.2290510	2.9641010
H	-0.2388190	2.5616730	1.9290040
H	0.4049280	2.8530150	3.5654810
H	-1.2766970	2.2947320	3.3549580
C	-2.6873370	-0.0629190	1.6152250
C	-2.9088200	-0.2020490	2.9958950
H	-2.0662160	-0.2994120	3.6796930
C	-4.1885890	-0.2286490	3.5562980
H	-4.3042770	-0.3373830	4.6312060
C	-5.3073760	-0.1197640	2.7401350

H	-6.3057170	-0.1394220	3.1670320
C	-5.1281000	0.0128860	1.3635880
H	-5.9893350	0.0951750	0.7068460
C	-3.8480300	0.0385540	0.8215830
H	-3.7491870	0.1376580	-0.2559860
C	-1.9704310	2.0852840	-0.5240690
C	-2.7757250	2.8415660	0.3367240
H	-2.5862280	2.8128370	1.4056040
C	-3.8257020	3.6108520	-0.1547120
H	-4.4306120	4.1958210	0.5314950
C	-4.1139450	3.6134610	-1.5175310
H	-4.9410090	4.2032110	-1.9003960
C	-3.3367460	2.8481350	-2.3840980
H	-3.5561040	2.8376390	-3.4473600
C	-2.2725800	2.0986760	-1.8921070
H	-1.6563180	1.5160770	-2.5709210
C	0.7311060	2.6682410	-1.3615960
C	0.3492580	3.9753860	-1.0379550
H	-0.2141590	4.1546780	-0.1264860
C	0.6693670	5.0418420	-1.8712930
H	0.3629450	6.0473370	-1.5993570
C	1.3817180	4.8229200	-3.0485630
H	1.6308420	5.6549890	-3.6996440
C	1.7769910	3.5295520	-3.3796310
H	2.3366440	3.3473030	-4.2920670
C	1.4586730	2.4663080	-2.5399490
H	1.7782620	1.4587390	-2.7954010
C	2.8594540	1.3741850	0.0925190
C	2.8892200	2.4523870	0.9830150
H	1.9713450	2.7469000	1.4845590
C	4.0694680	3.1482260	1.2273390
H	4.0721420	3.9783010	1.9273310
C	5.2411550	2.7870340	0.5674510
H	6.1620280	3.3317190	0.7504240
C	5.2210190	1.7274730	-0.3375200
H	6.1261180	1.4471270	-0.8673630
C	4.0414690	1.0303850	-0.5735080
H	4.0288000	0.2064150	-1.2814830
C	2.6956890	-1.5146430	0.3909560
C	3.1634310	-1.1578700	1.6679950
H	2.7235690	-0.2977980	2.1628300
C	4.1753870	-1.8667310	2.3020920
H	4.5060630	-1.5583760	3.2896760
C	4.7629930	-2.9613470	1.6711010
H	5.5556460	-3.5190140	2.1598760
C	4.3258010	-3.3220680	0.4015640
H	4.7823370	-4.1625920	-0.1121490
C	3.3047080	-2.6145210	-0.2325230
H	3.0066350	-2.9187610	-1.2270500
C	0.6162050	-2.5316460	-1.6402080
C	0.2761780	-3.8723800	-1.4352380
H	-0.2185800	-4.1569220	-0.5107430
C	0.5589870	-4.8333320	-2.4009770
H	0.2917430	-5.8705580	-2.2236170
C	1.1841050	-4.4677990	-3.5916450
H	1.4040470	-5.2174910	-4.3451250

C	1.5204630	-3.1333110	-3.8118790
H	2.0013210	-2.8389830	-4.7398250
C	1.2421290	-2.1756600	-2.8408190
H	1.5138750	-1.1353340	-3.0038010
C	-2.0652830	-1.9229820	-0.8135910
C	-2.9576250	-2.7243250	-0.0898260
H	-2.8313130	-2.8362700	0.9833770
C	-4.0277820	-3.3551980	-0.7174500
H	-4.7011470	-3.9764740	-0.1347020
C	-4.2475680	-3.1738070	-2.0805890
H	-5.0892480	-3.6559190	-2.5679580
C	-3.3839660	-2.3607000	-2.8109610
H	-3.5491840	-2.2044230	-3.8726580
C	-2.3033100	-1.7486100	-2.1839990
H	-1.6228920	-1.1256210	-2.7576030

2c’

E(M062X/6-31G**) = -2103.011218 a.u.

B	0.0953240	-0.6782290	1.2952930
C	1.2758900	0.1496810	0.5388600
C	0.6602860	1.4594490	0.0902730
C	-0.6850010	1.4073130	0.1355700
C	-1.1694320	0.0573160	0.6276490
C	2.6327470	0.1578420	1.1907950
C	3.4948430	-0.9443980	1.2610190
C	4.7010250	-0.8664640	1.9553620
C	5.0718380	0.3029600	2.6086350
C	4.2093470	1.3956870	2.5765210
C	3.0071460	1.3198910	1.8841630
C	1.4678080	2.5511150	-0.4999220
C	2.6262370	2.2675400	-1.2349680
C	3.3781420	3.2890170	-1.8060890
C	2.9948180	4.6173180	-1.6405430
C	1.8573610	4.9145020	-0.8925370
C	1.1034250	3.8939430	-0.3240930
C	-1.6372350	2.3792470	-0.4568490
C	-1.5788080	2.6702140	-1.8252430
C	-2.4750600	3.5673000	-2.3979650
C	-3.4427950	4.1868580	-1.6104070
C	-3.5115570	3.9018980	-0.2486400
C	-2.6186200	3.0011210	0.3243850
C	-2.5791600	-0.0478040	1.1243680
C	-3.6563410	-0.0995970	0.2326300
C	-4.9639600	-0.1255230	0.7044540
C	-5.2132380	-0.0951870	2.0757820
C	-4.1490090	-0.0350810	2.9708000
C	-2.8383210	-0.0104410	2.4965390
C	0.7675290	-1.0034830	-0.2987430
C	-0.6312980	-1.0545620	-0.2509730
C	1.5888390	-2.0934470	-0.8835620
C	2.5389990	-1.7687430	-1.8549380
C	1.4363000	-3.4246460	-0.4825870
C	3.3327880	-2.7629480	-2.4180340
H	2.6508090	-0.7353450	-2.1685580
C	2.2417260	-4.4135900	-1.0380120
H	0.6964090	-3.6794690	0.2701340
C	3.1899340	-4.0856190	-2.0052170

H	4.0663400	-2.5026880	-3.1740860
H	2.1262220	-5.4430920	-0.7151500
H	3.8156760	-4.8601670	-2.4367340
C	-1.4292890	-2.1893350	-0.7761070
C	-1.3836810	-2.4619270	-2.1466550
C	-2.2216110	-2.9828440	0.0604490
C	-2.1222860	-3.5152110	-2.6759370
H	-0.7633370	-1.8468730	-2.7921650
C	-2.9507750	-4.0407940	-0.4735850
H	-2.2663770	-2.7734940	1.1249260
C	-2.9052430	-4.3077520	-1.8400580
H	-2.0822850	-3.7181820	-3.7411780
H	-3.5583120	-4.6556800	0.1823630
H	-3.4787970	-5.1317250	-2.2523550
H	2.9487770	1.2369860	-1.3463530
H	4.2697190	3.0453220	-2.3753410
H	3.5832450	5.4154530	-2.0817210
H	1.5579790	5.9472540	-0.7437680
H	0.2258740	4.1348790	0.2674440
H	-0.8195810	2.1898140	-2.4357270
H	-2.4167580	3.7828600	-3.4602850
H	-4.2643460	4.3793330	0.3706190
H	-2.6803120	2.7745270	1.3848380
H	-4.1416630	4.8875050	-2.0563990
H	-3.4632750	-0.1123890	-0.8368220
H	-2.0075680	0.0475920	3.1942530
H	-4.3357280	-0.0055410	4.0396440
H	-6.2342270	-0.1141780	2.4439120
H	-5.7892030	-0.1645260	0.0005850
H	3.2378460	-1.8825780	0.7884340
H	5.3483400	-1.7374400	1.9850180
H	6.0131470	0.3593870	3.1458650
H	4.4673310	2.3126860	3.0969300
H	2.3434960	2.1779720	1.8854790
Cl	0.2345940	-1.8177610	2.6647170

e-ts1

E(M062X/6-31G**) = -2102.962518 a.u.

B	-0.7773800	-1.1153190	1.5788110
C	-1.4250630	-0.0786490	0.6938490
C	-0.2387830	-1.6032110	0.2680160
C	1.0820400	-1.0097930	0.1023760
C	1.4046980	0.3408680	0.2079350
C	-2.8903060	-0.4093310	0.6051000
C	-3.4485410	-1.0029360	-0.5323520
C	-4.8133360	-1.2697830	-0.5858870
C	-5.6329860	-0.9473560	0.4933560
C	-5.0841150	-0.3602450	1.6309290
C	-3.7181300	-0.1009120	1.6903620
C	-0.6910520	-2.7598350	-0.5302310
C	-0.3401400	-2.8666470	-1.8835170
C	-0.8249930	-3.9129380	-2.6580480
C	-1.6738960	-4.8655360	-2.0941450
C	-2.0345300	-4.7654480	-0.7526580
C	-1.5488110	-3.7179990	0.0236870
C	2.1584060	-2.0277710	-0.1306770
C	3.0436620	-1.9287080	-1.2100530

C	4.0223040	-2.8952030	-1.4127900
C	4.1336830	-3.9751540	-0.5385820
C	3.2557550	-4.0857660	0.5356190
C	2.2718450	-3.1215280	0.7338110
C	2.8570210	0.7046380	0.3088390
C	3.5279060	1.3869510	-0.7115980
C	4.8767580	1.7007840	-0.5870910
C	5.5769020	1.3429880	0.5636410
C	4.9188270	0.6661010	1.5863810
C	3.5690870	0.3481490	1.4573510
C	-0.9209880	1.1499080	0.1632520
C	0.4358040	1.3994180	0.0996430
C	-1.9197240	2.1847550	-0.2538460
C	-2.6153400	2.0713740	-1.4607990
C	-2.1737440	3.2825360	0.5728610
C	-3.5435920	3.0387160	-1.8355960
H	-2.4200470	1.2240390	-2.1117060
C	-3.0979640	4.2526700	0.1959800
H	-1.6311400	3.3787030	1.5091450
C	-3.7864400	4.1327090	-1.0087210
H	-4.0747030	2.9383500	-2.7771070
H	-3.2804310	5.1033550	0.8451870
H	-4.5080970	4.8884280	-1.3024370
C	0.9099690	2.7956290	-0.1642620
C	0.6943760	3.4158410	-1.3981540
C	1.5748170	3.5059570	0.8401270
C	1.1341490	4.7174010	-1.6230610
H	0.1755460	2.8730250	-2.1830970
C	2.0154660	4.8065790	0.6163730
H	1.7557200	3.0253340	1.7977660
C	1.7962340	5.4163930	-0.6167110
H	0.9584640	5.1859870	-2.5864010
H	2.5313390	5.3432930	1.4064440
H	2.1392730	6.4311330	-0.7922480
H	0.3096120	-2.1145390	-2.3222310
H	-0.5461690	-3.9839360	-3.7046220
H	-2.0549040	-5.6809160	-2.7008500
H	-2.6968770	-5.5022590	-0.3098150
H	-1.8375060	-3.6402940	1.0688840
H	2.9582350	-1.0886990	-1.8925730
H	4.6997070	-2.8055340	-2.2560970
H	3.3338310	-4.9233940	1.2213400
H	1.5844850	-3.2118920	1.5711960
H	4.9004540	-4.7270020	-0.6958720
H	2.9856570	1.6731090	-1.6080690
H	3.0577940	-0.1885730	2.2524440
H	5.4554620	0.3820020	2.4862220
H	6.6292530	1.5906240	0.6607710
H	5.3821170	2.2269870	-1.3909110
H	-2.8090460	-1.2548950	-1.3731190
H	-5.2361680	-1.7301750	-1.4731530
H	-6.6973260	-1.1554090	0.4488990
H	-5.7179830	-0.1050370	2.4740290
H	-3.2855240	0.3599860	2.5744550
Cl	-0.8939730	-1.6864060	3.2273660

3c'

E(M062X/6-31G**) = -2102.996420 a.u.

B	0.0264640	-1.7137390	-1.1188110
C	1.3625250	-1.2947280	-0.4673010
C	1.5896010	-0.0337080	0.0053120
C	0.6899130	1.1157130	-0.1929310
C	-0.6747620	1.1049790	-0.1593900
C	-1.5481820	-0.0622990	0.0384500
C	-1.3088800	-1.3107690	-0.4578650
C	2.3624100	-2.3825280	-0.2218830
C	3.0982140	-2.9458070	-1.2697520
H	2.9517220	-2.5814870	-2.2821390
C	4.0305970	-3.9484950	-1.0179250
H	4.6031720	-4.3675550	-1.8394470
C	4.2298810	-4.4109150	0.2807480
H	4.9552380	-5.1945520	0.4750180
C	3.4934680	-3.8631880	1.3281740
H	3.6419190	-4.2178780	2.3432550
C	2.5665880	-2.8560470	1.0786410
H	1.9992650	-2.4203990	1.8962500
C	2.8600190	0.2947960	0.7300280
C	4.1165920	0.1260270	0.1432470
H	4.1849690	-0.2485620	-0.8736400
C	5.2717750	0.4339130	0.8542980
H	6.2417920	0.3019040	0.3856200
C	5.1853930	0.9090770	2.1610790
H	6.0880900	1.1442870	2.7158370
C	3.9364500	1.0859290	2.7509260
H	3.8611250	1.4580090	3.7678620
C	2.7803510	0.7881950	2.0354920
H	1.8031410	0.9305540	2.4910940
C	1.4098800	2.4033940	-0.4747350
C	2.1476440	2.5090490	-1.6570810
H	2.1771700	1.6656810	-2.3428520
C	2.8346930	3.6809350	-1.9613690
H	3.3962140	3.7516740	-2.8876450
C	2.8016590	4.7571590	-1.0788750
H	3.3399980	5.6702450	-1.3121710
C	2.0802240	4.6544280	0.1084180
H	2.0568810	5.4863300	0.8052820
C	1.3878520	3.4856430	0.4079390
H	0.8280170	3.4068660	1.3347900
C	-1.4185090	2.3841930	-0.4373590
C	-1.6370320	2.7487000	-1.7678440
H	-1.2476520	2.1182990	-2.5629150
C	-2.3407680	3.9102080	-2.0774270
H	-2.4996280	4.1826190	-3.1160720
C	-2.8360890	4.7182040	-1.0583470
H	-3.3856470	5.6230590	-1.2975750
C	-2.6244600	4.3593570	0.2712490
H	-3.0081510	4.9838590	1.0718740
C	-1.9222410	3.1990360	0.5800890
H	-1.7674140	2.9196840	1.6178990
C	-2.8415910	0.2199240	0.7433970
C	-4.0419260	0.3487220	0.0419300
H	-4.0423030	0.2395560	-1.0387370

C	-5.2239790	0.6258110	0.7212270
H	-6.1512880	0.7295940	0.1667930
C	-5.2191430	0.7702370	2.1070060
H	-6.1427920	0.9842470	2.6352610
C	-4.0261200	0.6370170	2.8124630
H	-4.0164720	0.7435290	3.8927570
C	-2.8411980	0.3670320	2.1323540
H	-1.9063930	0.2675700	2.6790880
C	-2.3335690	-2.3879630	-0.2755480
C	-3.1175950	-2.8200250	-1.3502560
H	-2.9826690	-2.3637830	-2.3266230
C	-4.0758070	-3.8132590	-1.1690290
H	-4.6847180	-4.1319240	-2.0092530
C	-4.2541820	-4.3949960	0.0840090
H	-4.9998290	-5.1713090	0.2227650
C	-3.4716610	-3.9763390	1.1569570
H	-3.6040010	-4.4254680	2.1362430
C	-2.5183740	-2.9778440	0.9784890
H	-1.9112670	-2.6452040	1.8157520
Cl	0.0194840	-2.8954920	-2.4536680

2c(*I*Me)’

E(M062X/6-31G**) = -2407.755690 a.u.

B	0.3517640	-0.3582060	-1.4622370
C	1.1439150	0.3633630	-0.1530460
C	0.7586240	-0.6870380	0.9104590
C	-0.5251310	-1.0699420	0.7065970
C	-1.0577190	-0.4064050	-0.5900290
C	2.6032920	0.6333240	-0.3876750
C	3.0801780	1.8667280	-0.8551470
C	4.4230180	2.0424780	-1.1897780
C	5.3265870	0.9916800	-1.0725820
C	4.8690070	-0.2454040	-0.6197730
C	3.5307640	-0.4189100	-0.2860710
C	1.5942520	-1.0729670	2.0710610
C	2.5284690	-0.1900930	2.6337160
C	3.2945680	-0.5573790	3.7359140
C	3.1613230	-1.8248310	4.2952830
C	2.2553390	-2.7237680	3.7359150
C	1.4874890	-2.3540420	2.6380310
C	-1.4015320	-1.6991120	1.7293820
C	-1.4821510	-1.0785830	2.9871520
C	-2.3053130	-1.5807200	3.9884590
C	-3.0722720	-2.7204660	3.7572430
C	-3.0153550	-3.3406460	2.5128300
C	-2.1956400	-2.8327370	1.5078250
C	-2.3433470	-0.9542620	-1.1648090
C	-3.5497810	-0.8887170	-0.4505590
C	-4.7318530	-1.3902200	-0.9846340
C	-4.7403860	-1.9678250	-2.2532550
C	-3.5570520	-2.0337660	-2.9813500
C	-2.3748640	-1.5280480	-2.4411770
C	0.1515520	1.5365250	0.0364940
C	-1.0955430	1.1078010	-0.2226420
C	0.4818180	2.9197750	0.4518770
C	1.1468830	3.1775780	1.6557220
C	0.1121360	4.0051370	-0.3543860

C	1.4451880	4.4821230	2.0401400	H	-3.7156960	-3.1158190	4.5368690
H	1.4060650	2.3490060	2.3069960	H	-3.5606370	-0.4326810	0.5349630
C	0.4107070	5.3085570	0.0293900	H	-1.4604720	-1.5725620	-3.0267100
H	-0.4088910	3.8119710	-1.2878410	H	-3.5497930	-2.4724020	-3.9745560
C	1.0824430	5.5521830	1.2260860	H	-5.6636740	-2.3568060	-2.6714220
H	1.9579620	4.6617850	2.9802120	H	-5.6497310	-1.3245440	-0.4086930
H	0.1183090	6.1365840	-0.6090780	H	2.3976550	2.6974820	-0.9840450
H	1.3175490	6.5693090	1.5235420	H	4.7565290	3.0112560	-1.5492590
C	-2.3218620	1.9309260	-0.1565980	H	6.3715470	1.1311760	-1.3312150
C	-2.6819660	2.5863580	1.0270180	H	5.5566310	-1.0808800	-0.5250590
C	-3.1356270	2.0878810	-1.2871470	H	3.1921150	-1.3952890	0.0511810
C	-3.8220080	3.3835620	1.0789310	C	2.7495790	-2.9172920	-3.0231610
H	-2.0552970	2.4708470	1.9067890	C	1.9432100	-3.7668990	-2.3482880
C	-4.2732620	2.8865390	-1.2344280	C	1.1576350	-1.6825870	-2.0249020
H	-2.8649990	1.5786400	-2.2081700	H	3.6225440	-3.0953010	-3.6300750
C	-4.6212160	3.5361430	-0.0512290	H	1.9622460	-4.8391380	-2.2404320
H	-4.0851380	3.8861740	2.0046620	N	2.2583030	-1.6500890	-2.8195440
H	-4.8892950	3.0019640	-2.1209630	N	0.9746420	-2.9962160	-1.7487720
H	-5.5105840	4.1574600	-0.0108960	C	2.9046400	-0.5053310	-3.4663290
H	2.6729740	0.7883320	2.1908970	H	2.4636910	-0.3460740	-4.4512960
H	4.0046510	0.1508680	4.1514070	H	2.7899160	0.3906150	-2.8666820
H	3.7618430	-2.1125470	5.1523260	H	3.9650380	-0.7410750	-3.5598330
H	2.1466460	-3.7196690	4.1543130	C	-0.0709160	-3.5995570	-0.9351400
H	0.7803840	-3.0647320	2.2218040	H	0.2004100	-3.5619580	0.1197640
H	-0.8848710	-0.1897340	3.1690560	H	-1.0050640	-3.0707230	-1.1028750
H	-2.3484570	-1.0786680	4.9499410	H	-0.1876070	-4.6360220	-1.2537350
H	-3.6185550	-4.2212930	2.3144740	Cl	0.1305720	0.7706510	-3.0019990
H	-2.2020110	-3.3181320	0.5386900				

d-ts1

E(M062X/6-31G**) = -2407.697600 a.u.

B	0.0326380	-0.6193250	1.5205220
C	-1.1838500	-0.0878820	0.6296880
C	-0.7919930	-1.2288540	-0.3208970
C	0.5795460	-1.1598290	-0.5667820
C	1.2626740	0.1271160	-0.3804640
C	-2.5682910	-0.1052640	1.2370440
C	-2.7545370	0.0736660	2.6112320
C	-4.0319100	0.1563510	3.1642170
C	-5.1520830	0.0567300	2.3452910
C	-4.9843220	-0.1221310	0.9733130
C	-3.7081120	-0.1973010	0.4271470
C	-1.6776610	-2.2873370	-0.8518400
C	-1.6456760	-2.5803210	-2.2254830
C	-2.4574620	-3.5685040	-2.7704530
C	-3.3311360	-4.2840950	-1.9545910
C	-3.3848500	-3.9975410	-0.5927270
C	-2.5708180	-3.0103530	-0.0453350
C	1.2628740	-2.3214820	-1.2089180
C	1.9363840	-2.1509260	-2.4238700
C	2.5527450	-3.2298690	-3.0467040
C	2.5181700	-4.4923770	-2.4539870
C	1.8498420	-4.6708170	-1.2456680
C	1.2166950	-3.5927850	-0.6304850
C	2.7031910	0.3047760	-0.1317760
C	3.1766000	1.5728180	0.2888510
C	4.5080180	1.8119090	0.5974700

C	5.4499530	0.7867890	0.5318810
C	5.0136560	-0.4773290	0.1468140
C	3.6802320	-0.7159680	-0.1758510
C	-0.8951710	1.1823070	-0.1919990
C	0.4005860	1.2882050	-0.6073970
C	-1.9889050	2.0644290	-0.6815560
C	-2.2125490	2.2067810	-2.0578820
C	-2.8107350	2.7860640	0.1960550
C	-3.2083020	3.0494810	-2.5417450
H	-1.5877600	1.6469050	-2.7480520
C	-3.8059420	3.6313620	-0.2843070
H	-2.6783840	2.6744320	1.2690800
C	-4.0077690	3.7680430	-1.6559970
H	-3.3614770	3.1421740	-3.6125960
H	-4.4283470	4.1805060	0.4156750
H	-4.7860860	4.4253550	-2.0309220
C	0.8797430	2.4438700	-1.4220730
C	1.7141390	2.2335370	-2.5272470
C	0.5152710	3.7624330	-1.1159020
C	2.1566600	3.2978660	-3.3062030
H	2.0179960	1.2192980	-2.7686340
C	0.9570890	4.8289340	-1.8919490
H	-0.1368120	3.9487090	-0.2673840
C	1.7801850	4.6017680	-2.9927520
H	2.7982380	3.1071160	-4.1611690
H	0.6573570	5.8404500	-1.6350020
H	2.1258650	5.4328400	-3.5994970
H	-0.9737030	-2.0171380	-2.8661660

H	-2.4113980	-3.7750310	-3.8353450
H	-3.9673490	-5.0555500	-2.3771640
H	-4.0640580	-4.5462060	0.0525910
H	-2.6219780	-2.8013420	1.0171530
H	1.9746400	-1.1600510	-2.8690440
H	3.0664020	-3.0865150	-3.9923080
H	1.8128440	-5.6523580	-0.7834140
H	0.6732810	-3.7315490	0.3008190
H	3.0064080	-5.3335020	-2.9360830
H	2.4730490	2.3910910	0.3919960
H	3.4090590	-1.7251110	-0.4525440
H	5.7191830	-1.3021390	0.0904160
H	6.4919210	0.9682440	0.7749030
H	4.8063640	2.8088650	0.9102530
H	-1.8894640	0.1444830	3.2665530
H	-4.1468240	0.2950300	4.2348560
H	-6.1485200	0.1174290	2.7715520
H	-5.8502210	-0.1959420	0.3230210
H	-3.5896480	-0.3151880	-0.6456290
C	3.0526170	0.4893570	3.5258920
C	2.3310470	1.6358800	3.5397410
C	1.1651400	0.1151320	2.3887730
H	4.0230990	0.2536590	3.9323990
H	2.5356420	2.6020800	3.9720760
N	2.3206950	-0.4346600	2.8185200
N	1.1761220	1.3892230	2.8299150
C	2.7570450	-1.8144340	2.6212610
H	2.4082190	-2.4369410	3.4472170

H	2.3589760	-2.1948560	1.6812560
H	3.8454270	-1.8172700	2.5698550
C	0.1515150	2.4062600	2.6215310
H	-0.7763040	1.9294890	2.3144830
H	-0.0014680	2.9403300	3.5608080
H	0.4717500	3.1008140	1.8424260
Cl	-0.3702390	-2.1839260	2.4729260

3c(*I*Me)’

E(M062X/6-31G**) = -2407.761583 a.u.

B	-1.1355550	0.0955510	1.4252910
C	-0.9689390	1.3651960	0.4227520
C	0.1561230	1.4791710	-0.3339600
C	1.2742560	0.5098060	-0.2611190
C	1.1171460	-0.8466910	-0.2419040
C	-0.2074500	-1.5113680	-0.3441410
C	-1.2832180	-1.1515730	0.4050840
C	0.0716130	-0.0192680	2.5125700
N	0.5976500	-1.1212720	3.0848030
C	1.5004800	-0.7706300	4.0669490
H	2.0386660	-1.5128700	4.6343390
C	1.5224670	0.5829880	4.1131530
H	2.0709010	1.2657600	4.7420270
N	0.6315880	1.0260770	3.1579030
C	0.2595300	-2.5079080	2.7795390
H	-0.8121650	-2.5901980	2.6091600
H	0.5370530	-3.1150580	3.6412910
H	0.8019270	-2.8480840	1.8958920
C	0.2599480	2.4287310	2.9904120

H	0.7581790	2.8589730	2.1200650
H	0.5553600	2.9633470	3.8933580
H	-0.8210290	2.5034780	2.8626790
C	-2.0760480	2.3500980	0.2575360
C	-1.8592490	3.7213550	0.4426100
H	-0.8619340	4.0723750	0.6929930
C	-2.8961990	4.6382270	0.2940340
H	-2.7030170	5.6964910	0.4416660
C	-4.1723860	4.2006080	-0.0484140
H	-4.9816320	4.9141650	-0.1680710
C	-4.4026710	2.8388720	-0.2339480
H	-5.3940530	2.4851070	-0.5000030
C	-3.3685450	1.9225040	-0.0732430
H	-3.5610040	0.8614520	-0.2057750
C	0.2734090	2.5115510	-1.4226570
C	1.1657790	3.5882810	-1.3806860
H	1.8207640	3.7133020	-0.5237090
C	1.2238640	4.5050820	-2.4273870
H	1.9210490	5.3357150	-2.3731110
C	0.3932650	4.3617350	-3.5354970
H	0.4395140	5.0783430	-4.3495600
C	-0.4987510	3.2936600	-3.5898850
H	-1.1550810	3.1728510	-4.4462270
C	-0.5559240	2.3792800	-2.5427460
H	-1.2625720	1.5542480	-2.5771190
C	2.6513140	1.0965820	-0.3566960
C	3.2143970	1.7868330	0.7206160
H	2.6637570	1.8512980	1.6558260
C	4.4757830	2.3665270	0.6144260
H	4.9037810	2.8913750	1.4631120
C	5.1870950	2.2722740	-0.5796960
H	6.1676550	2.7291960	-0.6675570
C	4.6351570	1.5854790	-1.6583600
H	5.1836440	1.5066240	-2.5917580
C	3.3781960	0.9986970	-1.5455210
H	2.9422490	0.4673000	-2.3870630
C	2.3320890	-1.7232900	-0.0850400
C	3.1107080	-1.5547660	1.0717440
H	2.8334100	-0.7809100	1.7807760
C	4.2300210	-2.3410770	1.3181110
H	4.8087450	-2.1815670	2.2230820
C	4.6082890	-3.3212200	0.4039190
H	5.4825400	-3.9372830	0.5891960
C	3.8585000	-3.4924300	-0.7548420
H	4.1496700	-4.2412040	-1.4850630
C	2.7330600	-2.7070150	-0.9991920
H	2.1821630	-2.8570560	-1.9188970
C	-0.2976050	-2.5870050	-1.3811730
C	-0.5863350	-3.9115370	-1.0448930
H	-0.7558320	-4.1688640	-0.0027670
C	-0.6686420	-4.8910230	-2.0308590
H	-0.8915480	-5.9169110	-1.7543670
C	-0.4675460	-4.5569230	-3.3680790
H	-0.5343210	-5.3205290	-4.1366510
C	-0.1851480	-3.2367870	-3.7158000
H	-0.0338140	-2.9678760	-4.7567790

C	-0.0953070	-2.2609220	-2.7275580
H	0.1355780	-1.2312060	-2.9922910
C	-2.6131690	-1.7703120	0.1241240
C	-3.3178310	-2.4792400	1.1050680
H	-2.9044650	-2.5663020	2.1049780
C	-4.5475080	-3.0670380	0.8209950
H	-5.0690670	-3.6207140	1.5957130
C	-5.1117790	-2.9372910	-0.4454170
H	-6.0736970	-3.3900120	-0.6645560
C	-4.4345510	-2.2168410	-1.4263130
H	-4.8666840	-2.1025110	-2.4156450
C	-3.1982230	-1.6448850	-1.1439330
H	-2.6680220	-1.0918440	-1.9140370
Cl	-2.6406080	0.2970130	2.6159630

a-ts1’

E(M062X/6-31G**) = -1874.261783 a.u.

B	0.7361080	-0.0548610	1.3007630
C	-1.2967180	-0.8121180	-0.4393430
C	0.1171240	-0.8684020	-0.7059700
C	0.8452630	0.4386130	-0.7703950
C	1.8297860	0.2429140	0.2652950
C	-2.2542300	-1.8904780	-0.7671190
C	-2.2041420	-2.5514940	-2.0013400
C	-3.1223850	-3.5502260	-2.3067450
C	-4.0993200	-3.9085690	-1.3800240
C	-4.1529900	-3.2638790	-0.1456720
C	-3.2377380	-2.2623280	0.1587990
C	0.8203900	-2.1140180	-1.0933330
C	1.7421090	-2.1150770	-2.1493790
C	2.3972950	-3.2866220	-2.5175320
C	2.1449750	-4.4737050	-1.8347020
C	1.2326300	-4.4833320	-0.7800410
C	0.5742520	-3.3159890	-0.4130210
C	0.8610290	1.3236760	-1.9467940
C	1.5529320	2.5425960	-1.8820030
C	1.5028390	3.4346730	-2.9457200
C	0.7476950	3.1314360	-4.0792100
C	0.0440700	1.9306630	-4.1456880
C	0.0982970	1.0309420	-3.0852350
C	3.2747660	0.2989470	0.2138080
C	3.9932720	0.3577860	-0.9957100
C	5.3818800	0.3976800	-0.9935610
C	6.0849160	0.3754950	0.2103640
C	5.3889310	0.3144770	1.4182540
C	4.0019310	0.2758290	1.4202340
C	0.6229000	-0.9266610	2.5869020
C	1.5531640	-1.9444590	2.8612630
C	1.4002280	-2.7824170	3.9611150
C	0.3091370	-2.6169440	4.8145430
C	-0.6300230	-1.6194470	4.5600240
C	-0.4728130	-0.7888170	3.4536130
C	-1.6068570	0.3819440	0.1949770
C	-0.3736190	0.9503890	0.7131650
C	-0.2344750	2.4022170	0.9937500
C	-0.7924230	3.3719370	0.1459230
C	0.5102040	2.8311680	2.1012480

C	-0.6206530	4.7265240	0.4076200
H	-1.3552290	3.0535320	-0.7276150
C	0.6820210	4.1871040	2.3640990
H	0.9478410	2.0914380	2.7681670
C	0.1163860	5.1375950	1.5173280
H	-1.0573480	5.4627460	-0.2598660
H	1.2568970	4.5004250	3.2298040
H	0.2499760	6.1955360	1.7207390
C	-2.9404570	1.0041220	0.2679170
C	-3.3302970	1.7240550	1.4070430
C	-3.8456360	0.8965300	-0.7989800
C	-4.5890890	2.3098580	1.4811050
H	-2.6355240	1.8255340	2.2357100
C	-5.1040380	1.4815570	-0.7233560
H	-3.5475410	0.3583710	-1.6938660
C	-5.4803600	2.1894970	0.4170640
H	-4.8737440	2.8618240	2.3712260
H	-5.7903290	1.3916770	-1.5593990
H	-6.4621500	2.6488350	0.4738300
H	-1.2125640	-0.0160040	3.2521200
H	-1.4822080	-1.4939200	5.2211000
H	0.1899560	-3.2683710	5.6751460
H	2.1275100	-3.5651400	4.1543660
H	2.3979440	-2.0833710	2.1898460
H	-1.4359800	-2.2817230	-2.7207820
H	-3.0736070	-4.0509920	-3.2684460
H	-4.8134410	-4.6907990	-1.6174950
H	-4.9066190	-3.5447620	0.5831490
H	-3.2705400	-1.7627060	1.1228080
H	1.9310120	-1.1937000	-2.6934820
H	3.1056700	-3.2705330	-3.3399930
H	2.6593610	-5.3861560	-2.1191450
H	1.0366130	-5.4032770	-0.2382100
H	-0.1275460	-3.3201530	0.4165710
H	2.1183910	2.7822240	-0.9847720
H	2.0440840	4.3736540	-2.8876300
H	-0.4432740	0.0897790	-3.1346790
H	-0.5462410	1.6934950	-5.0250920
H	0.7064040	3.8320010	-4.9072720
H	3.4533810	0.3758270	-1.9369620
H	3.4542340	0.2314970	2.3579490
H	5.9312870	0.2982700	2.3584280
H	7.1699630	0.4050790	0.2078210
H	5.9198680	0.4427270	-1.9353770

d) Comparative Test of Different Density Functionals

Table S3. Comparison of relative Gibbs free energies (ΔG) and electronic energies (ΔE) (in kcal•mol^{-1}) for all the calculated species using different DFT methods.

Species	M062X-D3[a] ΔG (ΔE)	M062X[b] ΔG (ΔE)	M052X-D3[c] ΔG (ΔE)	ωB97XD[d] ΔG (ΔE)	B3LYP-D3(BJ)[e] ΔG (ΔE)
**2a′**	0.0 (0.0)	0.0 (0.0)	0.0 (0.0)	0.0 (0.0)	0.0 (0.0)
**b-ts-1**	21.9 (23.7)	21.2 (22.2)	16.1 (17.9)	20.1 (21.9)	14.4 (16.5)
**-int1**	20.8 (23.7)	20.8 (23.7)	14.4 (17.4)	18.6 (21.5)	12.1 (14.8)
**b-ts2**	24.3 (27.6)	24.3 (27.6)	16.6 (20.0)	21.7 (25.1)	14.1 (15.5)
**3a′**	6.4 (8.7)	6.5 (8.7)	-0.9 (1.3)	3.9 (6.2)	-10.6 (-5.6)
**a-ts-1**	32.7 (33.7)	33.1 (34.4)	32.1 (33.1)	33.2 (34.2)	27.6 (30.5)
**a-int1**	10.1 (9.5)	10.0 (9.5)	10.1 (9.4)	10.0 (9.4)	12.4 (12.2)
**a-ts2**	39.0 (41.0)	40.0 (41.2)	33.8 (35.0)	37.3 (38.5)	23.9 (25.8)
**a-int2**	30.8 (31.9)	30.9 (32.2)	24.0 (25.1)	28.8 (29.9)	17.0 (18.6)
**a-ts3**	41.8 (44.1)	41.7 (43.8)	37.6 (39.9)	39.9 (42.2)	29.8 (33.0)
**4a′**	-17.5 (-19.4)	-17.5 (-19.3)	-23.9 (-25.7)	-19.6 (-21.4)	-27.0 (-27.7)
**2a(*I*Me)′**	-10.3 (-31.5)	-7.5 (-28.6)	-8.4 (-29.6)	-6.9 (-28.1)	-11.0 (-31.1)
**c-ts1**	17.0 (-1.8)	19.8 (1.0)	13.6 (-5.2)	21.9 (3.1)	10.9 (-7.4)
**c-int1**	-5.8 (-25.6)	-3.0 (-22.8)	-3.6 (-23.4)	-0.8 (-20.6)	/ /
**c-ts2**	-0.2 (-18.9)	2.6 (-16.1)	0.2 (-18.5)	4.7 (-14.0)	/ /
**3a(*I*Me)′**	-20.5 (-37.6)	-17.6 (-34.8)	-21.3 (-38.4)	-17.8 (-34.9)	-27.7 (-44.1)
**2c′**	0.0 (0.0)	0.0 (0.0)	0.0 (0.0)	0.0 (0.0)	0.0 (0.0)
**e-ts1**	26.9 (30.6)	27.0 (30.6)	18.7 (22.4)	22.3 (26.0)	14.6 (16.9)
**3c′**	5.8 (9.3)	5.8 (9.2)	-2.3 (1.2)	0.0 (3.5)	-10.4 (-7.6)
**2c(*I*Me)′**	-22.2 (-41.9)	-19.6 (-39.3)	-21.2 (-40.9)	-19.0 (-38.7)	-22.9 (-41.9)
**d-ts1**	12.2 (-5.4)	14.7 (-2.8)	11.2 (-6.4)	17.1 (-0.5)	5.6 (-12.8)
**3c(*I*Me)′**	-29.0 (-45.6)	-26.5 (-43.0)	-29.1 (-45.7)	-26.7 (-43.2)	-36.0 (-52.7)

[a] This set of data is presented in the main text using M062X-D3/6-31G(d,p)/SMD(toluene). See the computational method section for the detailed basis set and solvation model used for the optimization calculations.

[b] Optimization calculations using M062X/6-31G(d,p)/SMD(toluene) (without D3 dispersion corrections).

[c] Single-point energy calculations using M052X-D3/6-311++G(d,p)/SMD(toluene).

[b] Single-point energy calculations using the ωB97XD/6-311++G(d,p)/SMD(toluene).

[e] Optimization calculations using the B3LYP-D3(BJ)/6-31G(d,p) and single-point energy calculations with B3LYP-D3(BJ)/6-311+G(d,p)/SMD(toluene). **c-int1** and **c-ts2** are not stationary points at this level of theory.

From the results given in Table S3, we conclude the following:

1. Overall, the different DFT methods give approximately consistent results.
2. B3LYP significantly overestimates the stability of **3** and **4** versus **2**, while M05-2X-D3 overestimates it only slightly.

3. The D3 corrections are clearly unimportant on the basis of the results obtained.
4. The slight overestimation of the stability of **3** versus **2** using M052X-D3 (point 2 above) does not create a conflict with our previous findings[23] since the major conclusions of this study do not depend on the relative stability of the two species. However, the current work specifically addresses the relative stability of **3** versus **2**, making an accurate estimation of their relative energies critical. We found that M062X-D3 gives results more consistent with the experimental observations. Moreover, both M062X and ωB97XD give similar results.

F. References

[1] H. Braunschweig, I. Fernández, G. Frenking, T. Kupfer, *Angew. Chem., Int. Ed.* **2008**, *47*, 1951-1954; *Angew. Chem.* **2008**, *120*, 1977-1980.

[2] H. Braunschweig, A. Damme, J. O. C. Jiménez-Halla, C. Hörl, I. Krummenacher, T. Kupfer, L. Mailänder, K. Radacki, *J. Am. Chem. Soc.* **2012**, *134*, 20169-20177.

[3] H. Braunschweig, T. Kupfer, *Chem. Commun.* **2008**, 4487-4489.

[4] H. Braunschweig, J. Maier, K. Radacki, J. Wahler, *Organometallics* **2013**, *32*, 6353-6359.

[5] J. J. Eisch, J. E. Galle, B. Shafii, A. L. Rheingold, *Organometallics* **1990**, *9*, 2342-2349.

[6] H. Braunschweig, C.-W. Chiu, D. Gamon, K. Gruß, C. Hörl, T. Kupfer, K. Radacki, J. Wahler, *Eur. J. Inorg. Chem.* **2013**, 1525-1530.

[7] G. Sheldrick, *Acta Crystallographica Section A* **2015**, *71*, 3-8.

[8] G. Sheldrick, *Acta Crystallographica Section A* **2008**, *64*, 112-122.

[9] M. J. Frisch, G. W. Trucks, H. B. Schlegel, G. E. Scuseria, M. A. Robb, J. R. Cheeseman, G. Scalmani, V. Barone, B. Mennucci, G. A. Petersson, H. Nakatsuji, M. Caricato, X. Li, H. P. Hratchian, A. F. Izmaylov, J. Bloino, G. Zheng, J. L. Sonnenberg, M. Hada, M. Ehara, K. Toyota, R. Fukuda, J. Hasegawa, M. Ishida, T. Nakajima, Y. Honda, O. Kitao, H. Nakai, T. Vreven, J. A. Montgomery, J. E. Peralta, F. Ogliaro, M. Bearpark, J. J. Heyd, E. Brothers, K. N. Kudin, V. N. Staroverov, R. Kobayashi, J. Normand, K. Raghavachari, A. Rendell, J. C. Burant, S.S. Iyengar, J. Tomasi, M. Cossi, N. Rega, J. M. Millam, M. Klene, J. E. Knox, J. B. Cross, V. Bakken, C. Adamo, J. Jaramillo, R. Gomperts, R. E. Stratmann, O. Yazyev, A. J. Austin, R. Cammi, C. Pomelli, J. W. Ochterski, R. L. Martin, K. Morokuma, V. G. Zakrzewski, G. A. Voth, P. Salvador, J. J. Dannenberg, S. Dapprich, A. D. Daniels, O. Farkas, J. B. Foresman, J. V. Ortiz, J. Cioslowski, D. J. Fox, *Gaussian 09*, revision E.01, Gaussian, Inc.: Wallingford, CT, **2016**.

[10] M. D. Hanwell, D. E. Curtis, D. C. Lonie, T. Vandermeersch, E. Zurek, G. R. Hutchison, *J. Cheminf.* **2012**, *4*, 17.

[11] T. Lu, F. Chen, *J. Comput. Chem.* **2012**, *33*, 580-592.

[12] C. Lee, W. Yang, R. G. Parr, *Phys. Rev. B.* **1988**, *37*, 785-789.

[13] a) G. A. Petersson, M. A. Al-Laham, *J. Chem. Phys.* **1991**, *94*, 6081-6090; b) G. A. Petersson, A. Bennett, T. G. Tensfeldt, M. A. Al-Laham, W. A. Shirley, J. Mantzaris, *J. Chem. Phys.* **1988**, *89*, 2193-2218.

[14] Y. Zhao, D. G. Truhlar, *Theor. Chem. Acc.* **2008**, *120*, 215-241.

[15] Y. Zhao, D. G. Truhlar, *J. Chem. Theory Comput.* **2008**, *4*, 1849-1868.

[16] N. Mardirossian, M. Head-Gordon, *J. Chem. Theory Comput.* **2016**, *12*, 4303-4325.

[17] M.-A. Légaré, G. Bélanger-Chabot, R. D. Dewhurst, E. Welz, I. Krummenacher, B. Engels, H. Braunschweig, *Science* **2018**, *359*, 896.

[18] A. V. Marenich, C. J. Cramer, D. G. Truhlar, *J. Phys. Chem. B.* **2009**, *113*, 6378-6396.

[19] S. Grimme, J. Antony, S. Ehrlich, H. Krieg, *J. Chem. Phys.* **2010**, *132*, 154104.

[20] P. C. Hariharan, J. A. Pople, *Theor. Chim. Acta* **1973**, *28*, 213-222.

[21] K. Fukui, *Acc. Chem. Res.* **1981**, *14*, 363-368.

[22] M. J. Frisch, G. W. Trucks, H. B. Schlegel, G. E. Scuseria, M. A. Robb, J. R. Cheeseman, G. Scalmani, V. Barone, B. Mennucci, G. A. Petersson, H. Nakatsuji, M. Caricato, X. Li, H. P. Hratchian, A. F. Izmaylov, J. Bloino, G. Zheng, J. L. Sonnenberg, M. Hada, M. Ehara, K. Toyota, R. Fukuda, J. Hasegawa, M. Ishida, T. Nakajima, Y. Honda, O. Kitao, H. Nakai, T. Vreven, J. A. Montgomery, J. E. Peralta, F. Ogliaro, M. Bearpark, J. J. Heyd, E. Brothers, K. N. Kudin, V. N. Staroverov, R. Kobayashi, J. Normand, K. Raghavachari, A. Rendell, J. C. Burant, S. S. Iyengar, J. Tomasi, M. Cossi, N. Rega, N. J. Millam, M. Klene, J. E. Knox, J. B. Cross, V. Bakken, C. Adamo, J. Jaramillo, R. Gomperts, R. E. Stratmann, O. Yazyev, A. J. Austin, R. Cammi, C. Pomelli, J. W. Ochterski, R. L. Martin, K. Morokuma, V. G. Zakrzewski, G. A. Voth, P. Salvador, J. J. Dannenberg, S. Dapprich, A. D. Daniels, Ö. Farkas, J. B. Foresman, J. V. Ortiz, J. Cioslowski, D. J. Fox, Gaussian 09, Revision D.01, Gaussian, Inc., Wallingford CT, 2013.

[23] Z. Wang, Y. Zhou, J.-X. Zhang, I. Krummenacher, H. Braunschweig, Z. Lin, *Chem. Eur. J.* **2018**, *24*, 9612-9621.
